# Archaeology in space: The Sampling Quadrangle Assemblages Research Experiment (SQuARE) on the International Space Station. Report 1: Squares 03 and 05

**DOI:** 10.1371/journal.pone.0304229

**Published:** 2024-08-07

**Authors:** Justin St. P. Walsh, Shawn Graham, Alice C. Gorman, Chantal Brousseau, Salma Abdullah

**Affiliations:** 1 Department of Art, Chapman University, Orange, CA, United States of America; 2 Space Engineering Research Center, University of Southern California, Marina del Rey, CA, United States of America; 3 Department of History, Carleton University, Ottawa, ON, United States of America; 4 College of Humanities, Arts and Social Sciences, Flinders University, Adelaide, Australia; 5 Archaeology Research Center, University of Southern California, Los Angeles, CA, United States of America; University of California Santa Cruz, UNITED STATES OF AMERICA

## Abstract

Between January and March 2022, crew aboard the International Space Station (ISS) performed the first archaeological fieldwork in space, the Sampling Quadrangle Assemblages Research Experiment (SQuARE). The experiment aimed to: (1) develop a new understanding of how humans adapt to life in an environmental context for which we are not evolutionarily adapted, using evidence from the observation of material culture; (2) identify disjunctions between planned and actual usage of facilities on a space station; (3) develop and test techniques that enable archaeological research at a distance; and (4) demonstrate the relevance of social science methods and perspectives for improving life in space. In this article, we describe our methodology, which involves a creative re-imagining of a long-standing sampling practice for the characterization of a site, the shovel test pit. The ISS crew marked out six sample locations (“squares”) around the ISS and documented them through daily photography over a 60-day period. Here we present the results from two of the six squares: an equipment maintenance area, and an area near exercise equipment and the latrine. Using the photographs and an innovative webtool, we identified 5,438 instances of items, labeling them by type and function. We then performed chronological analyses to determine how the documented areas were actually used. Our results show differences between intended and actual use, with storage the most common function of the maintenance area, and personal hygiene activities most common in an undesignated area near locations for exercise and waste.

## Introduction

The International Space Station Archaeological Project (ISSAP) aims to fill a gap in social science investigation into the human experience of long-duration spaceflight [1–3]. As the largest, most intensively inhabited space station to date, with over 270 visitors from 23 countries during more than 23 years of continuous habitation, the International Space Station (ISS) is the ideal example of a new kind of spacefaring community—“a microsociety in a miniworld” [4]. While it is possible to interview crew members about their experiences, the value of an approach focused on material culture is that it allows identification of longer-term patterns of behaviors and associations that interlocutors are unable or even unwilling to articulate. In this respect, we are inspired by previous examples of contemporary archaeology such as the Tucson Garbage Project and the Undocumented Migration Project [5–7]. We also follow previous discussions of material culture in space contexts that highlight the social and cultural features of space technology [8, 9].

Our primary goal is to identify how humans adapt to life in a new environment for which our species has not evolved, one characterized by isolation, confinement, and especially microgravity. Microgravity introduces opportunities, such as the ability to move and work in 360 degrees, and to carry out experiments impossible in full Earth gravity, but also limitations, as unrestrained objects float away. The most routine activities carried out on Earth become the focus of intense planning and technological intervention in microgravity. By extension, our project also seeks to develop archaeological techniques that permit the study of other habitats in remote, extreme, or dangerous environments [10, 11]. Since it is too costly and difficult to visit our archaeological site in person, we have to creatively re-imagine traditional archaeological methods to answer key questions. To date, our team has studied crew-created visual displays [12, 13], meanings and processes associated with items returned to Earth [14], distribution of different population groups around the various modules [15], and the development of machine learning (ML) computational techniques to extract data about people and places, all from historic photographs of life on the ISS [16].

From January to March 2022, we developed a new dataset through the first archaeological work conducted off-Earth. We documented material culture in six locations around the ISS habitat, using daily photography taken by the crew which we then annotated and studied as evidence for changes in archaeological assemblages of material culture over time. This was the first time such data had been captured in a way that allowed statistical analysis. Here, we present the data and results from Squares 03 and 05, the first two sample locations to be completed.

## Materials and methods

### SQuARE concept and planning

Gorman proposed the concept behind the investigation, deriving it from one of the most traditional terrestrial archaeological techniques, the shovel test pit. This method is used to understand the overall characteristics of a site quickly through sampling. A site is mapped with a grid of one-meter squares. Some of the squares are selected for initial excavation to understand the likely spatial and chronological distribution of features across the entire site. In effect, the technique is a way to sample a known percentage of the entire site systematically. In the ISS application of this method, we documented a notional stratigraphy through daily photography, rather than excavation.

Historic photography is a key dataset for the International Space Station Archaeological Project. Tens of thousands of images have been made available to us, either through publication [17], or through an arrangement with the ISS Research Integration Office, which supplied previously unpublished images from the first eight years of the station’s habitation. These photographs are informative about the relationships between people, places, and objects over time in the ISS. However, they were taken randomly (from an archaeological perspective) and released only according to NASA’s priorities and rules. Most significantly, they were not made with the purpose of answering archaeological questions. By contrast, the photographs taken during the present investigation were systematic, representative of a defined proportion of the habitat’s area, and targeted towards capturing archaeology’s primary evidence: material culture. We were interested in how objects move around individual spaces and the station, what these movements revealed about crew adherence to terrestrial planning, and the creative use of material culture to make the laboratory-like interior of the ISS more habitable.

Access to the field site was gained through approval of a proposal submitted to the Center for the Advancement of Science in Space (also known as the ISS National Laboratory [ISS NL]). Upon acceptance, Axiom Space was assigned as the Implementation Partner for carriage of the experiment according to standard procedure. No other permits were required for this work.

### Experiment design

Since our work envisioned one-meter sample squares, and recognizing the use of acronyms as a persistent element of spacefaring culture, we named our payload the Sampling Quadrangle Assemblages Research Experiment (SQuARE). Permission from the ISS NL to conduct SQuARE was contingent on using equipment that was already on board the space station. SQuARE required only five items: a camera, a wide-angle lens, adhesive tape (for marking the boundaries of the sample locations), a ruler (for scale), and a color calibration card (for post-processing of the images). All of these were already present on the ISS.

Walsh performed tests on the walls of a terrestrial art gallery to assess the feasibility of creating perfect one-meter squares in microgravity. He worked on a vertical surface, using the Pythagorean theorem to determine where the corners should be located. The only additional items used for these tests were two metric measuring tapes and a pencil for marking the wall (these were also already on the ISS). While it was possible to make a square this way, it also became clear that at least two people were needed to manage holding the tape measures in position while marking the points for the corners. This was not possible in the ISS context.

Walsh and Gorman identified seven locations for the placement of squares. Five of these were in the US Orbital Segment (USOS, consisting of American, European, and Japanese modules) and two in the Russian Orbital Segment. Unfortunately, tense relations between the US and Russian governments meant we could only document areas in the USOS. The five locations were (with their SQuARE designations):

01—an experimental rack on the forward wall, starboard end, of the Japanese Experiment Module
02—an experimental rack on the forward wall, port end, of the European laboratory module Columbus
03—the starboard Maintenance Work Area (workstation) in the US Node 2 module
04—the wall area “above” (according to typical crew body orientation) the galley table in the US Node 1 module
05—the aft wall, center location, of the US Node 3 module

Our square selection encompassed different modules and activities, including work and leisure. We also asked the crew to select a sixth sample location based on their understanding of the experiment and what they thought would be interesting to document. They chose a workstation on the port wall of the US laboratory module, at the aft end, which they described in a debriefing following their return to Earth in June 2022 as “our central command post, like our shared office situation in the lab.” Results from the four squares not included here will appear in future publications.

Walsh worked with NASA staff to determine payload procedures, including precise locations for the placement of the tape that would mark the square boundaries. The squares could not obstruct other facilities or experiments, so (unlike in terrestrial excavations, where string is typically used to demarcate trench boundaries) only the corners of each square were marked, not the entire perimeter. We used Kapton tape due to its bright yellow-orange color, which aided visibility for the crew taking photographs and for us when cropping the images. In practice, due to space constraints, the procedures that could actually be performed by crew in the ISS context, and the need to avoid interfering with other ongoing experiments, none of the locations actually measured one square meter or had precise 90° corners like a trench on Earth.

On January 14, 2022, NASA astronaut Kayla Barron set up the sample locations, marking the beginning of archaeological work in space (S1 Movie). For 30 days, starting on January 21, a crew member took photos of the sample locations at approximately the same time each day; the process was repeated at a random time each day for a second 30-day period to eliminate biases. Photography ended on March 21, 2022. The crew were instructed not to move any items prior to taking the photographs. Walsh led image management, including color and barrel distortion correction, fixing the alignment of each image, and cropping them to the boundaries of the taped corners.

### Data processing—Item tagging, statistics, visualizations

We refer to each day’s photo as a “context” by analogy with chronologically-linked assemblages of artifacts and installations at terrestrial archaeological sites (S1 and S2 Datasets). As previously noted, each context represented a moment roughly 24 hours distant from the previous one, showing evidence of changes in that time. ISS mission planners attempted to schedule the activity at the same time in the first month, but there were inevitable changes due to contingencies. Remarkably, the average time between contexts in Phase 1 was an almost-perfect 24h 0m 13s. Most of the Phase 1 photos were taken between 1200 and 1300 GMT (the time zone in which life on the ISS is organized). In Phase 2, the times were much more variable, but the average time between contexts during this period was still 23h 31m 45s. The earliest Phase 2 photo was taken at 0815 GMT, and the latest at 2101. We did not identify any meaningful differences between results from the two phases.

Since the “test pits” were formed of images rather than soil matrices, we needed a tool to capture information about the identity, nature, and location of every object. An open-source image annotator platform [18] mostly suited our needs. Brousseau rebuilt the platform to work within the constraints of our access to the imagery (turning it into a desktop tool with secure access to our private server), to permit a greater range of metadata to be added to each item or be imported, to autosave, and to export the resulting annotations. The tool also had to respect privacy and security limitations required by NASA.

The platform Brousseau developed and iterated was rechristened “Rocket-Anno” (S1 File). For each context photograph, the user draws an outline around every object, creating a polygon; each polygon is assigned a unique ID and the user provides the relevant descriptive information, using a controlled vocabulary developed for ISS material culture by Walsh and Gorman. Walsh and Abdullah used Rocket-Anno to tag the items in each context for Squares 03 and 05. Once all the objects were outlined for every context’s photograph, the tool exported a JSON file with all of the metadata for both the images themselves and all of the annotations, including the coordinate points for every polygon (S3 Dataset). We then developed Python code using Jupyter “notebooks” (an interactive development environment) that ingests the JSON file and generates dataframes for various facets of the data. Graham created a “core” notebook that exports summary statistics, calculates Brainerd-Robinson coefficients of similarity, and visualizes the changing use of the square over time by indicating use-areas based on artifact types and subtypes (S2 File). Walsh and Abdullah also wrote detailed square notes with context-by-context discussions and interpretations of features and patterns.

We asked NASA for access to the ISS Crew Planner, a computer system that shows each astronaut’s tasks in five-minute increments, to aid with our interpretation of contexts, but were denied. As a proxy, we use another, less detailed source: the ISS Daily Summary Reports (DSRs), published on a semi-regular basis by NASA on its website [19]. Any activities mentioned in the DSRs often must be connected with a context by inference. Therefore, our conclusions are likely less precise than if we had seen the Crew Planner, but they also more clearly represent the result of simply observing and interpreting the material culture record.

## Results

The crew during our sample period formed ISS Expedition 66 (October 2021-March 2022). They were responsible for the movement of objects in the sample squares as they carried out their daily tasks. The group consisted of two Russians affiliated with Roscosmos (the Russian space agency, 26%), one German belonging to the European Space Agency (ESA, 14%), and four Americans employed by NASA (57%). There were six men (86%) and one woman (14%), approximately equivalent to the historic proportions in the ISS population (84% and 16%, respectively). The Russian crew had their sleeping quarters at the aft end of the station, in the Zvezda module. The ESA astronaut slept in the European Columbus laboratory module. The four NASA crew slept in the US Node 2 module (see below). These arrangements emphasize the national character of discrete spaces around the ISS, also evident in our previous study of population distributions [15]. Both of the sample areas in this study were located in US modules.

### Square 03

**Fig 1:**
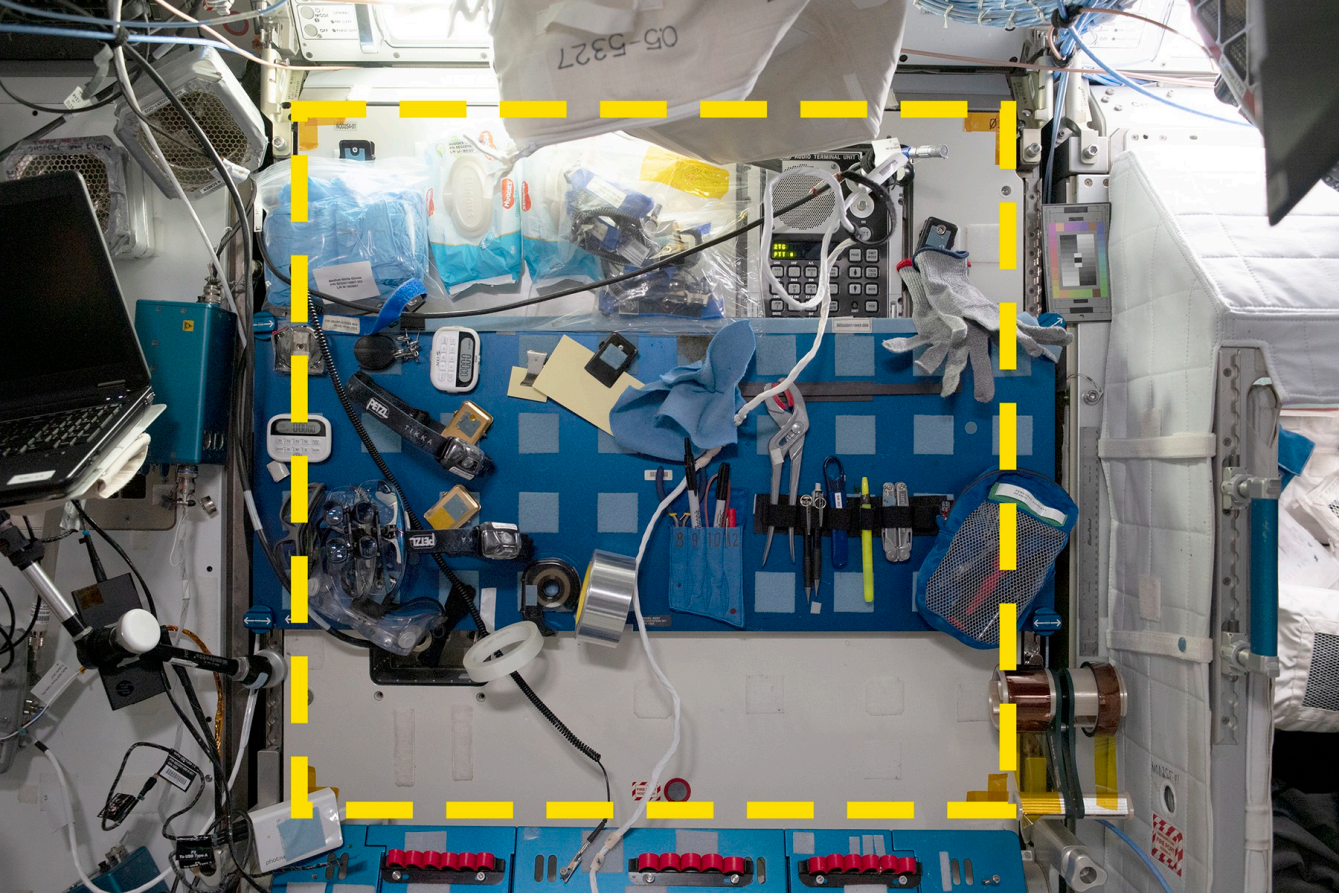
A sample location from the Sampling Quadrangle Assemblages Research Experiment (SQuARE), Square 03 in the starboard Maintenance Work Area of the International Space Station. An open crew berth is visible at right. The yellow dotted line indicates the boundaries of the sample area. Credit: NASA/ISSAP.

**Fig 2:**
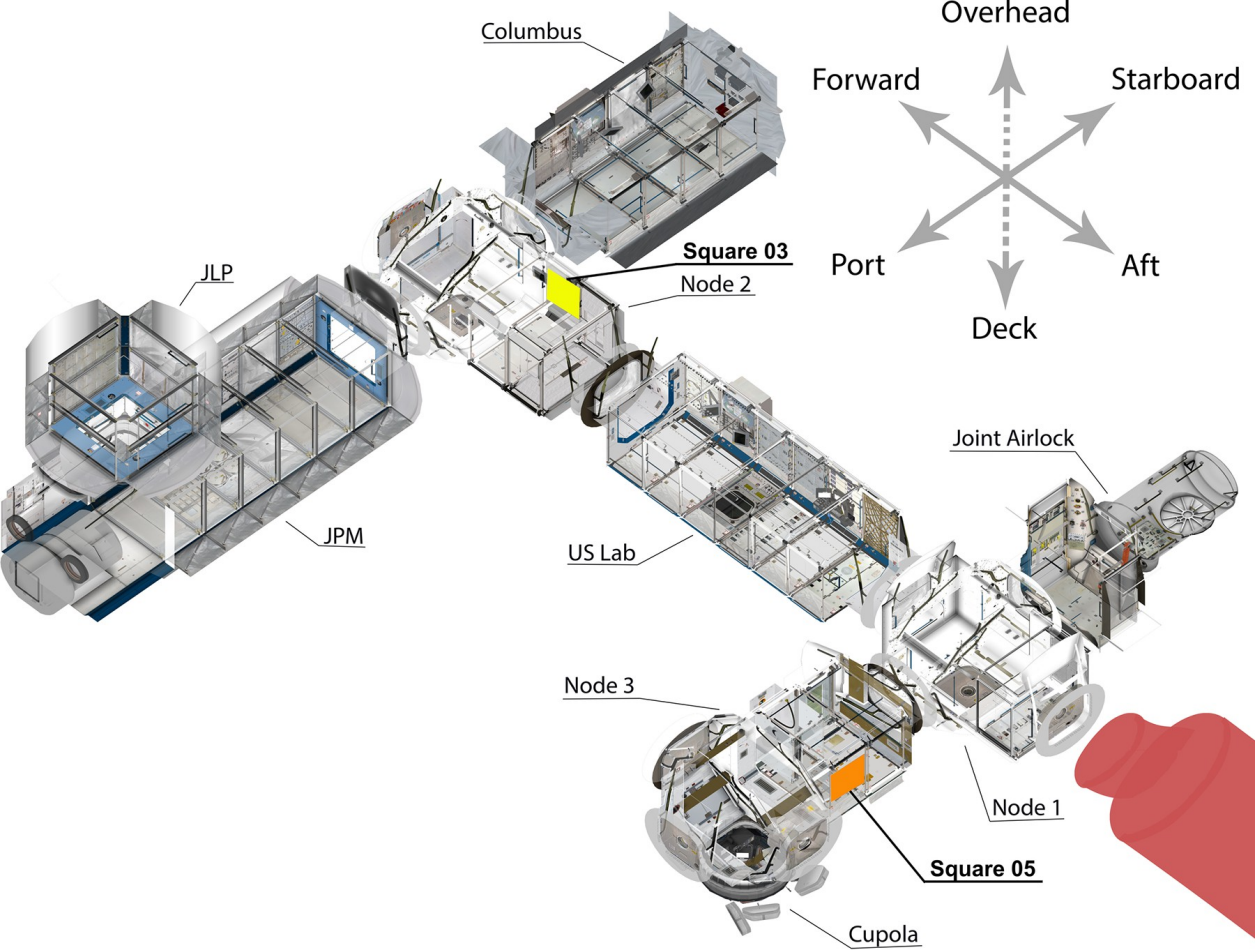
A cutaway image of the International Space Station’s US Orbital Segment, showing the locations of Square 03 (at upper center, in yellow) and 05 (at lower right, in orange). Credit: Tor Finseth, by permission, modified by Justin Walsh.

Square 03 was placed in the starboard Maintenance Work Area (MWA, Fig 1), one of a pair of workstations located opposite one another in the center of the Node 2 module, with four crew berths towards the aft and a series of five ports for the docking of visiting crew/cargo vehicles and two modules on the forward end (Fig 2). Node 2 (sometimes called “Harmony”) is a connector that links the US, Japanese, and European lab modules. According to prevailing design standards when the workstation was developed, an MWA “shall serve as the primary location for servicing and repair of maximum sized replacement unit/system components” [20]. Historic images published by NASA showing its use suggested that its primary function was maintenance of equipment and also scientific work that did not require a specific facility such as a centrifuge or furnace.

Square 03 measured 90.3 cm (top) x 87.8 (left) x 89.4 (bottom) x 87.6 (right), for an area of approximately 0.79 m^2^. Its primary feature was a blue metal panel with 40 square loop-type Velcro patches arranged in four rows of ten. During daily photography, many items were attached to the Velcro patches (or held by a clip or in a resealable bag which had its own hook-type Velcro). Above and below the blue panel were additional Velcro patches placed directly on the white plastic wall surface. These patches were white, in different sizes and shapes and irregularly arranged, indicating that they had been placed on the wall in response to different needs. Some were dirty, indicating long use. The patches below the blue panel were rarely used during the sample period, but the patches above were used frequently to hold packages of wet wipes, as well as resealable bags with electrostatic dispersion kits and other items. Outside the sample area, the primary features were a crew berth to the right, and a blue metal table attached to the wall below. This table, the primary component of the MWA, “provides a rigid surface on which to perform maintenance tasks,” according to NASA [21]. It is modular and can be oriented in several configurations, from flat against the wall to horizontal (*i*.*e*., perpendicular to the wall). A laptop to the left of the square occasionally showed information about work happening in the area.

**Fig 3:**
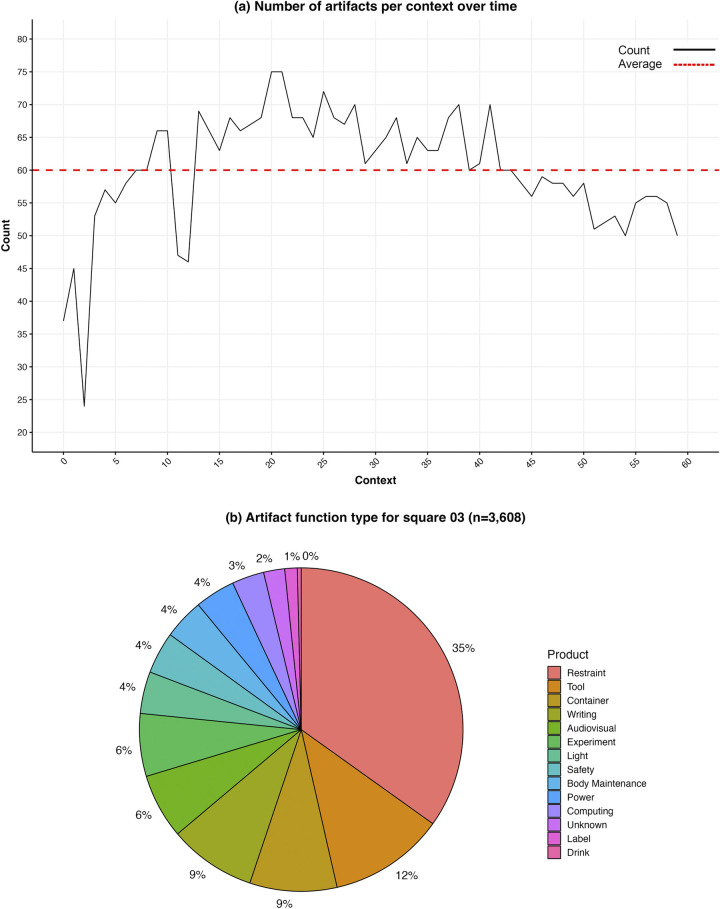
(a) Count of artifacts in Square 03 over time. (b) Proportions of artifacts by function in Square 03. Credit: Rao Hamza Ali.

In the 60 context photos of Square 03, we recorded 3,608 instances of items, an average of 60.1 (median = 60.5) per context. The lowest count was 24 in context 2 (where most of the wall was hidden from view behind an opaque storage bag), and the highest was 75 in both contexts 20 and 21. For comparison between squares, we can also calculate the item densities per m^2^. The average count was 76.1/m^2^ (minimum = 30, maximum = 95). The count per context (Fig 3(A)) began much lower than average in the first three contexts because of a portable glovebag and a stowage bag that obscured much of the sample square. It rose to an above-average level which was sustained (with the exception of contexts 11 and 12, which involved the appearance of another portable glovebag) until about context 43, when the count dipped again and the area seemed to show less use. Contexts 42–59 showed below-average numbers, as much as 20% lower than previously.

74 types of items appeared at least once here, belonging to six categories: equipment (41%), office supplies (31%), electronic (17%), stowage (9%), media (1%), and food (<1%). To better understand the significance of various items in the archaeological record, we assigned them to functional categories (Table 1, Fig 3(B)). 35% of artifacts were restraints, or items used for holding other things in place; 12% for tools; 9% for containers; 9% for writing items; 6% for audiovisual items; 6% for experimental items; 4% for lights; 4% for safety items; 4% for body maintenance; 4% for power items; 3% for computing items; 1% for labels; and less than 1% drinks. We could not identify a function for two percent of the items.

**Table 1:** Proportions of items in various functional categories for Square 03.

Functional categories	Square 03 percentage
Restraint (clips, bungee cords, adhesive tape, cable ties, carabiners)	35%
Tool (screwdrivers, pliers, box cutters, measuring tape)	12%
Container (resealable bags, cargo transfer bags, mesh bags)	9%
Writing implement (pens, markers, notepads)	9%
Audiovisual (microphones, headphones, cables, video cameras)	6%
Experiment (portable glovebags, loupes for magnification, digital timers)	6%
Light (lamps for video recording, headlamps)	4%
Safety (nitrile gloves, safety goggles and glasses)	4%
Body maintenance (dry and wet wipes)	4%
Power (electrostatic dispersion kits, power cables)	4%
Computing (laptops, USB hubs and cables, digital media readers)	3%
Label	1%
Drink (water pouches)	1%

One of the project goals is understanding cultural adaptations to the microgravity environment. We placed special attention on “gravity surrogates,” pieces of (often simple) technology that are used in space to replicate the terrestrial experience of things staying where they are placed. Gravity surrogates include restraints and containers. It is quite noticeable that gravity surrogates comprise close to half of all items (44%) in Square 03, while the tools category, which might have been expected to be most prominent in an area designated for maintenance, is less than one-third as large (12%). Adding other groups associated with work, such as “experiment” and “light,” only brings the total to 22%.

### Square 05

**Fig 4:**
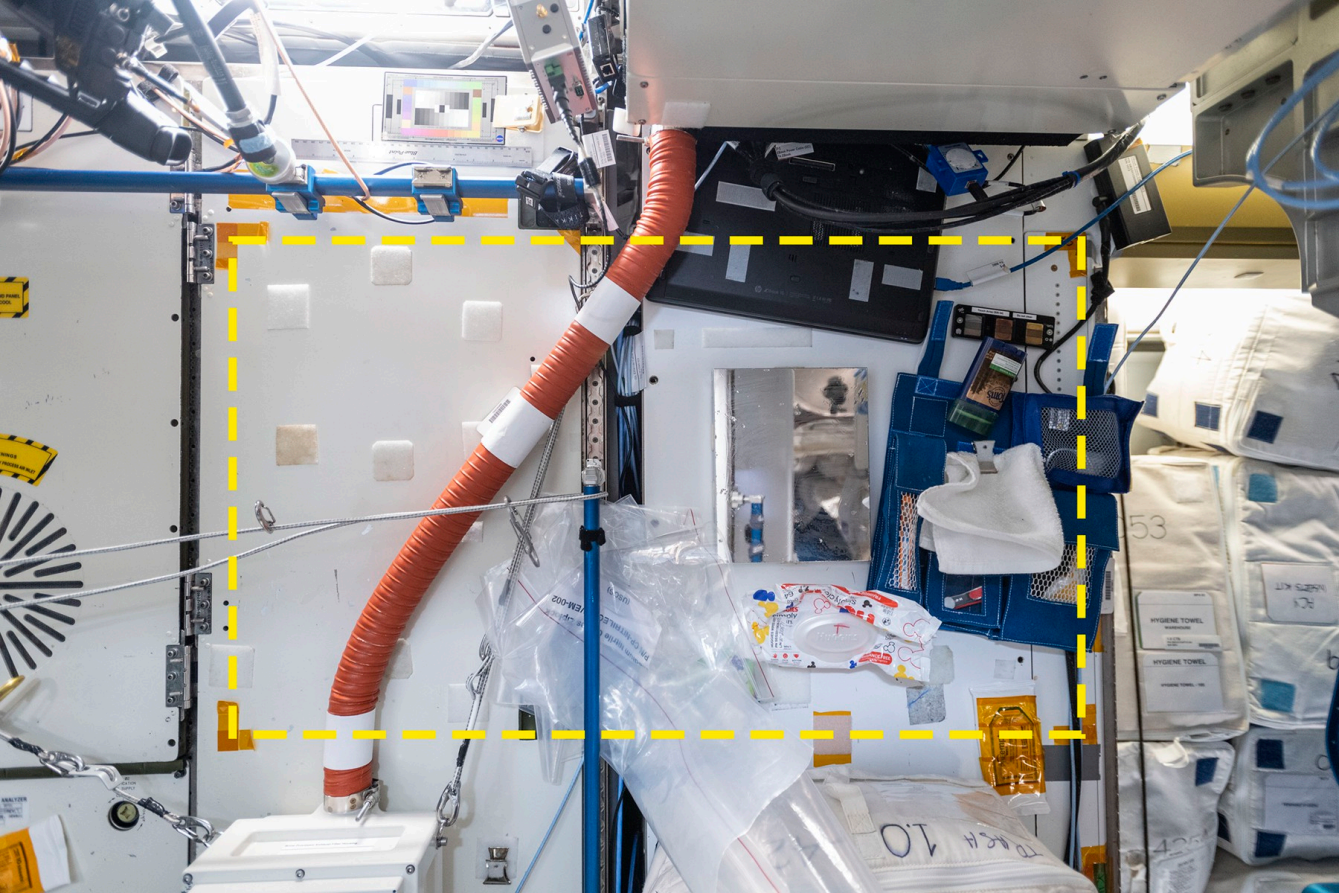
Square 05 on the aft wall of the Node 3 module of the International Space Station. The yellow dotted line indicates the boundaries of the sample area. The ARED machine is at the far upper right, on the overhead wall. The TVIS treadmill is outside this image to the left, on the forward wall. The WHC is directly behind the photographer. Credit: NASA/ISSAP.

Square 05 (Figs 2 and 4) was placed in a central location on the aft wall of the multipurpose Node 3 (“Tranquility”) module. This module does not include any specific science facilities. Instead, there are two large pieces of exercise equipment, the TVIS (Treadmill with Vibration Isolation Stabilization System, on the forward wall at the starboard end), and the ARED (Advanced Resistive Exercise Device, on the overhead wall at the port end). Use of the machines forms a significant part of crew activities, as they are required to exercise for two hours each day to counteract loss of muscle mass and bone density, and enable readjustment to terrestrial gravity on their return. The Waste and Hygiene Compartment (WHC), which includes the USOS latrine, is also here, on the forward wall in the center of the module, opposite Square 05. Finally, three modules are docked at Node 3’s port end. Most notable is the Cupola, a kind of miniature module on the nadir side with a panoramic window looking at Earth. This is the most popular leisure space for the crew, who often describe the hours they spend there. The Permanent Multipurpose Module (PMM) is docked on the forward side, storing equipment, food, and trash. In previous expeditions, some crew described installing a curtain in the PMM to create a private space for changing clothes and performing body maintenance activities such as cleaning oneself [22, 23], but it was unclear whether that continued to be its function during the expedition we observed. One crew member during our sample period posted a video on Instagram showing the PMM interior and their efforts to re-stow equipment in a bag [24]. The last space attached to Node 3 is an experimental inflatable module docked on the aft side, called the Bigelow Expandable Activity Module (BEAM), which is used for storage of equipment.

Square 05 was on a mostly featureless wall, with a vertical handrail in the middle. Handrails are metal bars located throughout the ISS that are used by the crew to hold themselves in place or provide a point from which to propel oneself to another location. NASA’s most recent design standards acknowledge that “[t]hey also serve as convenient locations for temporary mounting, affixing, or restraint of loose equipment and as attachment points for equipment” [25]. The handrail in Square 05 was used as an impromptu object restraint when a resealable bag filled with other bags was squeezed between the handrail and the wall.

The Brine Processing Assembly (BPA), a white plastic box which separates water from other components of urine for treatment and re-introduction to the station’s drinkable water supply [26], was fixed to the wall outside the square boundaries at lower left. A bungee cord was attached to both sides of the box; the one on the right was connected at its other end to the handrail attachment bracket. Numerous items were attached to or wedged into this bungee cord during the survey, bringing “gravity” into being. A red plastic duct ran through the square from top center into the BPA. This duct led from the latrine via the overhead wall. About halfway through the survey period, in context 32, the duct was wrapped in Kapton tape. According to the DSR for that day, “the crew used duct tape [*sic*] to make a seal around the BPA exhaust to prevent odor permeation in the cabin” [27], revealing an aspect of the crew’s experience of this area that is captured only indirectly in the context photograph. Permanently attached to the wall were approximately 20 loop-type Velcro patches in many shapes and sizes, placed in a seemingly random pattern that likely indicates that they were put there at different times and for different reasons.

Other common items in Square 05 were a mirror, a laptop computer, and an experimental item belonging to the German space agency DLR called the Touch Array Assembly [28]. The laptop moved just three times, and only by a few centimeters each time, during the sample period. The Touch Array was a black frame enclosing three metal surfaces which were being tested for their bacterial resistance; members of the crew touched the surfaces at various moments during the sample period. Finally, and most prominent due to its size, frequency of appearance, and use (judged by its movement between context photos) was an unidentified crew member’s toiletry kit.

By contrast with Square 03, 05 was the most irregular sample location, roughly twice as wide as it was tall. Its dimensions were 111 cm (top) x 61.9 (left) x 111.4 (bottom) x 64.6 (right), for an area of approximately 0.7 m^2^, about 89% of Square 03. We identified 1,830 instances of items in the 60 contexts, an average of 30.5 (median = 32) per context. The minimum was 18 items in context 5, and the maximum was 39 in contexts 24, 51, and 52. The average item density was 43.6/m^2^ (minimum = 26, maximum = 56), 57% of Square 03.

**Fig 5:**
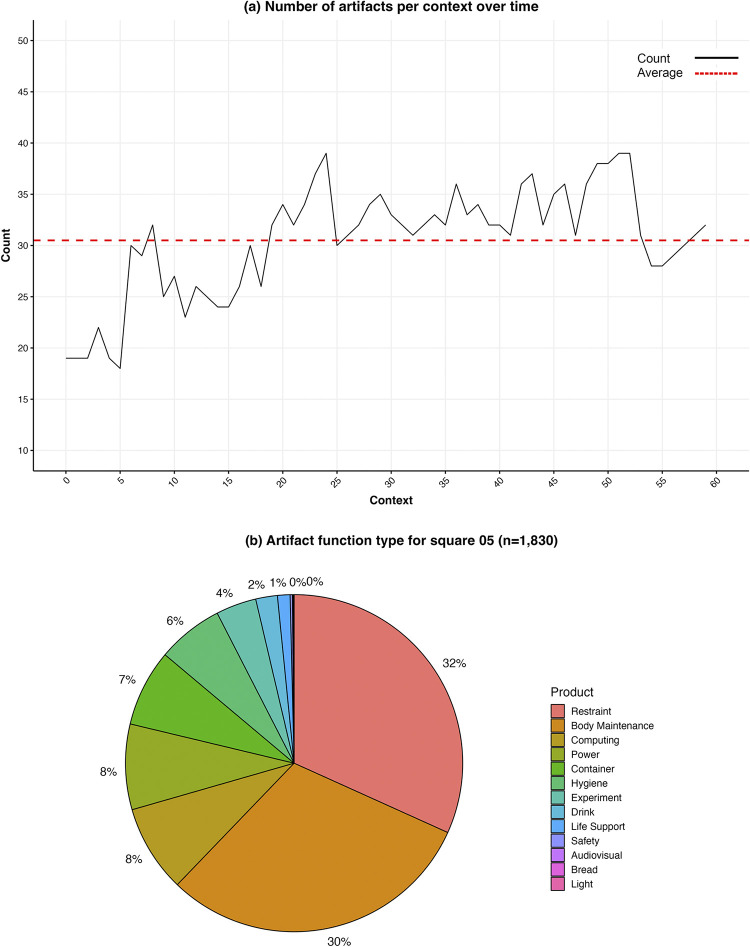
(a) Count of artifacts and average count in Square 05 over time. (b) Proportions of artifacts by function in Square 05. Credit: Rao Hamza Ali.

The number of items trended upward throughout the sample period (Fig 5(A)). The largest spike occurred in context 6 with the appearance of the toiletry kit, which stored (and revealed) a number of related items. The kit can also be linked to one of the largest dips in item count, seen from contexts 52 to 53, when it was closed (but remained in the square). Other major changes can often be attributed to the addition and removal of bungee cords, which had other items such as carabiners and brackets attached. For example, the dip seen in context 25 correlates with the removal of a bungee cord with four carabiners.

41 different item types were found in Square 05, about 55% as many as in Square 03. These belonged to five different categories: equipment (63%), electronic (17%), stowage (10%), office supplies (5%), and food (2%). The distribution of function proportions was quite different in this sample location (Table 2 and Fig 5(B)). Even though restraints were still most prominent, making up 32% of all items, body maintenance was almost as high (30%), indicating how strongly this area was associated with the activity of cleaning and caring for oneself. Computing (8%, represented by the laptop, which seems not to have been used), power (8%, from various cables), container (7%, resealable bags and Cargo Transfer Bags), and hygiene (6%, primarily the BPA duct) were the next most common items. Experiment was the function of 4% of the items, mostly the Touch Array, which appeared in every context, followed by drink (2%) and life support (1%). Safety, audiovisual, food, and light each made up less than 1% of the functional categories.

**Table 2:** Proportions of items in various functional categories for Square 05.

Functional categories	Square 05 percentage
Restraints	32%
Body maintenance	30%
Computing	8%
Power	8%
Container	7%
Hygiene (human waste treatment facilities)	6%
Experiment	4%
Drink	2%
Life support (equipment associated with the ISS oxygen generation system)	1%

## Discussion

Tracking changes over time is critical to understanding the activity happening in each area. We now explore how the assemblages change by calculating the Brainerd-Robinson Coefficient of Similarity [29, 30] as operationalized by Peeples [31, 32]. This metric is used in archaeology for comparing all pairs of the contexts by the proportions of categorical artifact data, here functional type. Applying the coefficient to the SQuARE contexts enables identification of time periods for distinct activities using artifact function and frequency alone, independent of documentary or oral evidence.

### Square 03

**Fig 6:**
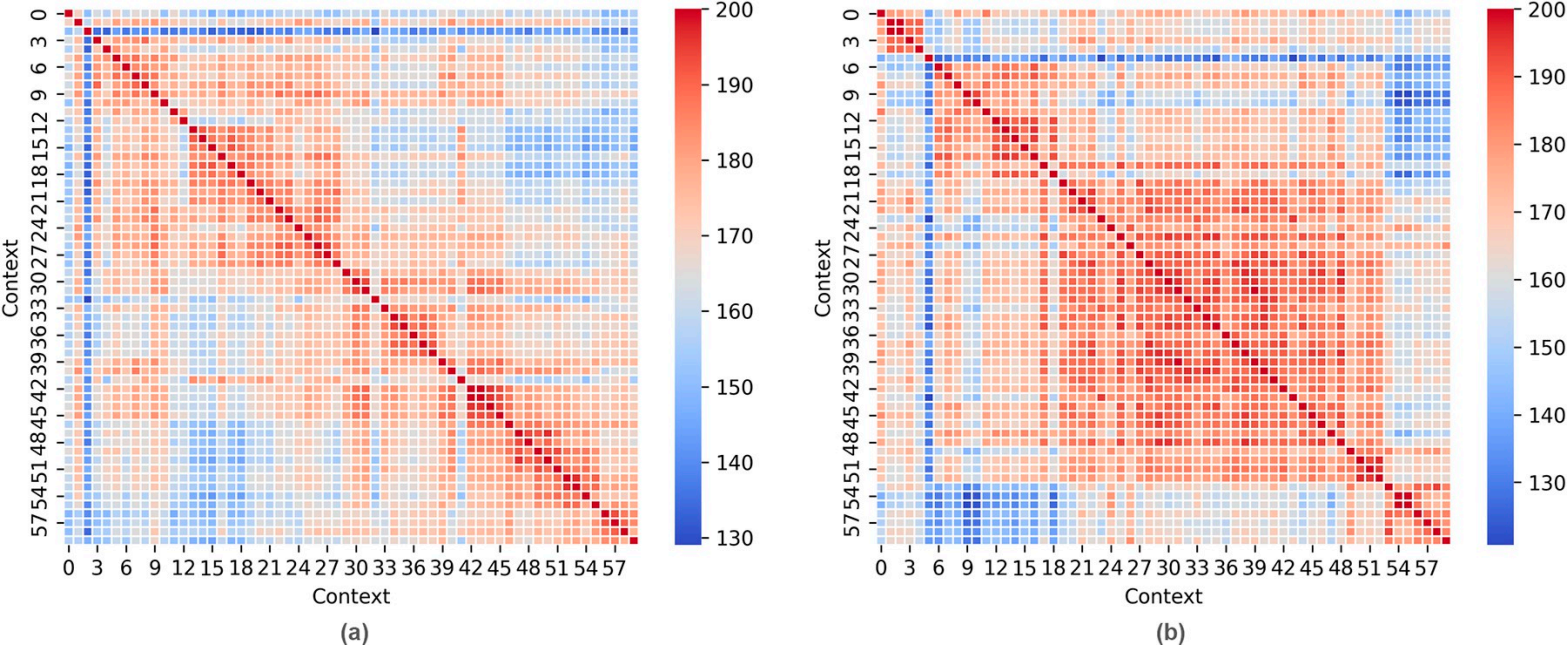
Visualization of Brainerd-Robinson similarity, compared context-by-context by item function, for (a) Square 03 and (b) Square 05. The more alike a pair of contexts is, the higher the coefficient value, with a context compared against itself where a value of 200 equals perfect similarity. The resulting matrix of coefficients is visualized on a scale from blue to red where blue is lowest and red is highest similarity. The dark red diagonal line indicates complete similarity, where each context is compared to itself. Dark blue represents a complete difference. Credit: Shawn Graham.

Multiple phases of activities took place in the square. Moments of connected activity are visible as red clusters in contexts 0–2, 11–12, 28–32, and 41 (Fig 6(A)). Combining this visualization with close observation of the photos themselves, we argue that there are actually eight distinct chronological periods.

1. Contexts 0–2: Period 1 (S1 Fig in S3 File) is a three-day period of work involving a portable glovebag (contexts 0–1) and a large blue stowage bag (context 2). It is difficult to describe trends in functional types because the glovebag and stowage bag obstruct the view of many objects. Items which appear at the top of the sample area, such as audiovisual and body maintenance items, are overemphasized in the data as a result. It appears that some kind of science is happening here, perhaps medical sample collection due to the presence of several small resealable bags visible in the glovebag. The work appears particularly intense in context 1, with the positioning of the video camera and light to point into the glovebag. These items indicate observation and oversight of crew activities by ground control. A white cargo transfer bag for storage and the stowage bag for holding packing materials in the context 2 photo likely relate to the packing of a Cargo Dragon vehicle that was docked to Node 2. The Dragon departed from the ISS for Earth, full of scientific samples, equipment, and crew personal items, a little more than three hours after the context 2 photo was taken [33].
2. Contexts 3–10: Period 2 (S2 Fig in S3 File) was a “stable” eight-day period in the sample, when little activity is apparent, few objects were moved or transferred in or out the square, and the primary function of the area seems to be storage rather than work. In context 6, a large Post-It notepad appeared in the center of the metal panel with a phone number written on it. This number belonged to another astronaut, presumably indicating that someone on the ISS had been told to call that colleague on the ground (for reasons of privacy, and in accordance with NASA rules for disseminating imagery, we have blurred the number in the relevant images). In context 8, the same notepad sheet had new writing appear on it, this time reading “COL A1 L1,” the location of an experimental rack in the European lab module.
3. Contexts 11–12: Period 3 (S3 Fig in S3 File) involves a second appearance of a portable glovebag (a different one from that used in contexts 0–1, according to its serial number), this time for a known activity, a concrete hardening experiment belonging to the European Space Agency [34, 35]. This two-day phase indicates how the MWA space can be shared with non-US agencies when required. It also demonstrates the utility of this flexible area for work beyond biology/medicine, such as material science. Oversight of the crew’s activities by ground staff is evident from the positioning of the video camera and LED light pointing into the glovebag.
4. Contexts 13–27: Period 4 (S4 Fig in S3 File) is another stable fifteen-day period, similar to Period 2. Many items continued to be stored on the aluminum panel. The LED light’s presence is a trace of the activity in Period 3 that persists throughout this phase. Only in context 25 can a movement of the lamp potentially be connected to an activity relating to one of the stored items on the wall: at least one nitrile glove was removed from a resealable bag behind the lamp. In general, the primary identifiable activity during Period 4 is storage.
5. Contexts 28–32: Period 5 (S5 Fig in S3 File), by contrast, represents a short period of five days of relatively high and diverse activity. In context 28, a Microsoft Hololens augmented reality headset appeared. According to the DSR for the previous day, a training activity called Sidekick was carried out using the headset [36]. The following day, a Saturday, showed no change in the quantity or type of objects, but many were moved around and grouped by function—adhesive tape rolls were placed together, tools were moved from Velcro patches into pouches or straightened, and writing implements were placed in a vertical orientation when previously they were tilted. Context 29 represents a cleaning and re-organization of the sample area, which is a common activity for the crew on Saturdays [37]. Finally, in context 32, an optical coherence tomography scanner—a large piece of equipment for medical research involving crew members’ eyes—appeared [38]. This device was used previously during the sample period, but on the same day as the ESA concrete experiment, so that earlier work seems to have happened elsewhere [39].
6. Contexts 33–40: Period 6 (S6 Fig in S3 File) is the third stable period, in which almost no changes are visible over eight days. The only sign of activity is a digital timer which was started six hours before the context 39 image was made and continued to run at least through context 42.
7. Context 41: Period 7 (S7 Fig in S3 File) is a single context in which medical sample collection may have occurred. Resealable bags (some holding others) appeared in the center of the image and at lower right. One of the bags at lower right had a printed label reading “Reservoir Containers.” We were not able to discern which type of reservoir containers the label refers to, although the DSR for the day mentions “[Human Research Facility] Generic Saliva Collection,” without stating the location for this work [40]. Evidence from photos of other squares shows that labeled bags could be re-used for other purposes, so our interpretation of medical activity for this context is not conclusive.
8. Contexts 42–60: Period 8 (S8 Fig in S3 File) is the last and longest period of stability and low activity—eighteen days in which no specific activity other than the storage of items can be detected. The most notable change is the appearance for the first time of a foil water pouch in the central part of the blue panel.

In the standards used at the time of installation, “stowage space” was the sixth design requirement listed for the MWA after accessibility; equipment size capability; scratch-resistant surfaces; capabilities for electrical, mechanical, vacuum, and fluid support during maintenance; and the accommodation of diagnostic equipment [20]. Only capabilities for fabrication were listed lower than stowage. Yet 50 of the 60 contexts (83%) fell within stable periods where little or no activity is identifiable in Square 03. According to the sample results, therefore, this area seems to exist not for “maintenance,” but primarily for the storage and arrangement of items. The most recent update of the design standards does not mention the MWA, but states, “Stowage location of tool kits should be optimized for accessibility to workstations and/or maintenance workbenches” [25]. Our observation confirms the importance of this suggestion.

The MWA was also a flexible location for certain science work, like the concrete study or crew health monitoring. Actual maintenance of equipment was hardly in evidence in the sample (possibly contexts 25, 39, and 44), and may not even have happened at all in this location. Some training did happen here, such as review of procedures for the Electromagnetic Levitator camera (instructions for changing settings on a high-speed camera appeared on the laptop screen; the day’s DSR shows that this camera is part of the Electromagnetic Levitator facility, located in the Columbus module [41]. The training required the use of the Hololens system (context 28 DSR, cited above).

Although many item types were represented in Square 03, it became clear during data capture how many things were basically static, unmoving and therefore unused, especially certain tools, writing implements, and body maintenance items. The MWA was seen as an appropriate place to store these items. It may be the case that their presence here also indicates that their function was seen as an appropriate one for this space, but the function(s) may not be carried out—or perhaps not in this location. Actualization of object function was only visible to us when the state of the item changed—it appeared, it moved, it changed orientation, it disappeared, or, in the case of artifacts that were grouped in collections rather than found as singletons, its shape changed or it became visibly smaller/lesser. We therefore have the opportunity to explore not only actuality of object use, but also *potentiality* of use or function, and the meaning of that quality for archaeological interpretation [42, 43]. This possibility is particularly intriguing in light of the archaeological turn towards recognizing the agency of objects to impact human activity [44, 45]. We will explore these implications in a future publication.

### Square 05

We performed the same chronological analysis for Square 05. Fig 6(B) represents the analysis for both item types and for item functions. We identified three major phases of activity, corresponding to contexts 0–5, 6–52, and 53–59 (S9-S11 Figs in S3 File). The primary characteristics of these phases relate to an early period of unclear associations (0–5) marked by the presence of rolls of adhesive tape and a few body maintenance items (toothpaste and toothbrush, wet wipes); the appearance of a toiletry kit on the right side of the sample area, fully open with clear views of many of the items contained within (6–52); and finally, the closure of the toiletry kit so that its contents can no longer be seen (53–59). We interpret the phases as follows:

1. Contexts 0–5: In Period 1 (six days, S9 Fig in S3 File), while items such as a mirror, dental floss picks, wet wipes, and a toothbrush held in the end of a toothpaste tube were visible, the presence of various other kinds of items confounds easy interpretation. Two rolls of duct tape were stored on the handrail in the center of the sample area, and the Touch Array and laptop appeared in the center. Little movement can be identified, apart from a blue nitrile glove that appeared in context 1 and moved left across the area until it was wedged into the bungee cord for contexts 3 and 4. The tape rolls were removed prior to context 5. A collection of resealable bags was wedged behind the handrail in context 3, remaining there until context 9. Overall, this appears to be a period characterized by eclectic associations, showing an area without a clear designated function.
2. Contexts 6–52: Period 2 (S10 Fig in S3 File) is clearly the most significant one for this location due to its duration (47 days). It was dominated by the number of body maintenance items located in and around the toiletry kit, especially a white hand towel (on which a brown stain was visible from context 11, allowing us to confirm that the same towel was present until context 46). A second towel appeared alongside the toiletry kit in context 47, and the first one was fixed at the same time to the handrail, where it remained through the end of the sample period. A third towel appeared in context 52, attached to the handrail together with the first one by a bungee cord, continuing to the end of the sample period. Individual body maintenance items moved frequently from one context to the next, showing the importance of this type of activity for this part of Node 3. For reasons that are unclear, the mirror shifted orientation from vertical to diagonal in context 22, and then was put back in a vertical orientation in context 31 (a Monday, a day which is not traditionally associated with cleaning and organization).
  Collections of resealable bags appeared at various times, including a large one labeled “KYNAR BAG OF ZIPLOCKS” in green marker at the upper left part of the sample area beginning of context 12 (Kynar is a non-flammable plastic material that NASA prefers for resealable bags to the generic commercial off-the-shelf variety because it is non-flammable; however, its resistance to heat makes it less desirable for creating custom sizes, so bags made from traditional but flammable low-density polyethylene still dominate on the ISS [14]). The Kynar bag contained varying numbers of bags within it over time; occasionally, it appeared to be empty. The Touch Array changed orientation on seven of 47 days in period 2, or 15% of the time (12% of all days in the survey), showing activity associated with scientific research in this area. In context 49, a life-support item, the Airborne Particulate Monitor (APM) was installed [46]. This device, which measures “real-time particulate data” to assess hazards to crew health [47], persisted through the end of the sample period.
3. Contexts 53–59: Period 3 (S11 Fig in S3 File) appears as a seven-day phase marked by low activity. Visually, the most notable feature is the closure of the toiletry kit, which led to much lower counts of body maintenance items. Hardly any of the items on the wall moved at all during this period.

While body maintenance in the form of cleaning and caring for oneself could be an expected function for an area with exercise and excretion facilities, it is worth noting that the ISS provides, at most, minimal accommodation for this activity. A description of the WHC stated, “To provide privacy…an enclosure was added to the front of the rack. This enclosure, referred to as the Cabin, is approximately the size of a typical bathroom stall and provides room for system consumables and hygiene item stowage. Space is available to also support limited hygiene functions such as hand and body washing” [48]. A diagram of the WHC in the same publication shows the Cabin without a scale but suggests that it measures roughly 2 m (h) x .75 (w) x .75 (d), a volume of approximately 1.125 m^3^. NASA’s current design standards state that the body volume of a 95th percentile male astronaut is 0.99 m^3^ [20], meaning that a person of that size would take up 88% of the space of the Cabin, leaving little room for performing cleaning functions—especially if the Cabin is used as apparently intended, to also hold “system consumables and hygiene item[s]” that would further diminish the usable volume. This situation explains why crews try to adapt other spaces, such as storage areas like the PMM, for these activities instead. According to the crew debriefing statement, only one of them used the WHC for body maintenance purposes; it is not clear whether the toiletry kit belonged to that individual. But the appearance of the toiletry kit in Square 05—outside of the WHC, in a public space where others frequently pass by—may have been a response to the limitations of the WHC Cabin. It suggests a need for designers to re-evaluate affordances for body maintenance practices and storage for related items.

### Comparison

Although Square 03 and 05 were different sizes and shapes, comparing the density of items by function shows evidence of their usage (Table 3). The typical context in Square 03 had twice as many restraints and containers, but less than one-quarter as many body maintenance items as Square 05. 03 also had many tools, lights, audiovisual equipment, and writing implements, while there were none of any of these types in 05. 05 had life support and hygiene items which were missing from 03. It appears that flexibility and multifunctionality were key elements for 03, while in 05 there was emphasis on one primary function (albeit an improvised one, designated by the crew rather than architects or ground control), cleaning and caring for one’s body, with a secondary function of housing static equipment for crew hygiene and life support.

**Table 3:** Average item density (count per m^2^).

Function category	Square 03	Square 05
Audiovisual	4.9	0
Body maintenance	3.1	13.3
Computing	2.4	3.6
Container	6.6	3.2
Drink	0.3	0.9
Experiment	4.8	1.7
Hygiene	0	2.8
Life support	0	0.5
Light	3.2	0
Power	3.1	3.6
Restraint	26.5	13.8
Safety	3.2	0.1
Tool	8.8	0
Writing	6.6	0

As this is the first time such an analysis has been performed, it is not yet possible to say how typical or unusual these squares are regarding the types of activities taking place; but they provide a baseline for eventual comparison with the other four squares and future work on ISS or other space habitats.

## Conclusion

Some general characteristics are revealed by archaeological analysis of a space station’s material culture. First, even in a small, enclosed site, occupied by only a few people over a relatively short sample period, we can observe divergent patterns for different locations and activity phases. Second, while distinct functions are apparent for these two squares, they are not the functions that we expected prior to this research. As a result, our work fulfills the promise of the archaeological approach to understanding life in a space station by revealing new, previously unrecognized phenomena relating to life and work on the ISS. There is now systematically recorded archaeological data for a space habitat.

Squares 03 and 05 served quite different purposes. The reasons for this fact are their respective affordances and their locations relative to activity areas designated for science and exercise. Their national associations, especially the manifestation of the control wielded by NASA over its modules, also played a role in the use of certain materials, the placement of facilities, and the organization of work. How each area was used was also the result of an interplay between the original plans developed by mission planners and habitat designers (or the lack of such plans), the utility of the equipment and architecture in each location, and the contingent needs of the crew as they lived in the station. This interplay became visible in the station’s material culture, as certain areas were associated with particular behaviors, over time and through tradition—over the long duration across many crews (Node 2, location of Square 03, docked with the ISS in 2007, and Node 3, location of Square 05, docked in 2010), and during the specific period of this survey, from January to March 2022. During the crew debriefing, one astronaut said, “We were a pretty organized crew who was also pretty much on the same page about how to do things…. As time went on…we organized the lab and kind of got on the same page about where we put things and how we’re going to do things.” This statement shows how functional associations can become linked to different areas of the ISS through usage and mutual agreement. At the same time, the station is not frozen in time. Different people have divergent ideas about how and where to do things. It seems from the appearance of just one Russian item—a packet of generic wipes (*salfetky sukhiye*) stored in the toiletry kit throughout the sample period—that the people who used these spaces and carried out their functions did not typically include the ISS’s Russian crew. Enabling greater flexibility to define how spaces can be used could have a significant impact on improving crew autonomy over their lives, such as how and where to work. It could also lead to opening of all spaces within a habitat to the entire crew, which seems likely to improve general well-being.

An apparent disjunction between planned and actual usage appeared in Square 03. It is intended for maintenance as well as other kinds of work. But much of the time, there was nobody working here—a fact that is not captured by historic photos of the area, precisely because nothing is happening. The space has instead become the equivalent of a pegboard mounted on a wall in a home garage or shed, convenient for storage for all kinds of items—not necessarily items being used there—because it has an enormous number of attachment points. Storage has become its primary function. Designers of future workstations in space should consider that they might need to optimize for functions other than work, because most of the time, there might not be any work happening there. They could optimize for quick storage, considering whether to impose a system of organization, or allow users to organize as they want.

We expected from previous (though unsystematic) observation of historic photos and other research, that resealable plastic bags (combined with Velcro patches on the bags and walls) would be the primary means for creating gravity surrogates to control items in microgravity. They only comprise 7% of all items in Square 03 (256 instances). There are more than twice as many clips (572—more than 9 per context) in the sample. There were 193 instances of adhesive tape rolls, and more than 100 cable ties, but these were latent (not holding anything), representing potentiality of restraint rather than actualization. The squares showed different approaches to managing “gravity.” While Square 03 had a pre-existing structured array of Velcro patches, Square 05 showed a more expedient strategy with Velcro added in response to particular activities. Different needs require different affordances; creating “gravity” is a more nuanced endeavor than it initially appears. More work remains to be done to optimize gravity surrogates for future space habitats, because this is evidently one of the most critical adaptations that crews have to make in microgravity (44% of all items in Square 03, 39% in 05).

Square 05 is an empty space, seemingly just one side of a passageway for people going to use the lifting machine or the latrine, to look out of the Cupola, or get something out of deep storage in one of the ISS’s closets. In our survey, this square was a storage place for toiletries, resealable bags, and a computer that never (or almost never) gets used. It was associated with computing and hygiene simply by virtue of its location, rather than due to any particular facilities it possessed. It has no affordances for storage. There are no cabinets or drawers, as would be appropriate for organizing and holding crew personal items. A crew member decided that this was an appropriate place to leave their toiletry kit for almost two months. Whether this choice was appreciated or resented by fellow crew members cannot be discerned based on our evidence, but it seems to have been tolerated, given its long duration. The location of the other four USOS crew members’ toiletry kits during the sample period is unknown. A question raised by our observations is: how might a function be more clearly defined by designers for this area, perhaps by providing lockers for individual crew members to store their toiletries and towels? This would have a benefit not only for reducing clutter, but also for reducing exposure of toiletry kits and the items stored in them to flying sweat from the exercise equipment or other waste particles from the latrine. A larger compartment providing privacy for body maintenance and a greater range of motion would also be desirable.

As the first systematic collection of archaeological data from a space site outside Earth, this analysis of two areas on the ISS as part of the SQuARE payload has shown that novel insights into material culture use can be obtained, such as the use of wall areas as storage or staging posts between activities, the accretion of objects associated with different functions, and the complexity of using material replacements for gravity. These results enable better space station design and raise new questions that will be addressed through analysis of the remaining four squares.

## Supporting information

S1 Movie NASA astronaut Kayla Barron installs the first square for the Sampling Quadrangle Assemblages Research Experiment in the Japanese Experiment Module (also known as Kibo) on the International Space Station, January 14, 2022. She places Kapton tape to mark the square’s upper right corner. Credit: NASA.
(MP4)

S1 Dataset (ZIP)

S2 Dataset (ZIP)

S3 Dataset The image annotations are represented according to sample square in json formatted text files. The data is available in the ‘SQuARE-notebooks’ repository on Github.com in the ‘data’ subfolder at https://github.com/issarchaeologicalproject/SQuARE-notebooks/tree/main; archived version of the repository is at Zenodo, DOI: 10.5281/zenodo.10654812.
(ZIP)

S1 File The ‘Rocket-Anno’ image annotation software is available on Github at https://github.com/issarchaeologicalproject/MRE-RocketAnno. The archived version of the repository is at Zenodo, DOI: 10.5281/zenodo.10648399.
(ZIP)

S2 File The computational notebooks that process the data json files to reshape the data suitable for basic statistics as well as the computation of the Brainerd-Robinson coefficients of similarity are in the.ipynb notebook format. The code is available in the ‘SQuARE-notebooks’ repository on Github.com in the ‘notebooks’ subfolder at https://github.com/issarchaeologicalproject/SQuARE-notebooks/tree/main; archived version of the repository is at Zenodo, DOI: 10.5281/zenodo.10654812. The software can be run online in the Google Colab environment (https://colab.research.google.com) or any system running Jupyter Notebooks (https://jupyter.org/).
(ZIP)

S3 File (ZIP)
