# Supplementary figures and images for: Archaeology in space: The Sampling Quadrangle Assemblages Research Experiment (SQuARE) on the International Space Station. Report 1: Squares 03 and 05 (part 1 of 2)
Source: PLoS One. 2024 Aug 7;19(8):e0304229. doi: 10.1371/journal.pone.0304229 (PMC11305871; doi:10.1371/journal.pone.0304229)

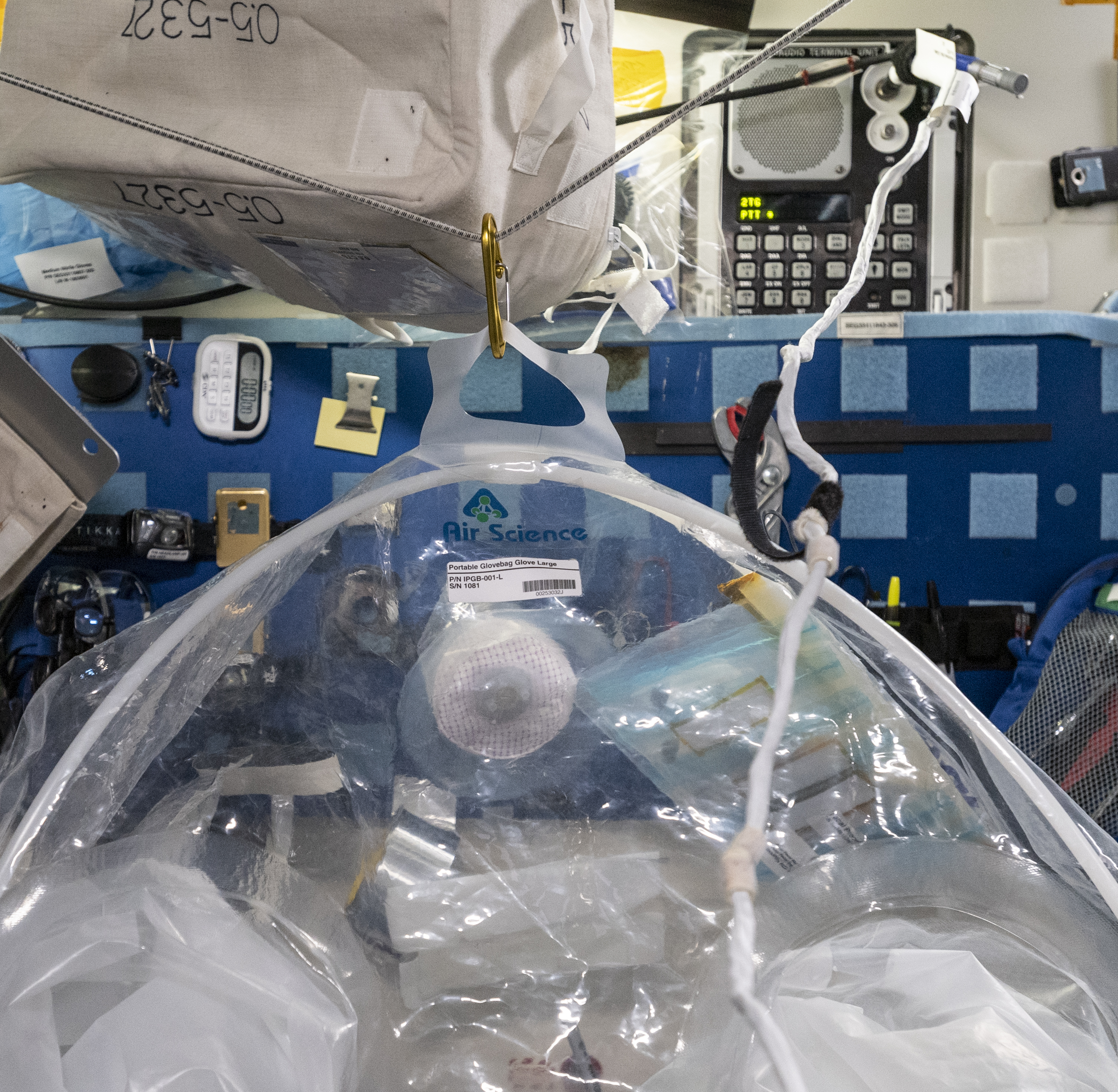

Supplement: S1 Dataset — (ZIP) [file pone.0304229.s002.zip › S03 - 00 - iss066e123429crop.jpg]

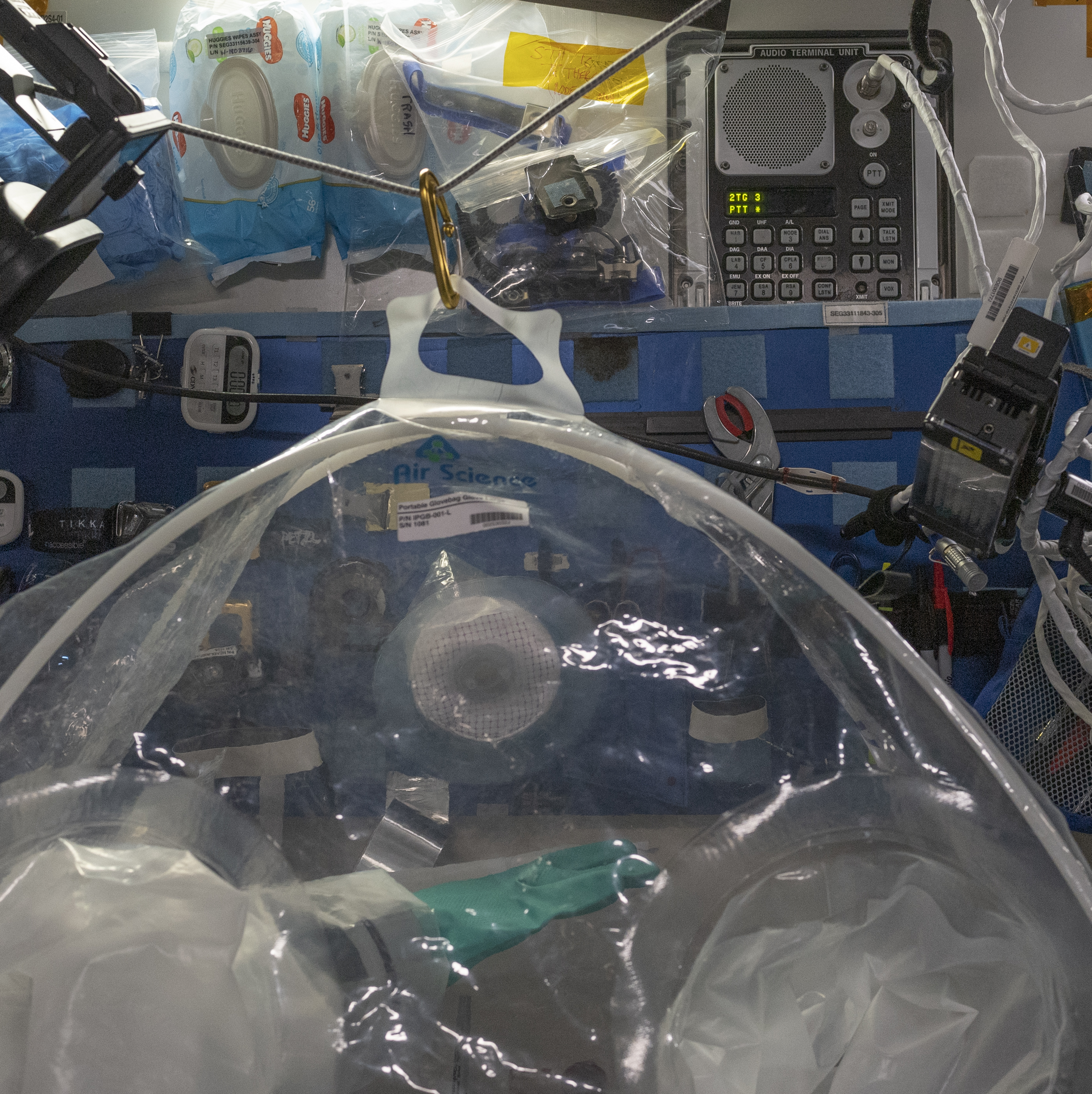

Supplement: S1 Dataset — (ZIP) [file pone.0304229.s002.zip › S03 - 01 - iss066e124425crop.jpg]

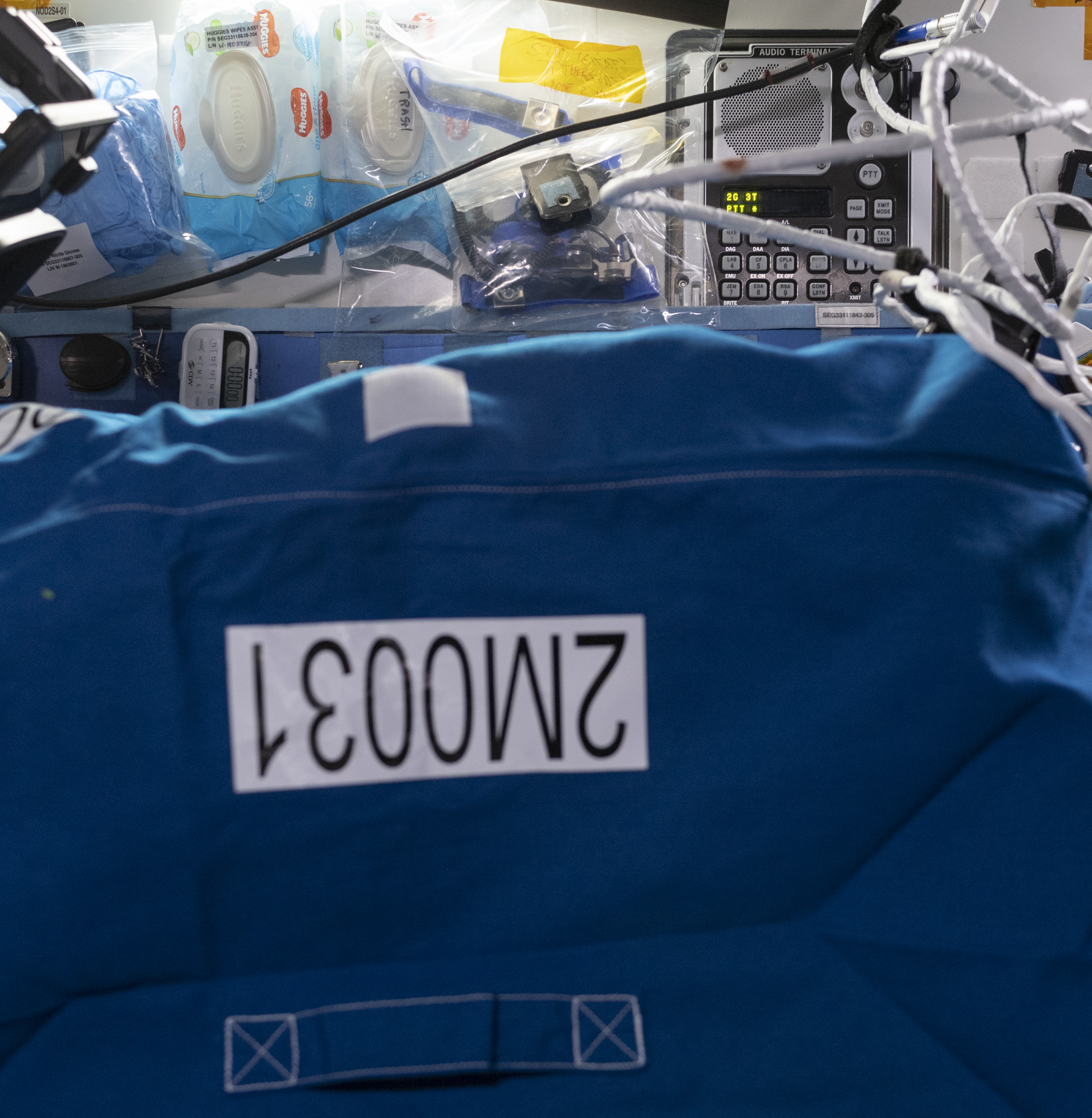

Supplement: S1 Dataset — (ZIP) [file pone.0304229.s002.zip › S03 - 02 - iss066e124435.jpg]

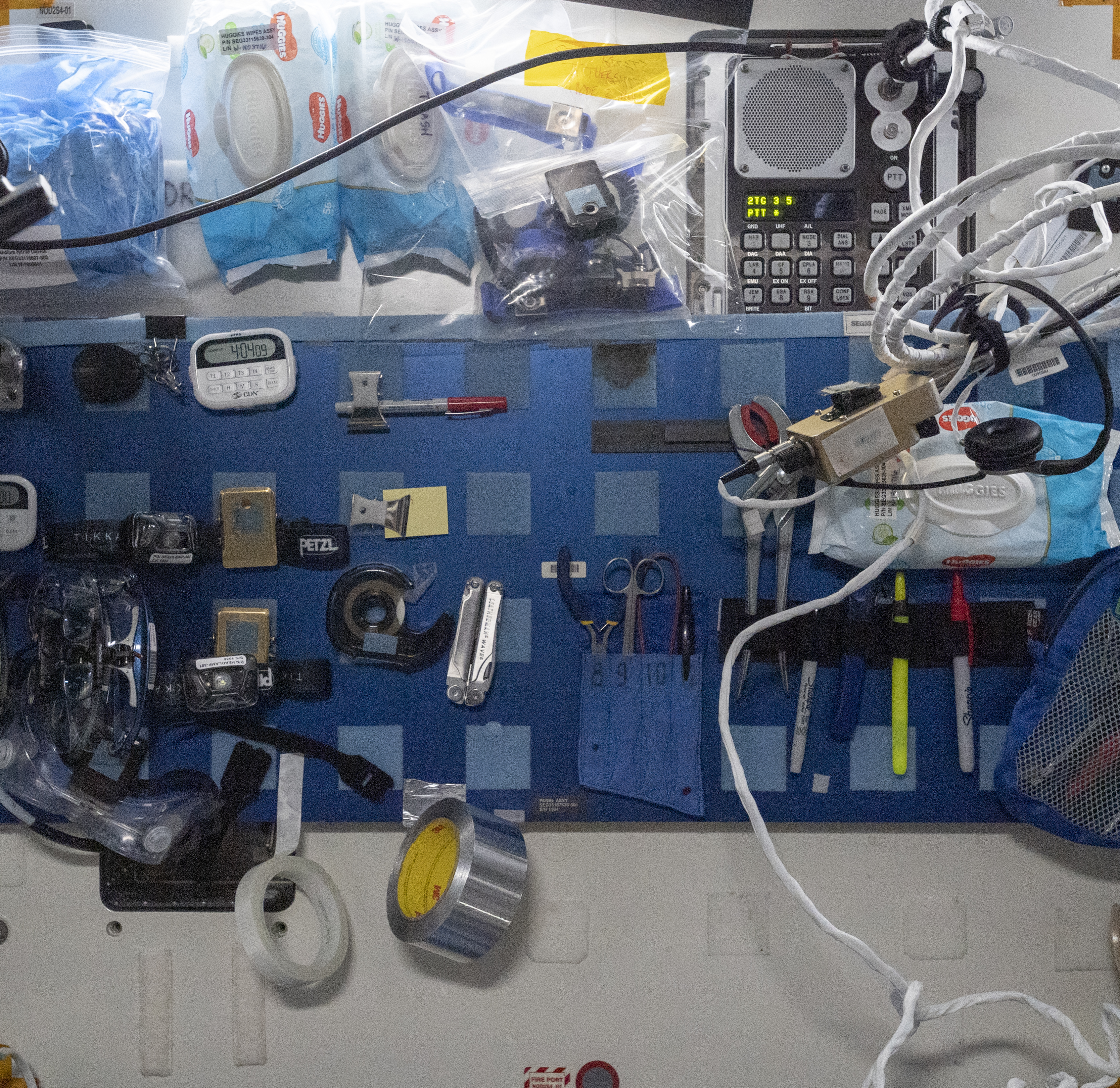

Supplement: S1 Dataset — (ZIP) [file pone.0304229.s002.zip › S03 - 03 - iss066e124597.jpg]

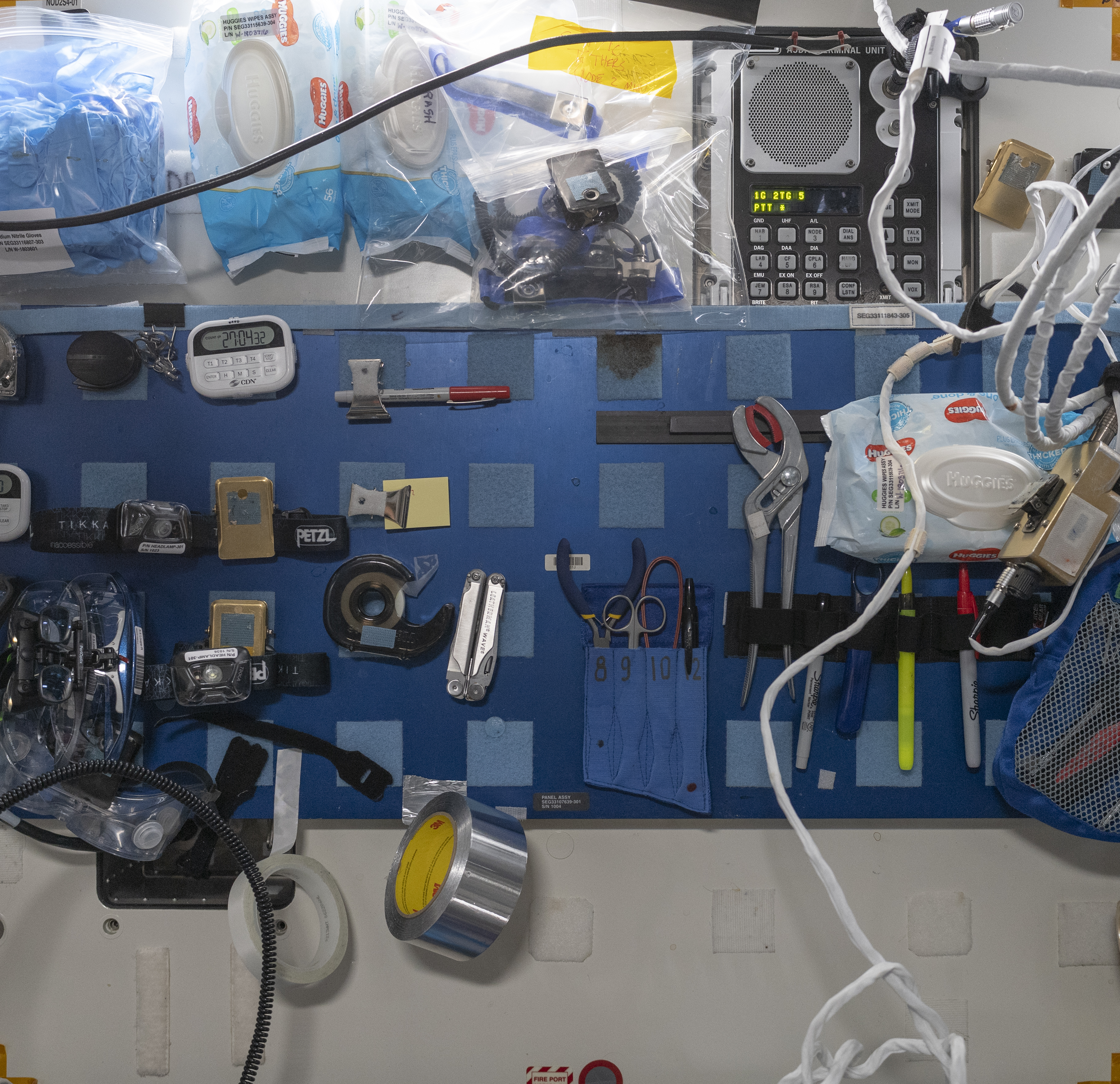

Supplement: S1 Dataset — (ZIP) [file pone.0304229.s002.zip › S03 - 04 - iss066e125373.jpg]

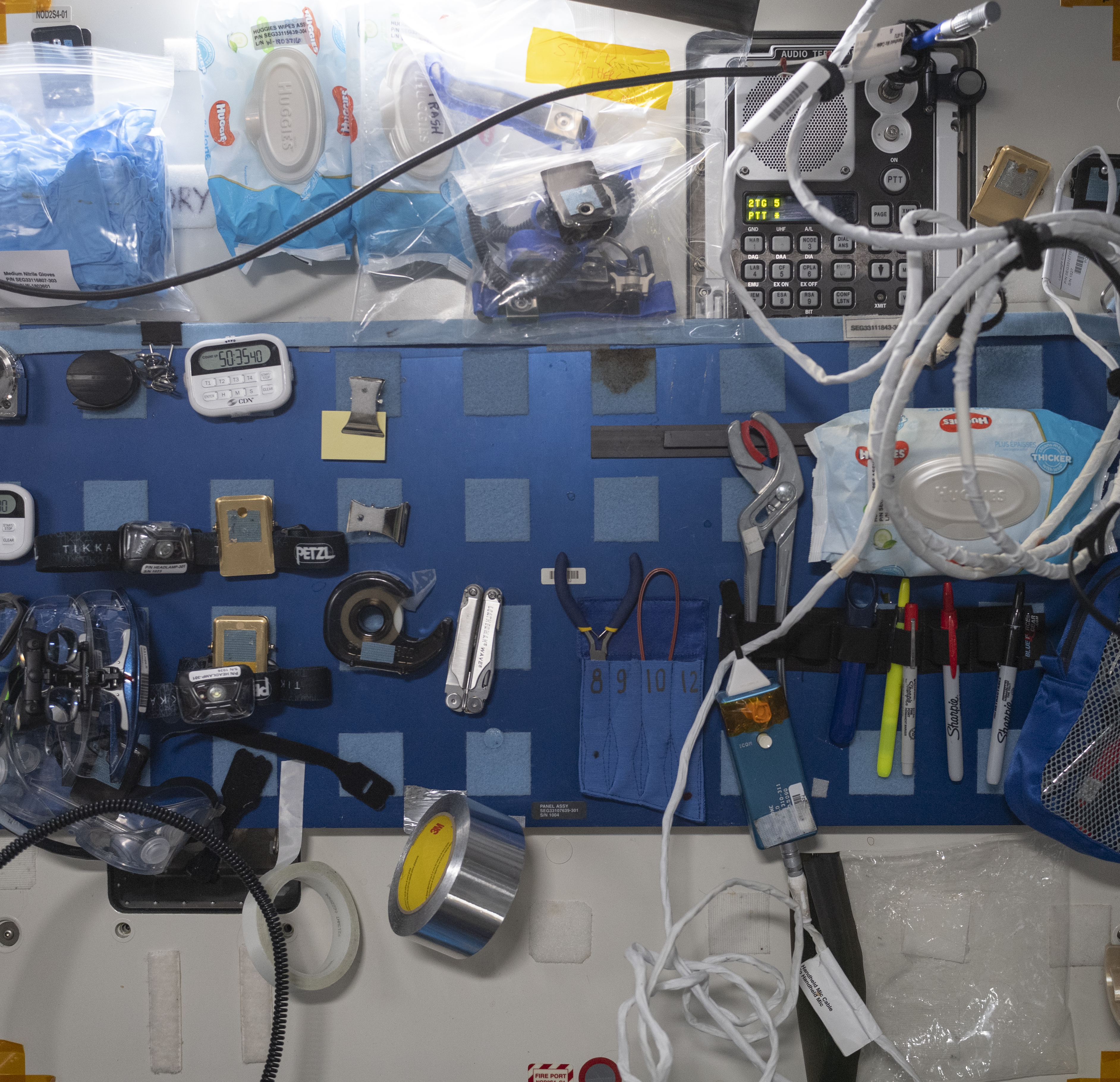

Supplement: S1 Dataset — (ZIP) [file pone.0304229.s002.zip › S03 - 05 - iss066e126151.jpg]

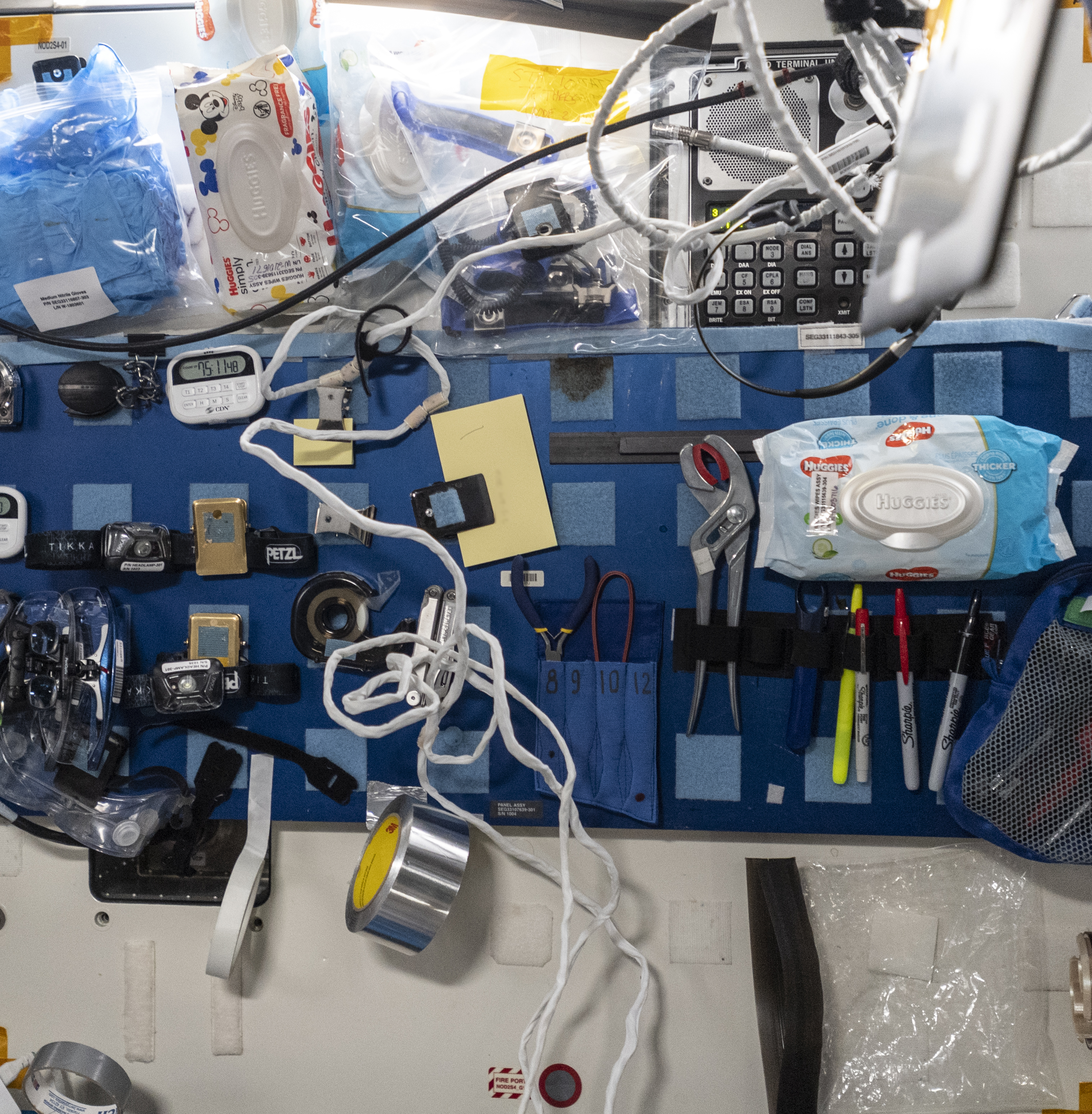

Supplement: S1 Dataset — (ZIP) [file pone.0304229.s002.zip › S03 - 06 - iss066e129516.jpg]

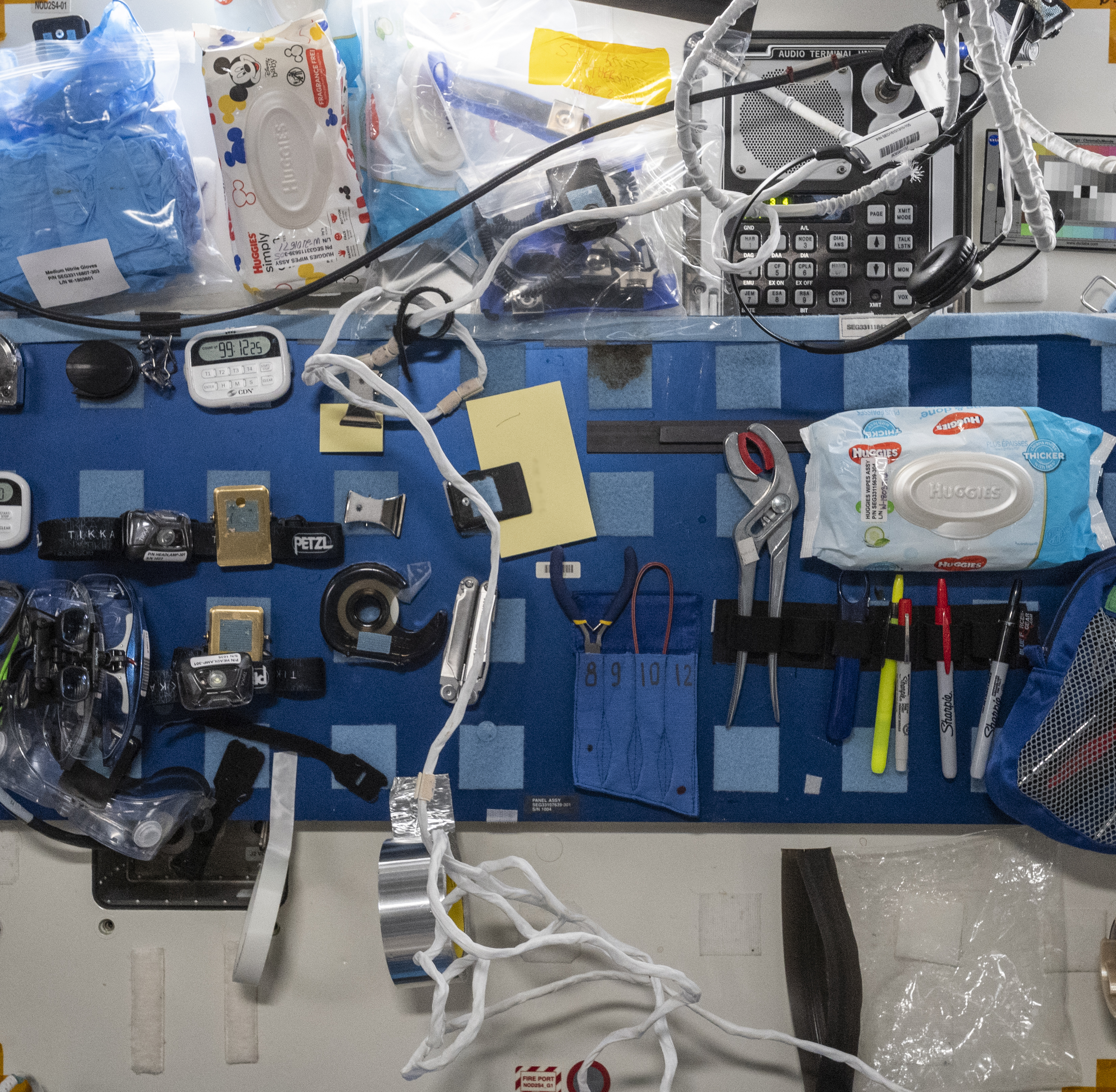

Supplement: S1 Dataset — (ZIP) [file pone.0304229.s002.zip › S03 - 07 - iss066e129752.jpg]

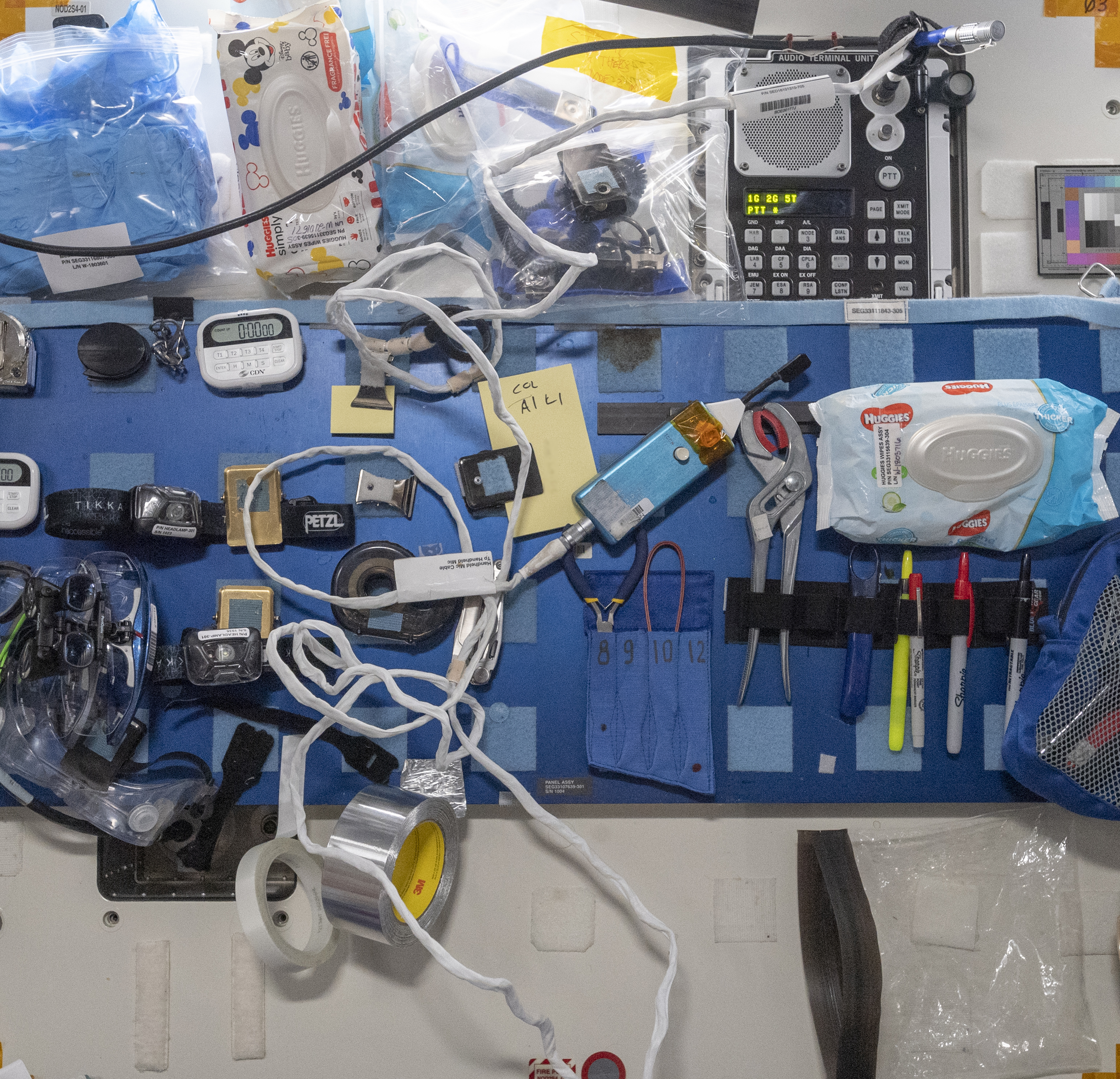

Supplement: S1 Dataset — (ZIP) [file pone.0304229.s002.zip › S03 - 08 - iss066e130178.jpg]

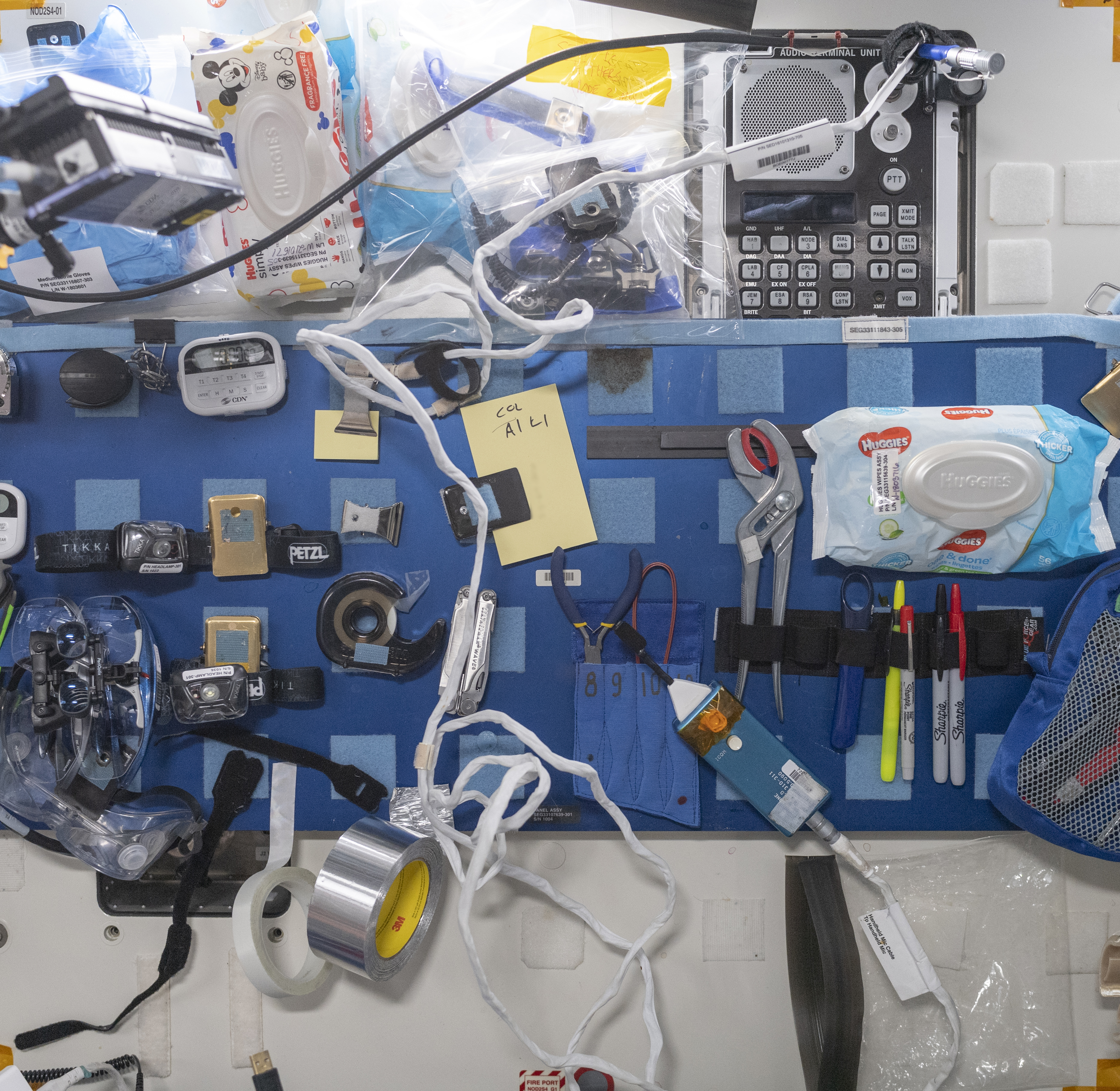

Supplement: S1 Dataset — (ZIP) [file pone.0304229.s002.zip › S03 - 09 - iss066e130194.jpg]

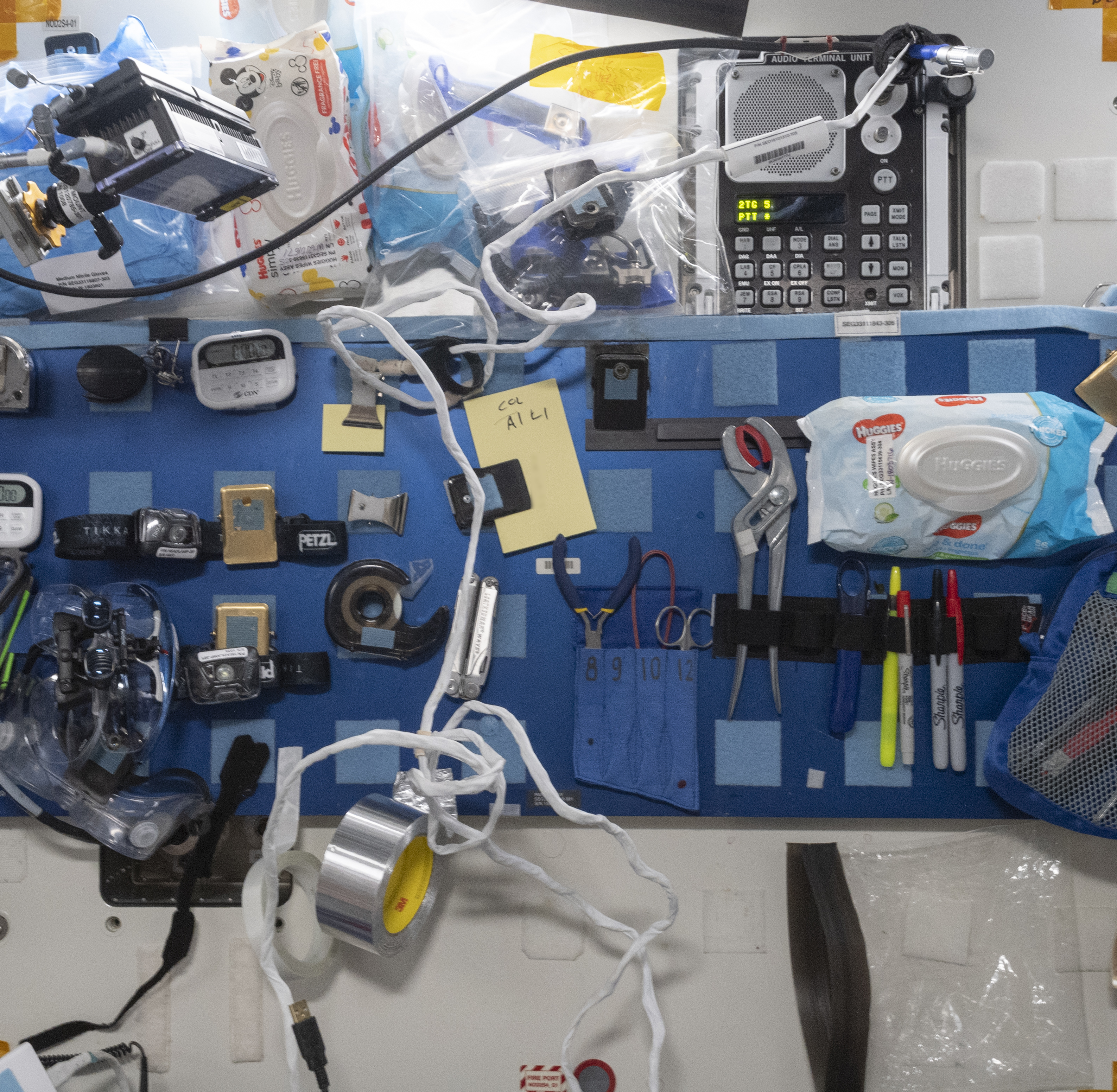

Supplement: S1 Dataset — (ZIP) [file pone.0304229.s002.zip › S03 - 10 - iss066e131827.jpg]

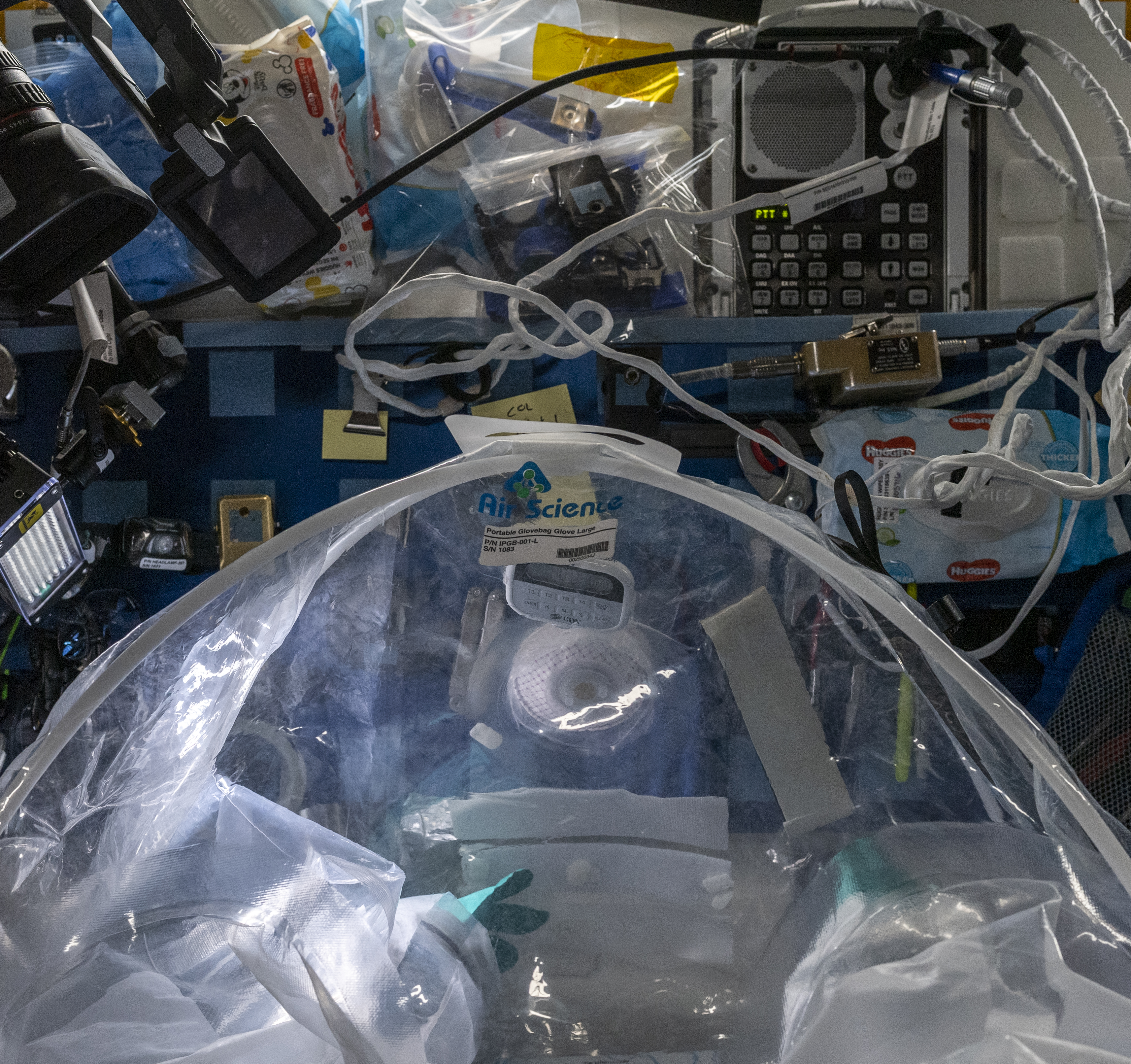

Supplement: S1 Dataset — (ZIP) [file pone.0304229.s002.zip › S03 - 11 - iss066e132465.jpg]

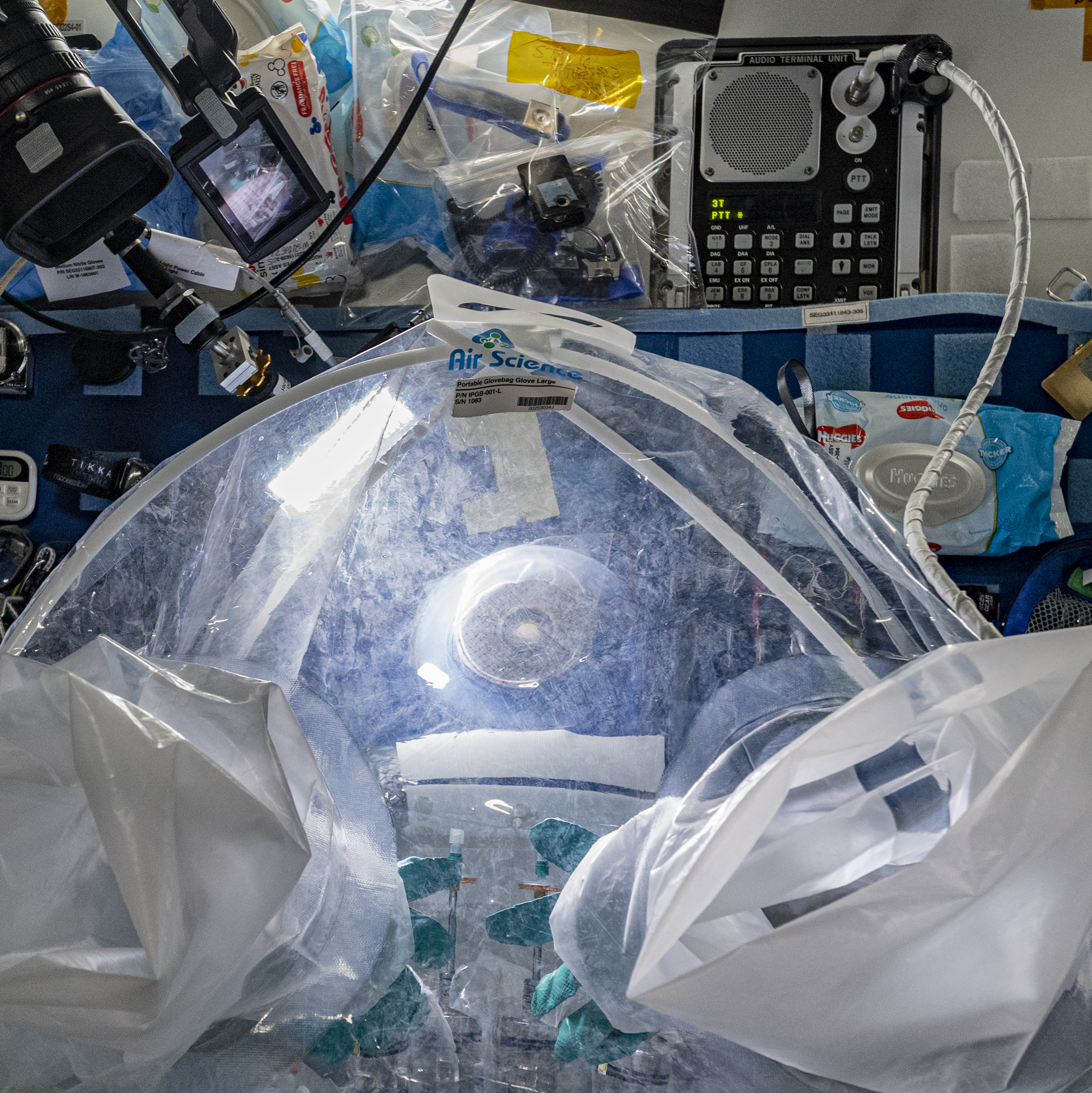

Supplement: S1 Dataset — (ZIP) [file pone.0304229.s002.zip › S03 - 12 - iss066e133908a.jpg]

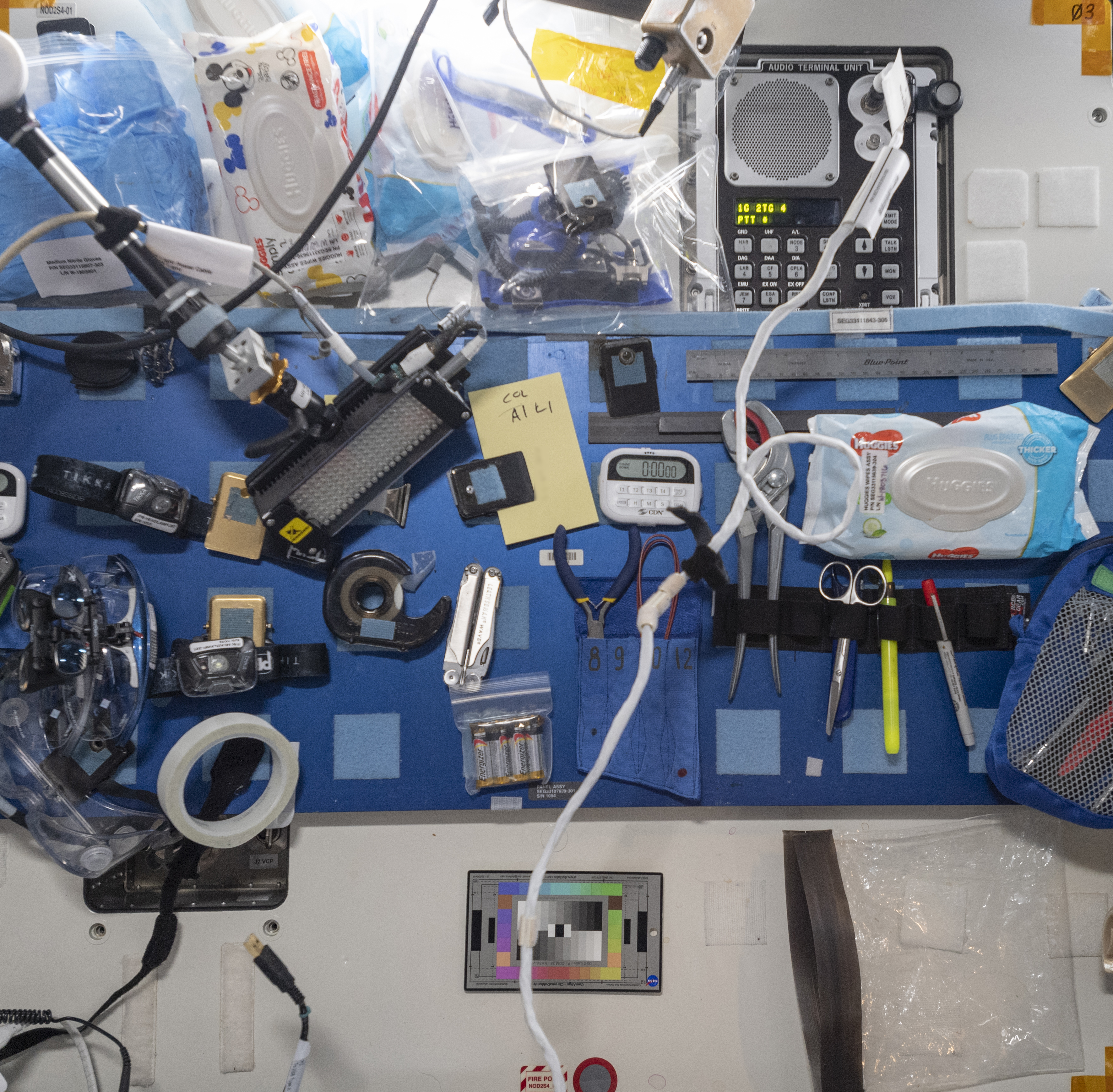

Supplement: S1 Dataset — (ZIP) [file pone.0304229.s002.zip › S03 - 13 - iss066e134829.jpg]

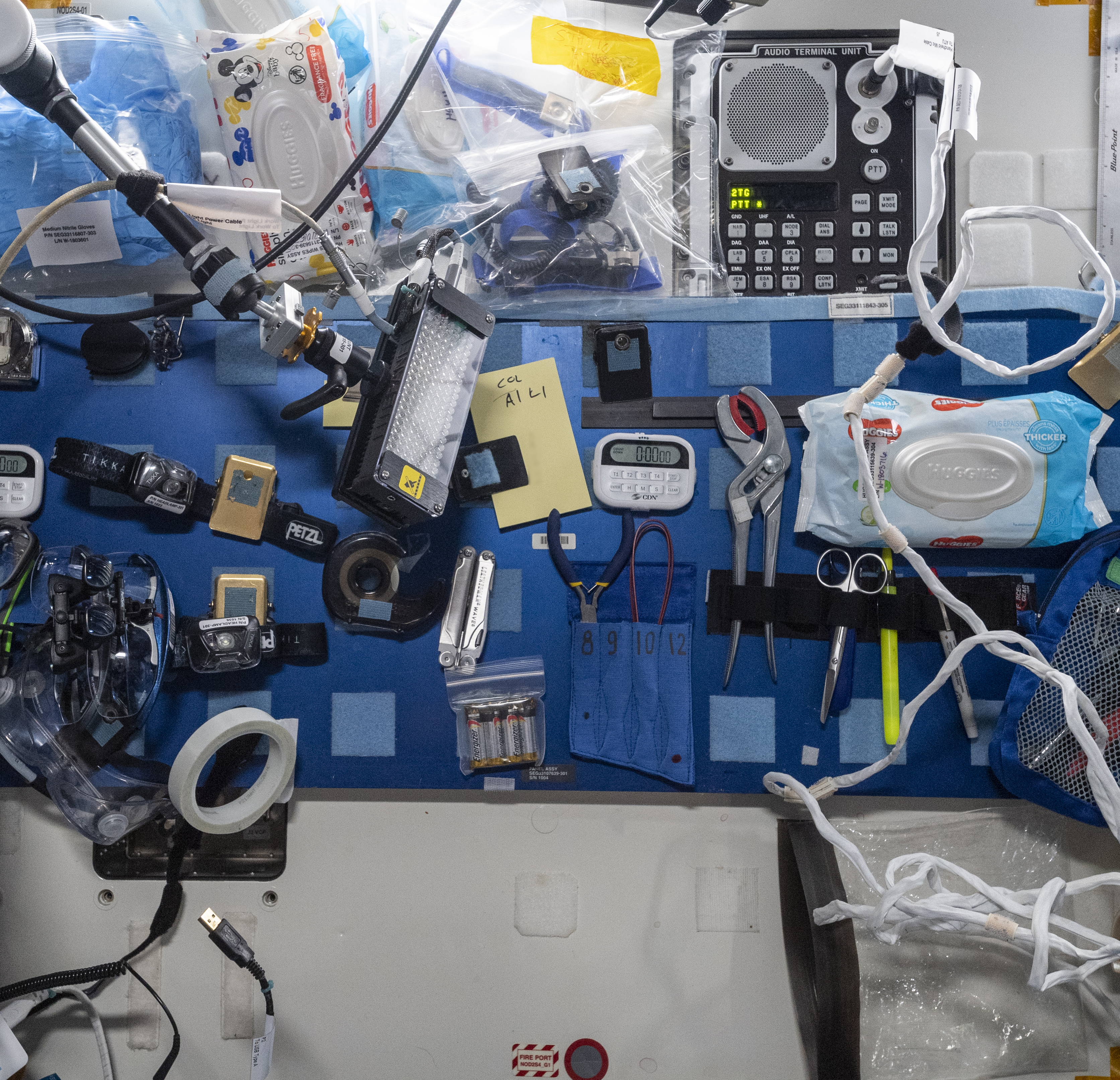

Supplement: S1 Dataset — (ZIP) [file pone.0304229.s002.zip › S03 - 14 - iss066e135846.jpg]

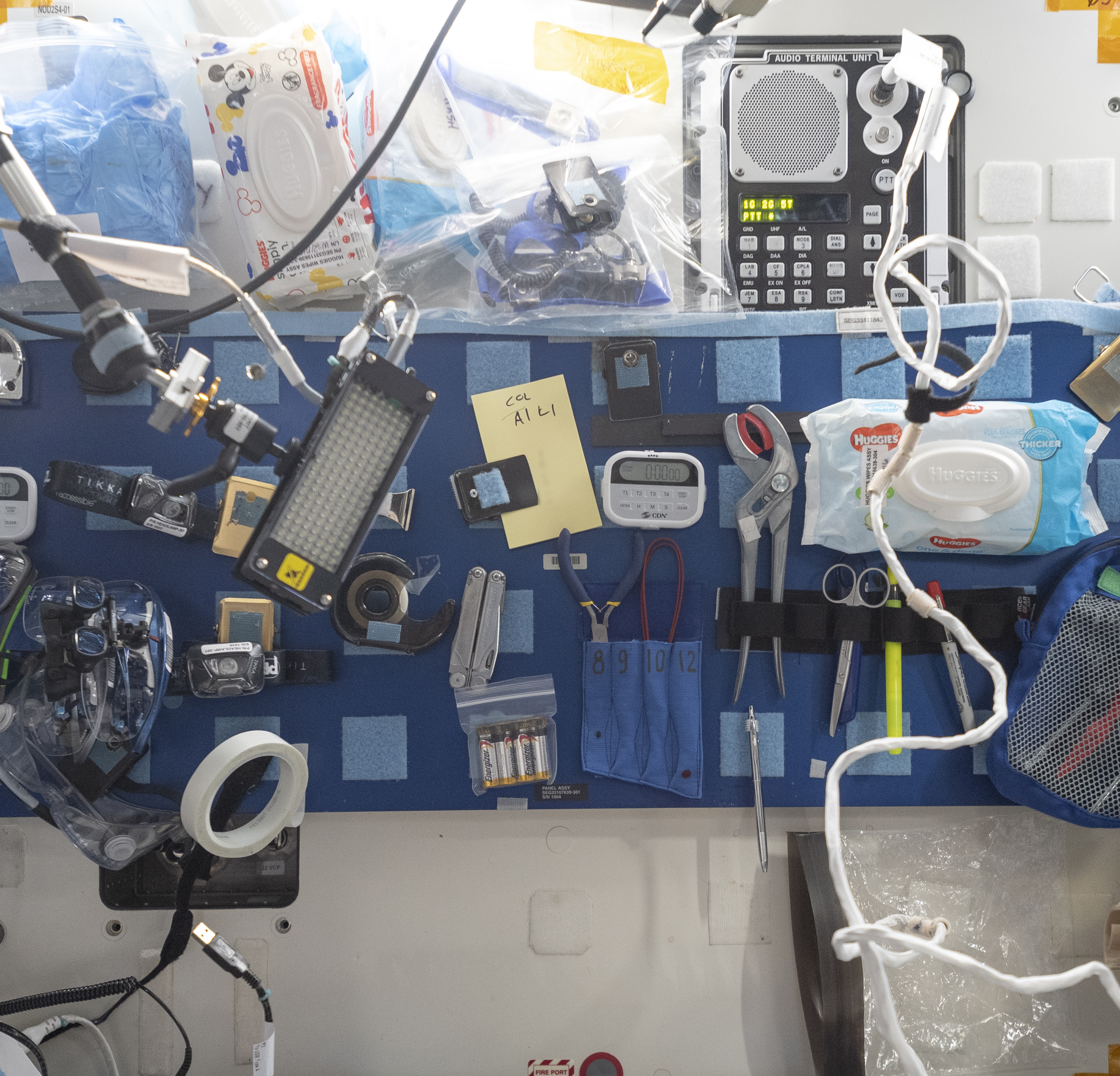

Supplement: S1 Dataset — (ZIP) [file pone.0304229.s002.zip › S03 - 15 - iss066e136130.jpg]

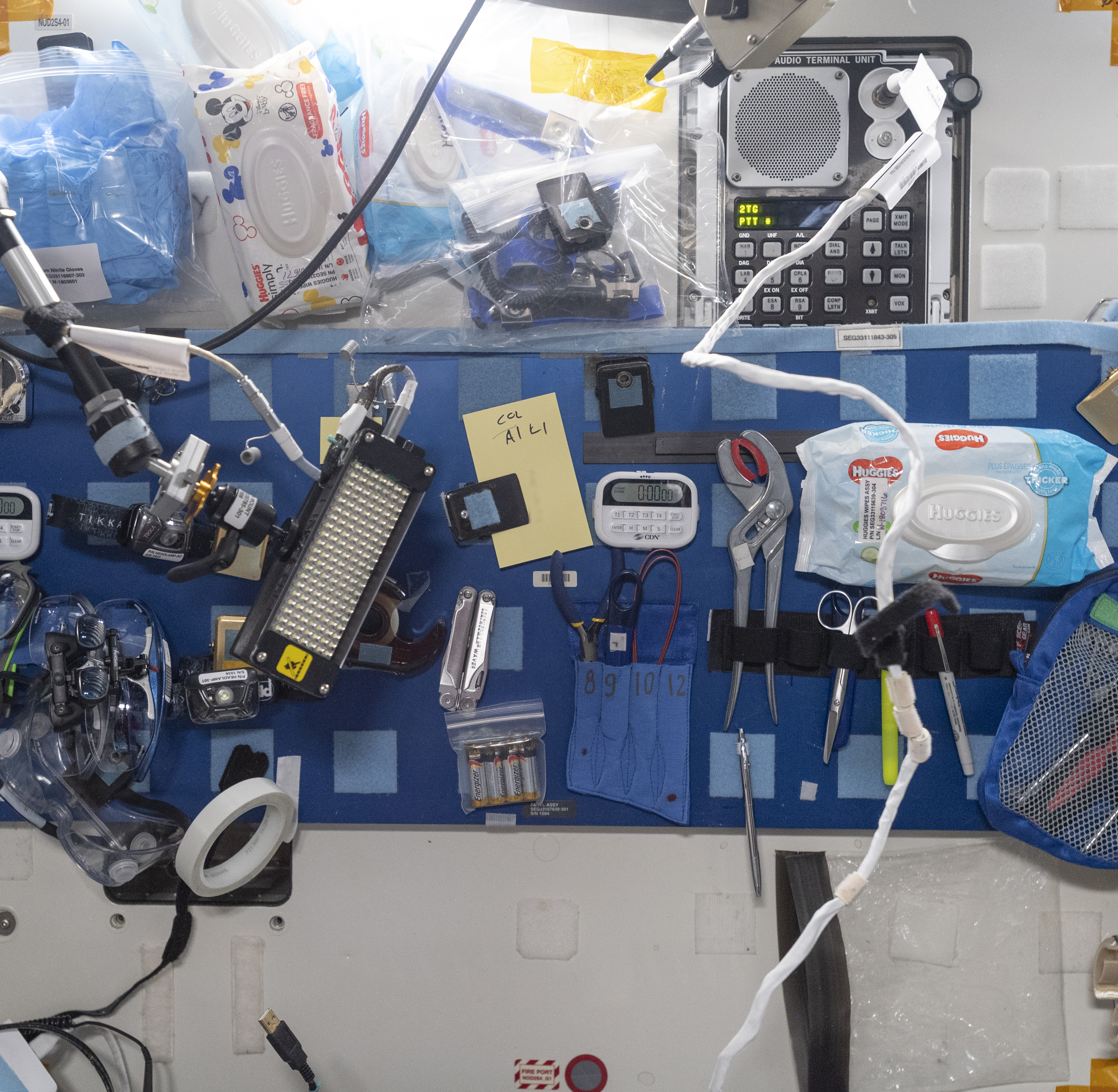

Supplement: S1 Dataset — (ZIP) [file pone.0304229.s002.zip › S03 - 16 - iss066e136635.jpg]

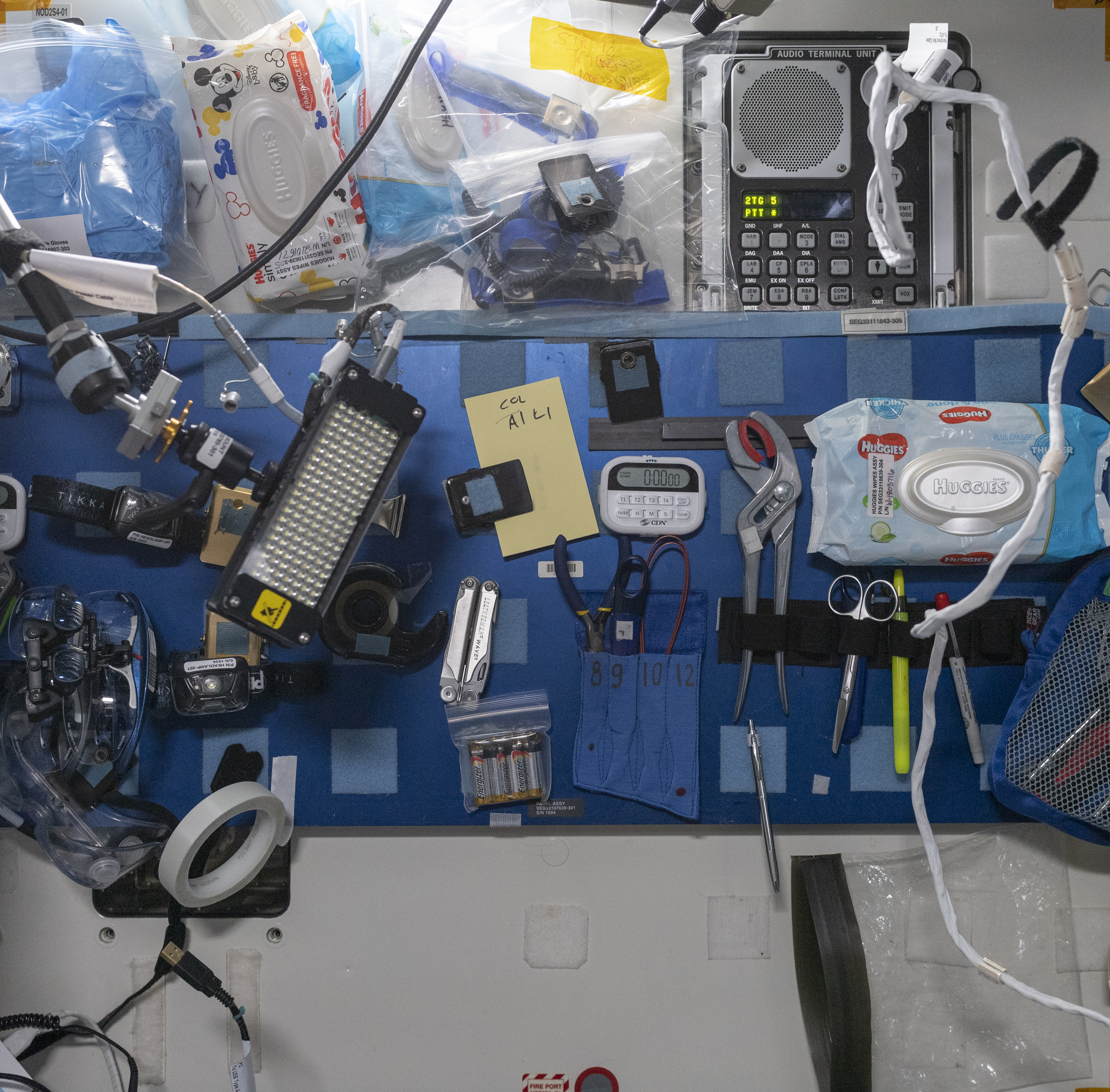

Supplement: S1 Dataset — (ZIP) [file pone.0304229.s002.zip › S03 - 17 - iss066e136641.jpg]

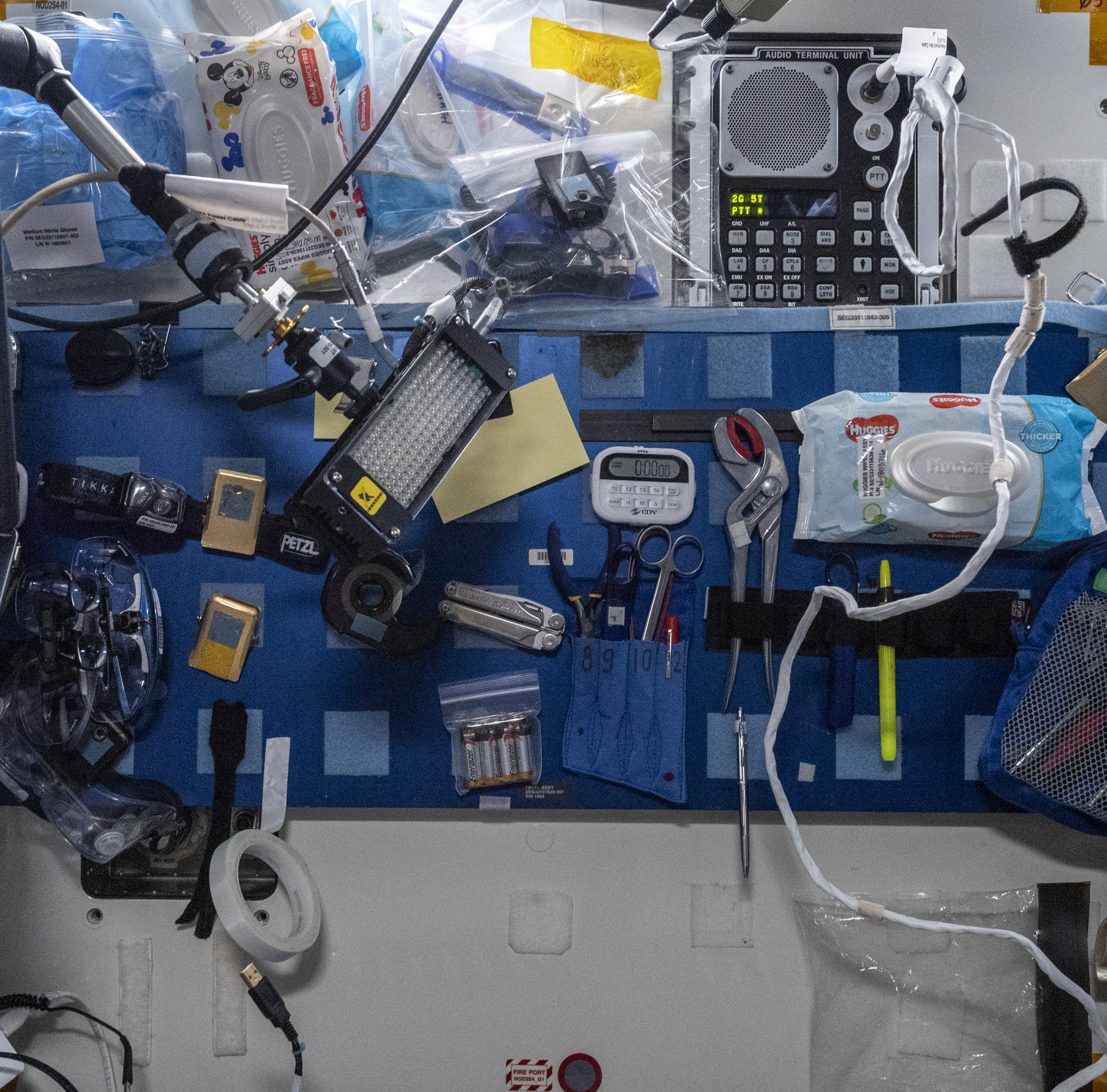

Supplement: S1 Dataset — (ZIP) [file pone.0304229.s002.zip › S03 - 18 - iss066e137044.jpg]

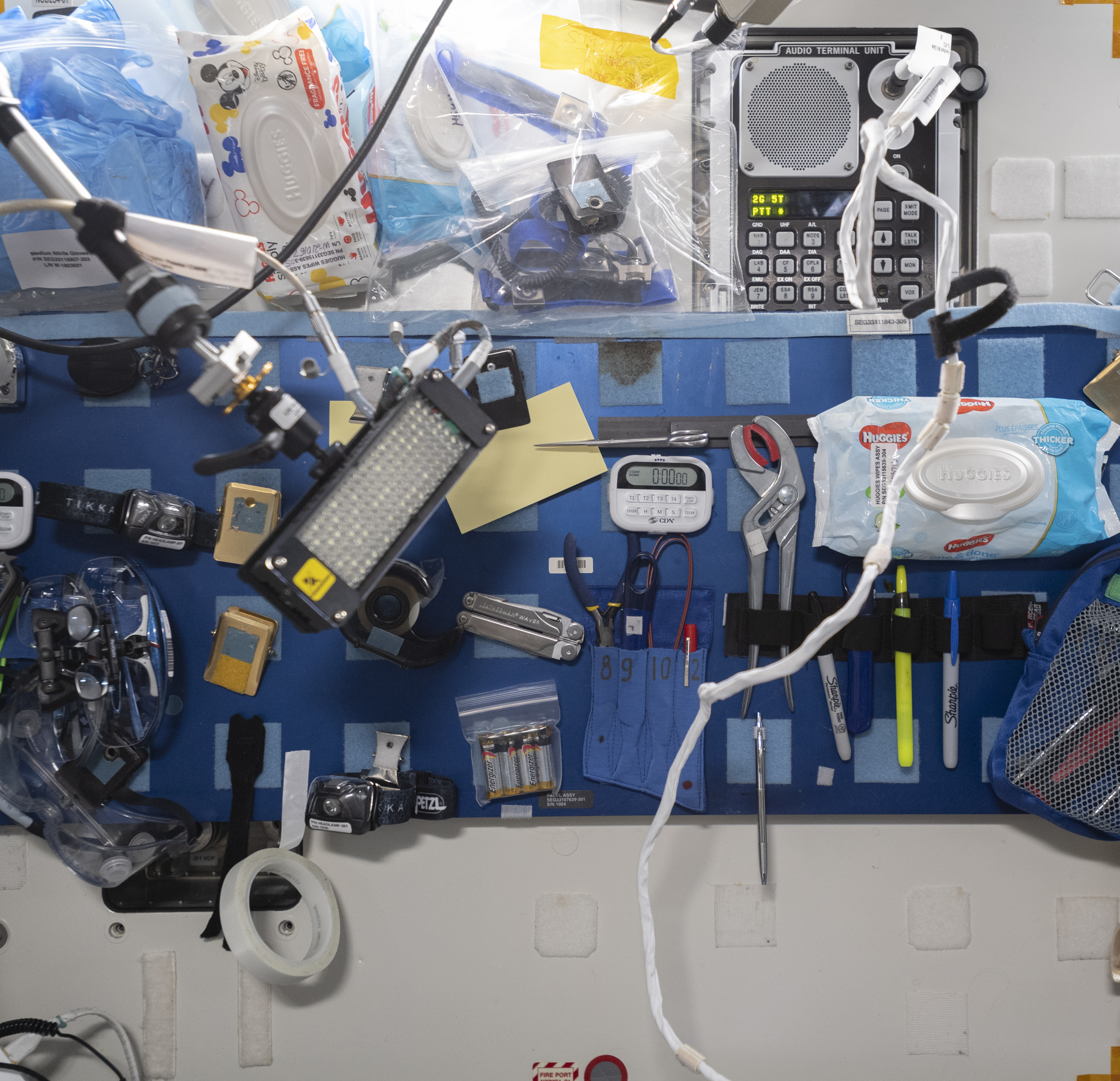

Supplement: S1 Dataset — (ZIP) [file pone.0304229.s002.zip › S03 - 19 - iss066e137904.jpg]

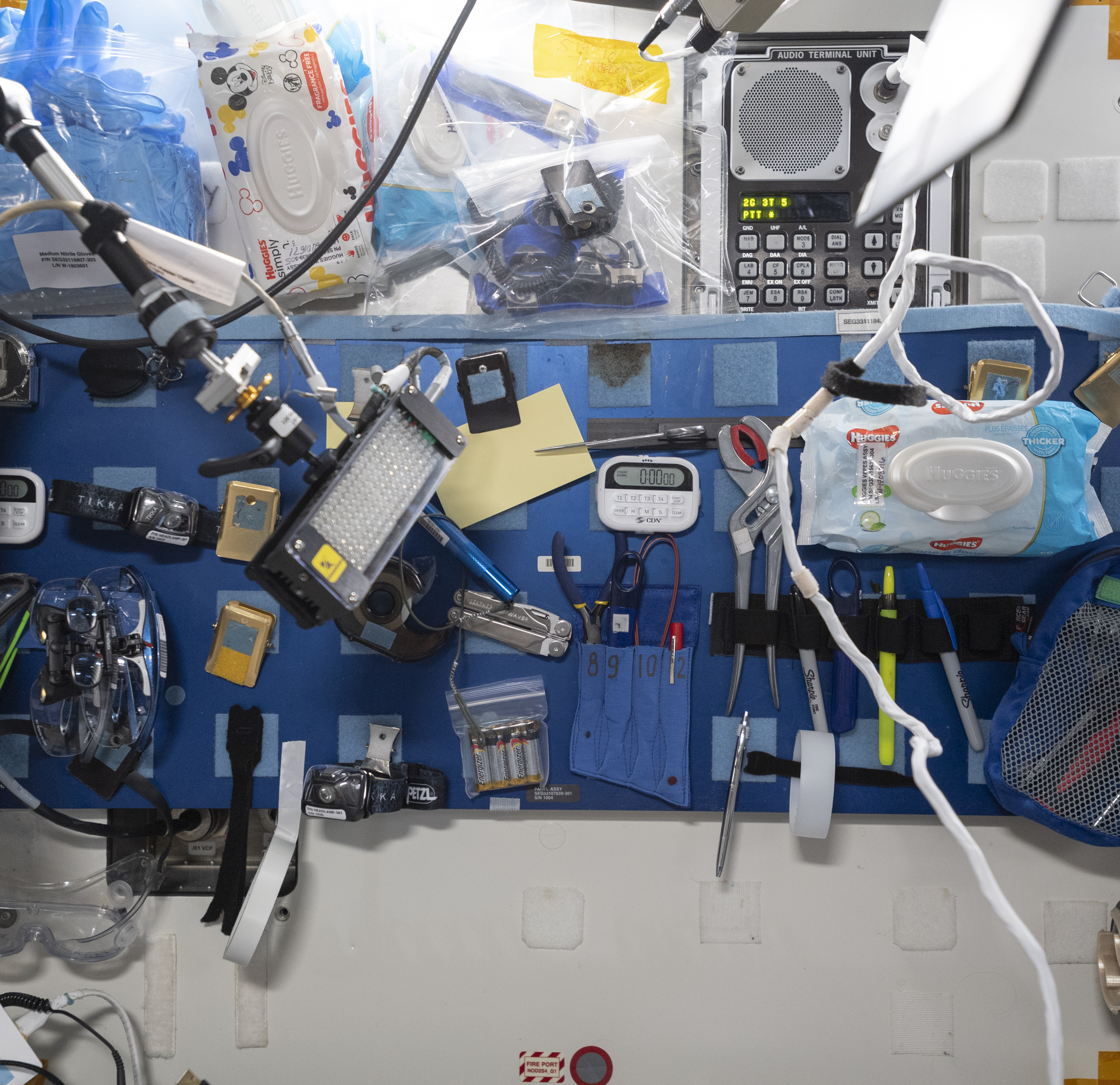

Supplement: S1 Dataset — (ZIP) [file pone.0304229.s002.zip › S03 - 20 - iss066e138184.jpg]

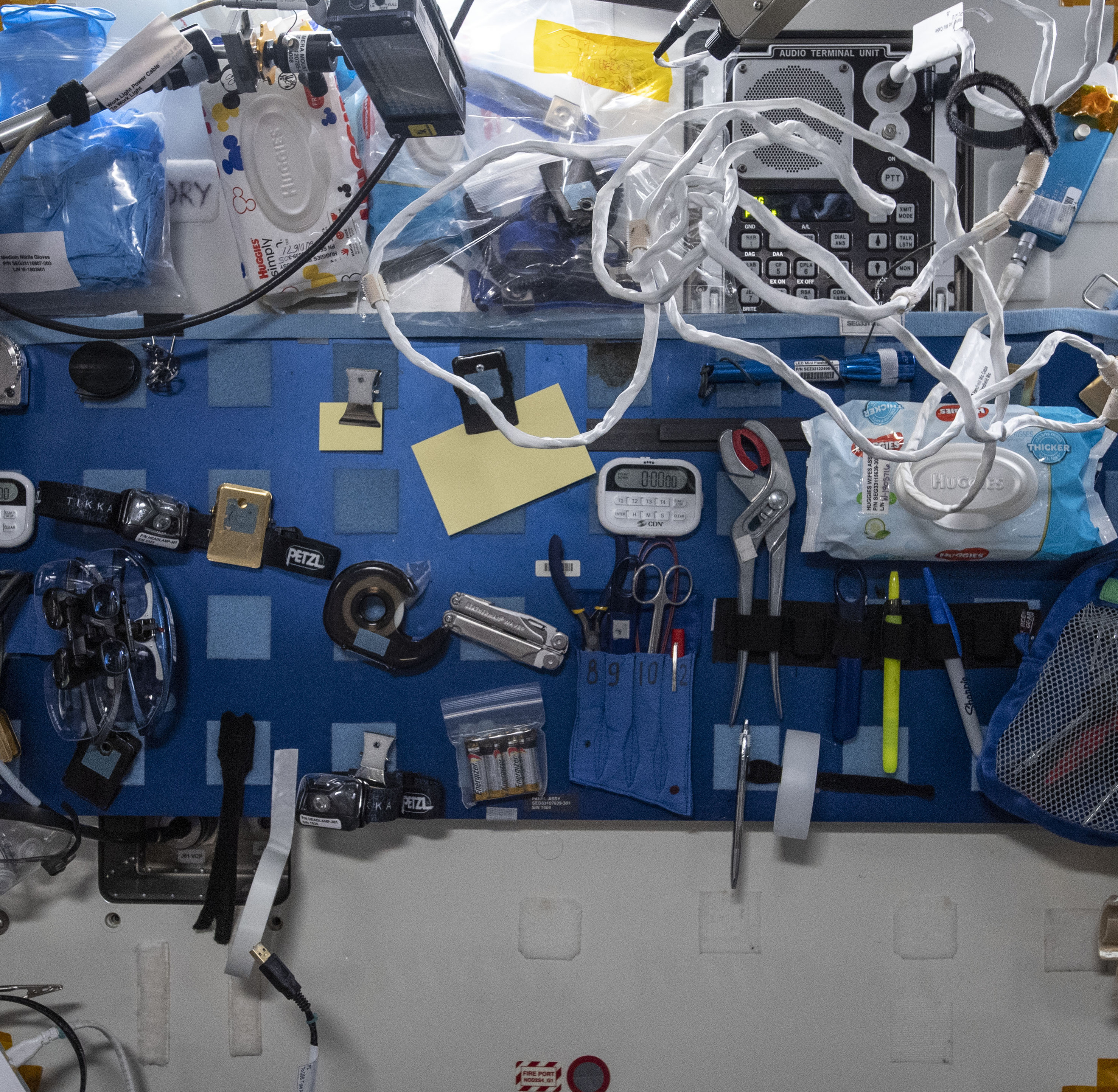

Supplement: S1 Dataset — (ZIP) [file pone.0304229.s002.zip › S03 - 21 - iss066e138210.jpg]

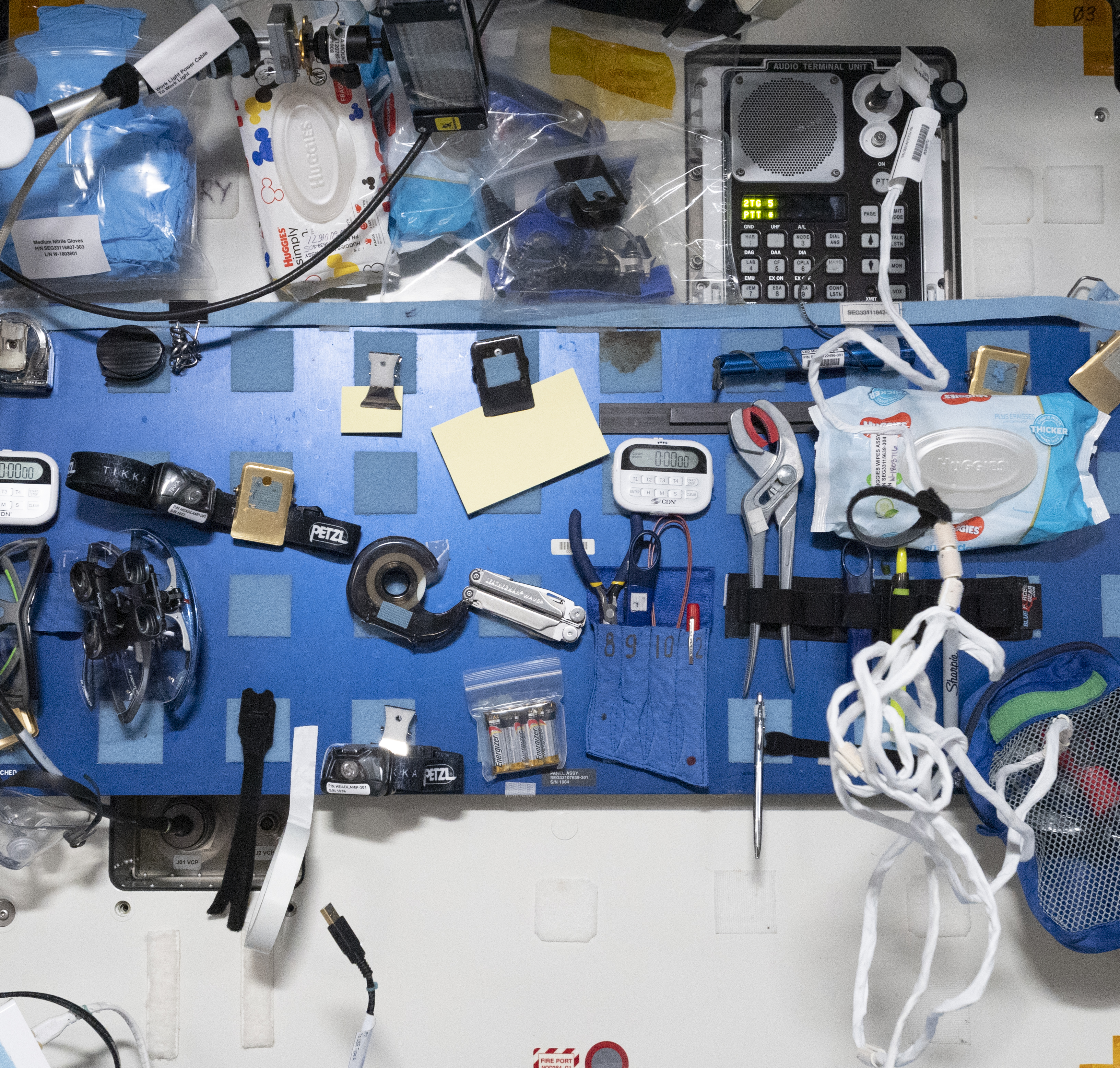

Supplement: S1 Dataset — (ZIP) [file pone.0304229.s002.zip › S03 - 22 - iss066e140897.jpg]

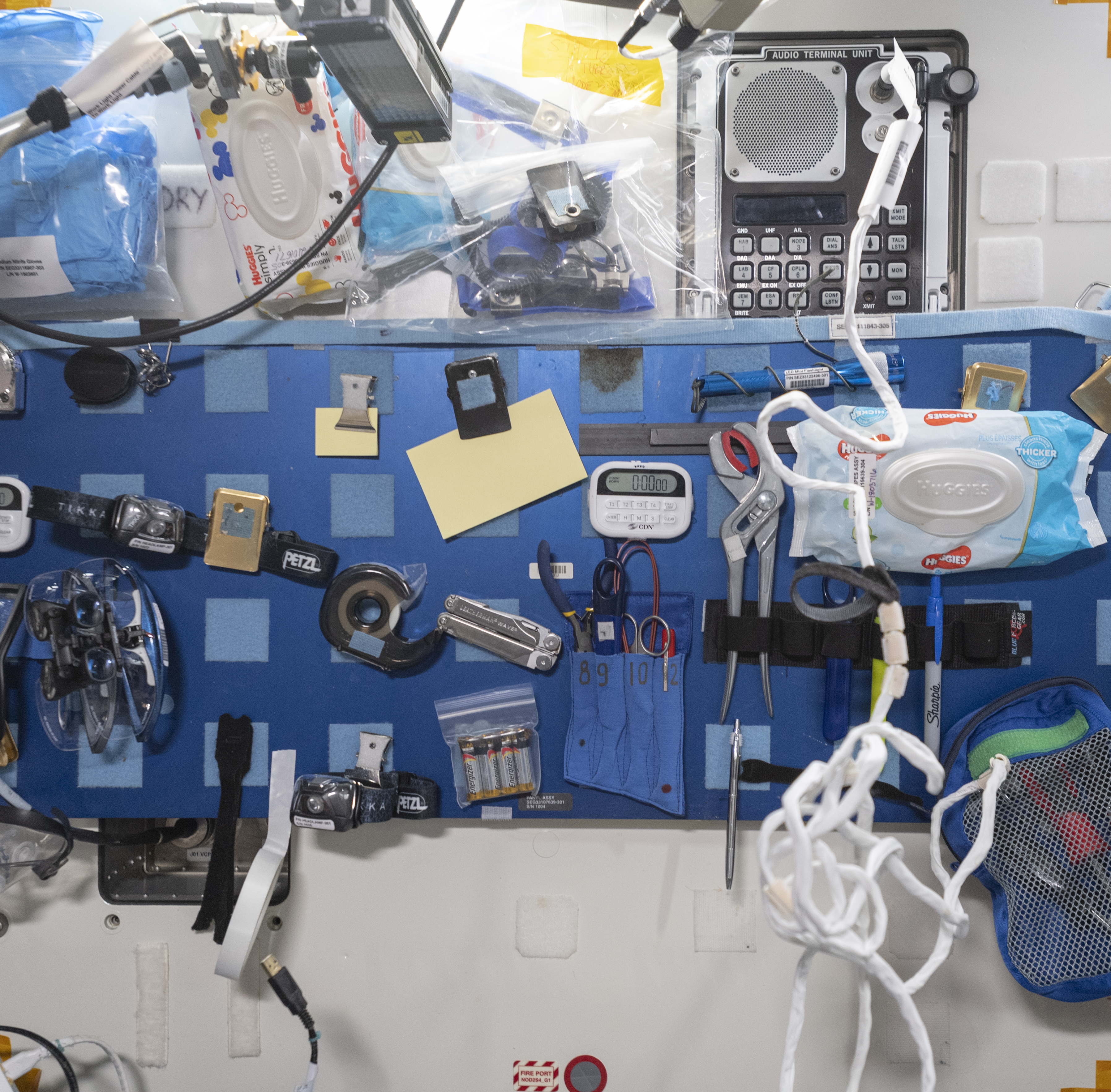

Supplement: S1 Dataset — (ZIP) [file pone.0304229.s002.zip › S03 - 23 - iss066e141169.jpg]

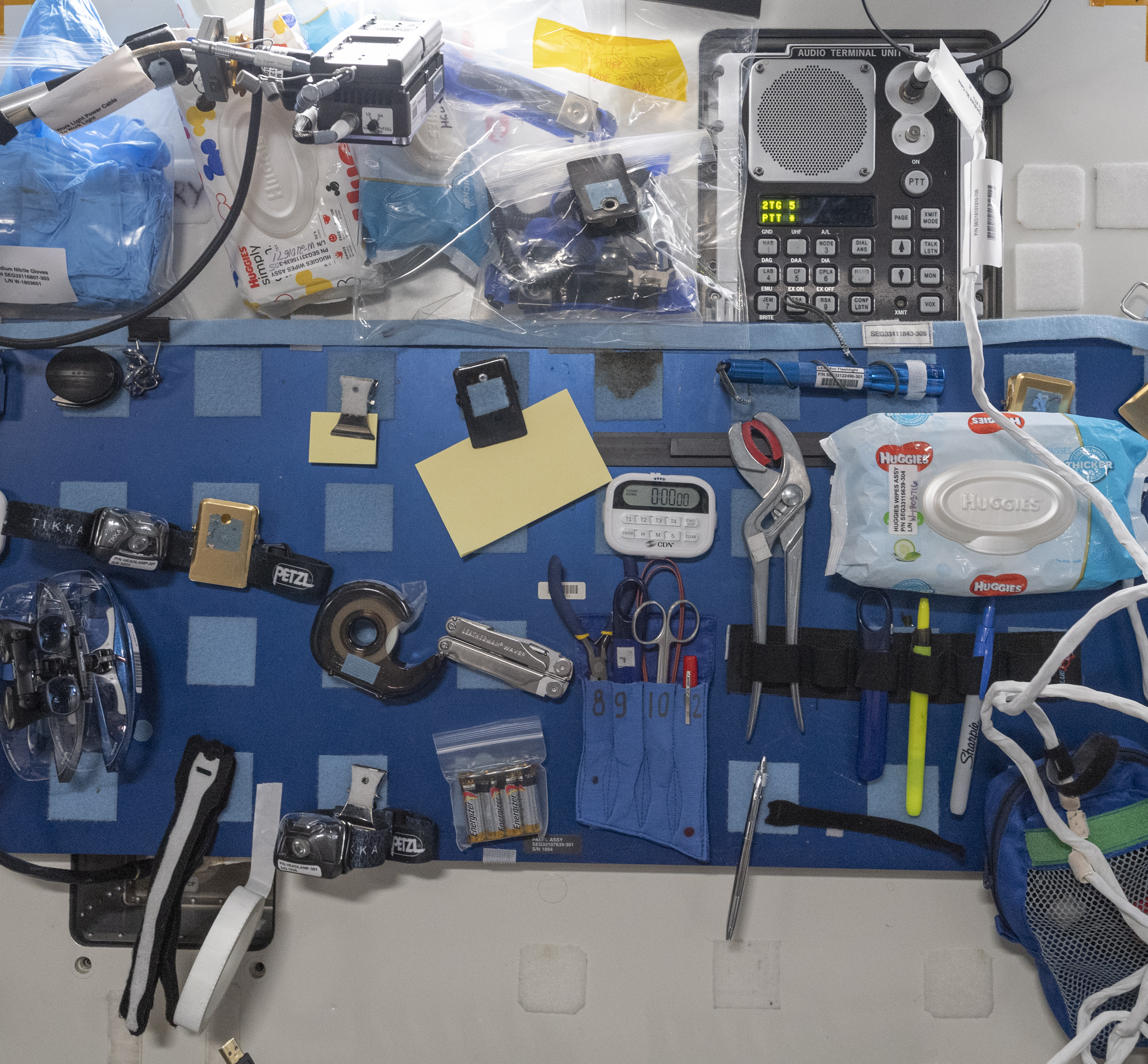

Supplement: S1 Dataset — (ZIP) [file pone.0304229.s002.zip › S03 - 24 - iss066e140917.jpg]

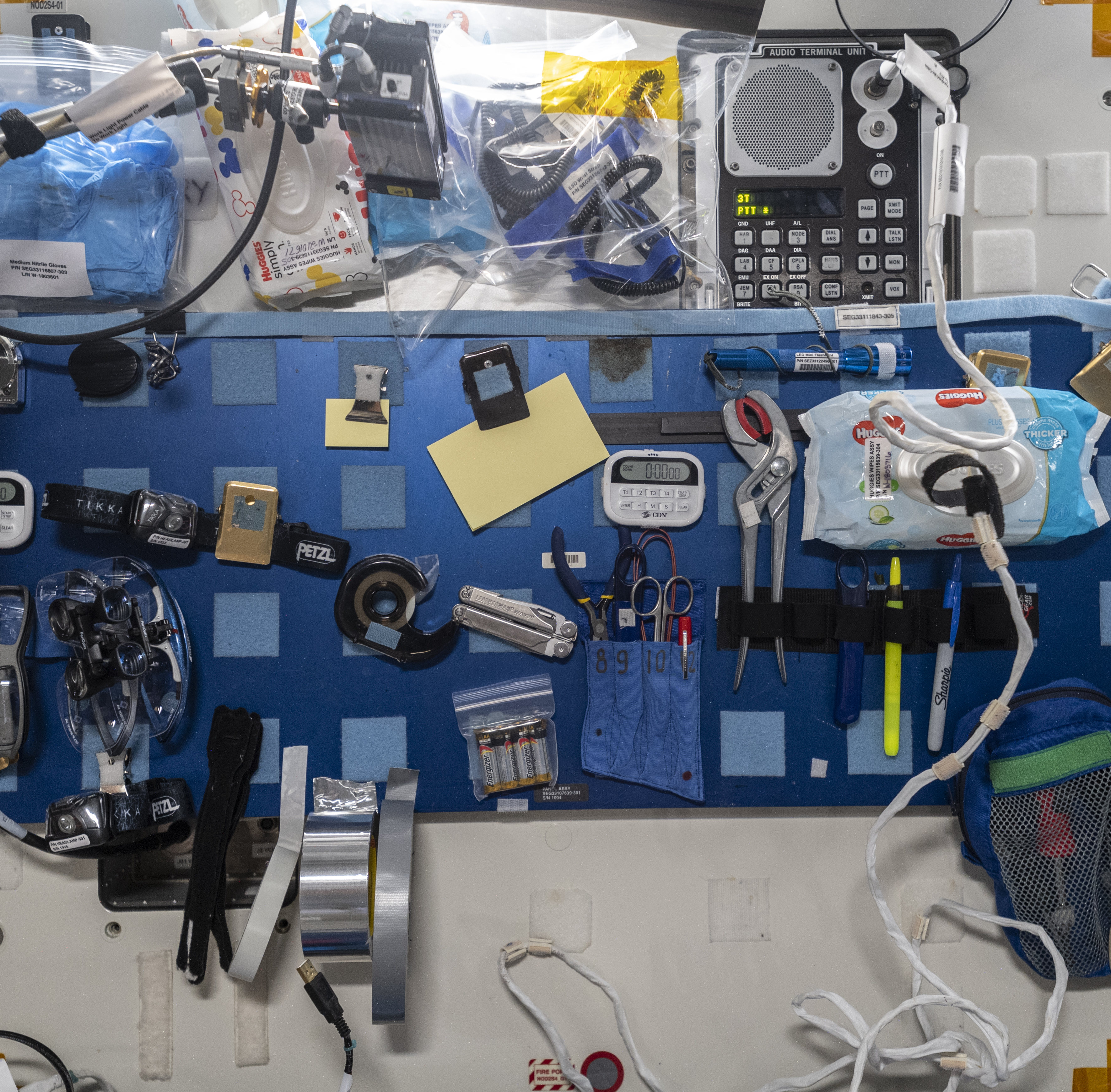

Supplement: S1 Dataset — (ZIP) [file pone.0304229.s002.zip › S03 - 25 - iss066e143019.jpg]

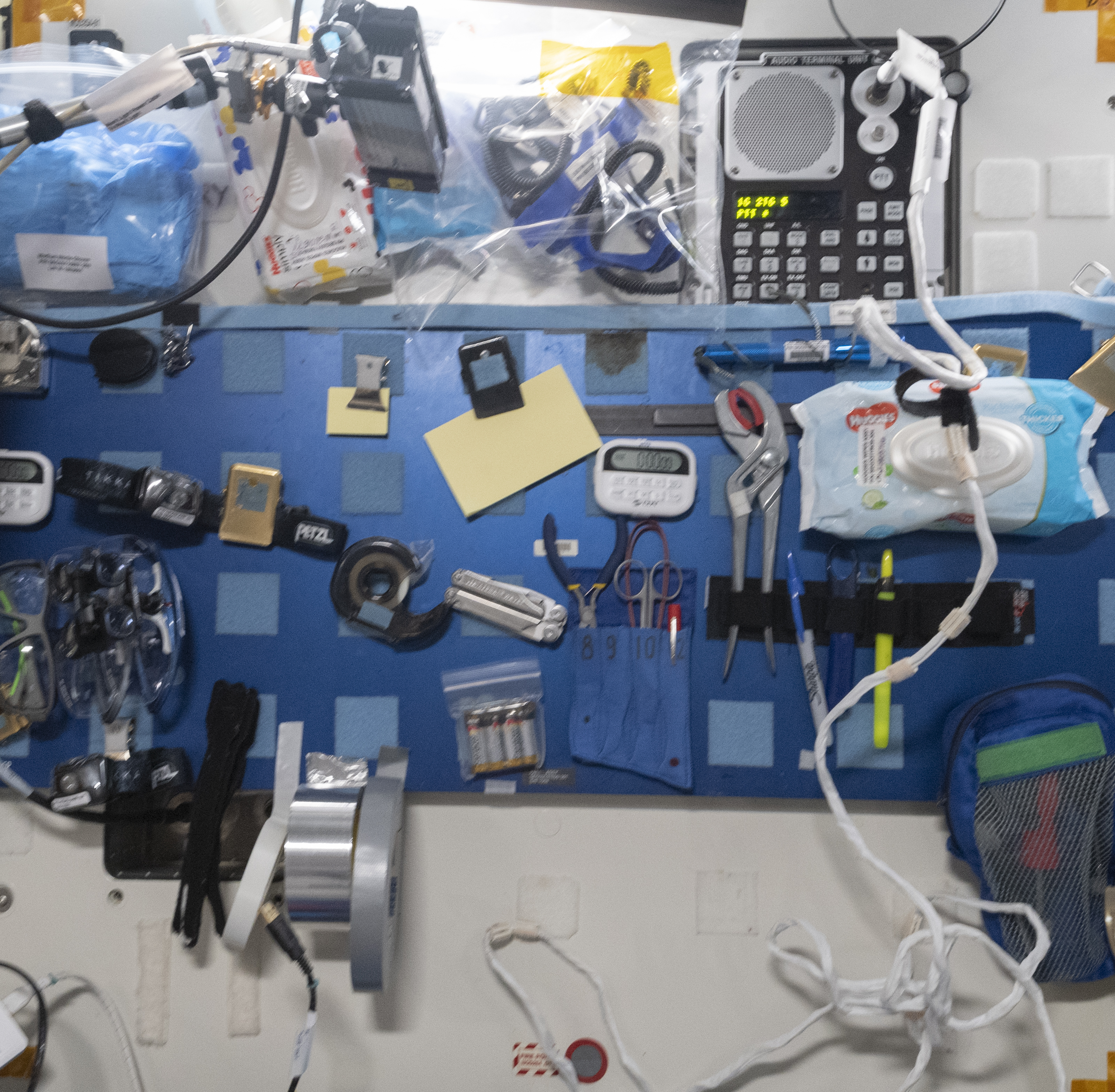

Supplement: S1 Dataset — (ZIP) [file pone.0304229.s002.zip › S03 - 26 - iss066e144198.jpg]

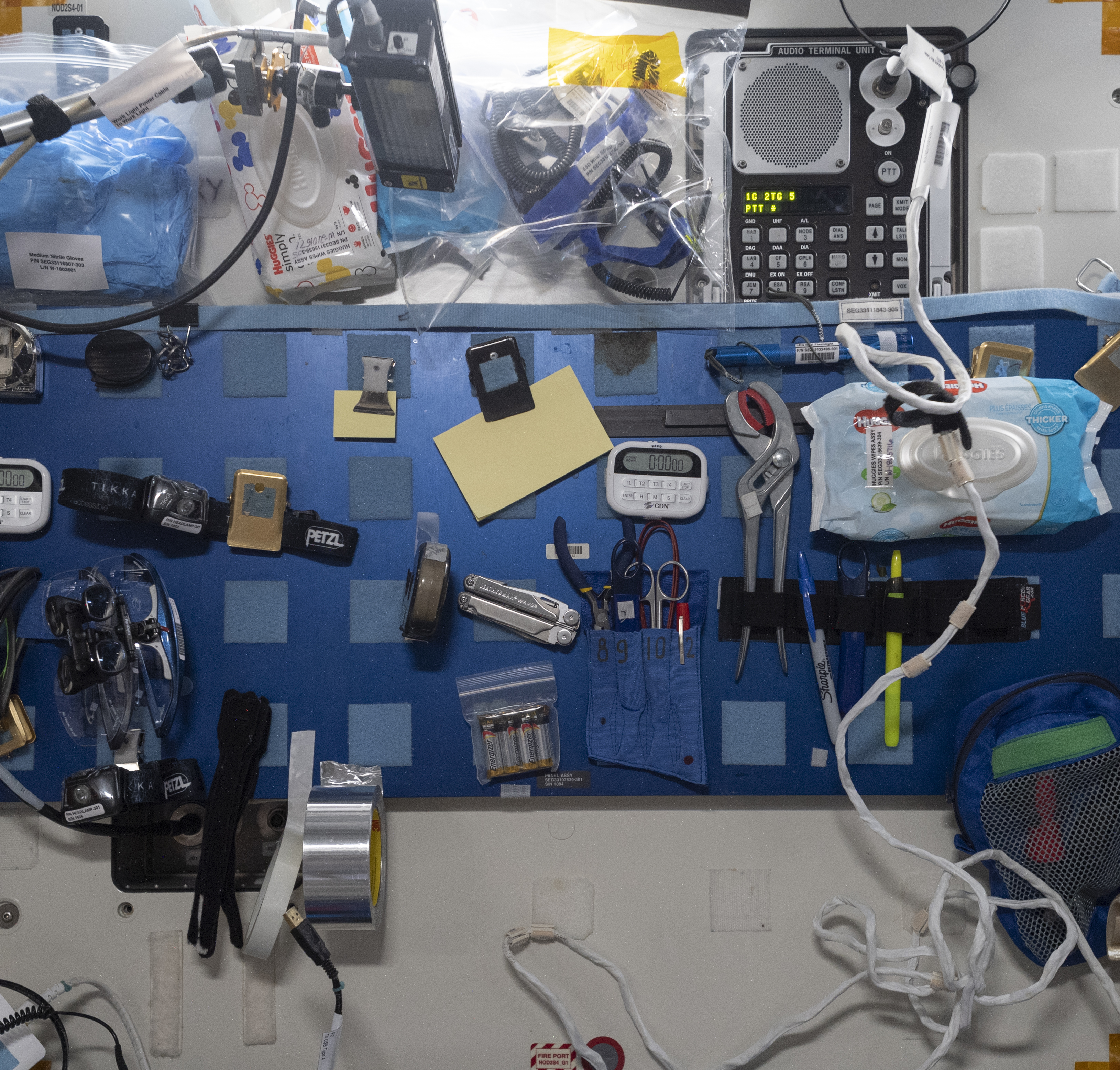

Supplement: S1 Dataset — (ZIP) [file pone.0304229.s002.zip › S03 - 27 - iss066e145793.jpg]

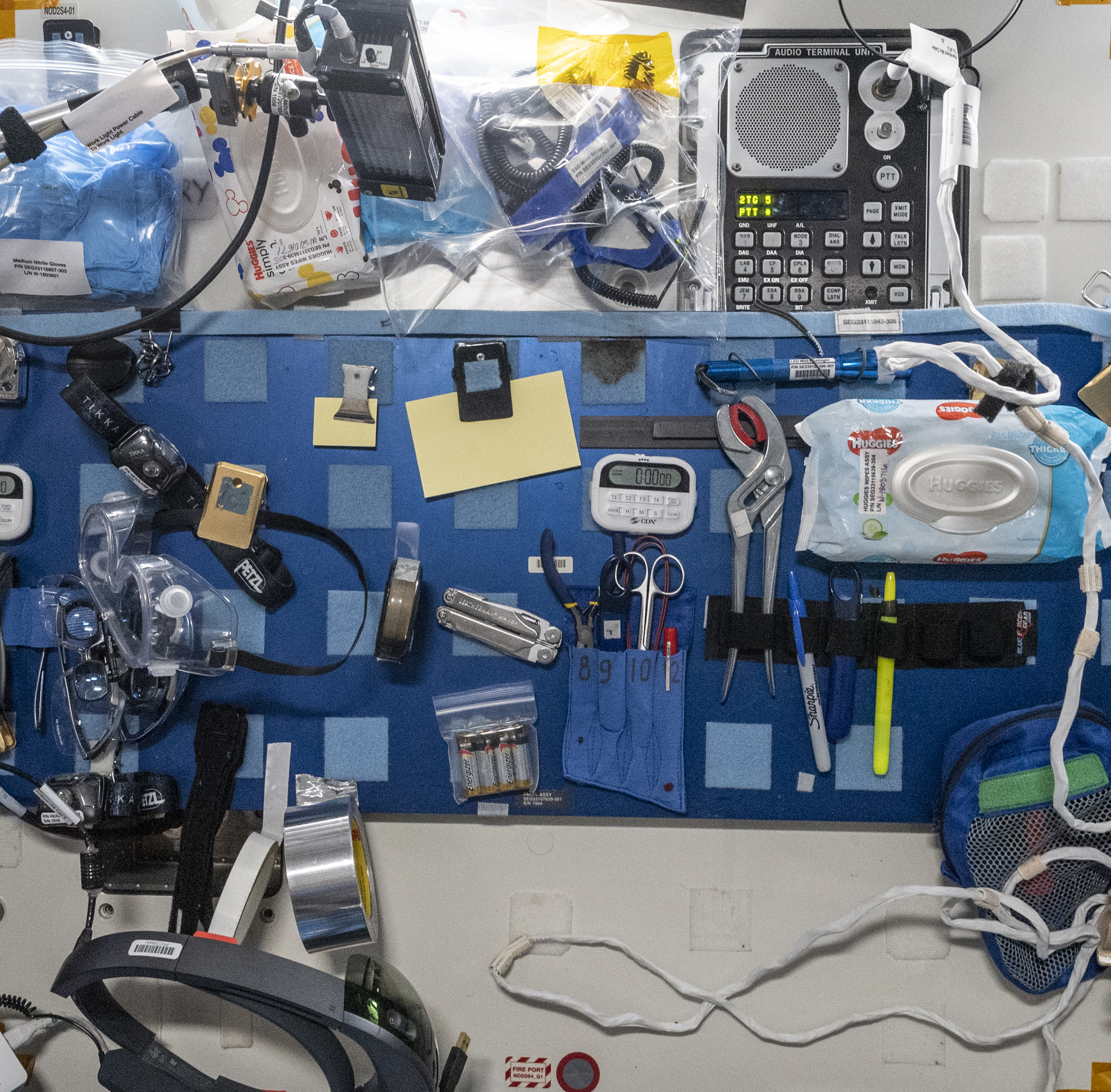

Supplement: S1 Dataset — (ZIP) [file pone.0304229.s002.zip › S03 - 28 - iss066e145975.jpg]

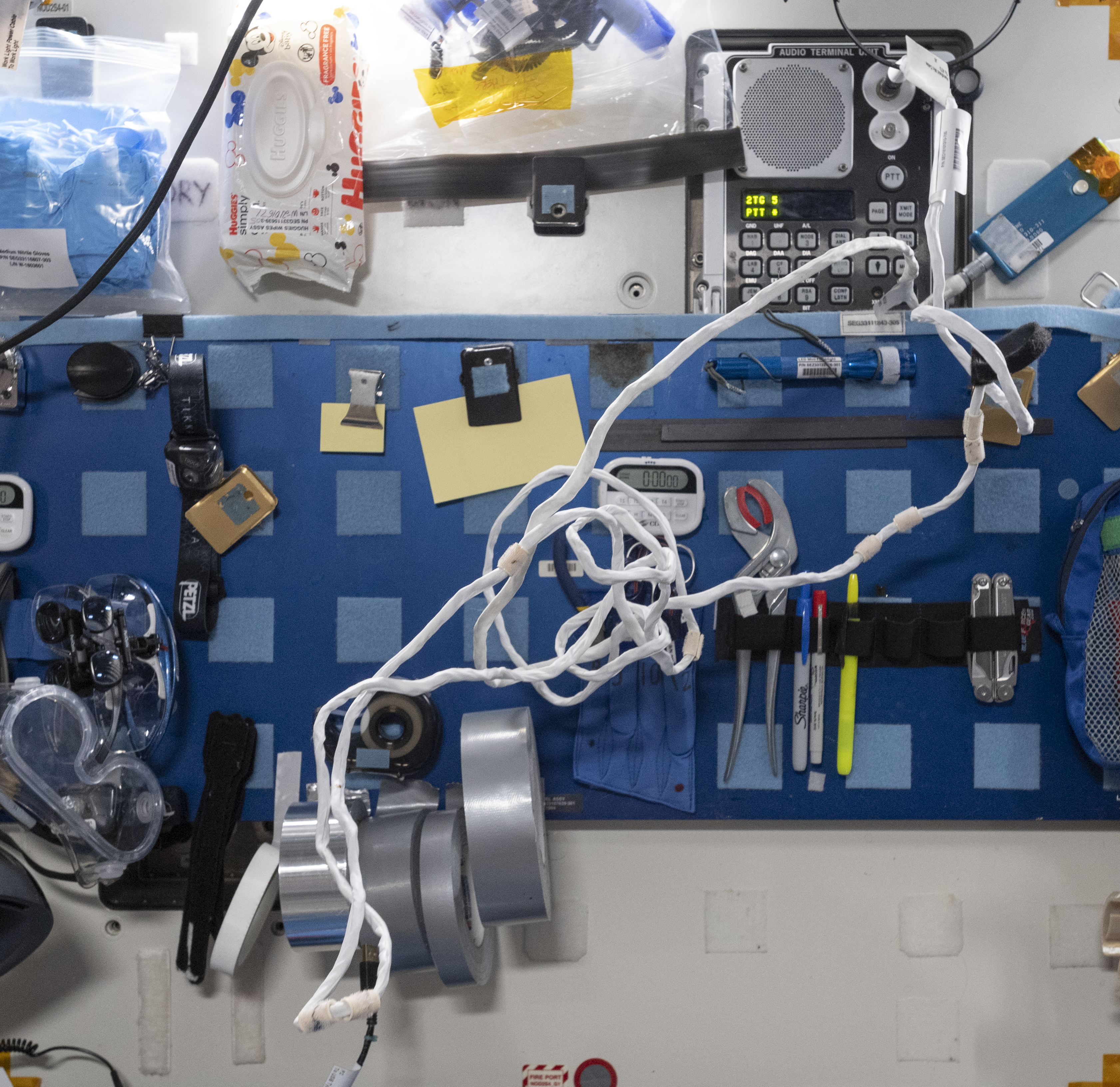

Supplement: S1 Dataset — (ZIP) [file pone.0304229.s002.zip › S03 - 29 - iss066e146553-2.jpg]

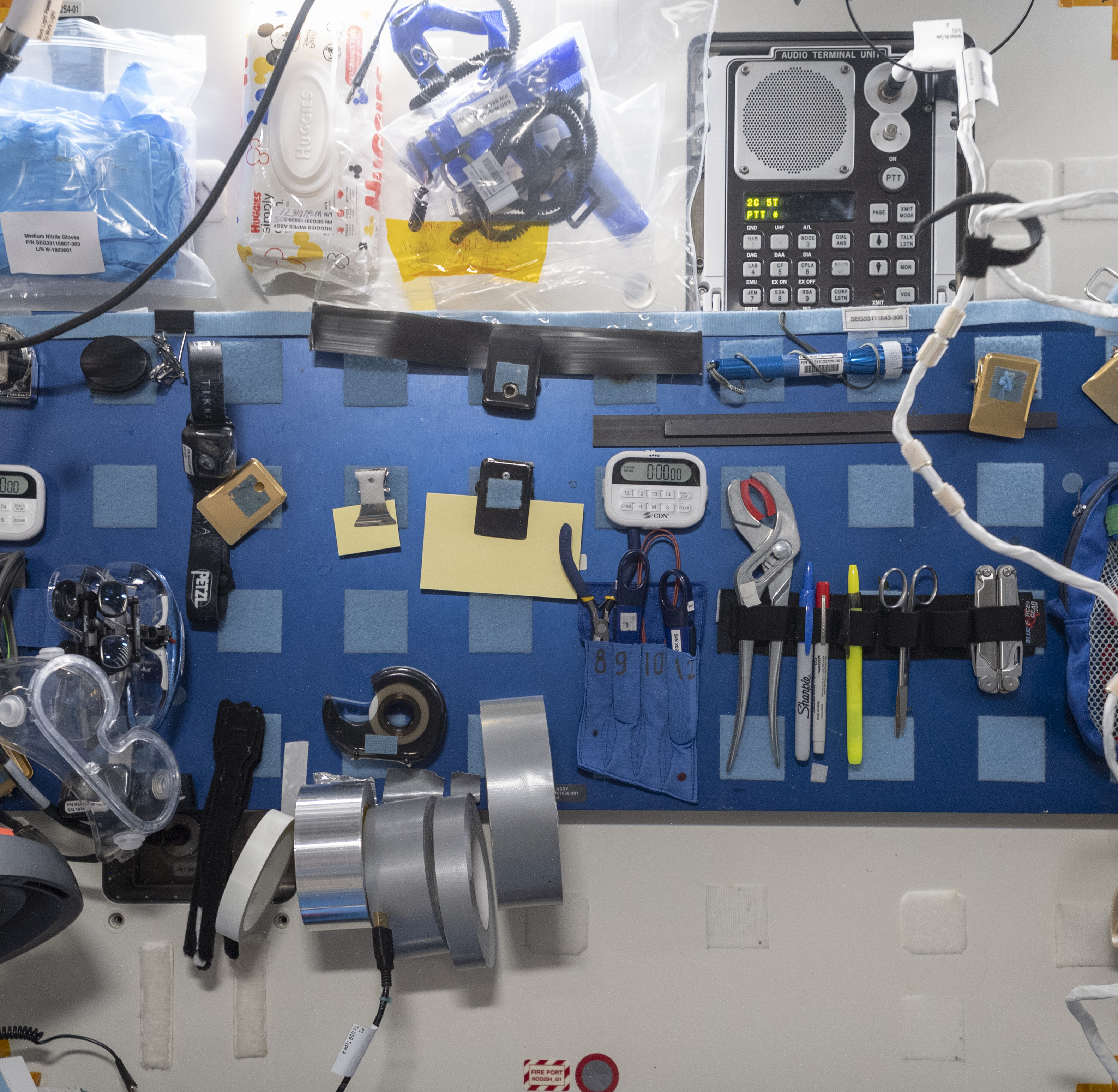

Supplement: S1 Dataset — (ZIP) [file pone.0304229.s002.zip › S03 - 30 - iss066e146486.jpg]

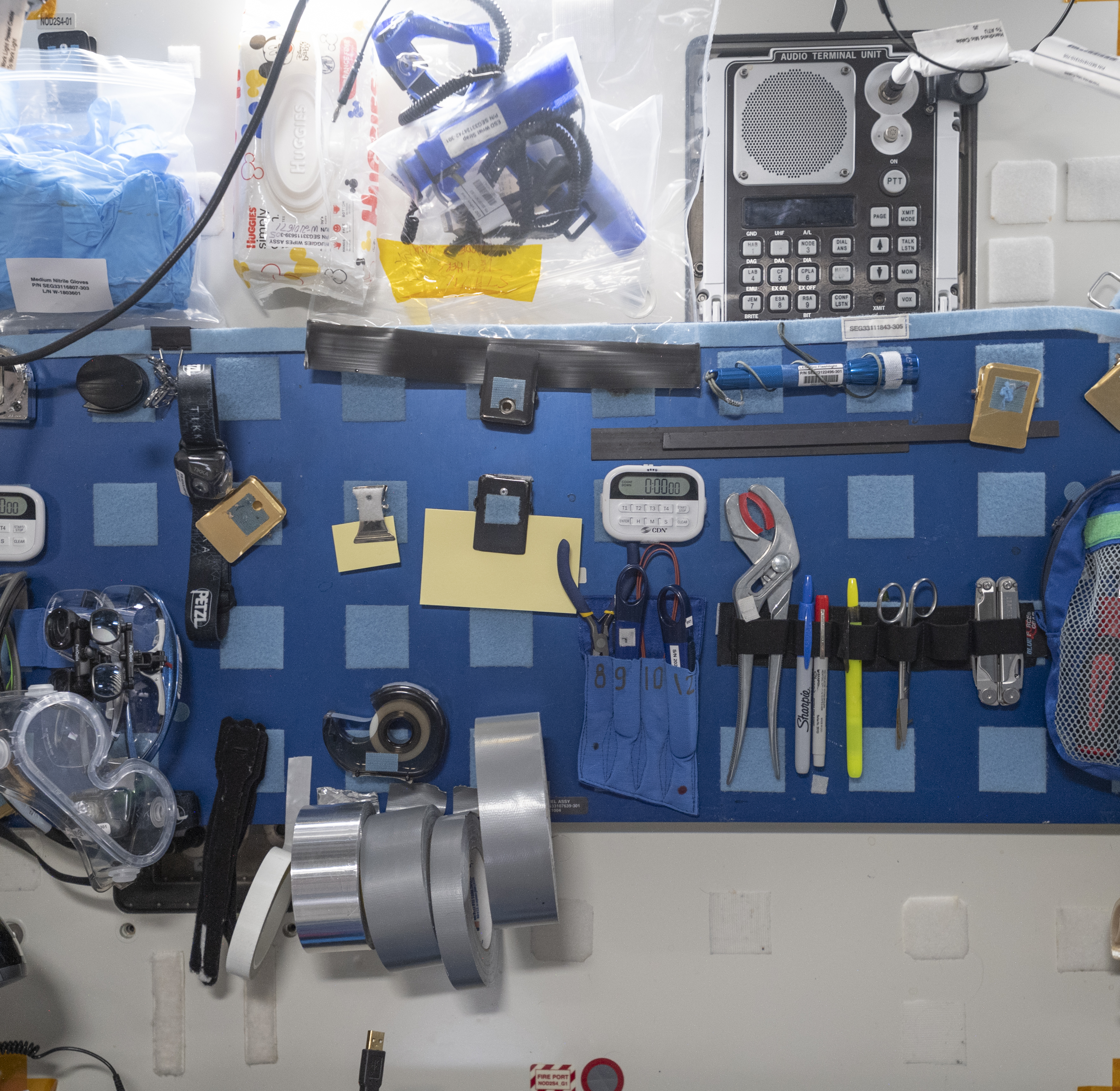

Supplement: S1 Dataset — (ZIP) [file pone.0304229.s002.zip › S03 - 31 - iss066e146499.jpg]

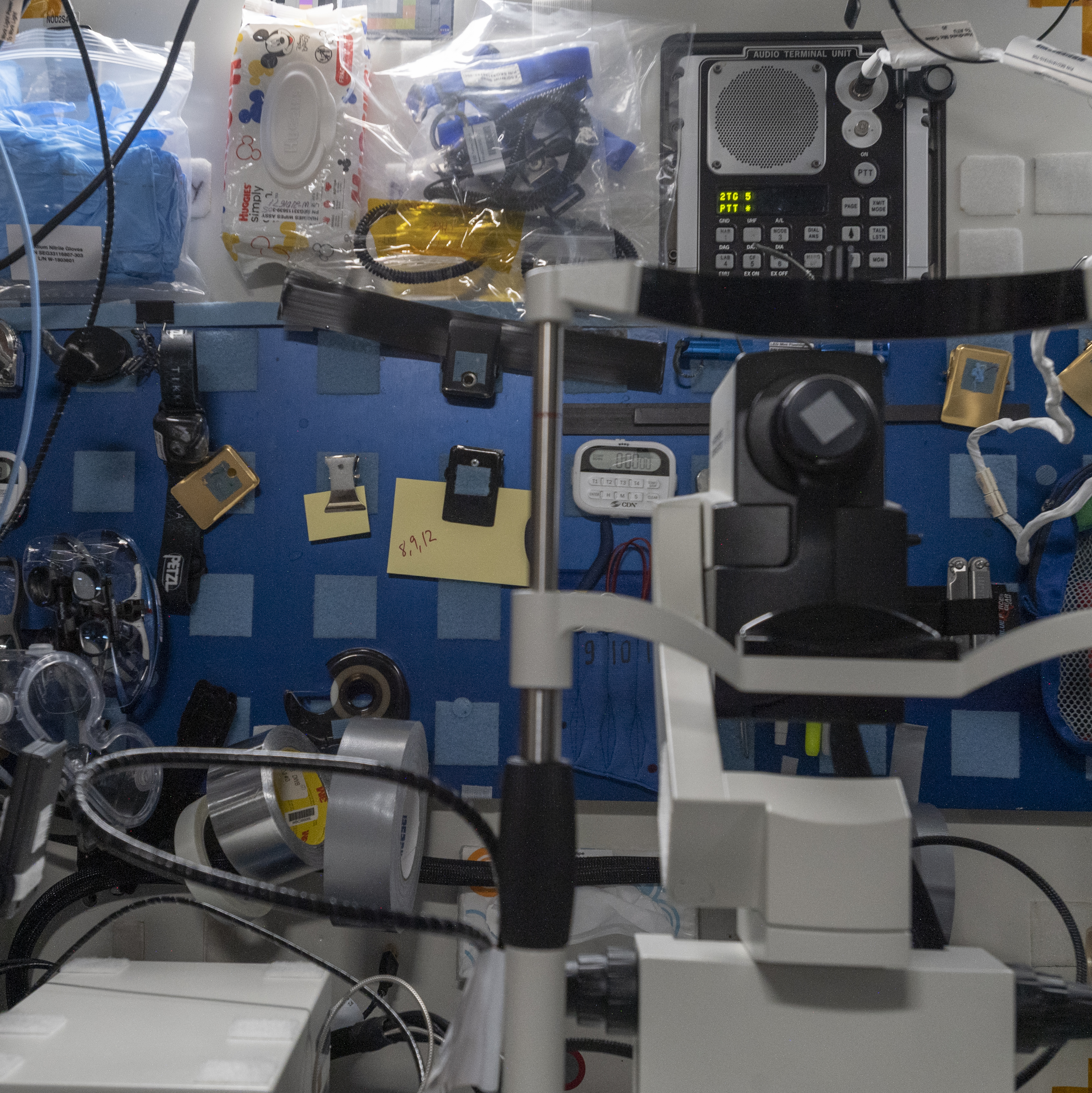

Supplement: S1 Dataset — (ZIP) [file pone.0304229.s002.zip › S03 - 32 - iss066e146532.jpg]

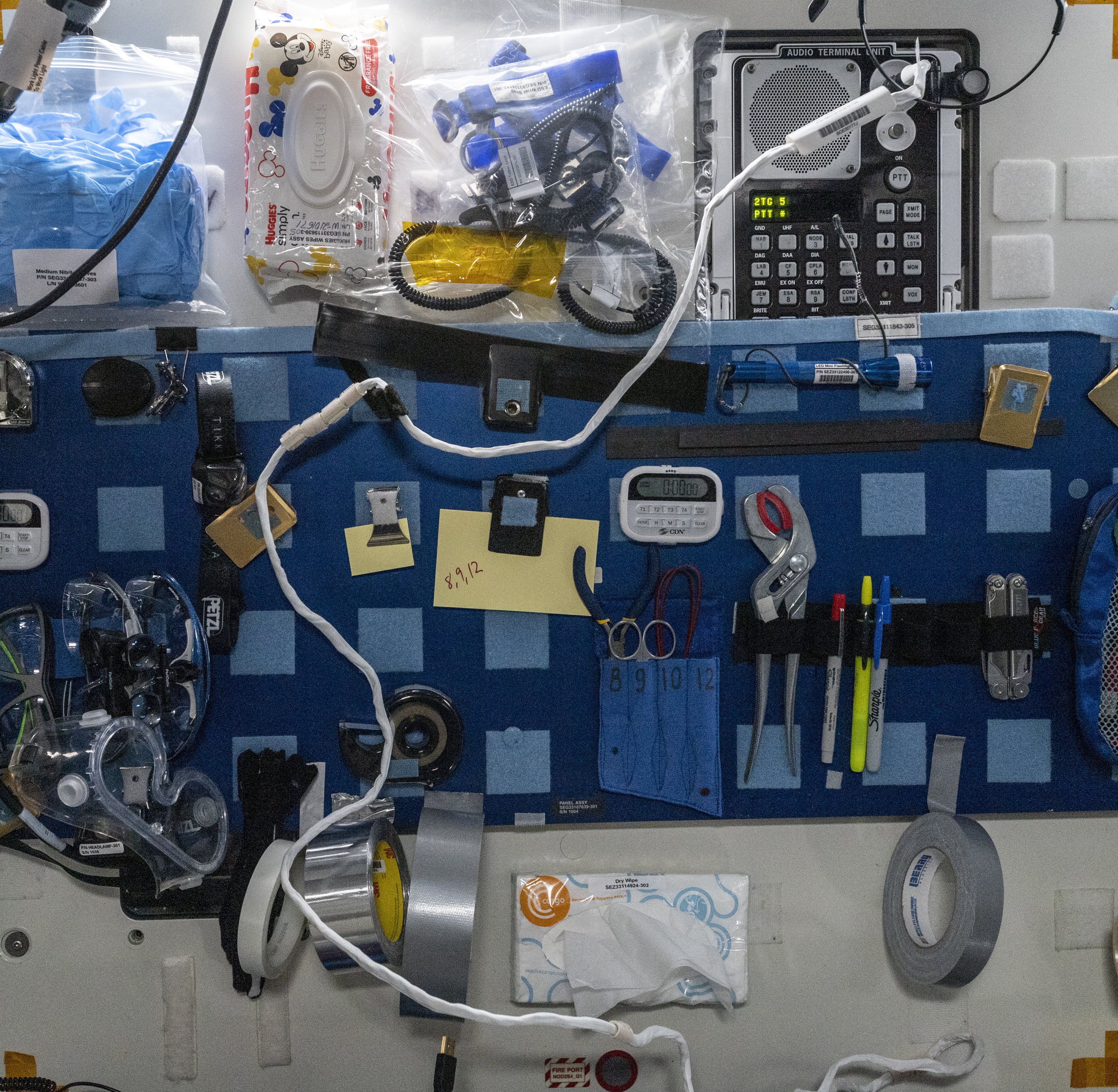

Supplement: S1 Dataset — (ZIP) [file pone.0304229.s002.zip › S03 - 33 - iss066e151981.jpg]

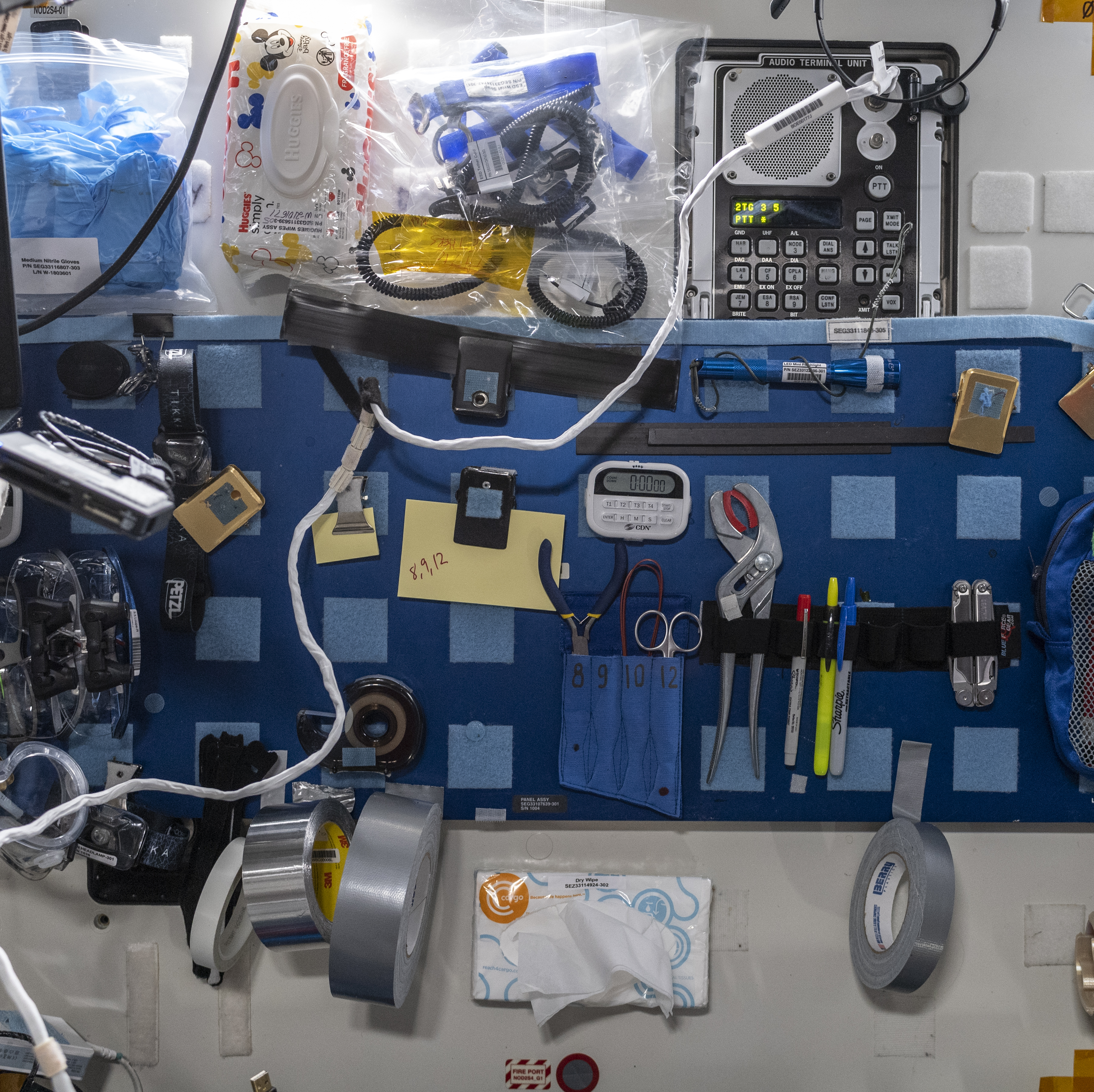

Supplement: S1 Dataset — (ZIP) [file pone.0304229.s002.zip › S03 - 34 - iss066e152107.jpg]

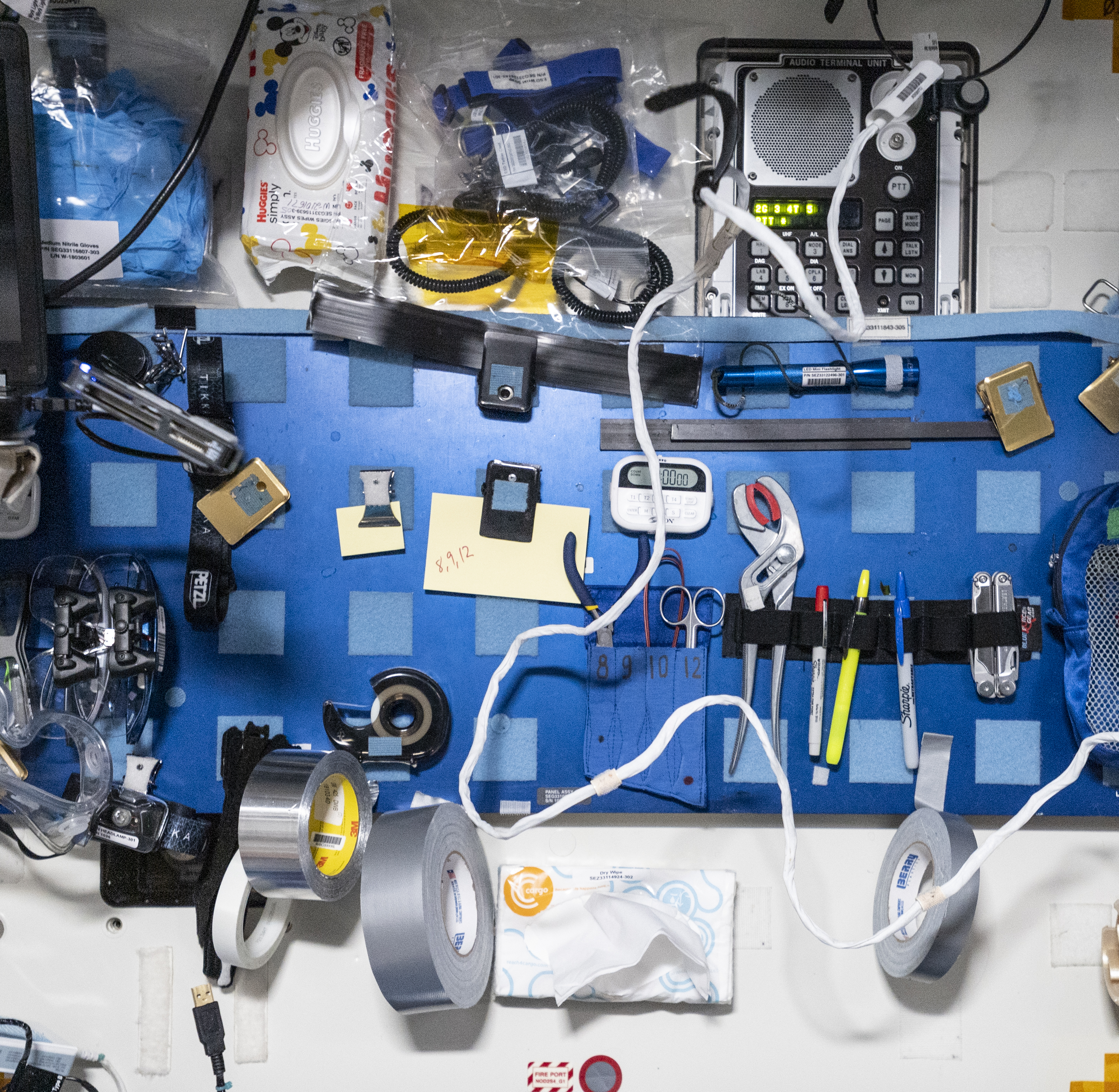

Supplement: S1 Dataset — (ZIP) [file pone.0304229.s002.zip › S03 - 35 - iss066e152770.jpg]

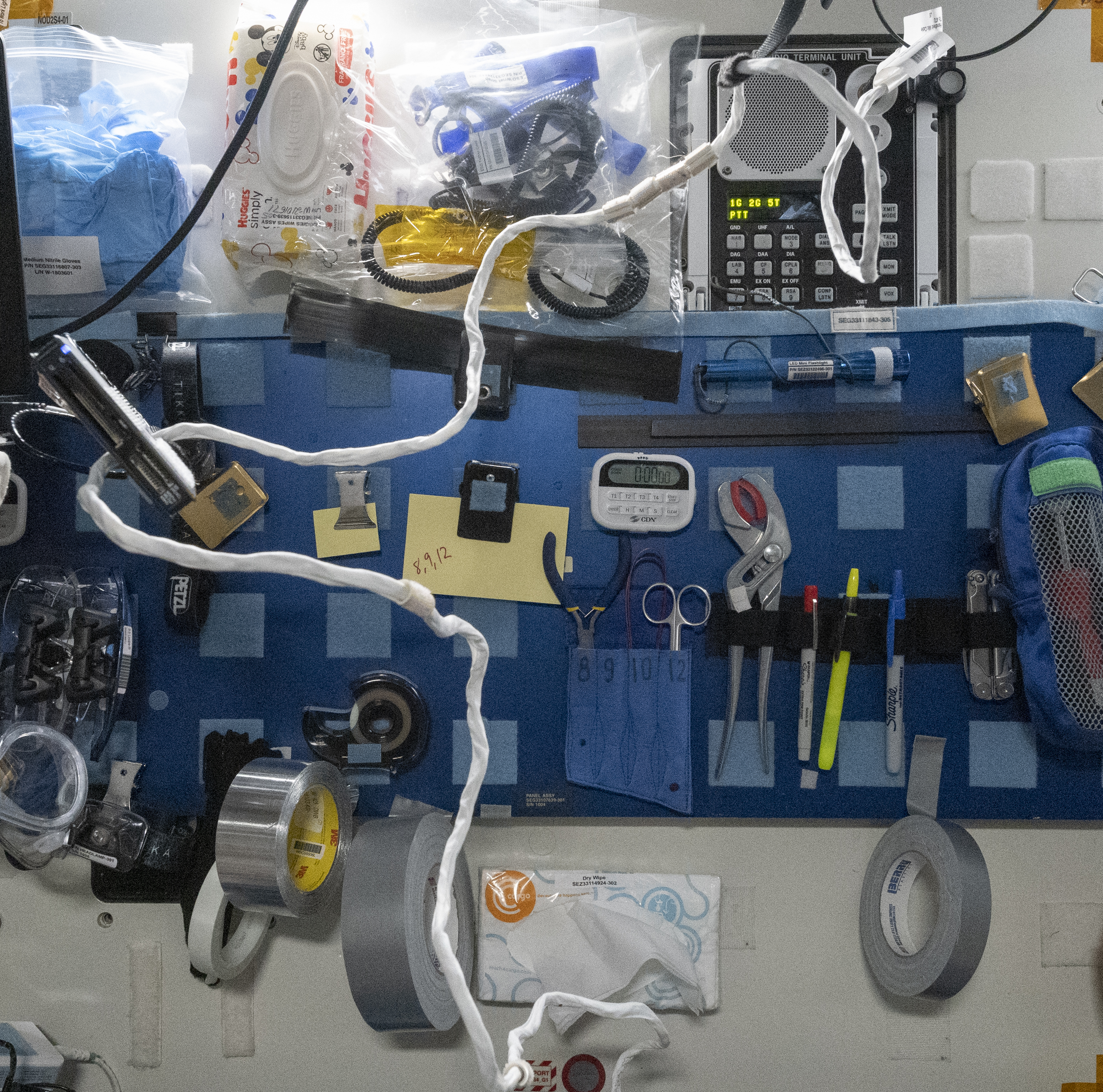

Supplement: S1 Dataset — (ZIP) [file pone.0304229.s002.zip › S03 - 36 - iss066e153134.jpg]

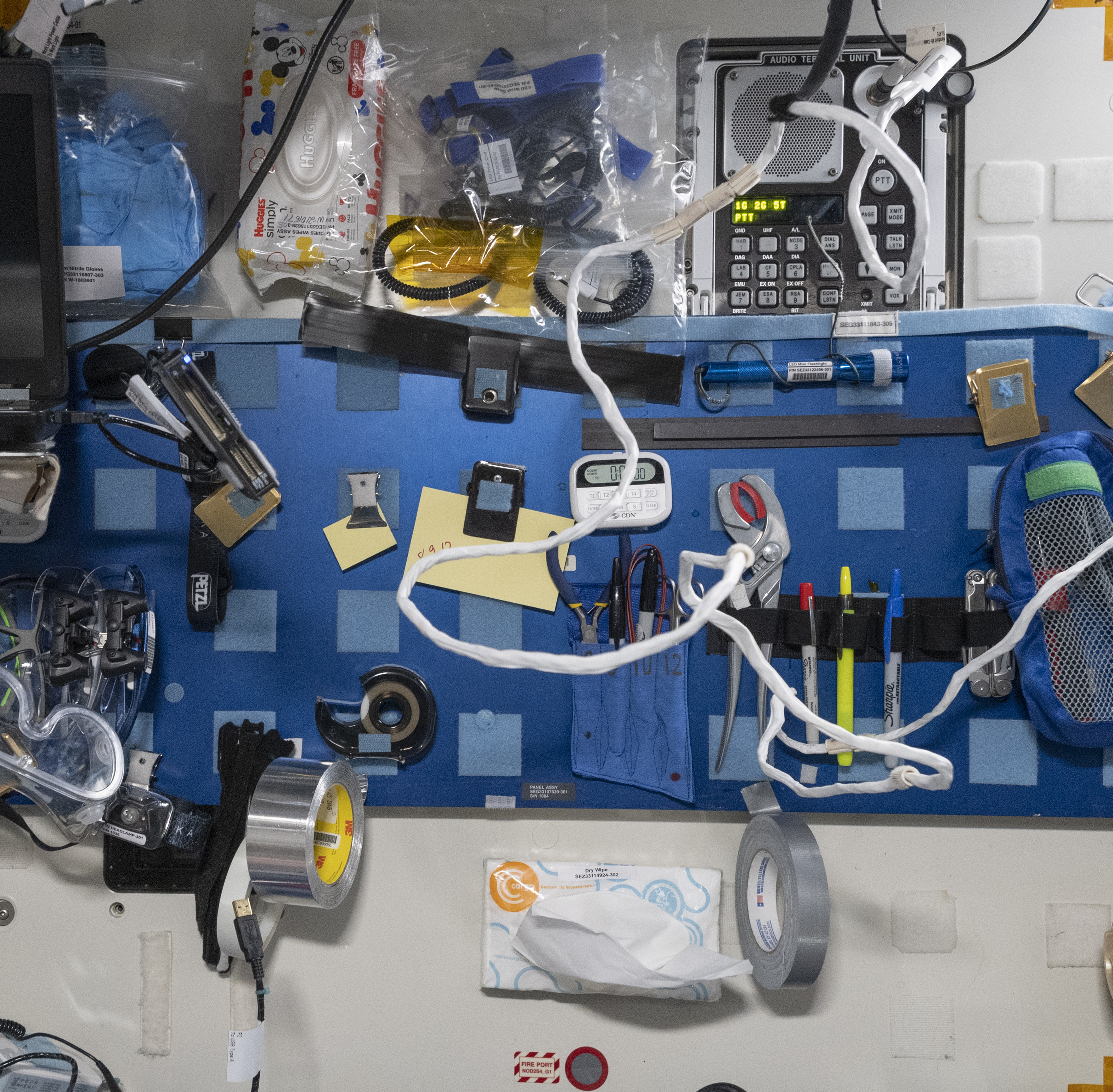

Supplement: S1 Dataset — (ZIP) [file pone.0304229.s002.zip › S03 - 37 - iss066e153156.jpg]

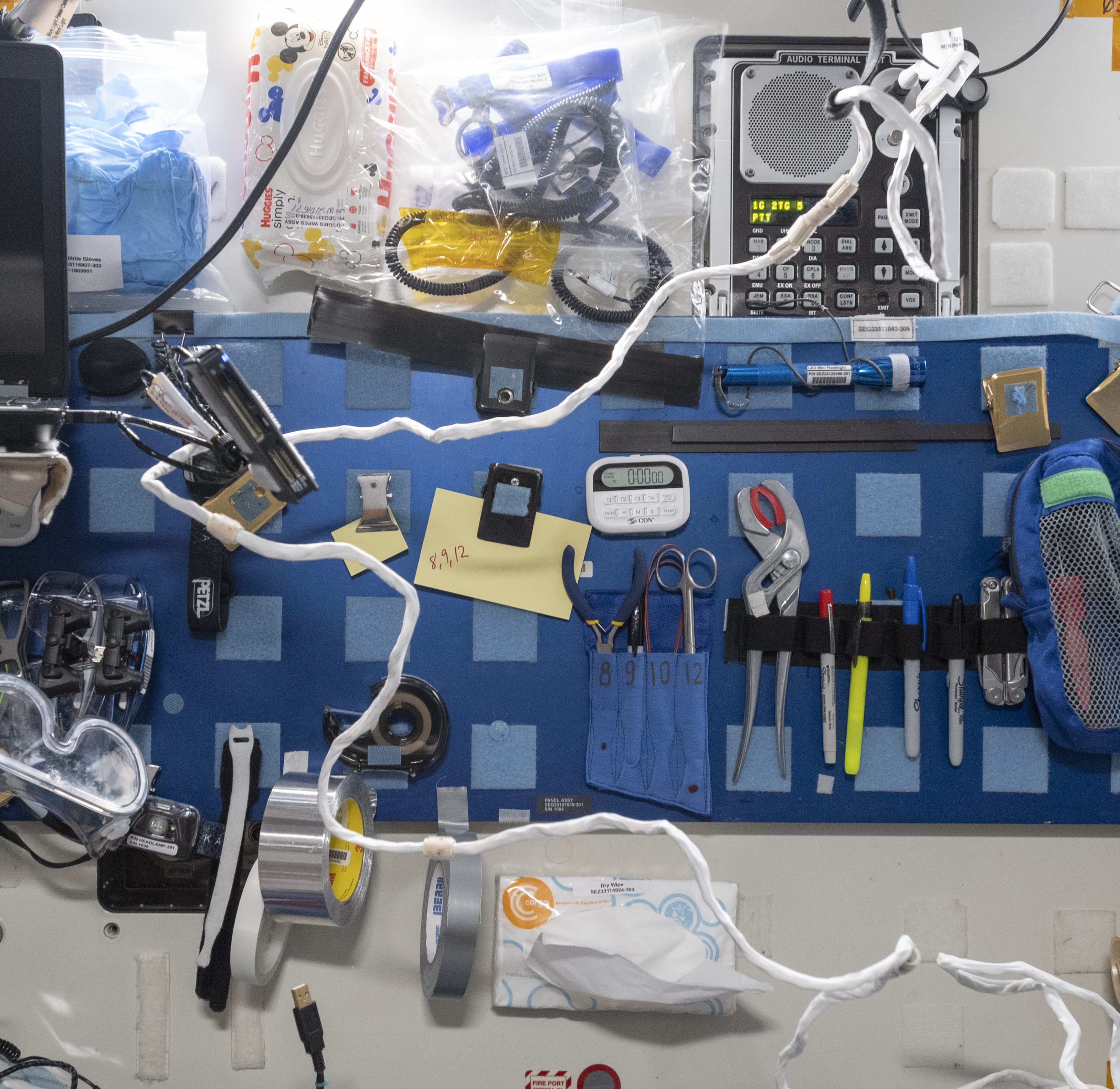

Supplement: S1 Dataset — (ZIP) [file pone.0304229.s002.zip › S03 - 38 - iss066e153248.jpg]

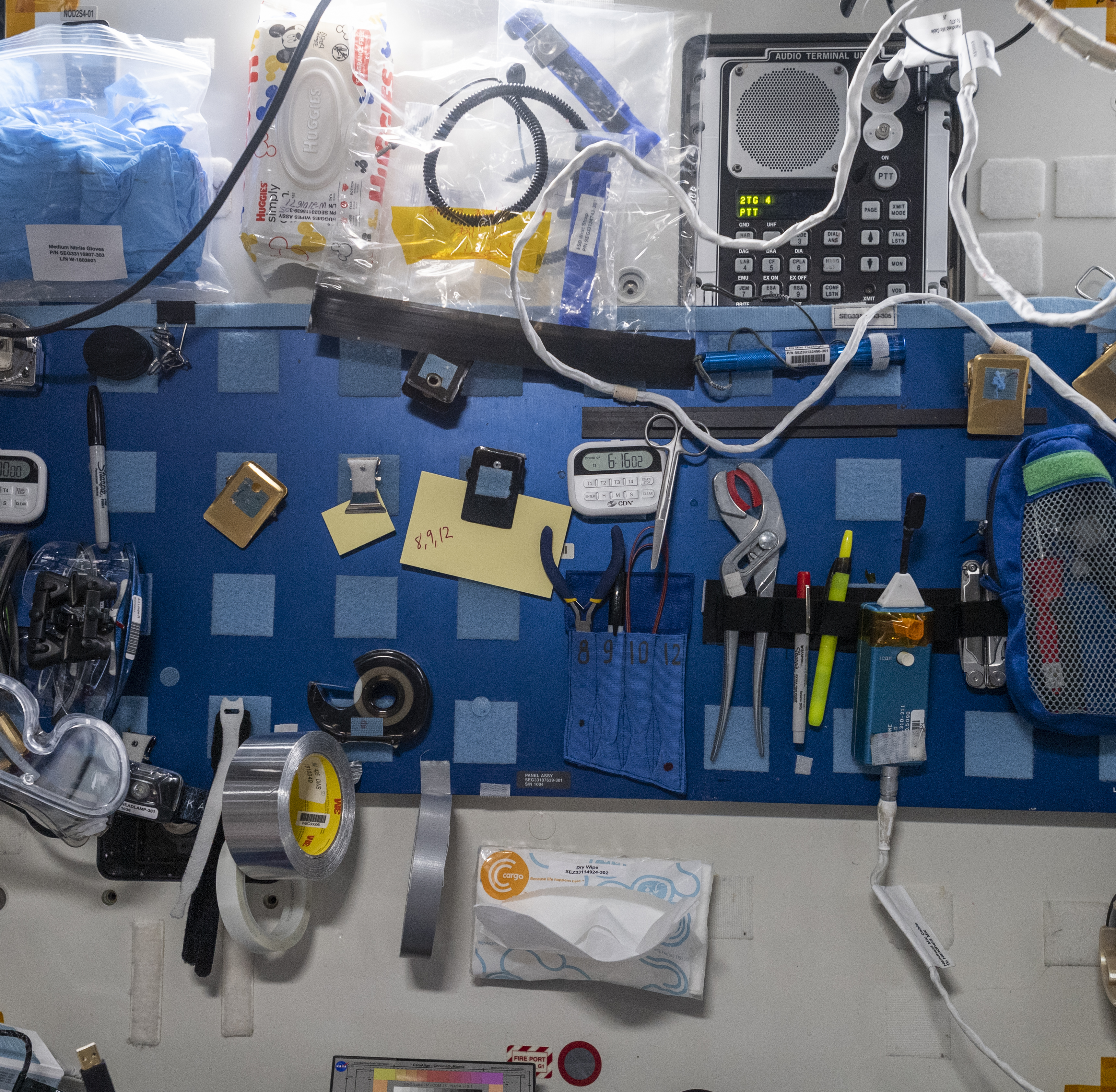

Supplement: S1 Dataset — (ZIP) [file pone.0304229.s002.zip › S03 - 39 - iss066e155007.jpg]

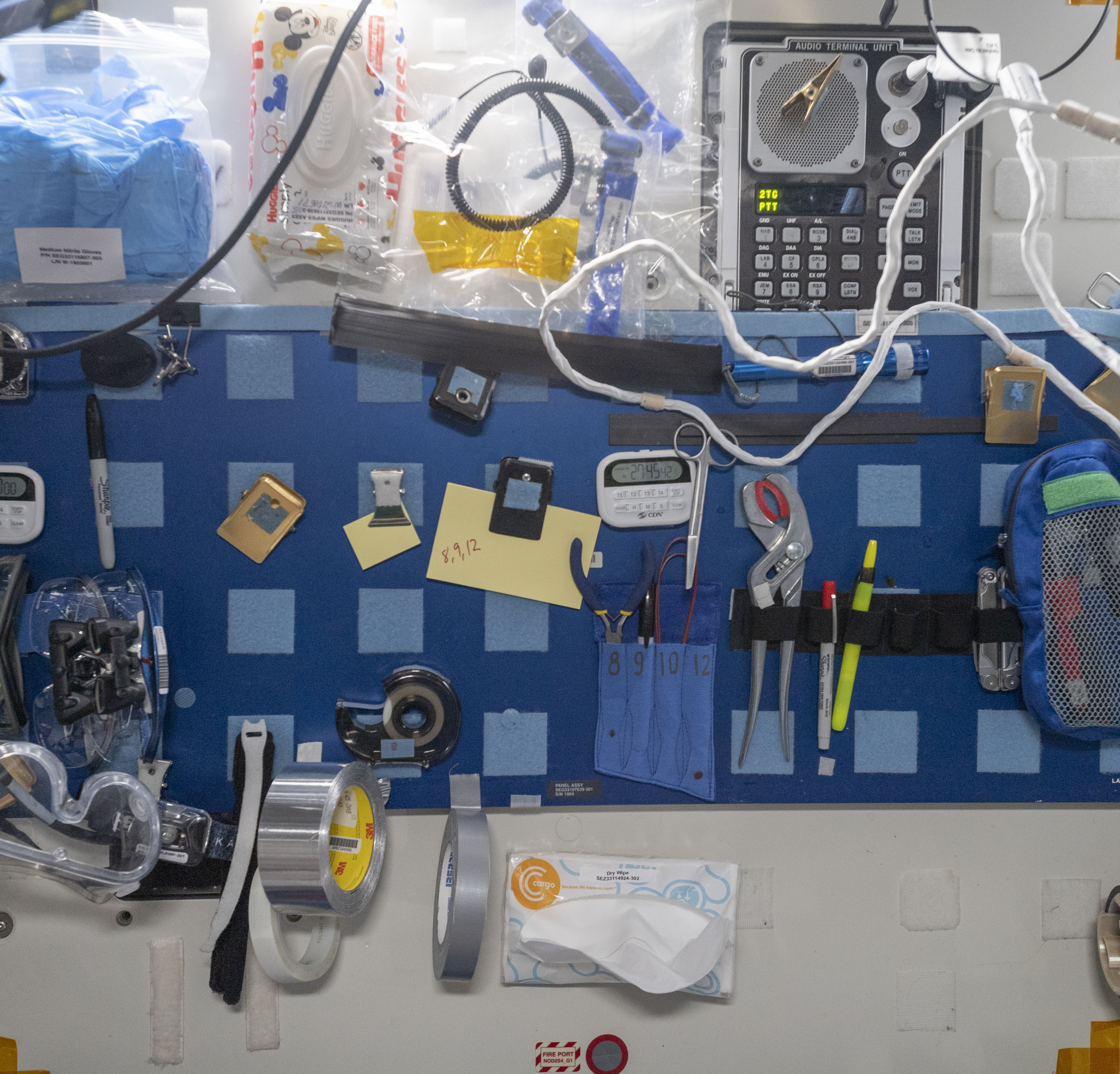

Supplement: S1 Dataset — (ZIP) [file pone.0304229.s002.zip › S03 - 40 - iss066e156056.jpg]

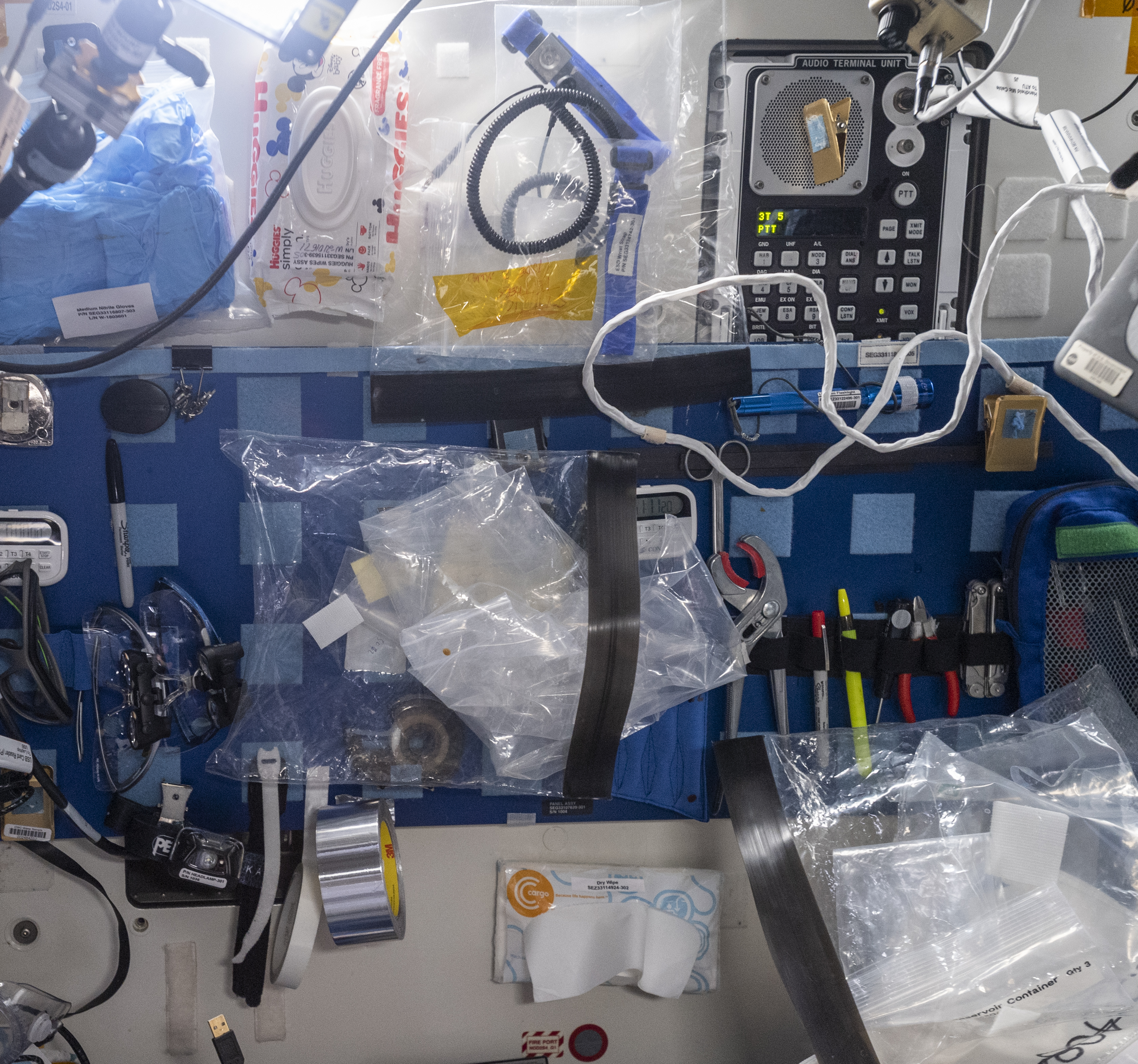

Supplement: S1 Dataset — (ZIP) [file pone.0304229.s002.zip › S03 - 41 - iss066e156453.jpg]

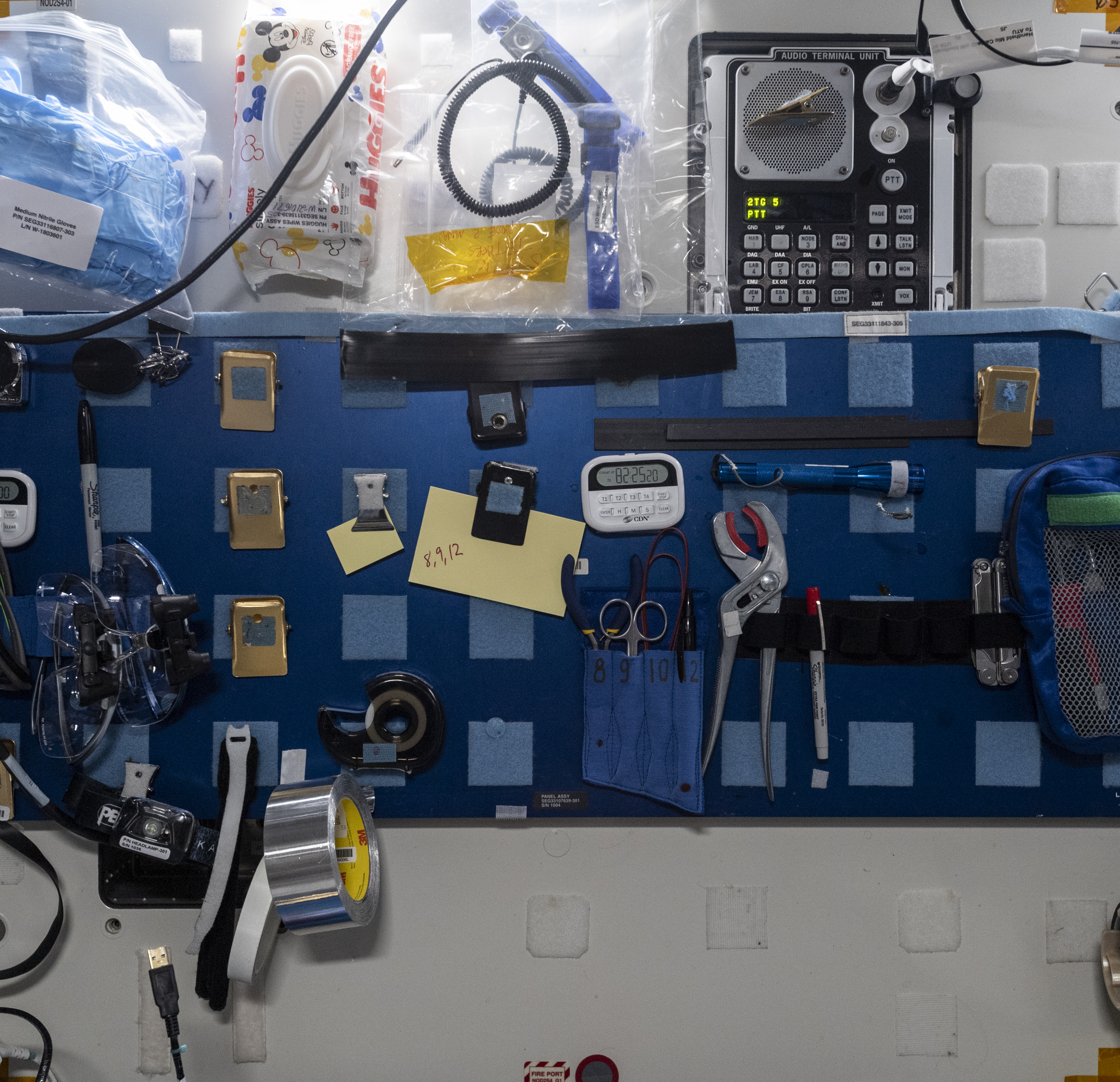

Supplement: S1 Dataset — (ZIP) [file pone.0304229.s002.zip › S03 - 42 - iss066e157052.jpg]

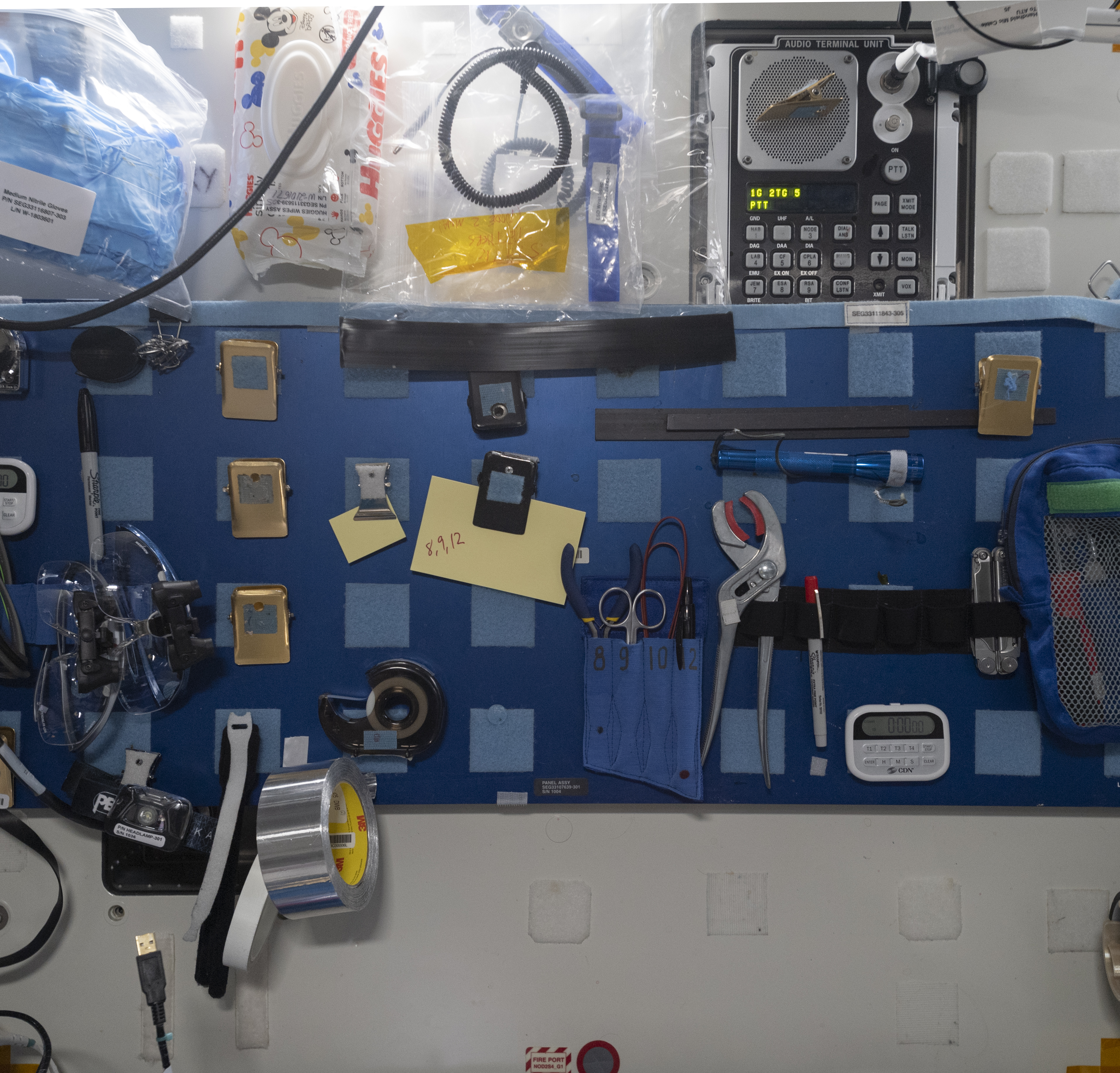

Supplement: S1 Dataset — (ZIP) [file pone.0304229.s002.zip › S03 - 43 - iss066e157140.jpg]

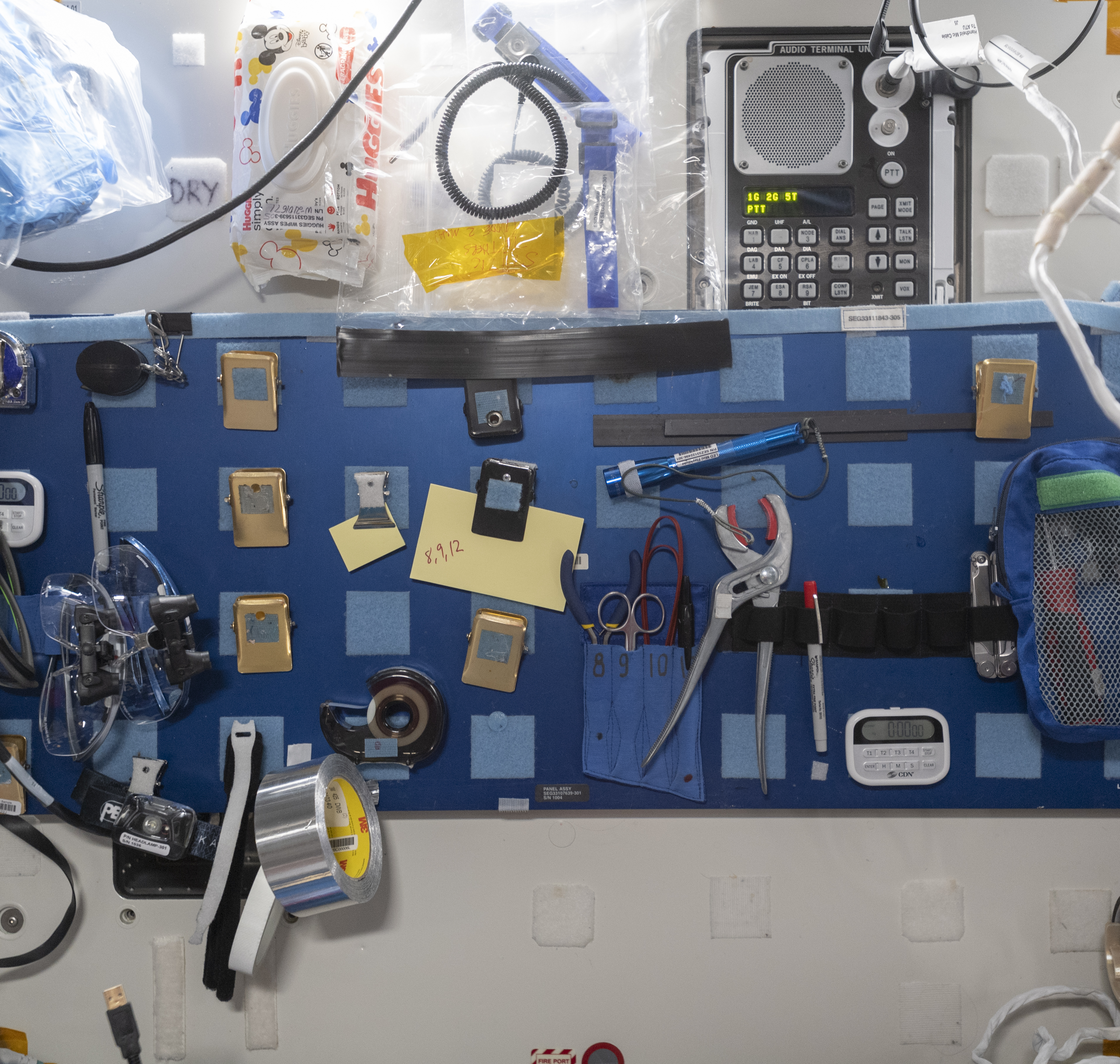

Supplement: S1 Dataset — (ZIP) [file pone.0304229.s002.zip › S03 - 44 - iss066e157134.jpg]

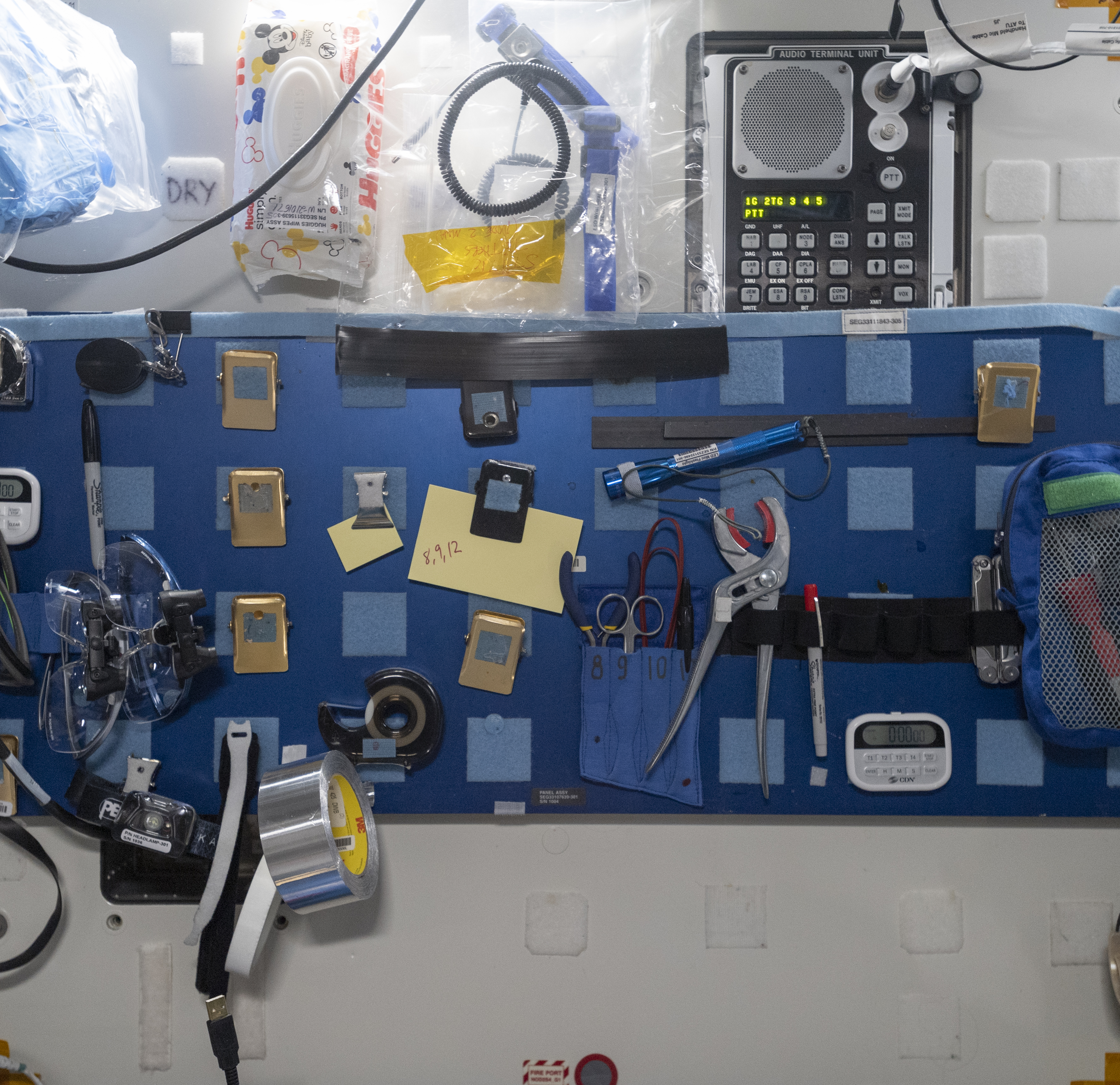

Supplement: S1 Dataset — (ZIP) [file pone.0304229.s002.zip › S03 - 45 - iss066e157598.jpg]

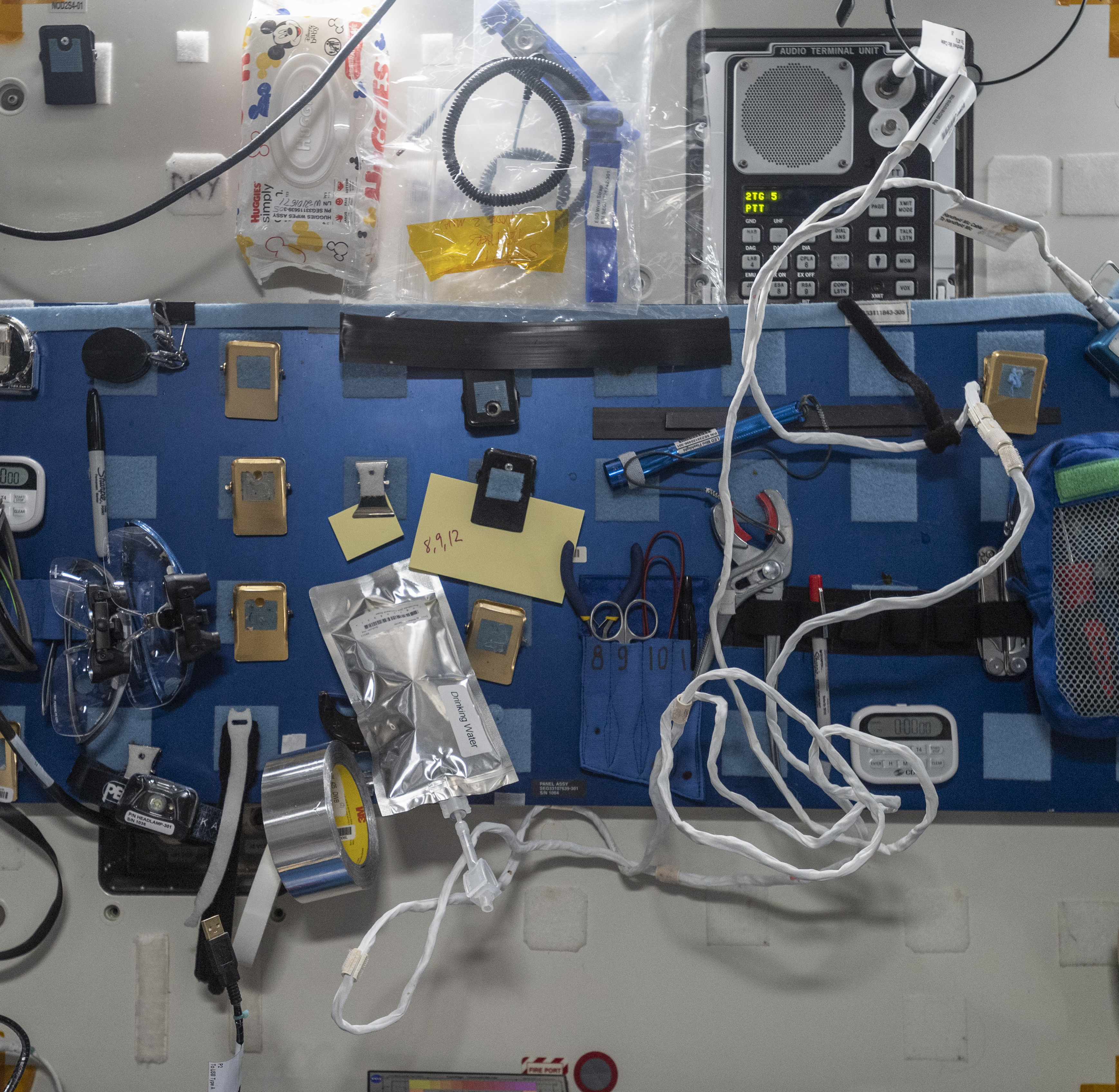

Supplement: S1 Dataset — (ZIP) [file pone.0304229.s002.zip › S03 - 46 - iss066e157917.jpg]

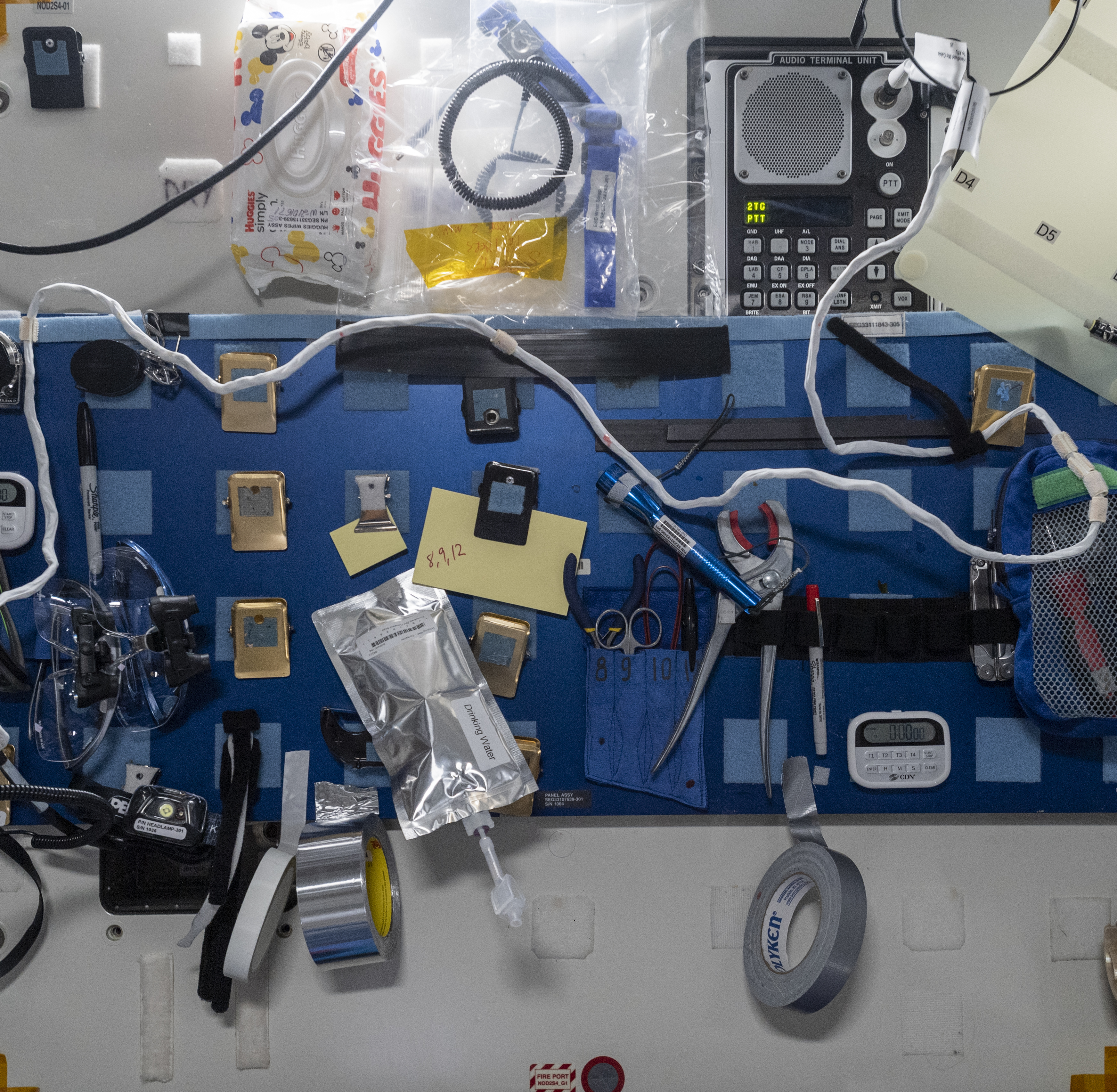

Supplement: S1 Dataset — (ZIP) [file pone.0304229.s002.zip › S03 - 47 - iss066e160709.jpg]

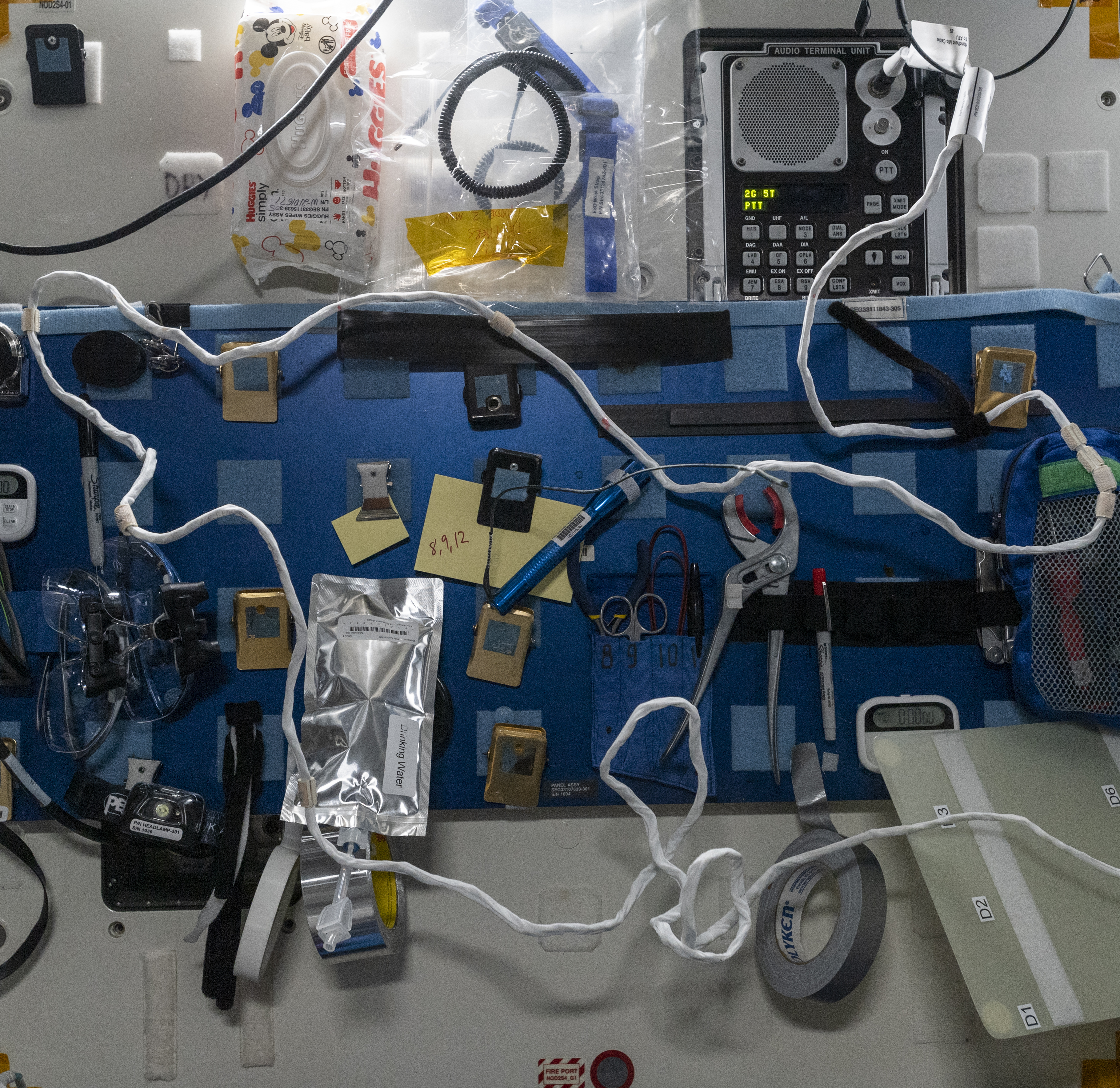

Supplement: S1 Dataset — (ZIP) [file pone.0304229.s002.zip › S03 - 48 - iss066e160939.jpg]

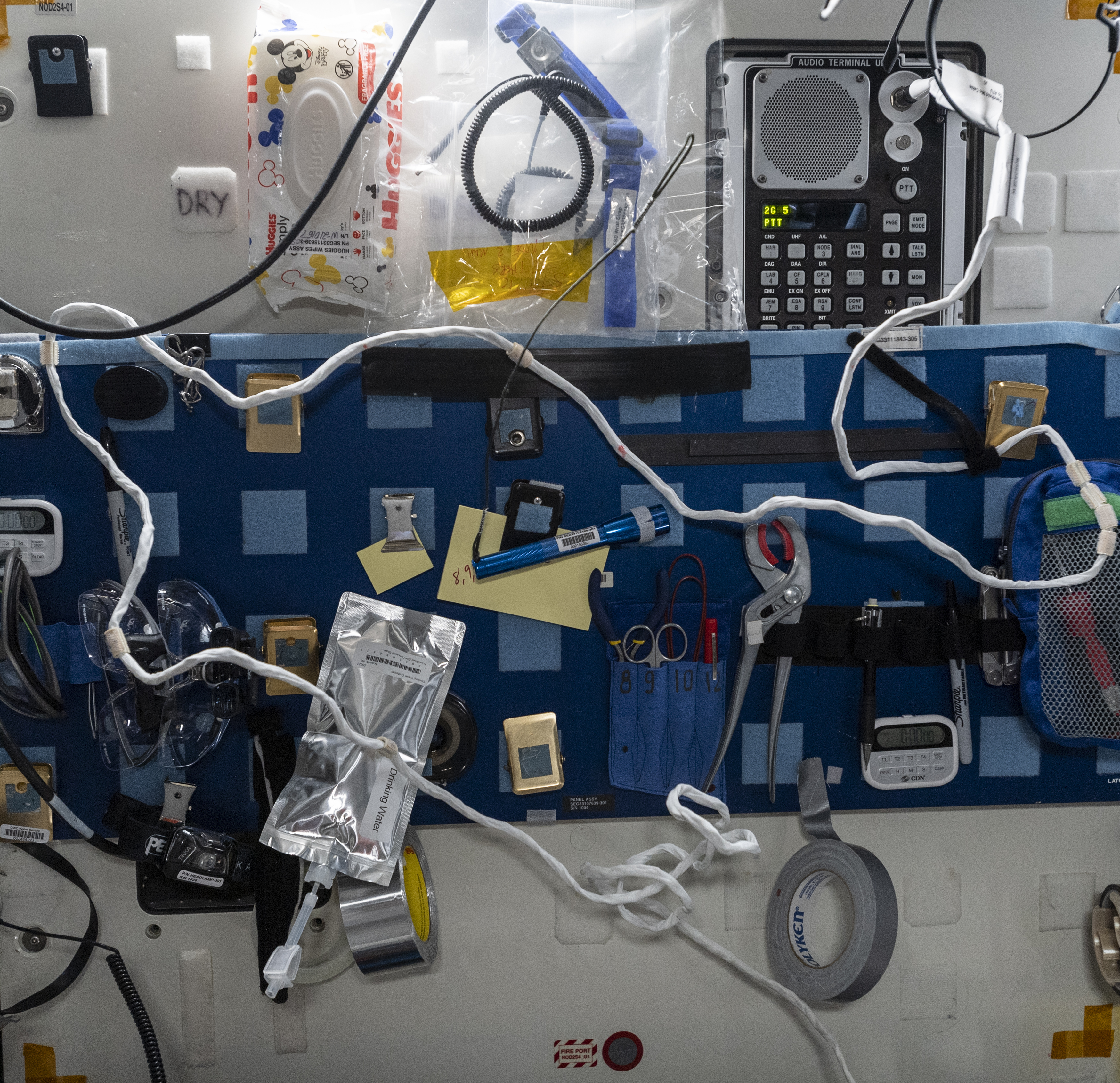

Supplement: S1 Dataset — (ZIP) [file pone.0304229.s002.zip › S03 - 49 - iss066e161298.jpg]

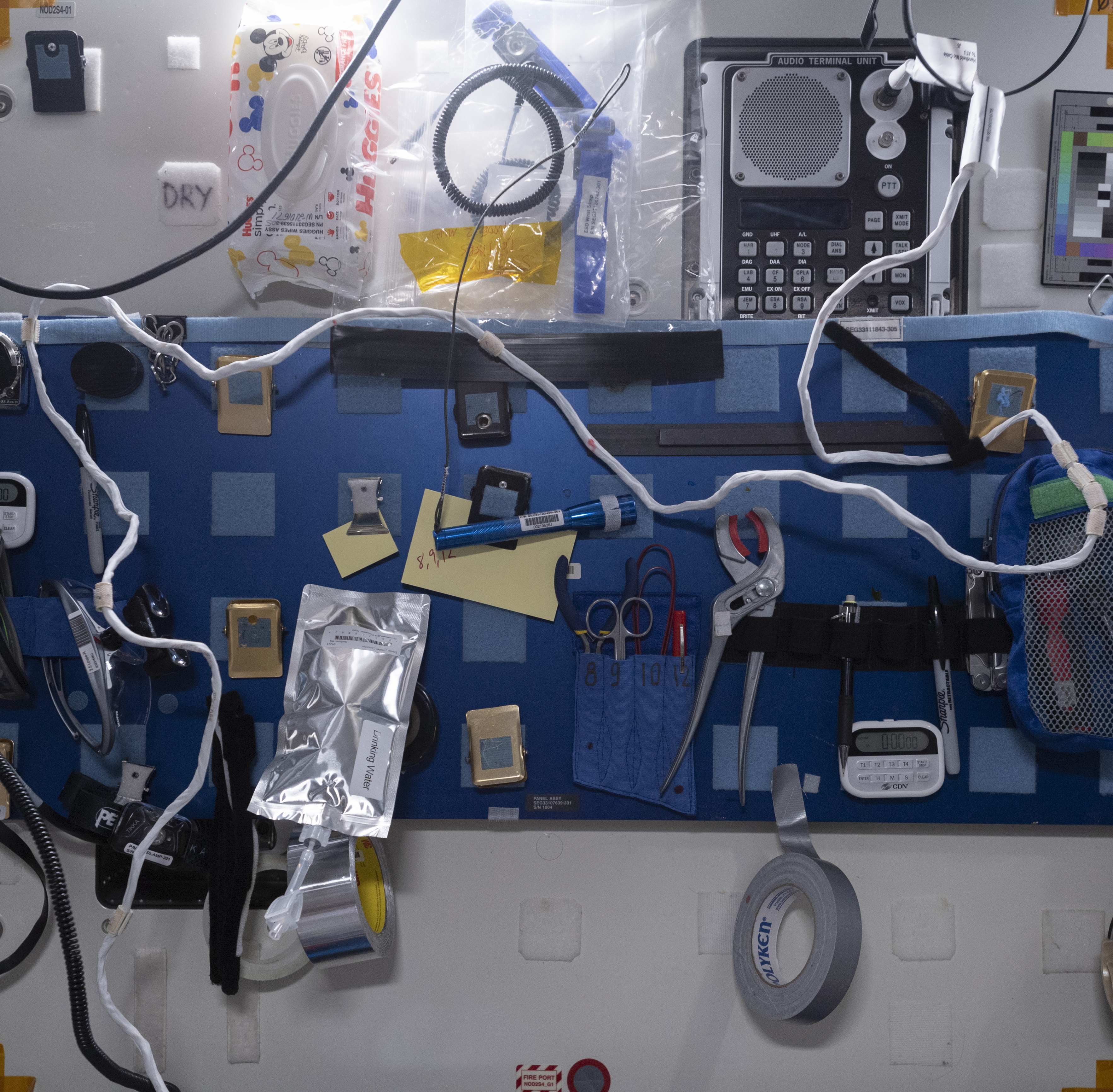

Supplement: S1 Dataset — (ZIP) [file pone.0304229.s002.zip › S03 - 50 - iss066e161495.jpg]

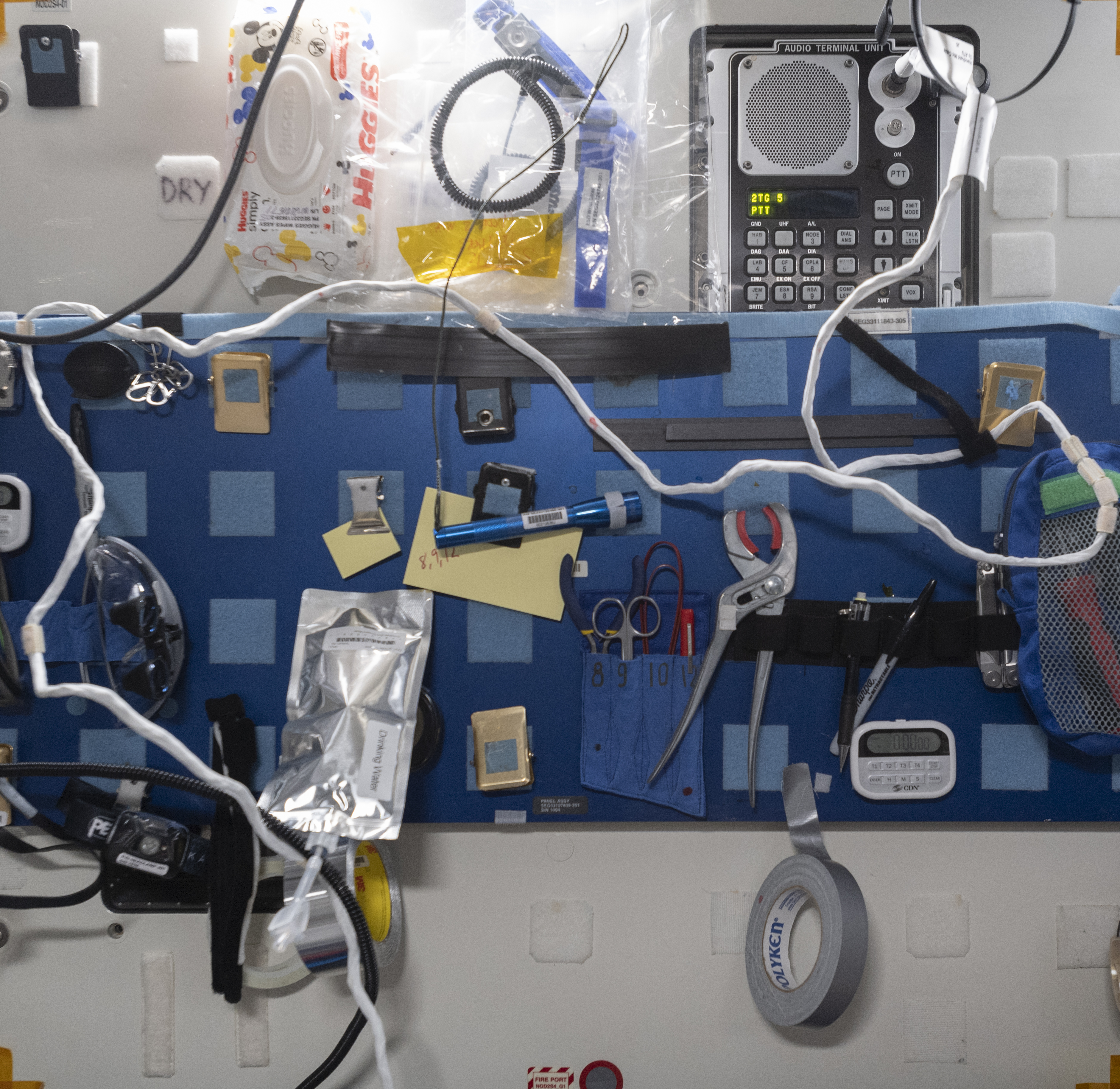

Supplement: S1 Dataset — (ZIP) [file pone.0304229.s002.zip › S03 - 51 - iss066e161488.jpg]

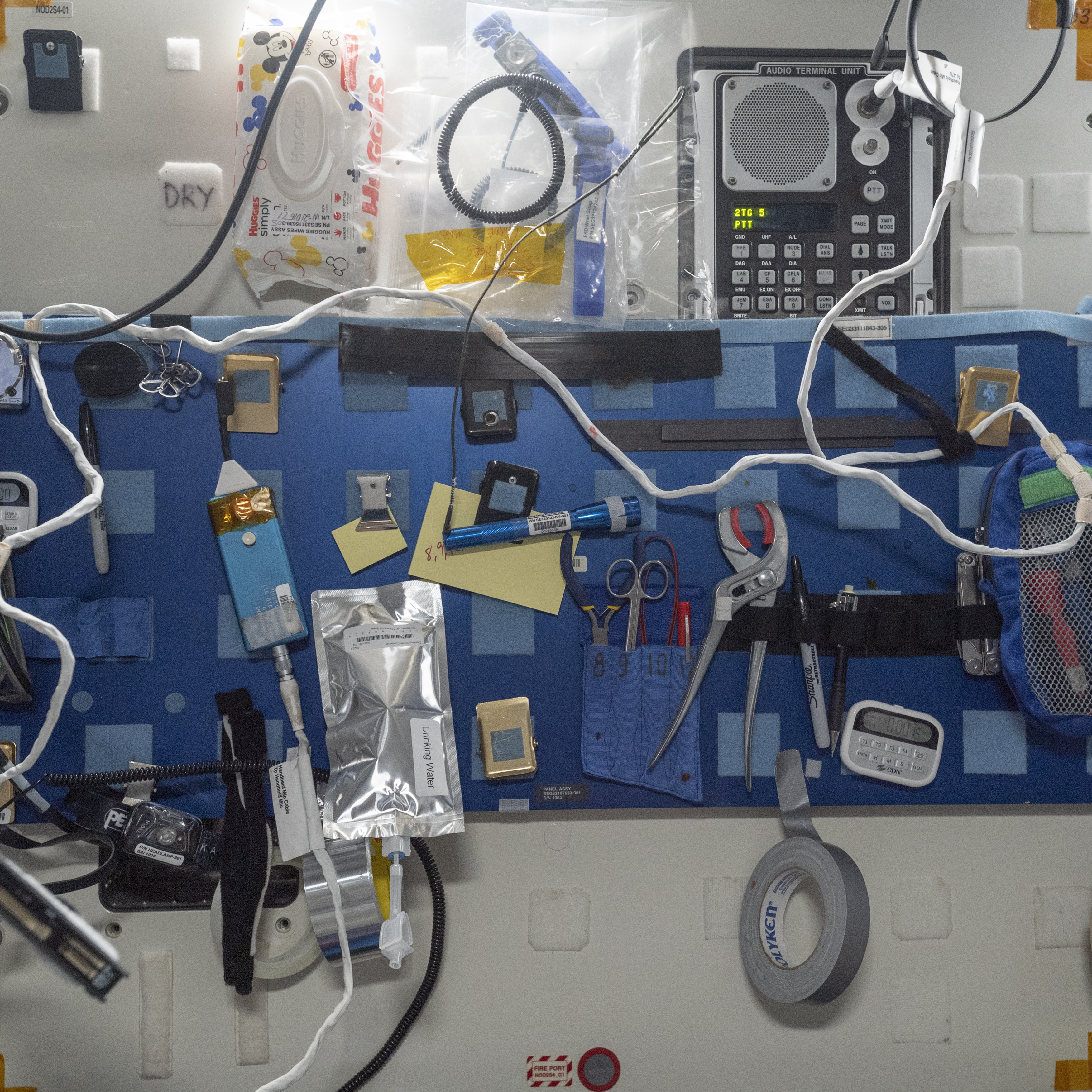

Supplement: S1 Dataset — (ZIP) [file pone.0304229.s002.zip › S03 - 52 - iss066e162127.jpg]

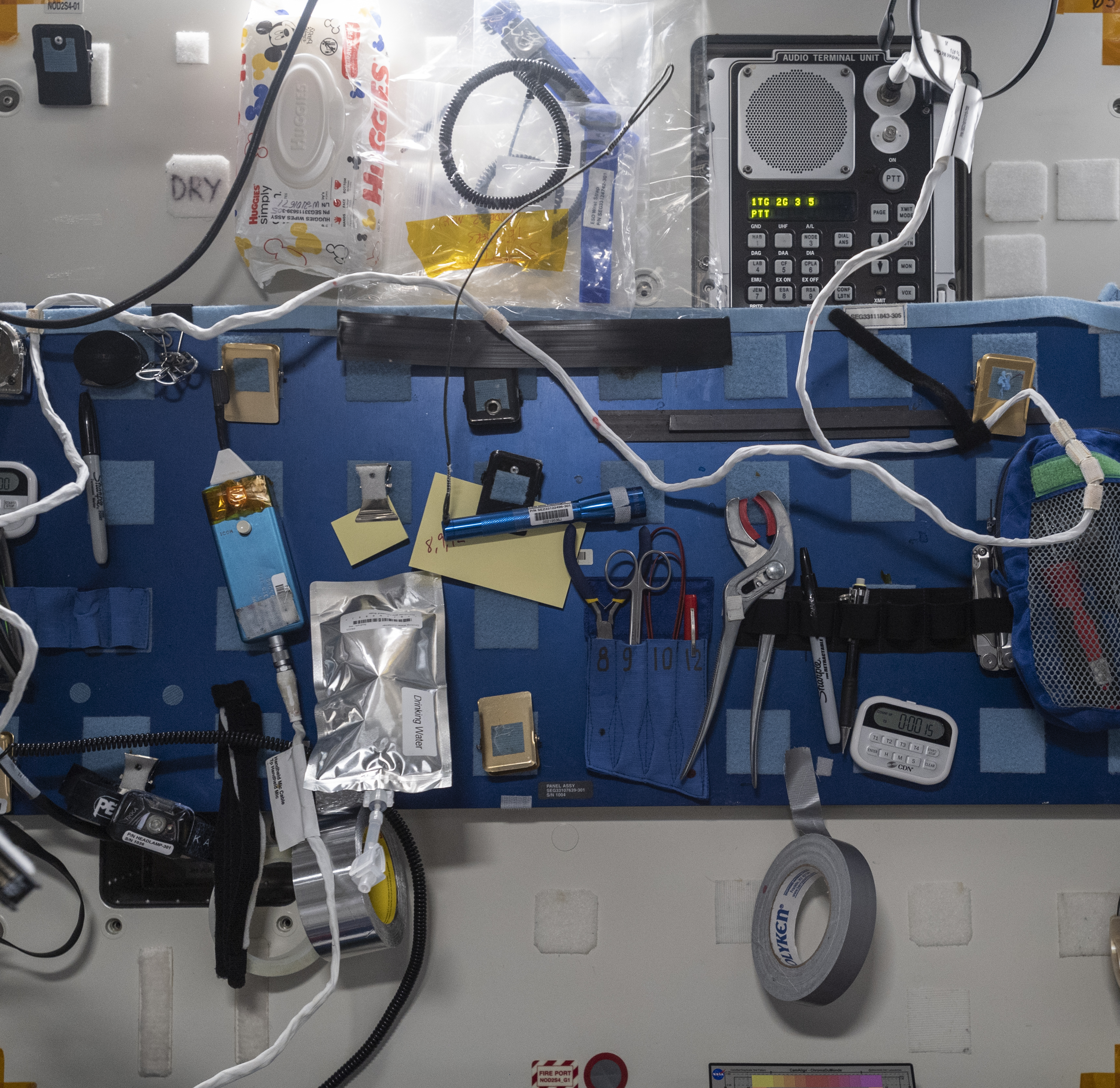

Supplement: S1 Dataset — (ZIP) [file pone.0304229.s002.zip › S03 - 53 - iss066e165111.jpg]

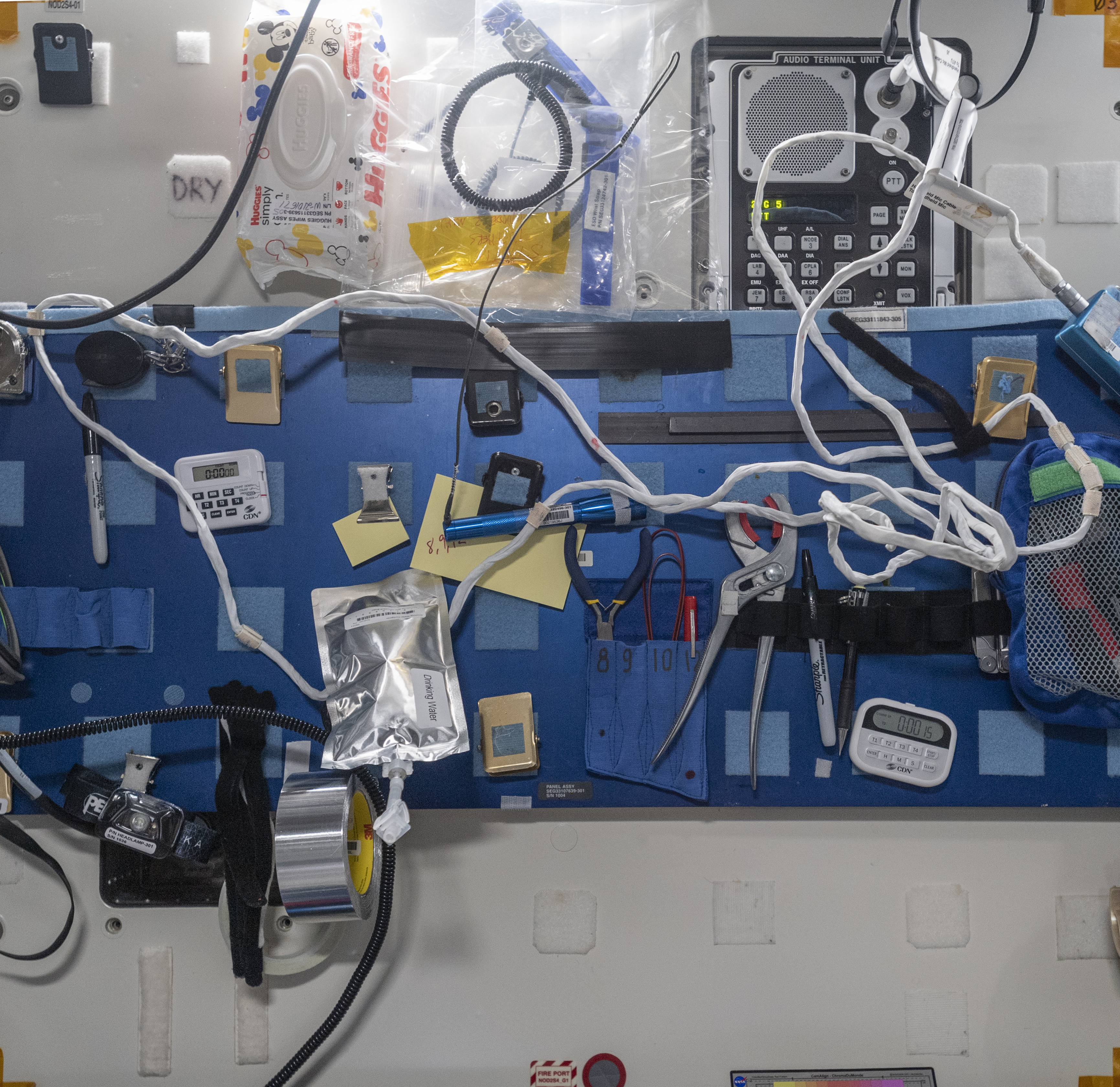

Supplement: S1 Dataset — (ZIP) [file pone.0304229.s002.zip › S03 - 54 - iss066e167403.jpg]

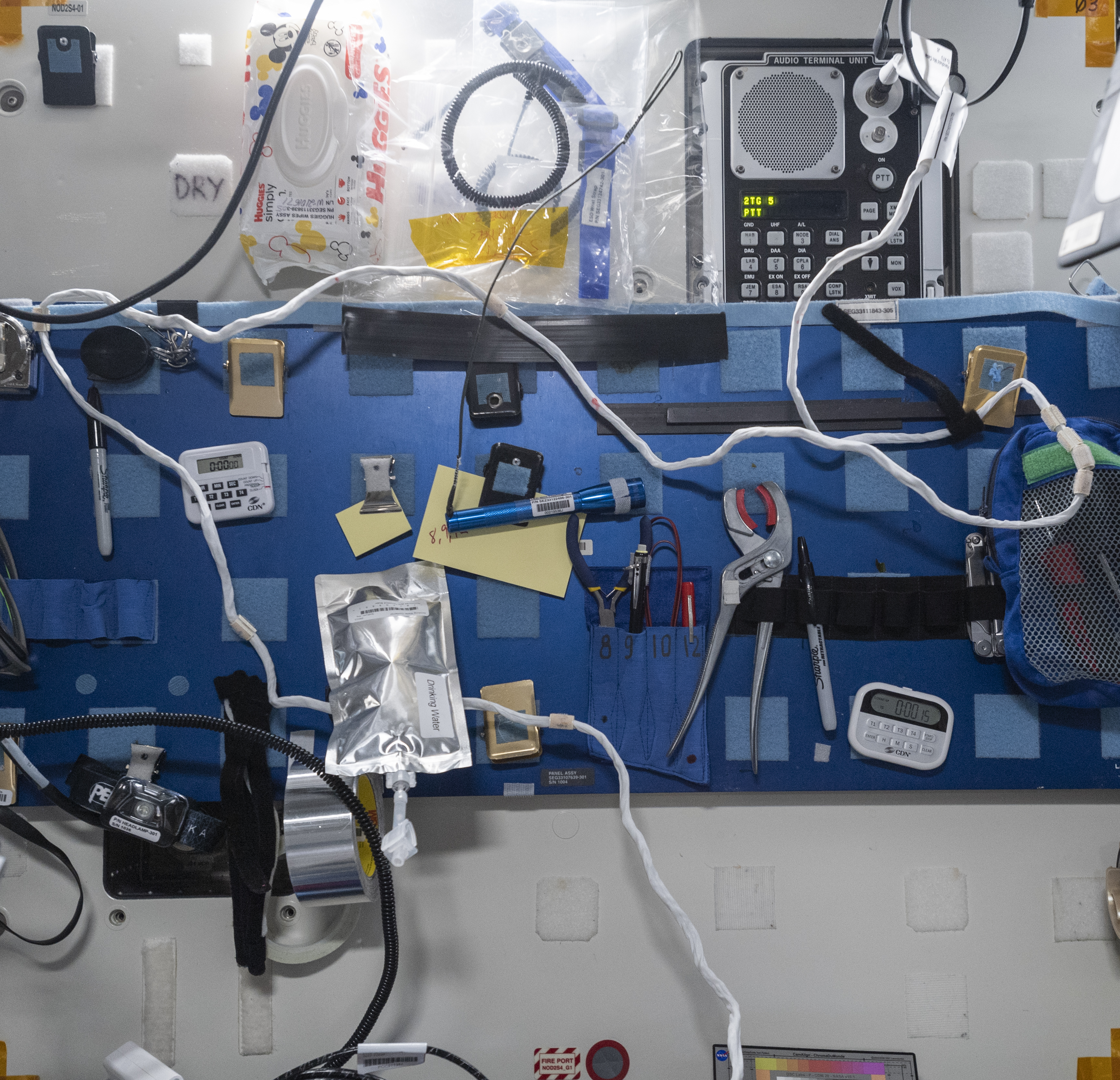

Supplement: S1 Dataset — (ZIP) [file pone.0304229.s002.zip › S03 - 55 - iss066e172257.jpg]

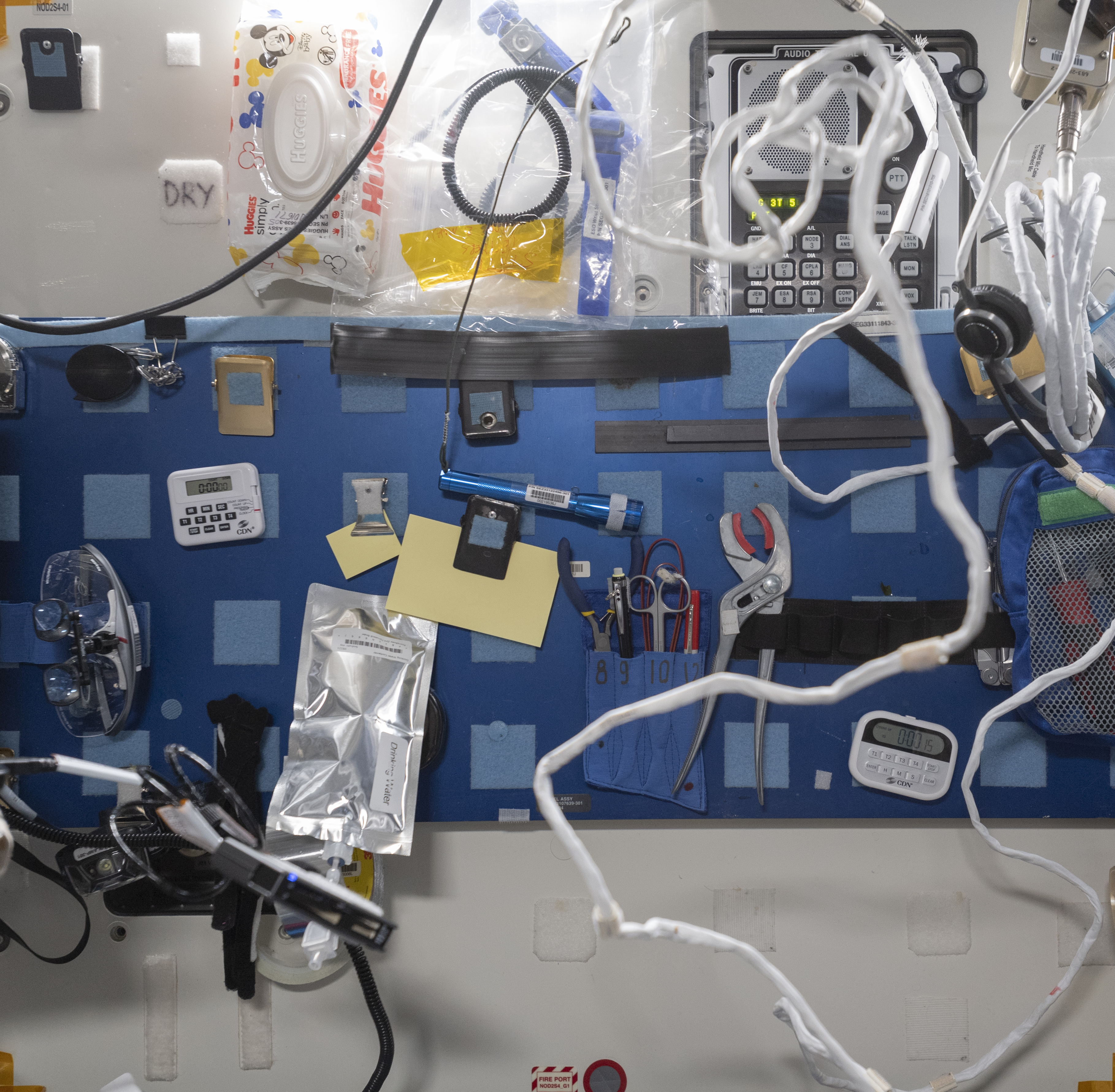

Supplement: S1 Dataset — (ZIP) [file pone.0304229.s002.zip › S03 - 56 - iss066e173220.jpg]

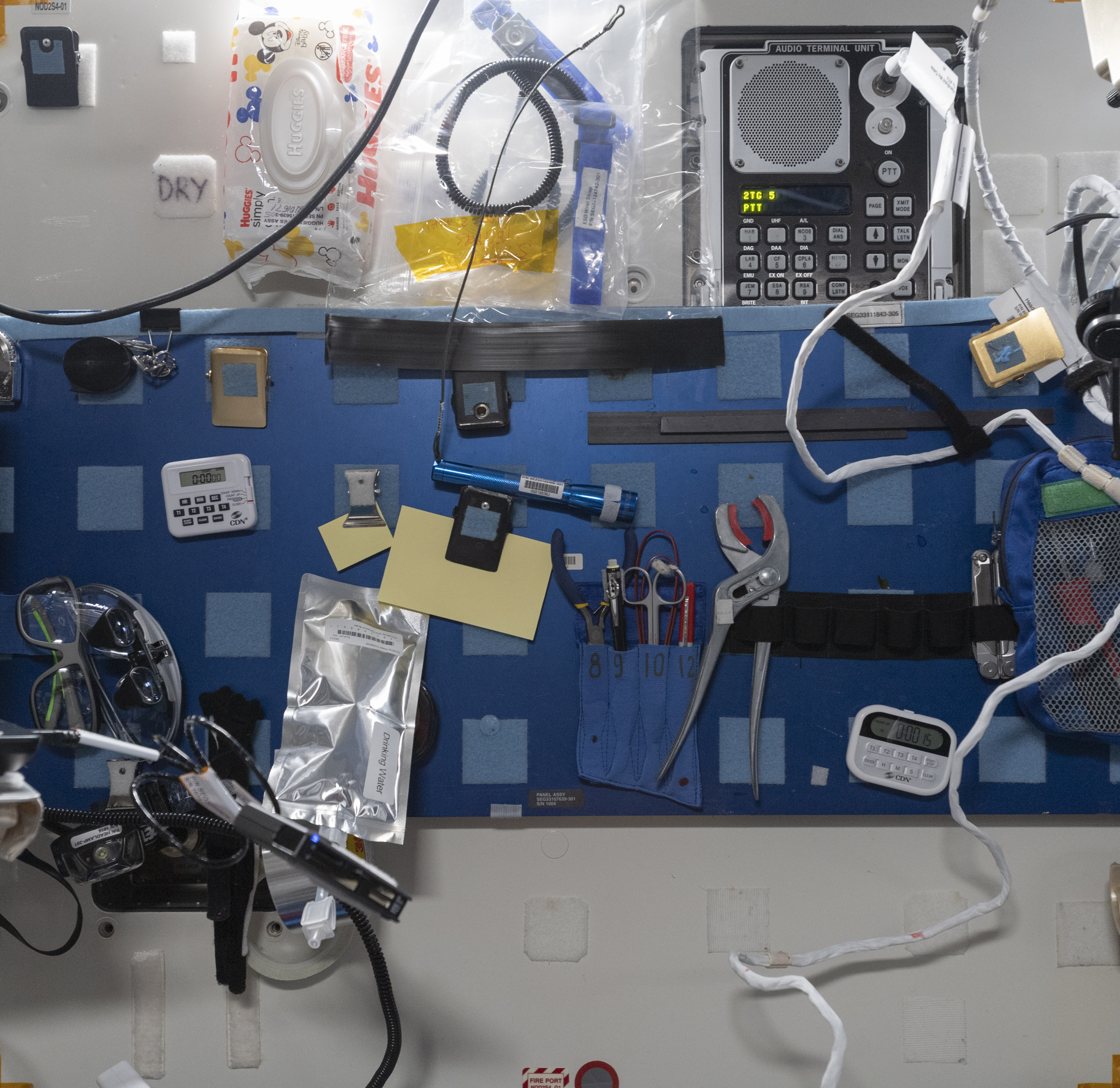

Supplement: S1 Dataset — (ZIP) [file pone.0304229.s002.zip › S03 - 57 - iss066e173216.jpg]

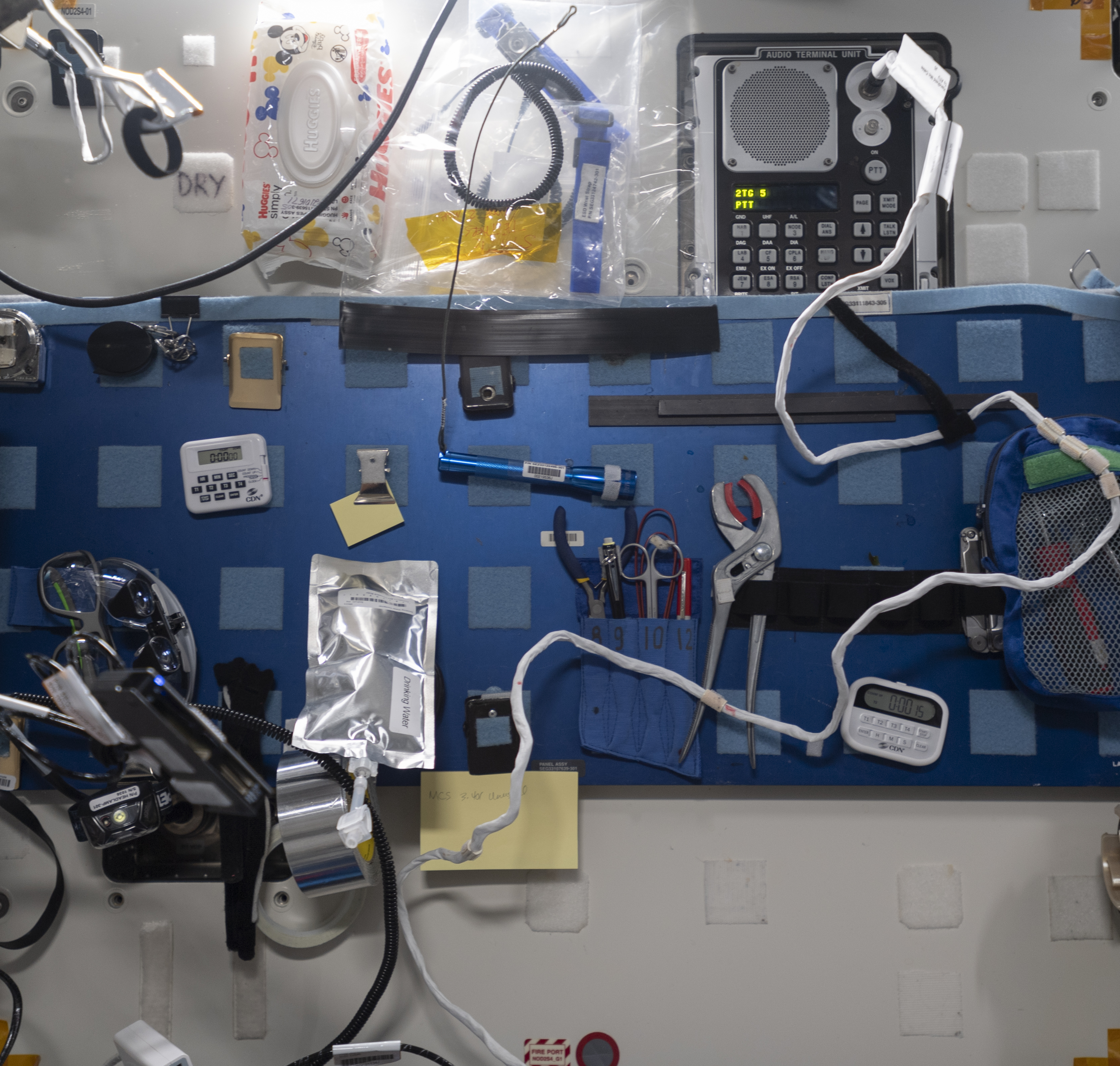

Supplement: S1 Dataset — (ZIP) [file pone.0304229.s002.zip › S03 - 58 - iss066e173124.jpg]

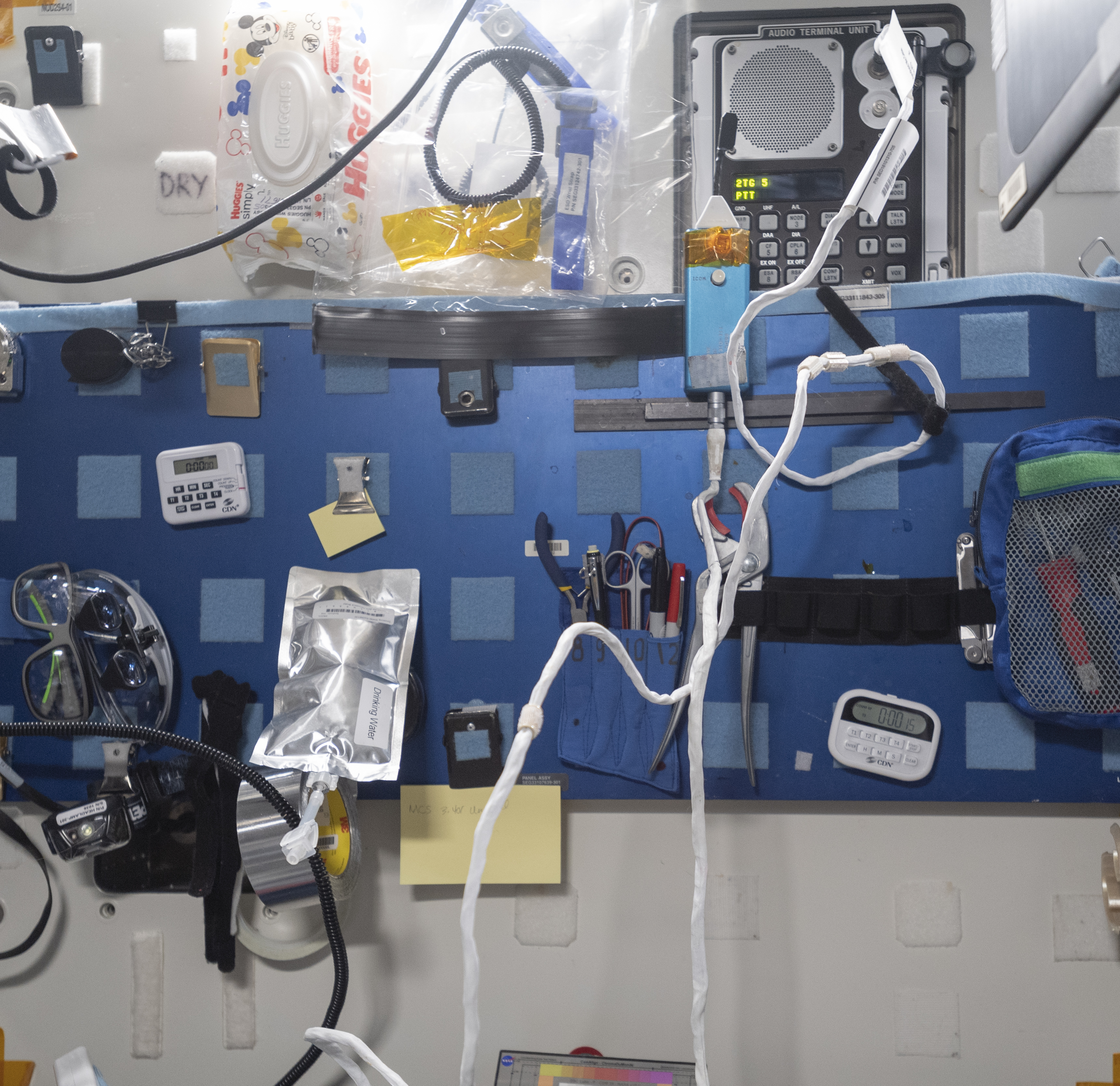

Supplement: S1 Dataset — (ZIP) [file pone.0304229.s002.zip › S03 - 59 - iss066e173142.jpg]

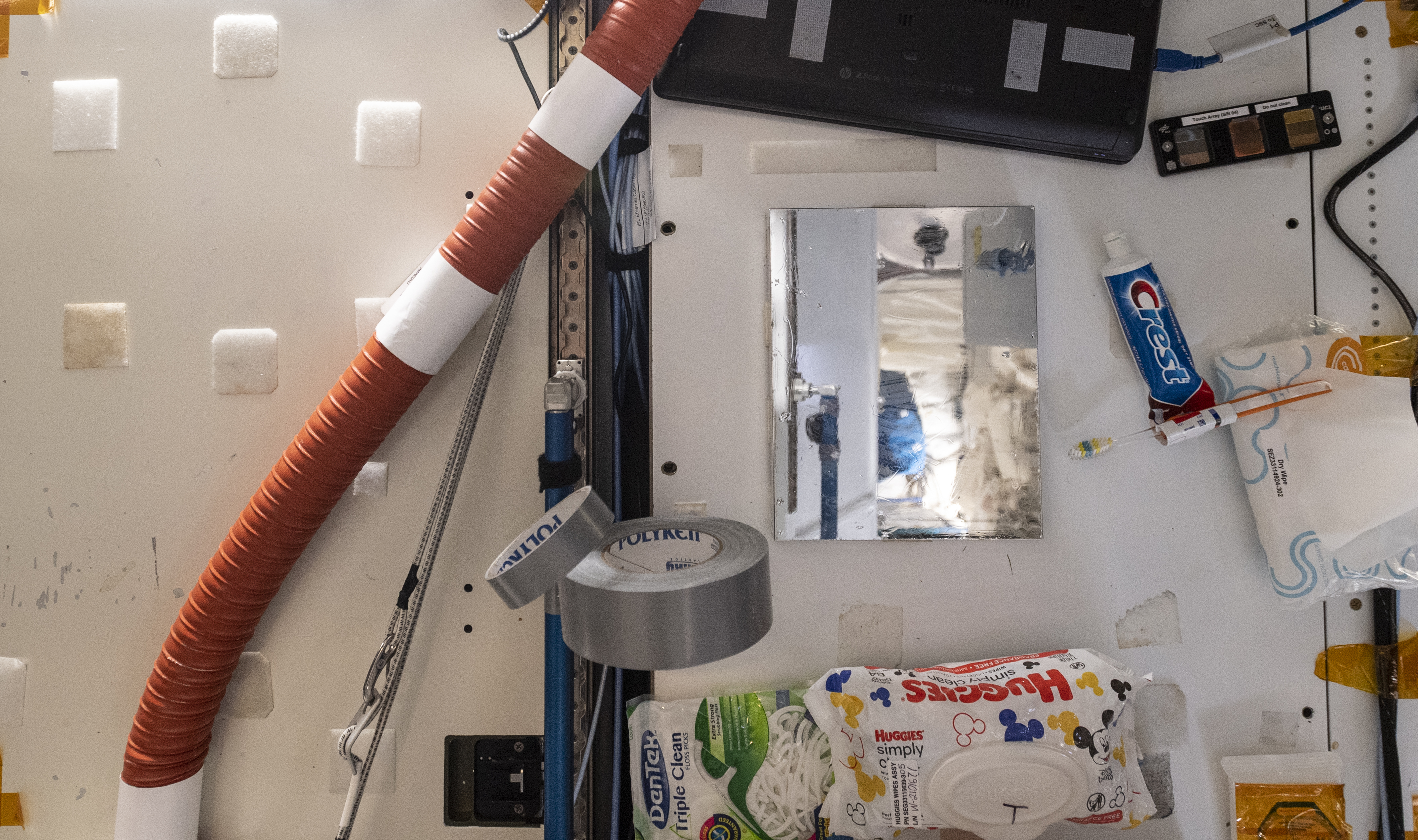

Supplement: S2 Dataset — (ZIP) [file pone.0304229.s003.zip › S05 - 00 - iss066e123436-2crop.jpg]

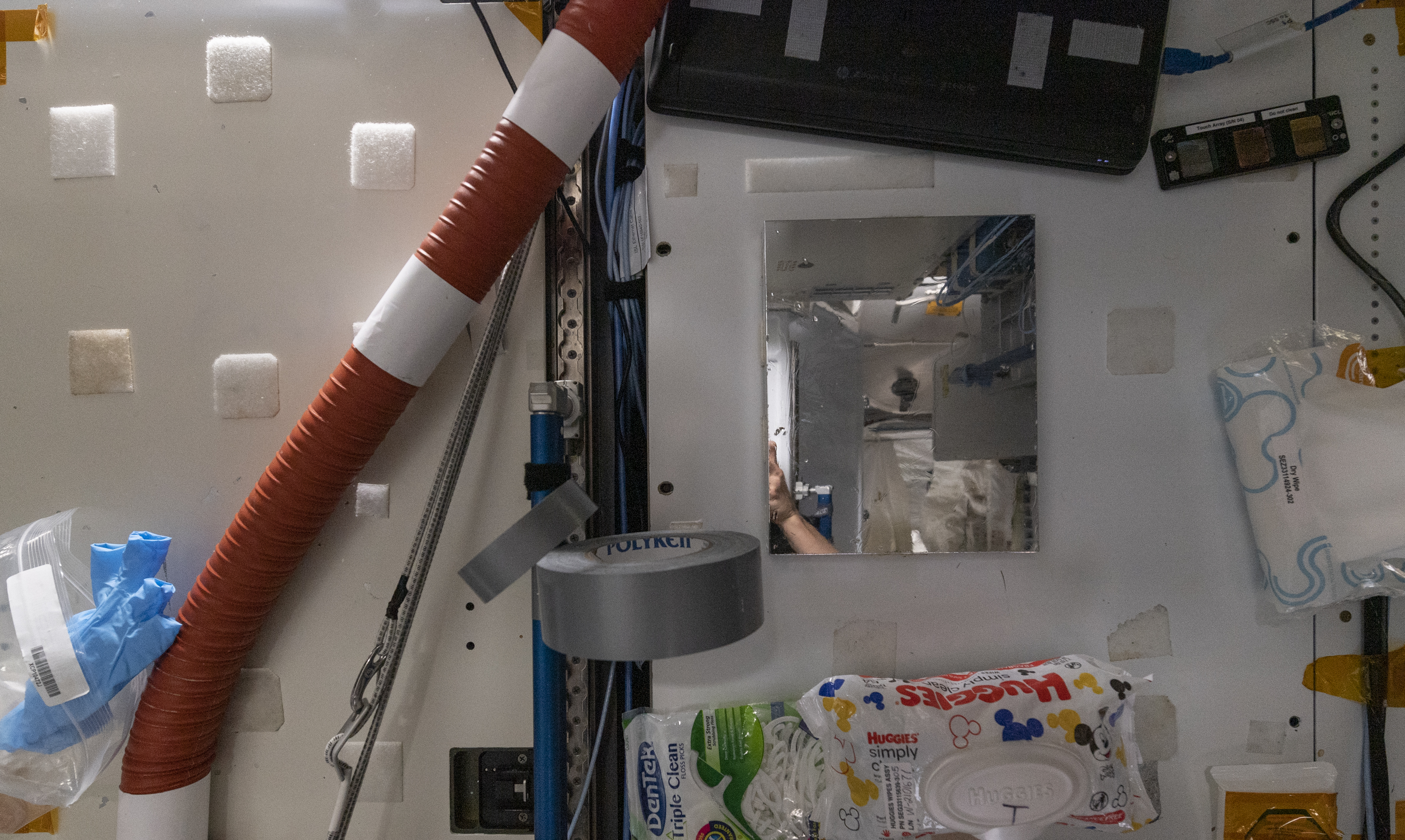

Supplement: S2 Dataset — (ZIP) [file pone.0304229.s003.zip › S05 - 01 - iss066e124440crop.jpg]

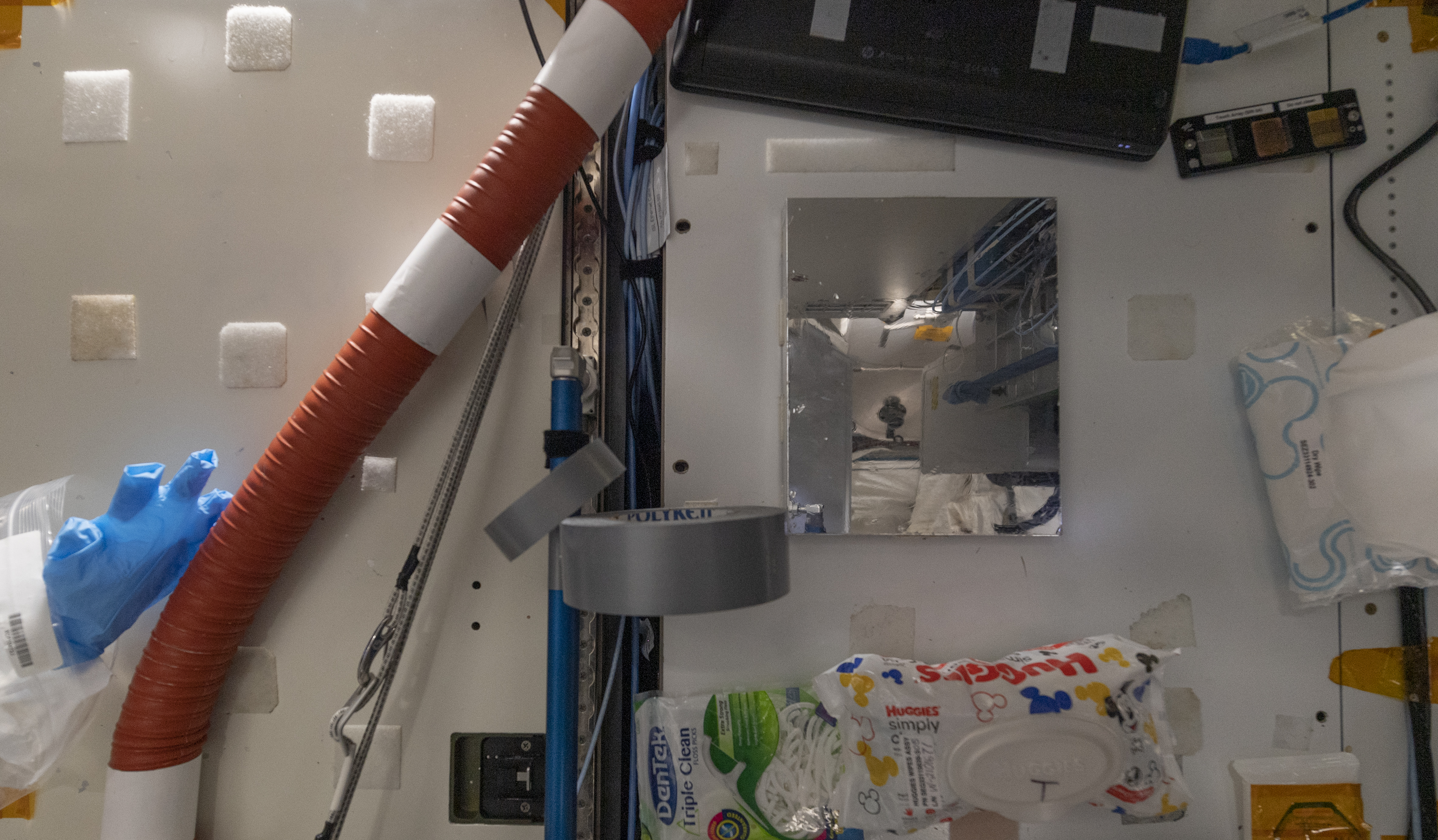

Supplement: S2 Dataset — (ZIP) [file pone.0304229.s003.zip › S05 - 02 - iss066e124441.jpg]

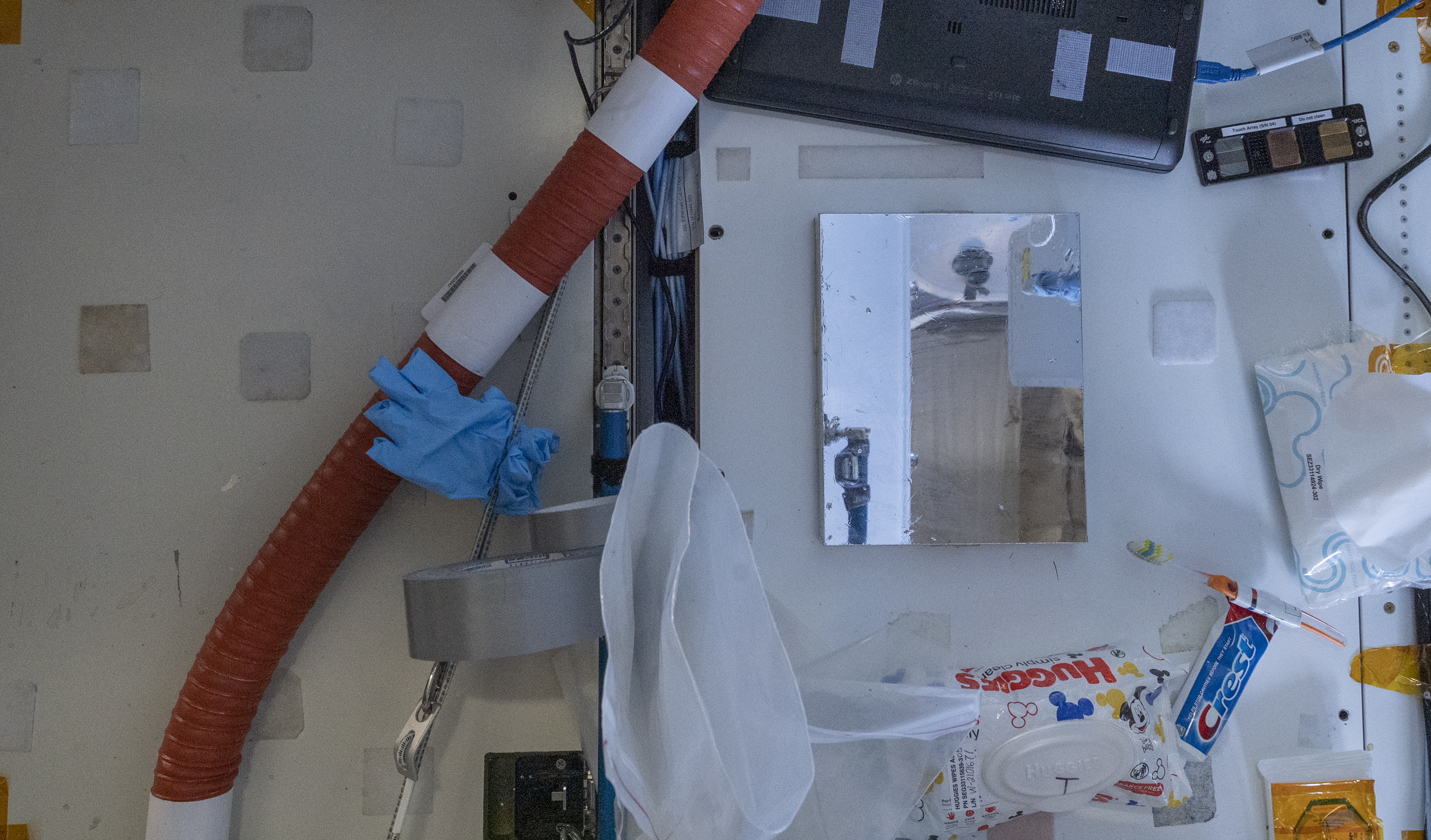

Supplement: S2 Dataset — (ZIP) [file pone.0304229.s003.zip › S05 - 03 - iss066e124608.jpg]

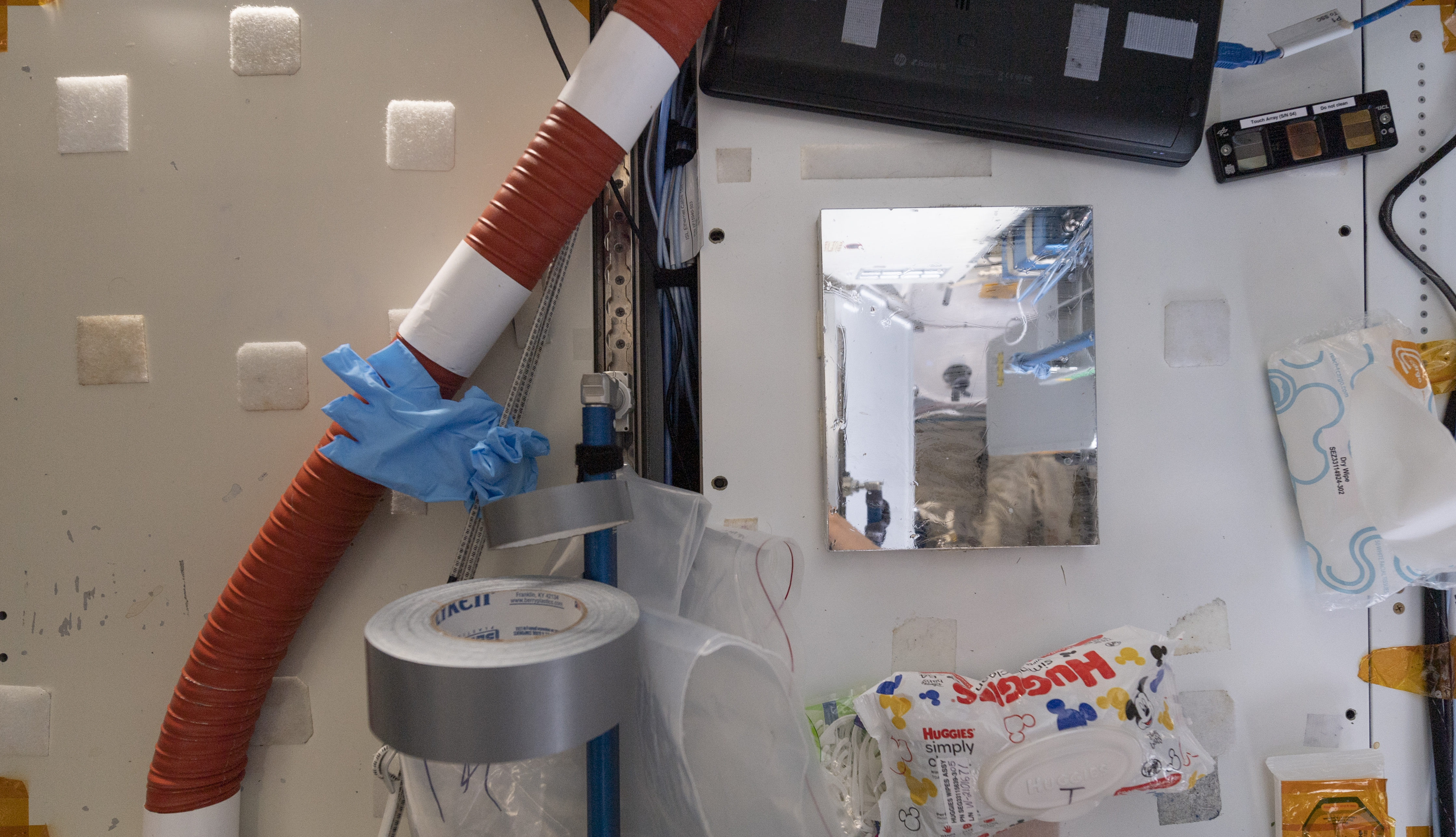

Supplement: S2 Dataset — (ZIP) [file pone.0304229.s003.zip › S05 - 04 - iss066e125378.jpg]

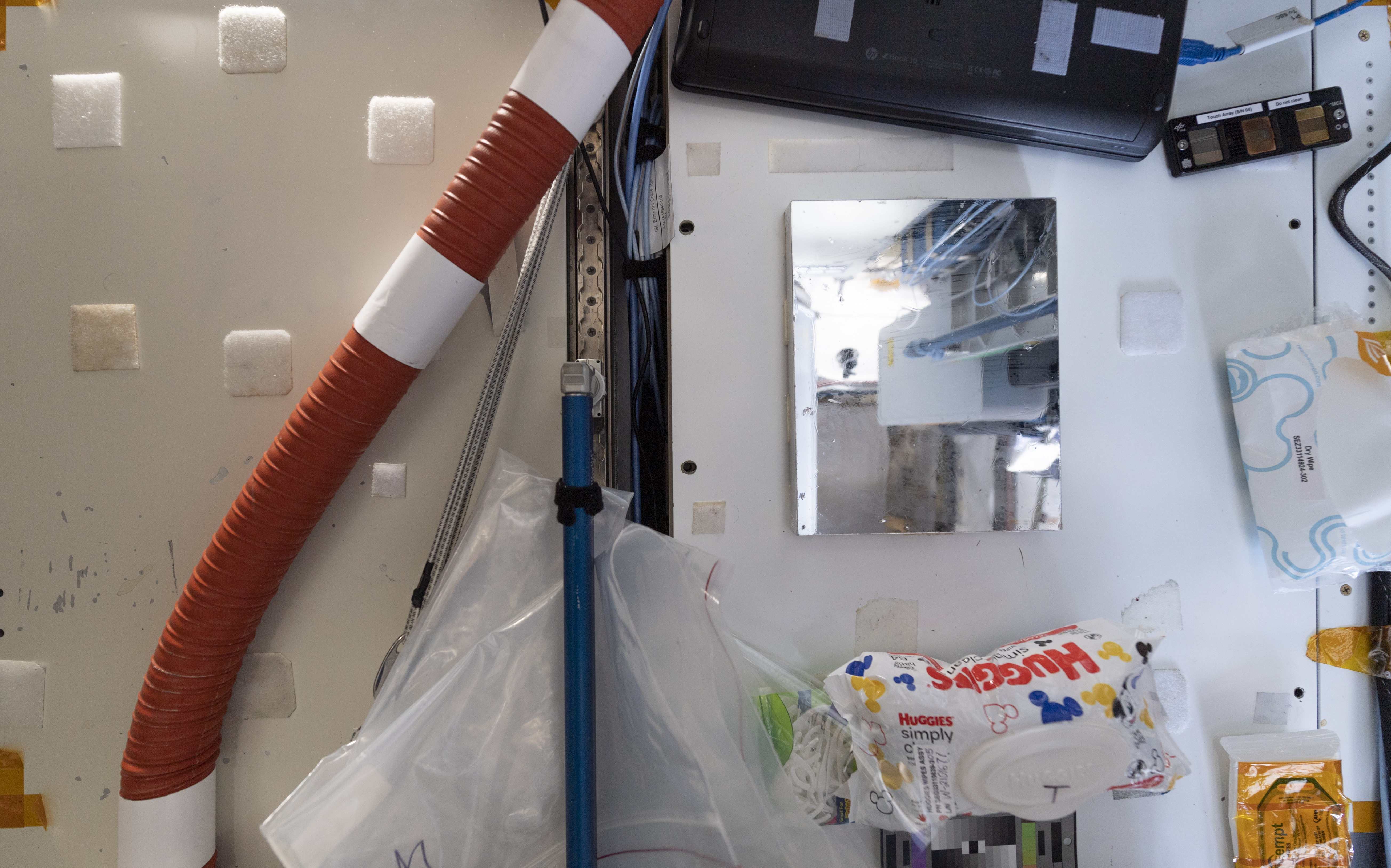

Supplement: S2 Dataset — (ZIP) [file pone.0304229.s003.zip › S05 - 05 - iss066e126156.jpg]

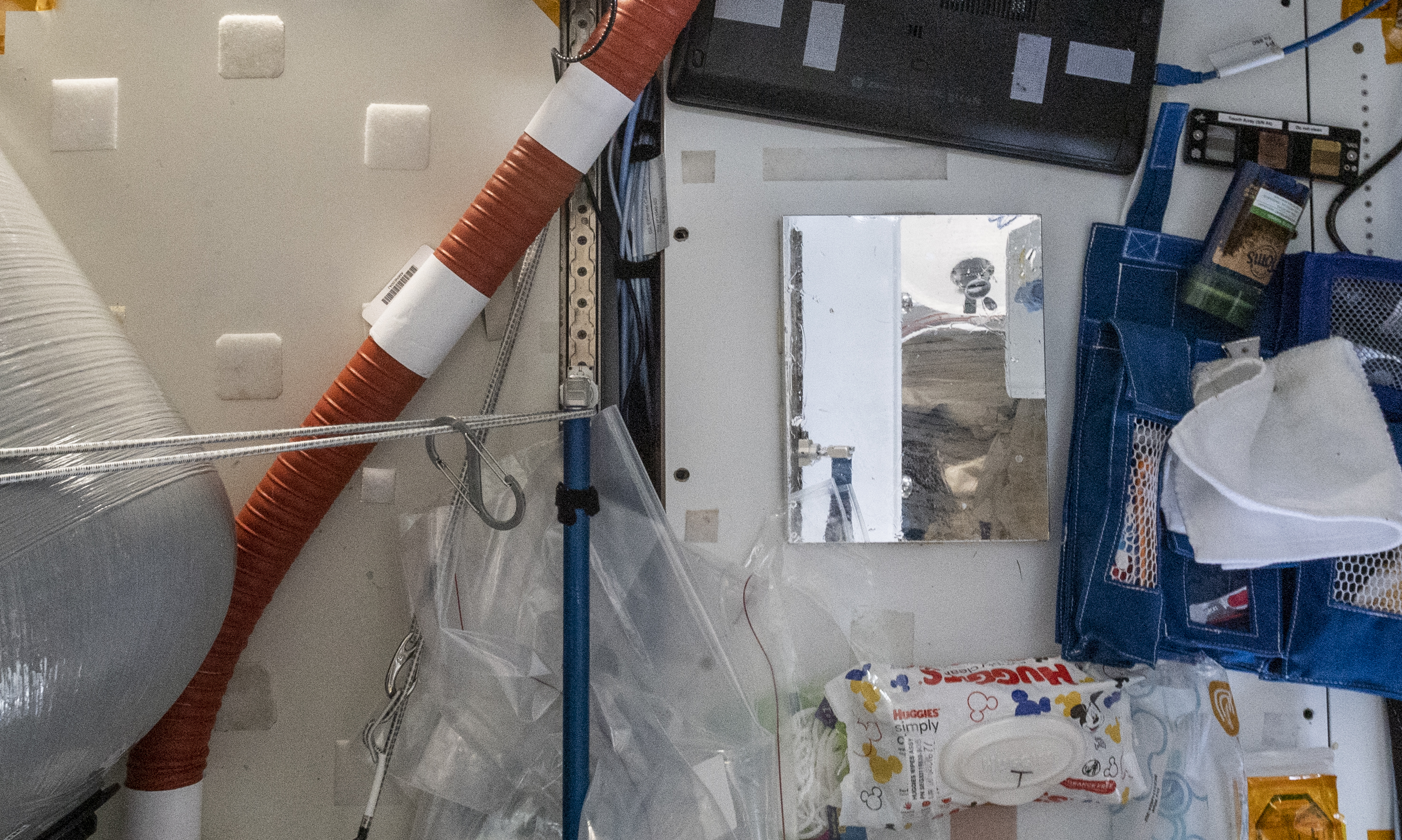

Supplement: S2 Dataset — (ZIP) [file pone.0304229.s003.zip › S05 - 06 - iss066e129537.jpg]

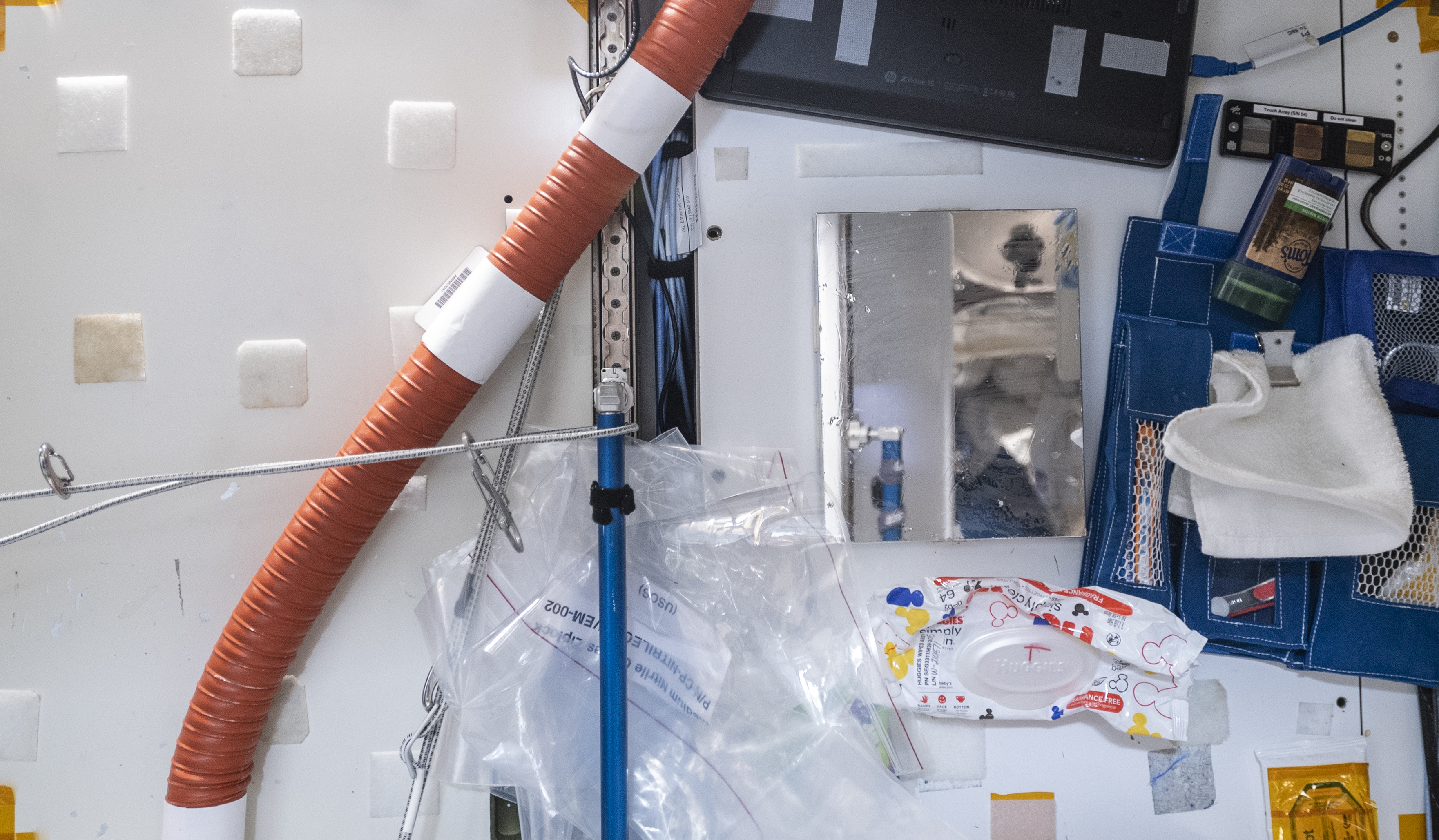

Supplement: S2 Dataset — (ZIP) [file pone.0304229.s003.zip › S05 - 07 - iss066e129764.jpg]

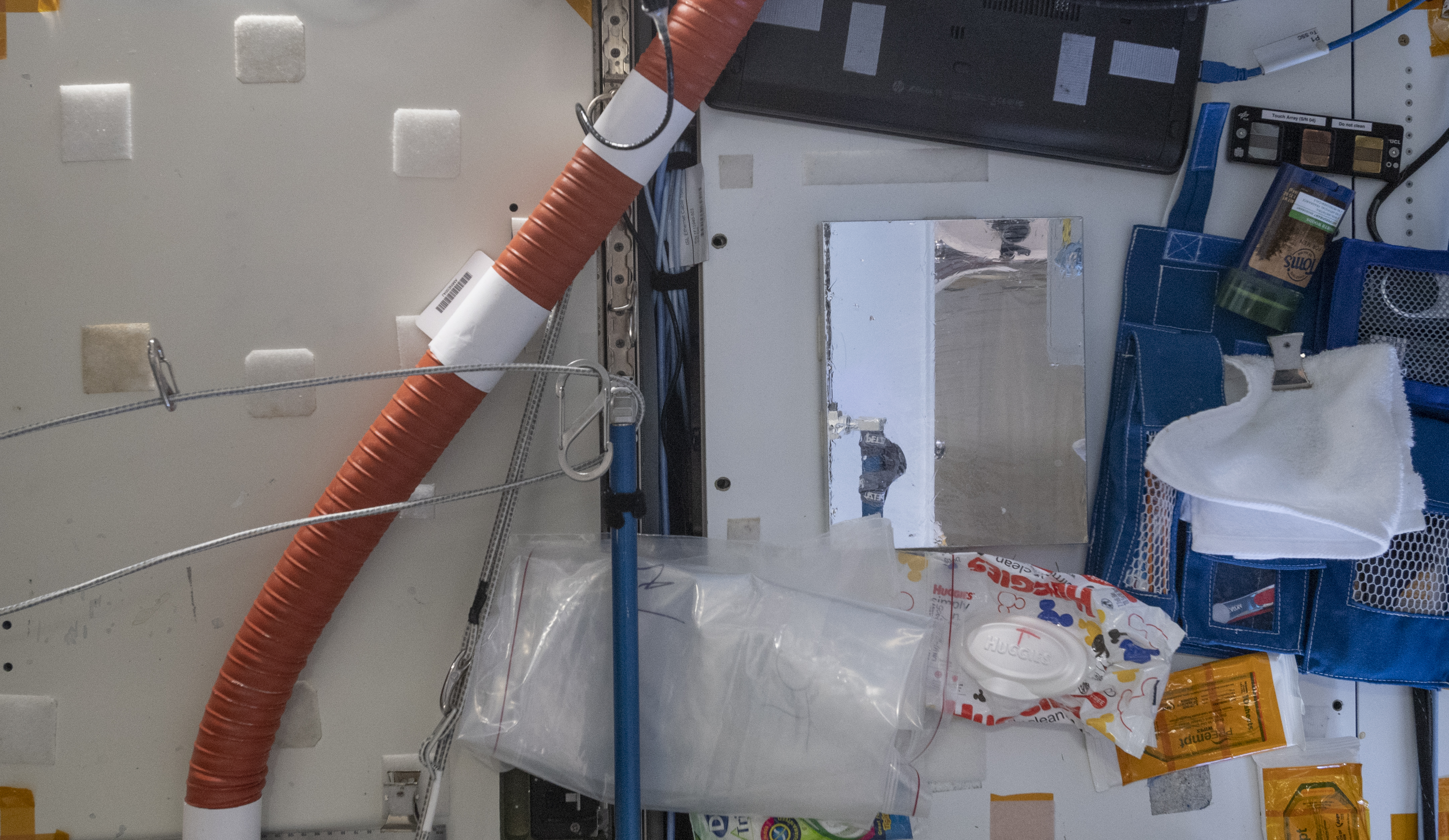

Supplement: S2 Dataset — (ZIP) [file pone.0304229.s003.zip › S05 - 08 - iss066e130192.jpg]

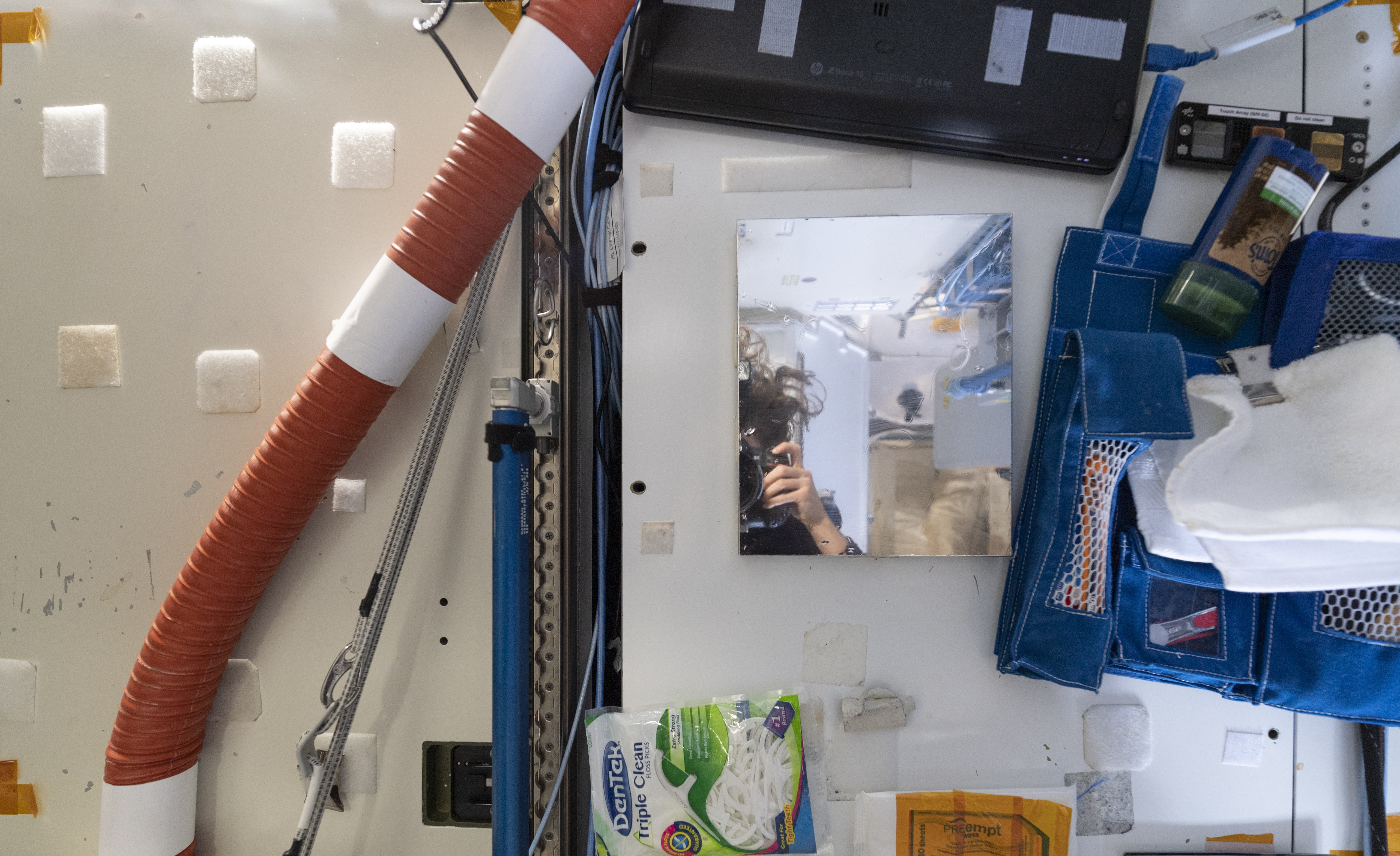

Supplement: S2 Dataset — (ZIP) [file pone.0304229.s003.zip › S05 - 09 - iss066e130200.jpg]

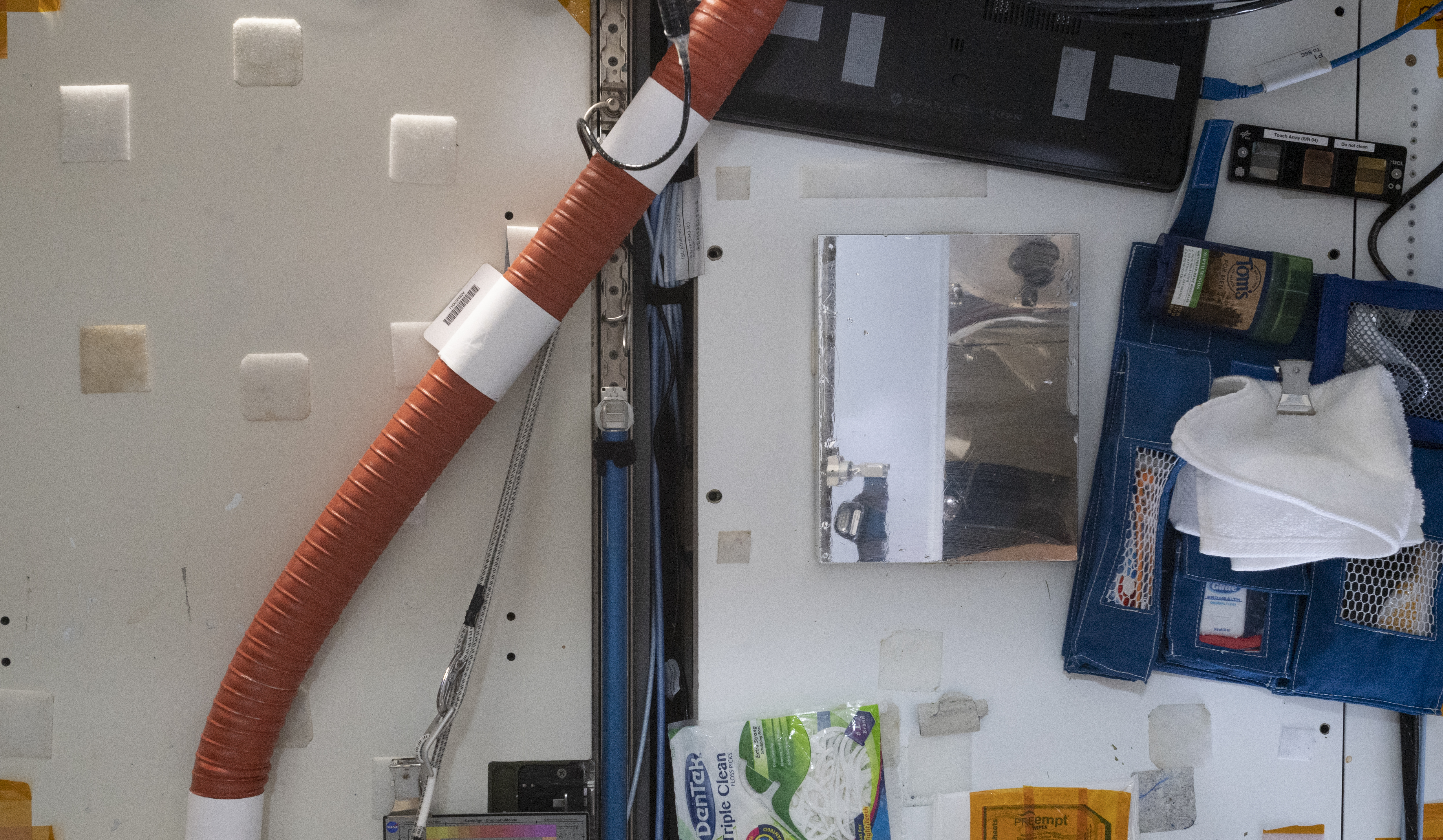

Supplement: S2 Dataset — (ZIP) [file pone.0304229.s003.zip › S05 - 10 - iss066e131835.jpg]

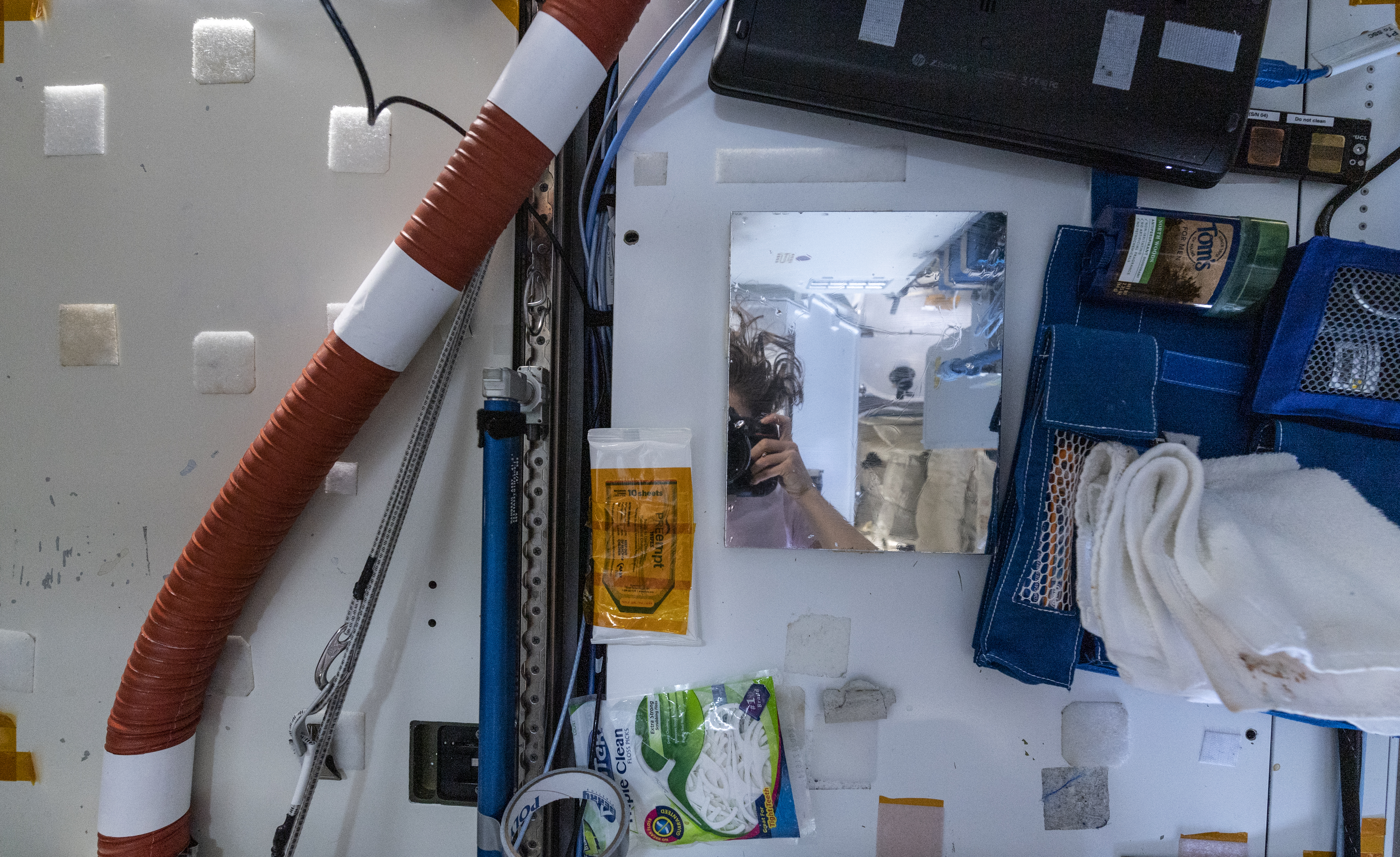

Supplement: S2 Dataset — (ZIP) [file pone.0304229.s003.zip › S05 - 11 - iss066e132464.jpg]

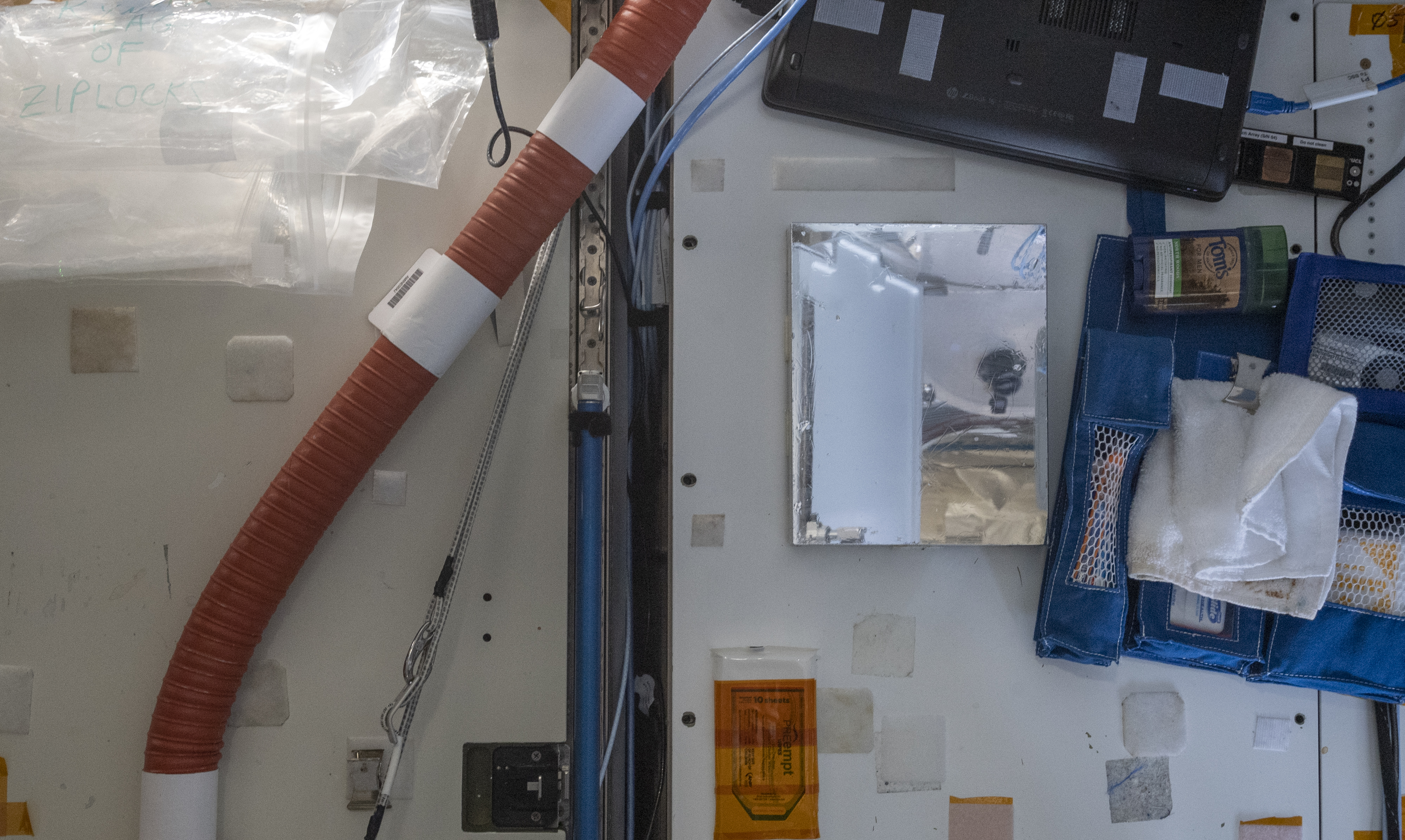

Supplement: S2 Dataset — (ZIP) [file pone.0304229.s003.zip › S05 - 12 - iss066e133920.jpg]

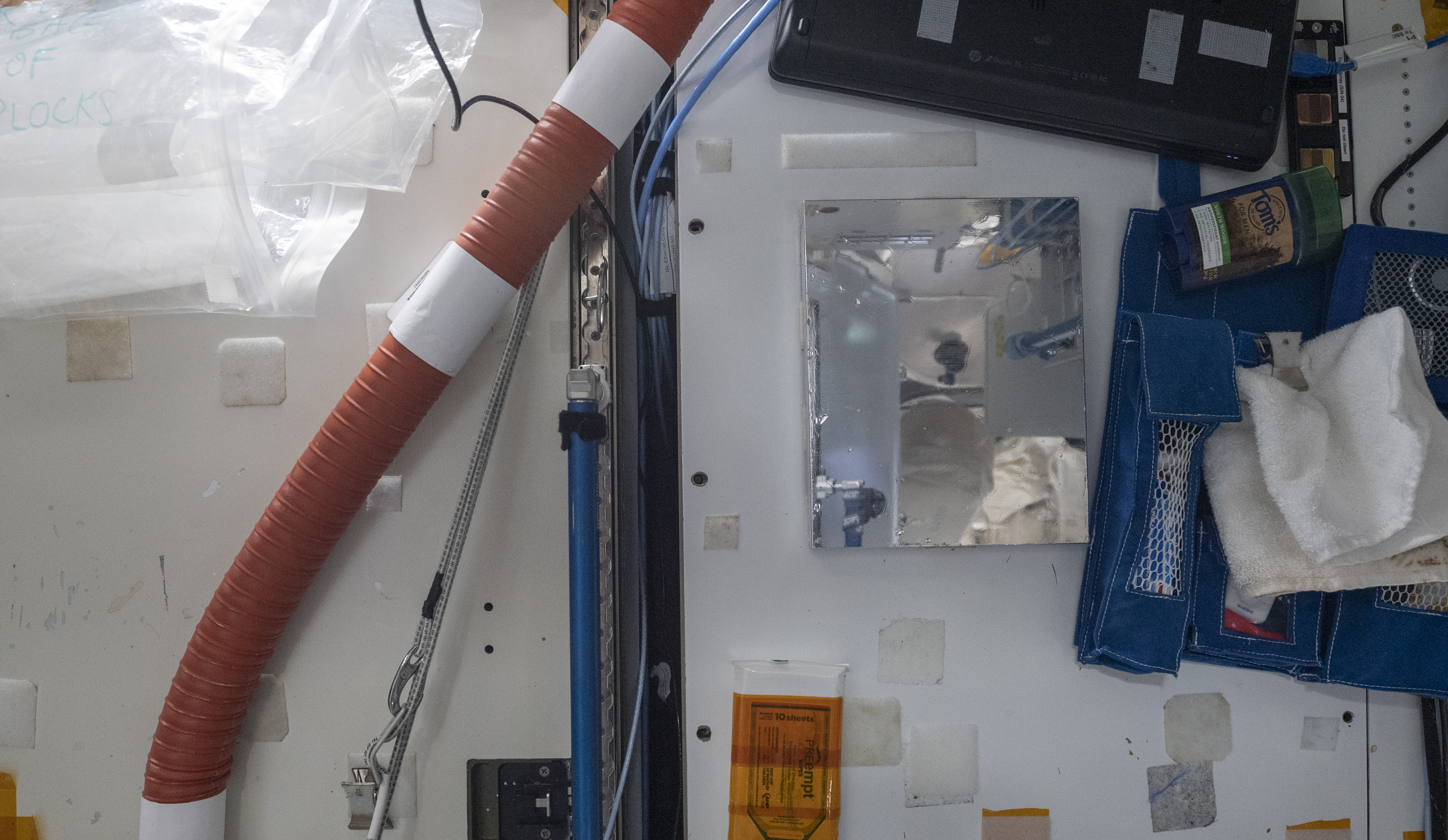

Supplement: S2 Dataset — (ZIP) [file pone.0304229.s003.zip › S05 - 13 - iss066e134844.jpg]

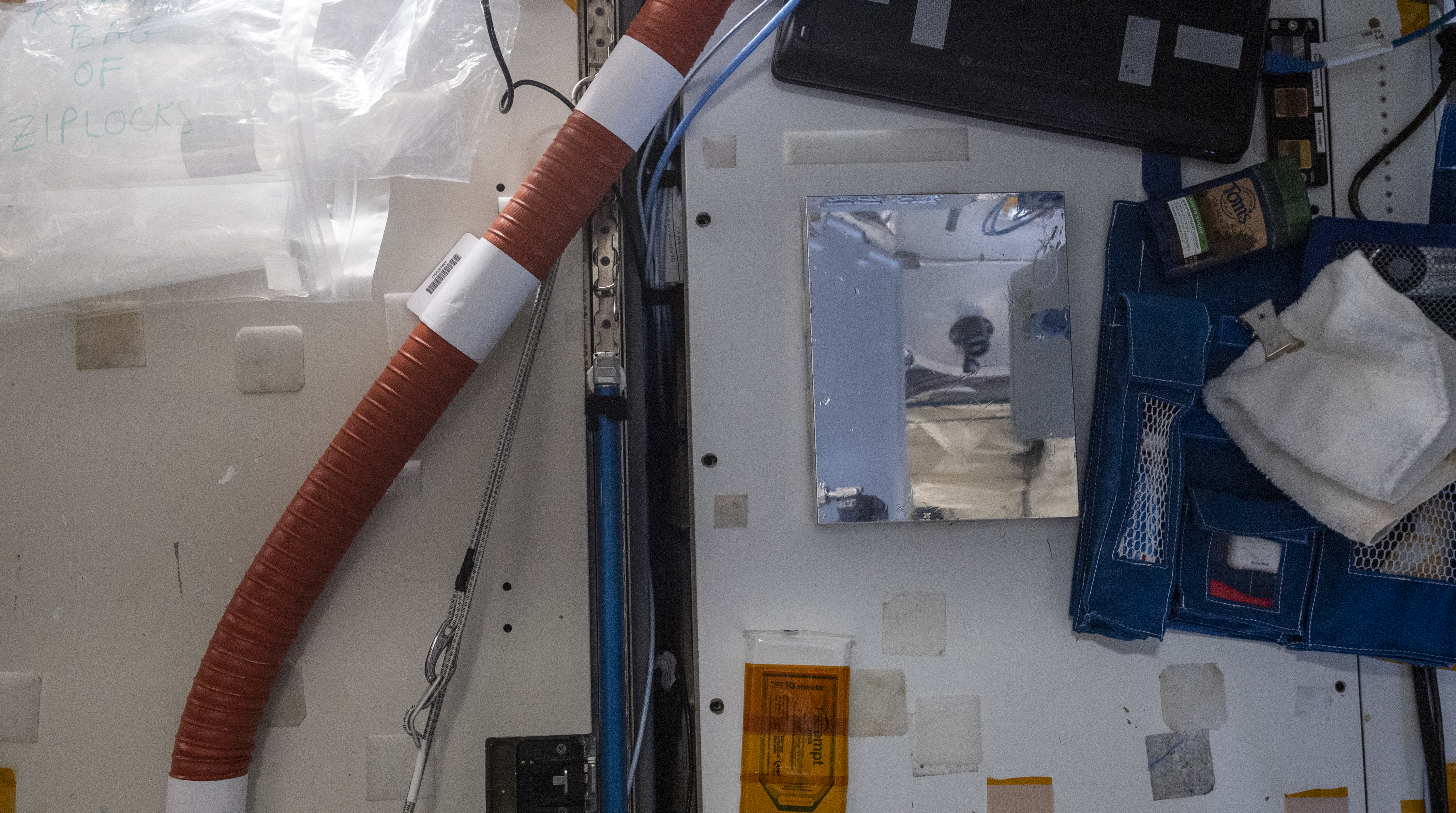

Supplement: S2 Dataset — (ZIP) [file pone.0304229.s003.zip › S05 - 14 - iss066e135860.jpg]

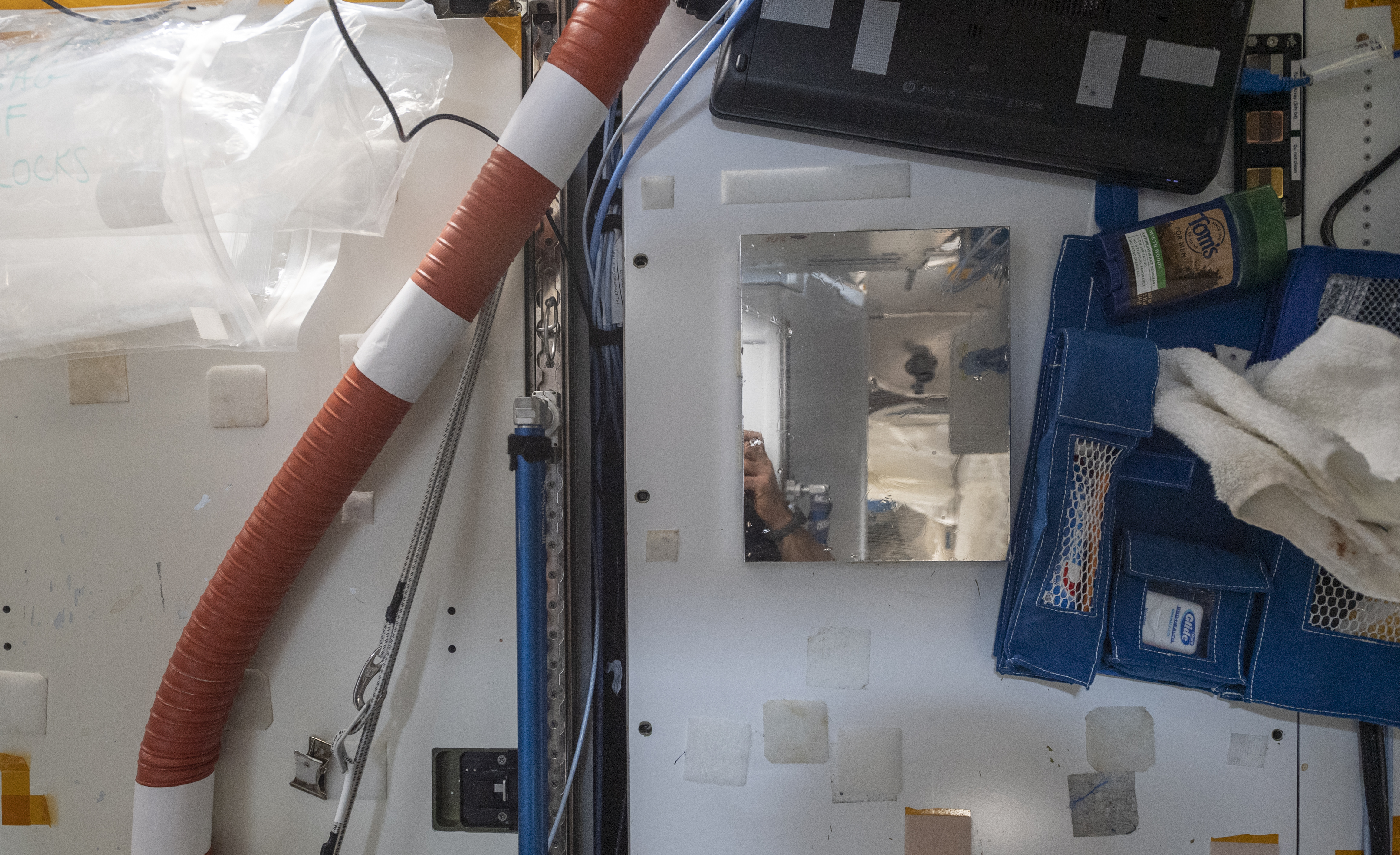

Supplement: S2 Dataset — (ZIP) [file pone.0304229.s003.zip › S05 - 15 - iss066e136141.jpg]

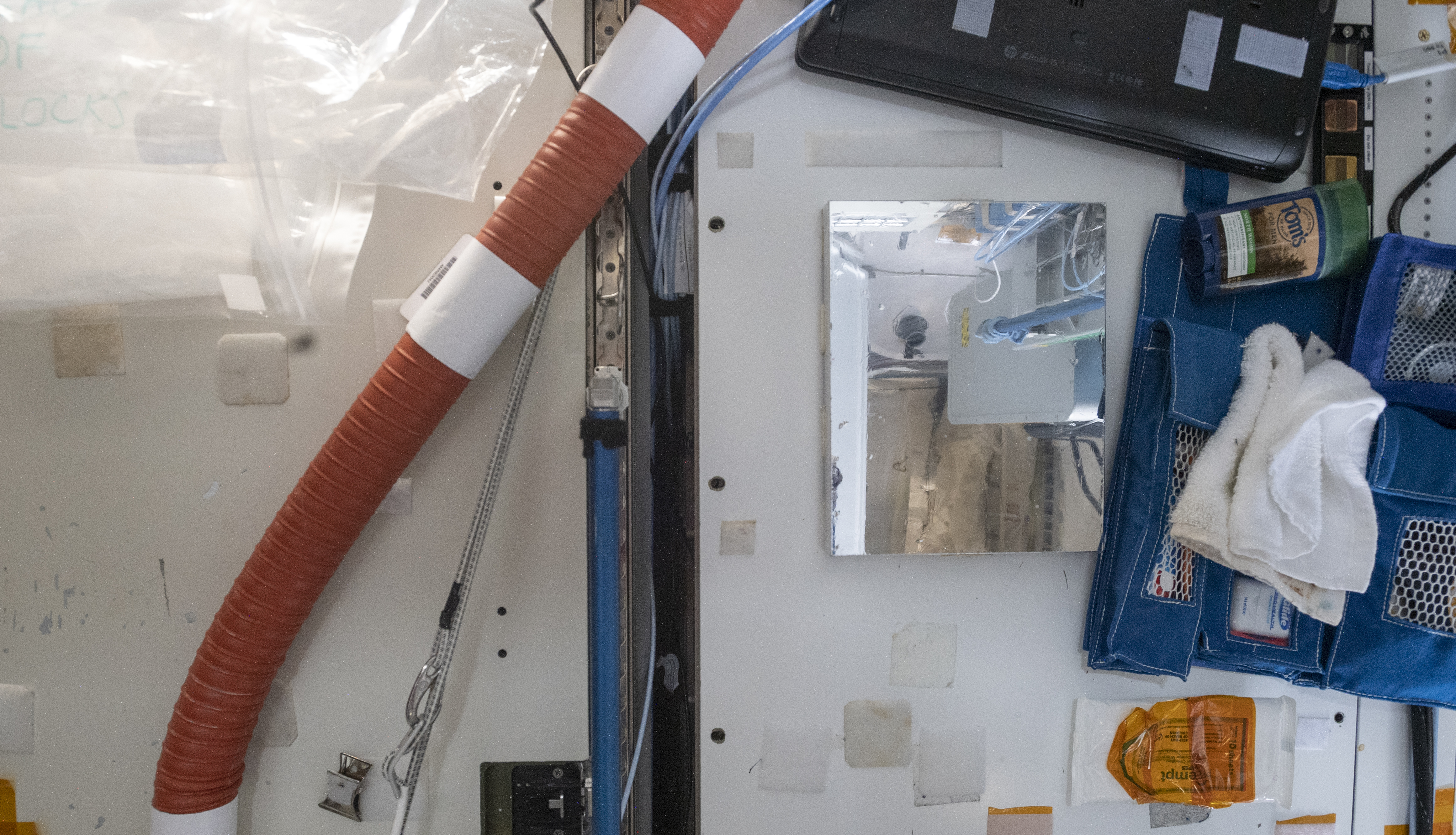

Supplement: S2 Dataset — (ZIP) [file pone.0304229.s003.zip › S05 - 16 - iss066e136639.jpg]

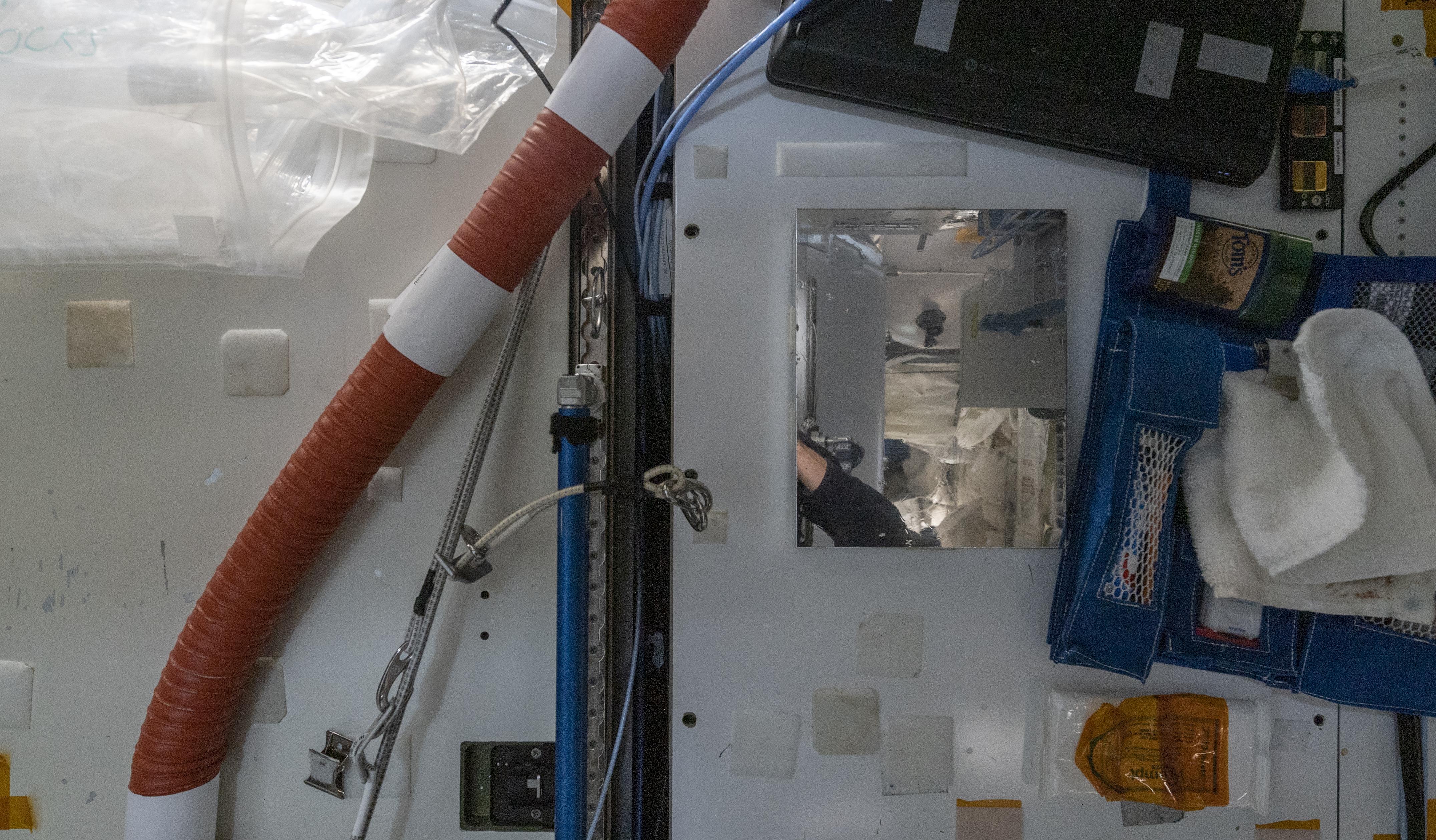

Supplement: S2 Dataset — (ZIP) [file pone.0304229.s003.zip › S05 - 17 - iss066e136647.jpg]

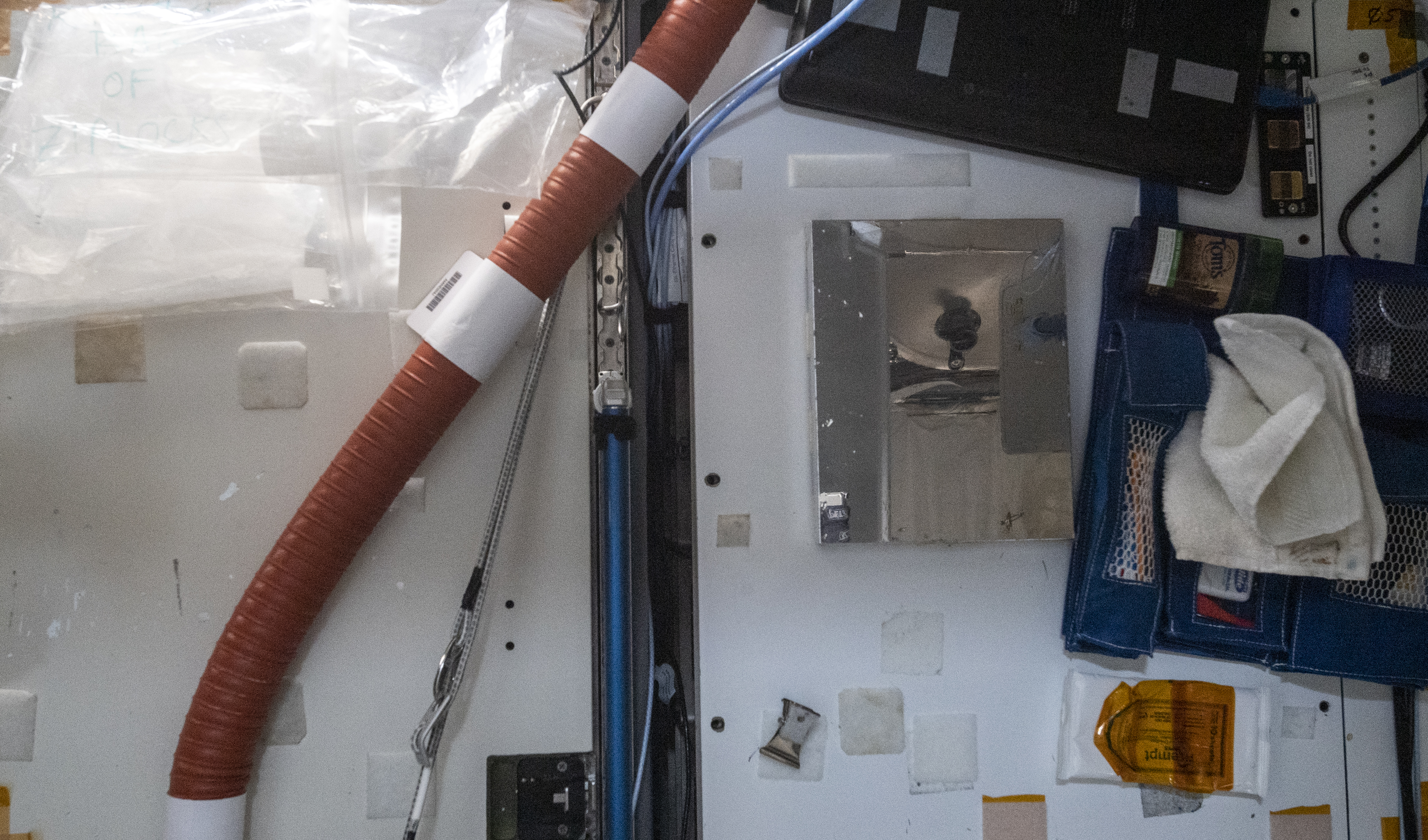

Supplement: S2 Dataset — (ZIP) [file pone.0304229.s003.zip › S05 - 18 - iss066e137063.jpg]

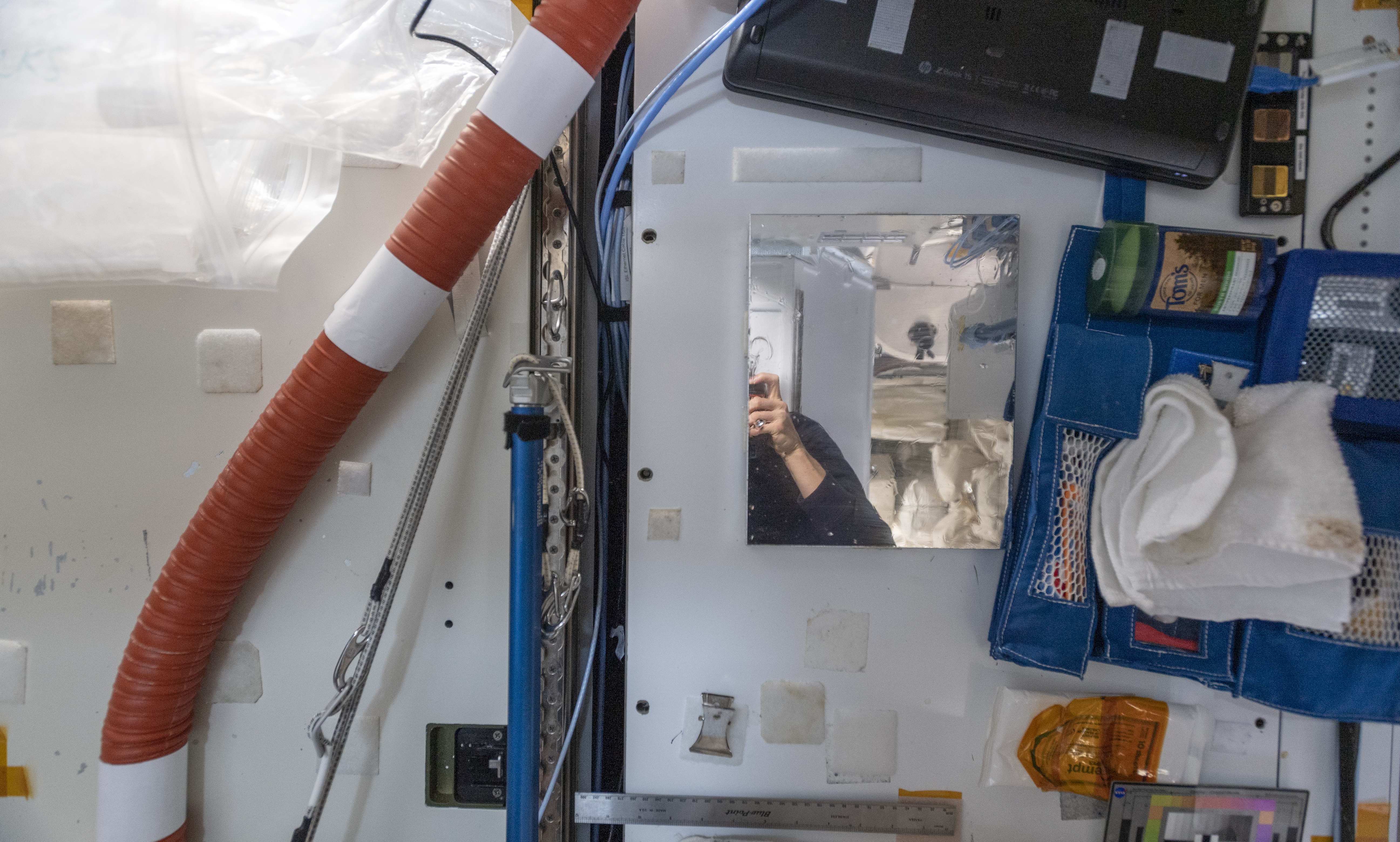

Supplement: S2 Dataset — (ZIP) [file pone.0304229.s003.zip › S05 - 19 - iss066e137912.jpg]

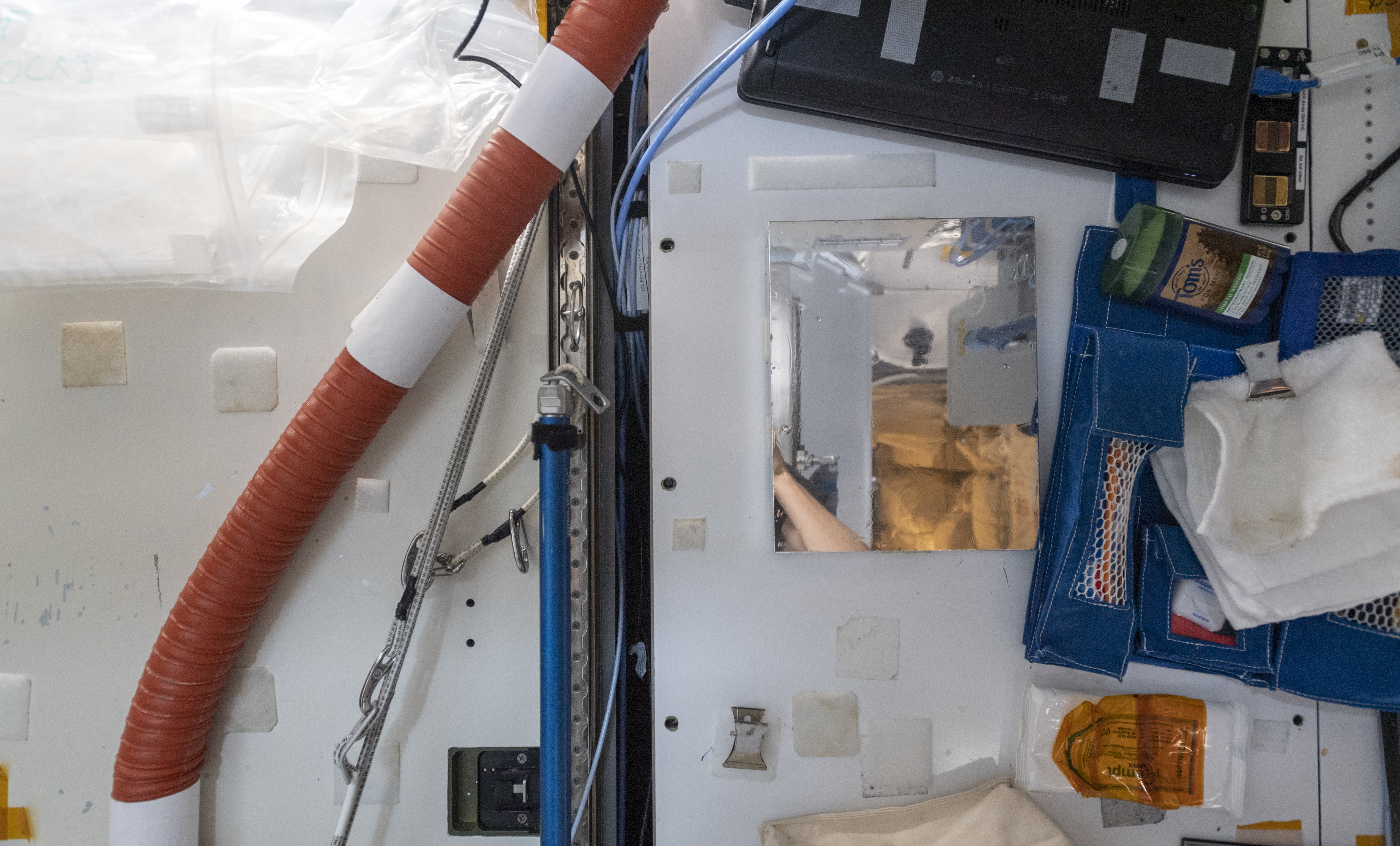

Supplement: S2 Dataset — (ZIP) [file pone.0304229.s003.zip › S05 - 20 - iss066e138189.jpg]

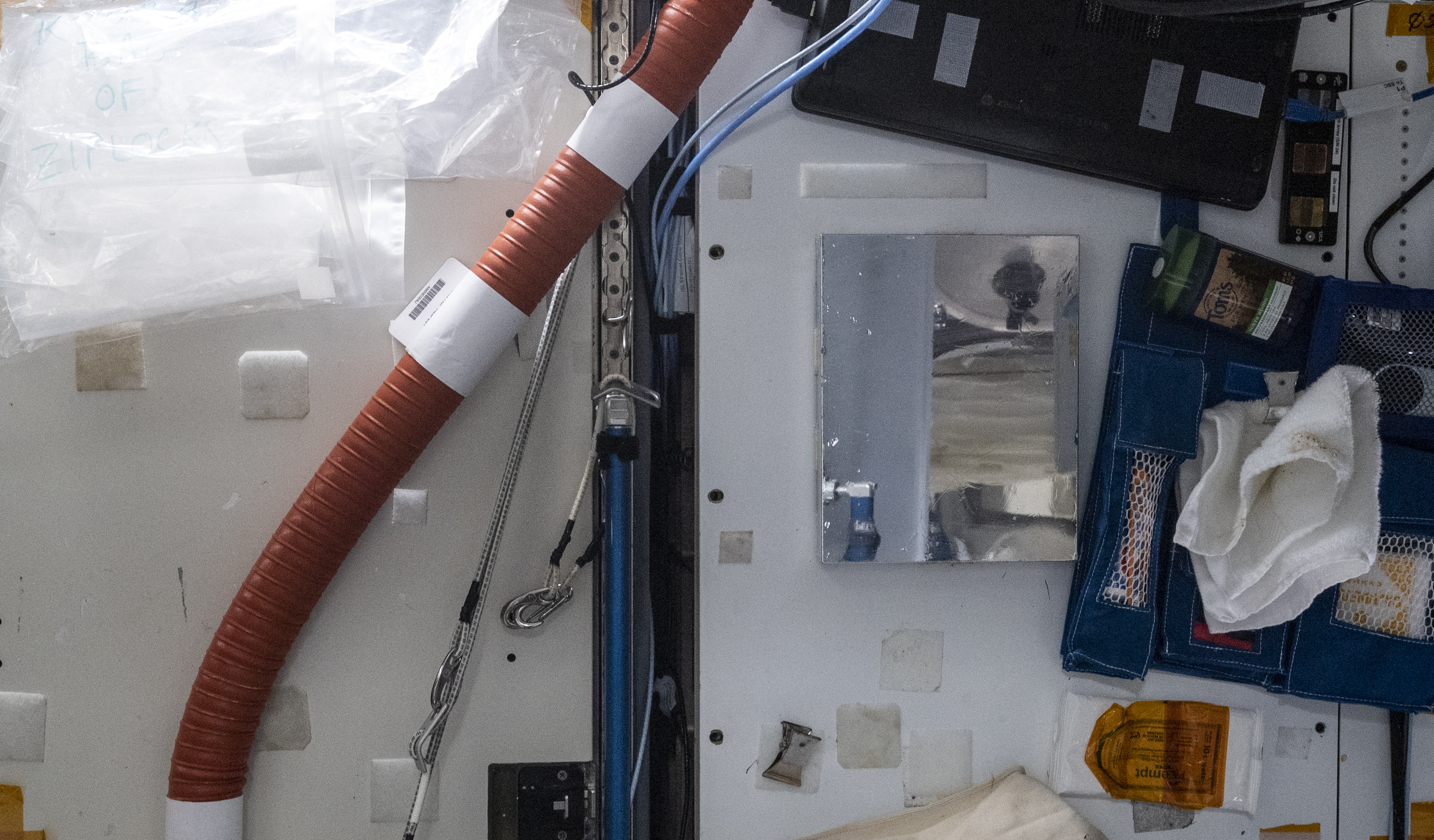

Supplement: S2 Dataset — (ZIP) [file pone.0304229.s003.zip › S05 - 21 - iss066e138225.jpg]

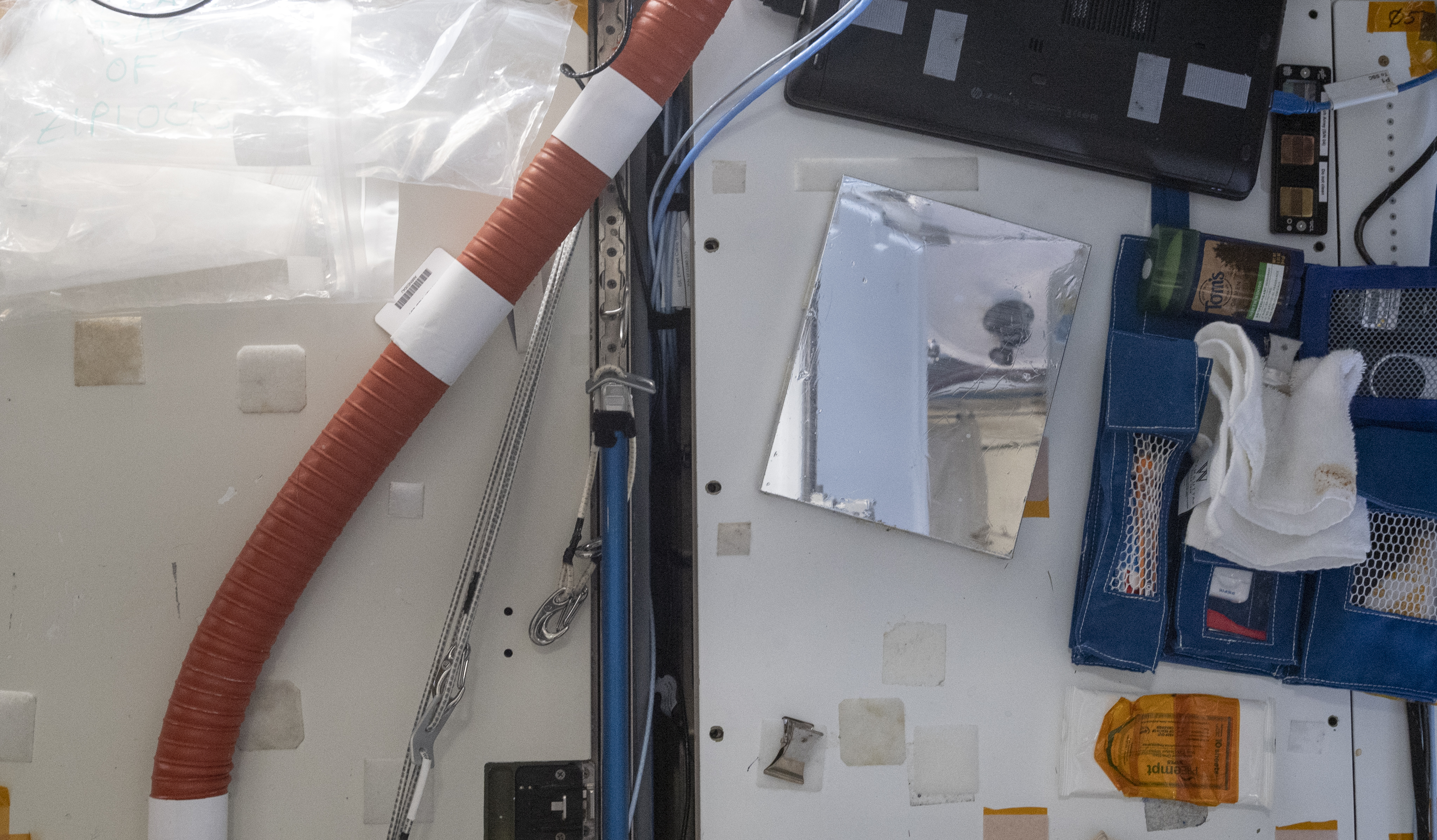

Supplement: S2 Dataset — (ZIP) [file pone.0304229.s003.zip › S05 - 22 - iss066e140915.jpg]

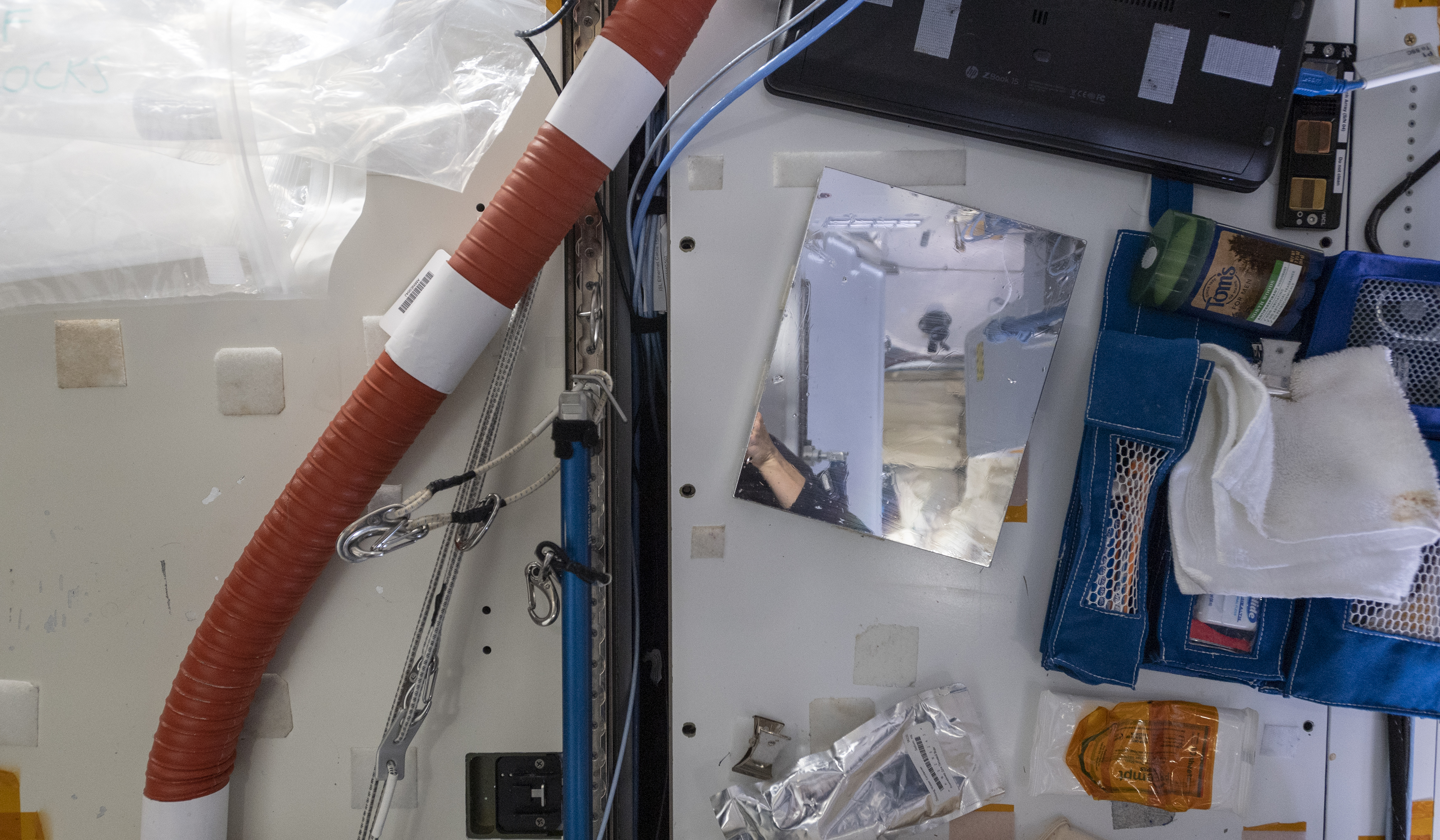

Supplement: S2 Dataset — (ZIP) [file pone.0304229.s003.zip › S05 - 23 - iss066e141174.jpg]

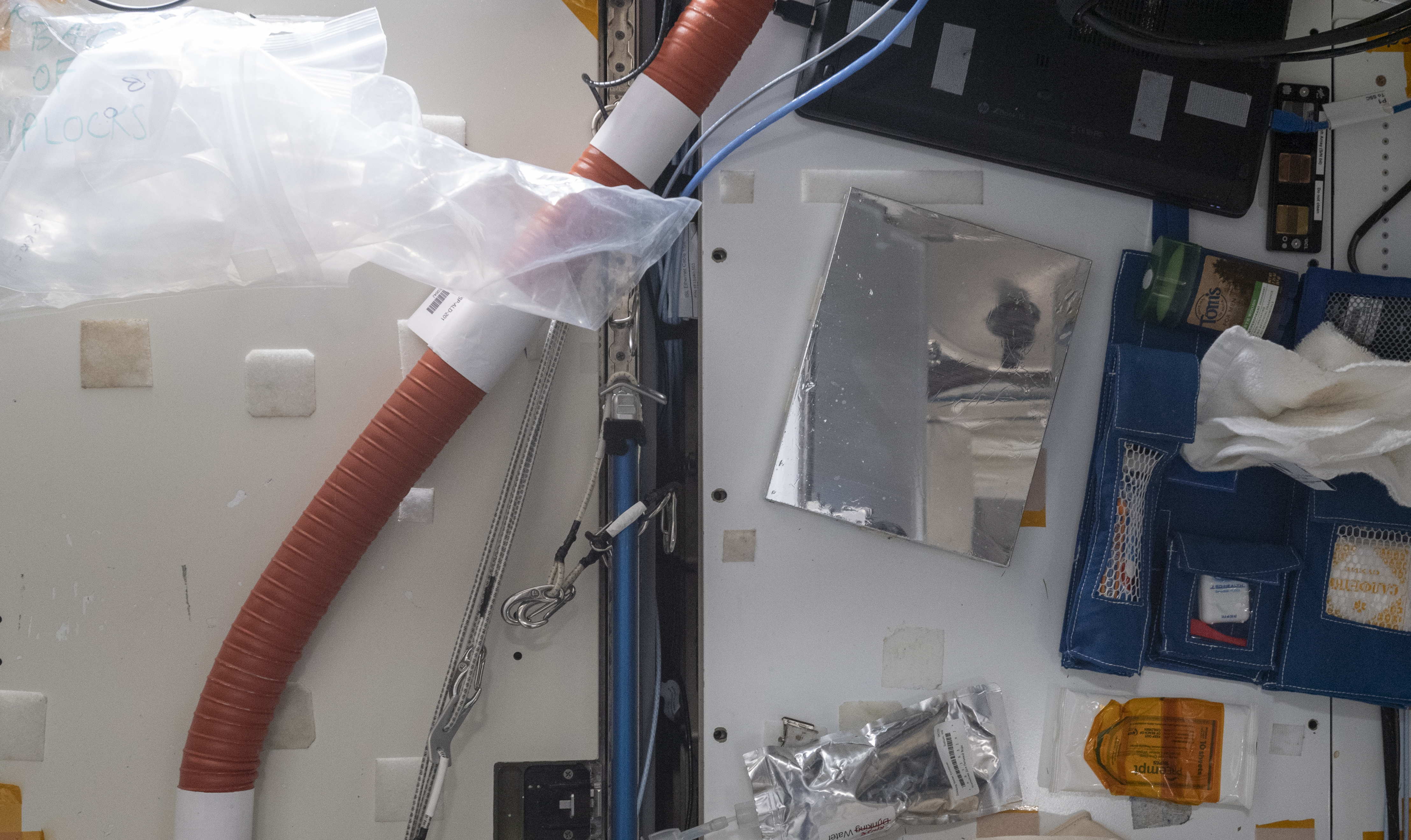

Supplement: S2 Dataset — (ZIP) [file pone.0304229.s003.zip › S05 - 24 - iss066e140935.jpg]

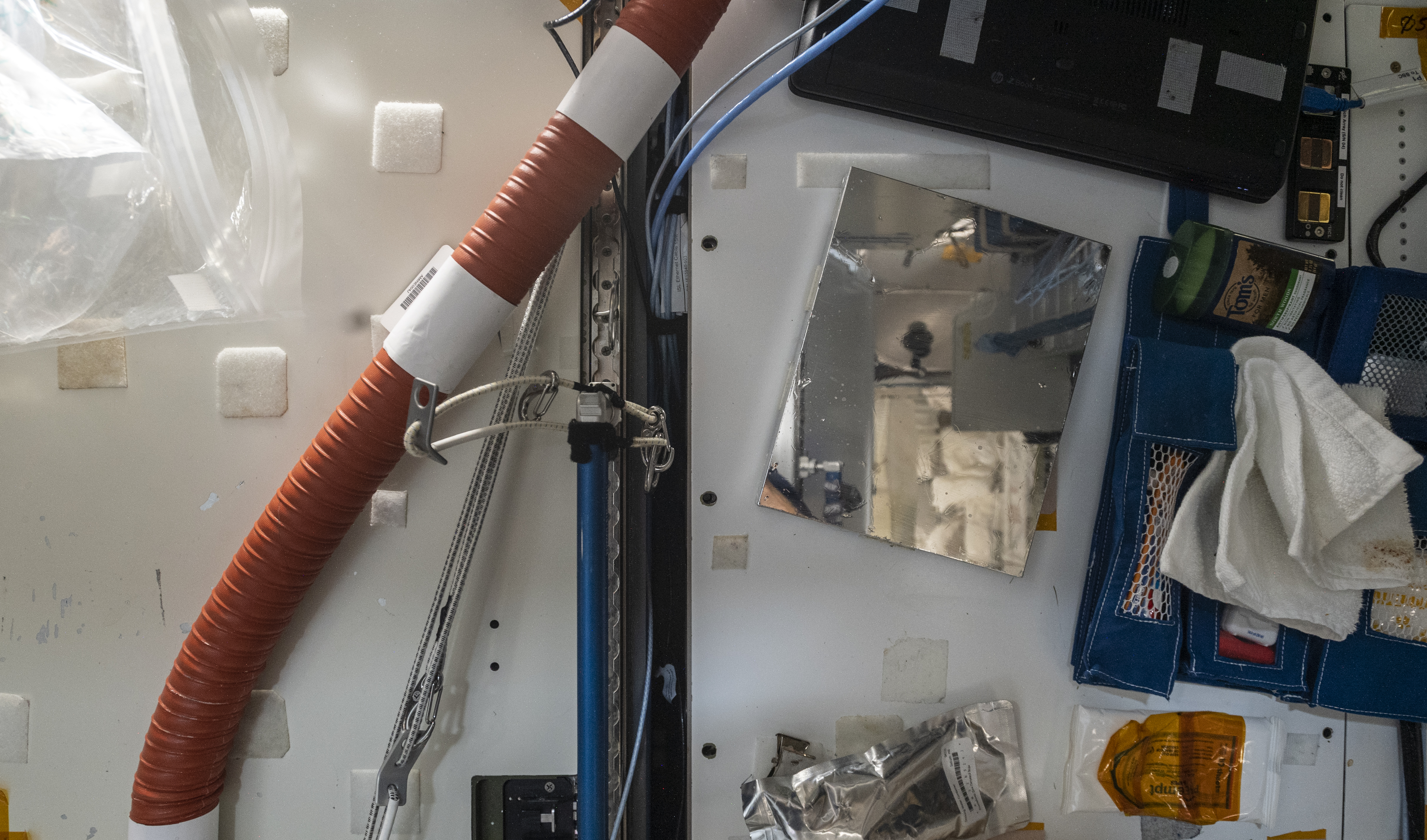

Supplement: S2 Dataset — (ZIP) [file pone.0304229.s003.zip › S05 - 25 - iss066e143011.jpg]

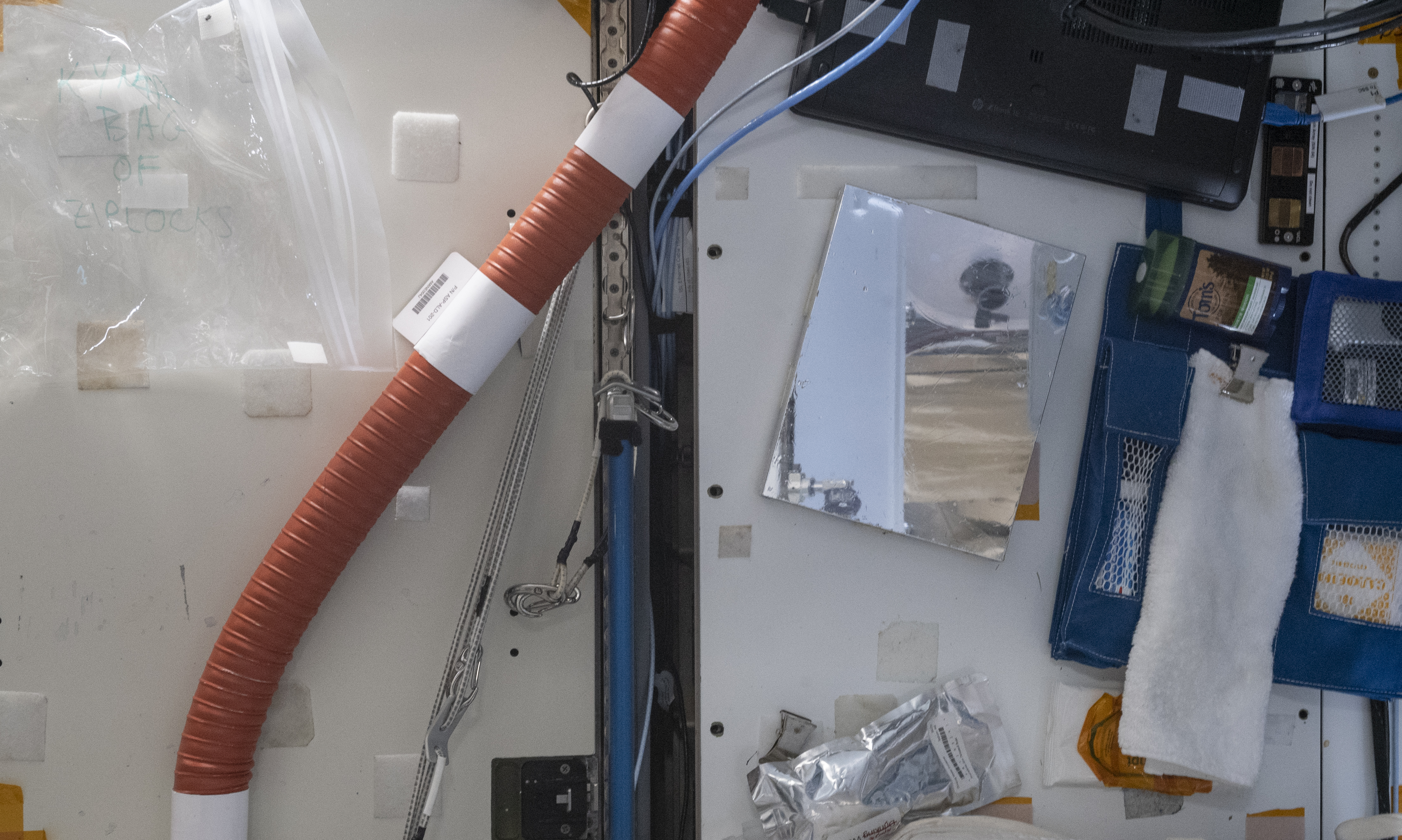

Supplement: S2 Dataset — (ZIP) [file pone.0304229.s003.zip › S05 - 26 - iss066e144209.jpg]

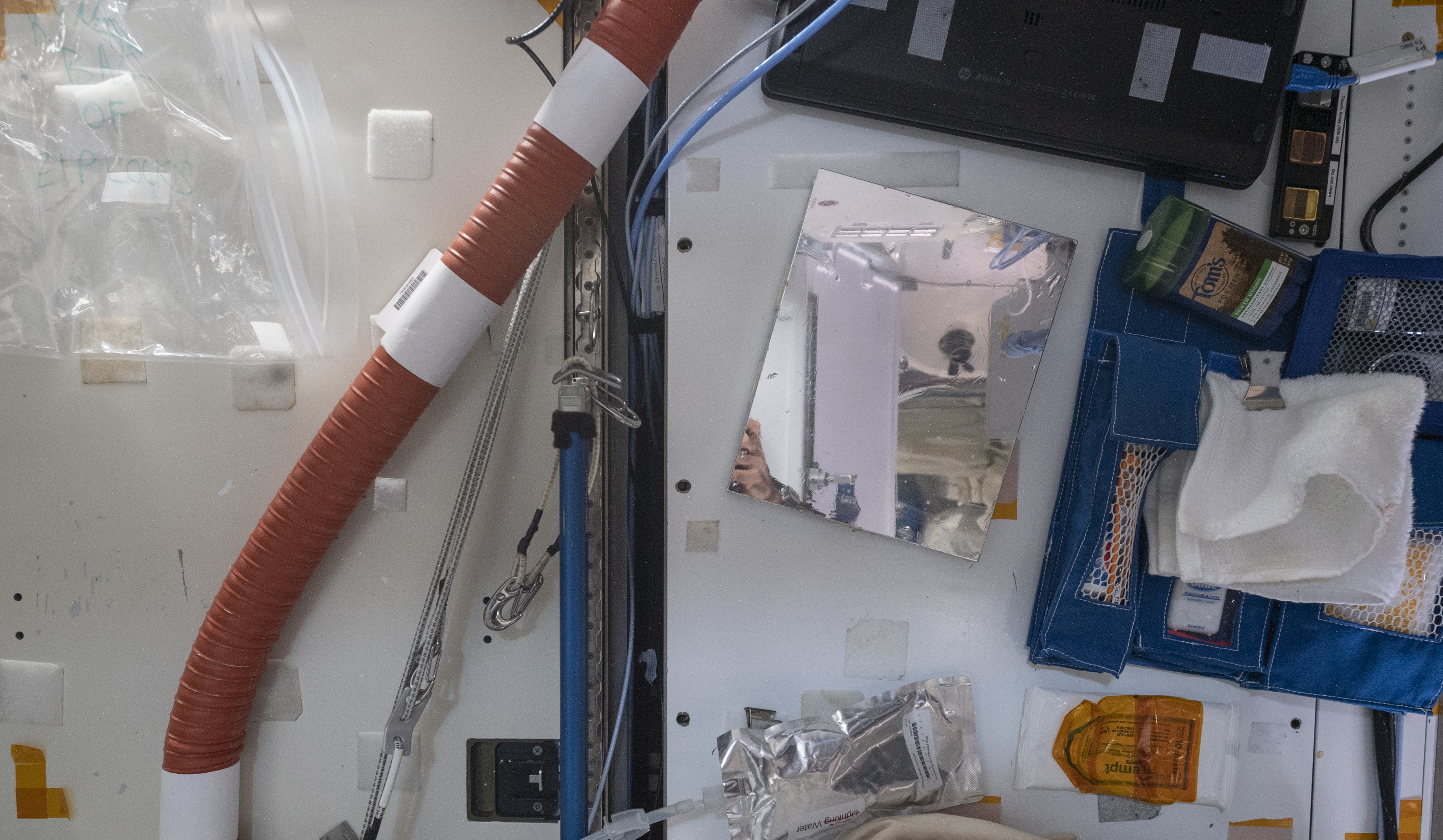

Supplement: S2 Dataset — (ZIP) [file pone.0304229.s003.zip › S05 - 27 - iss066e145803.jpg]

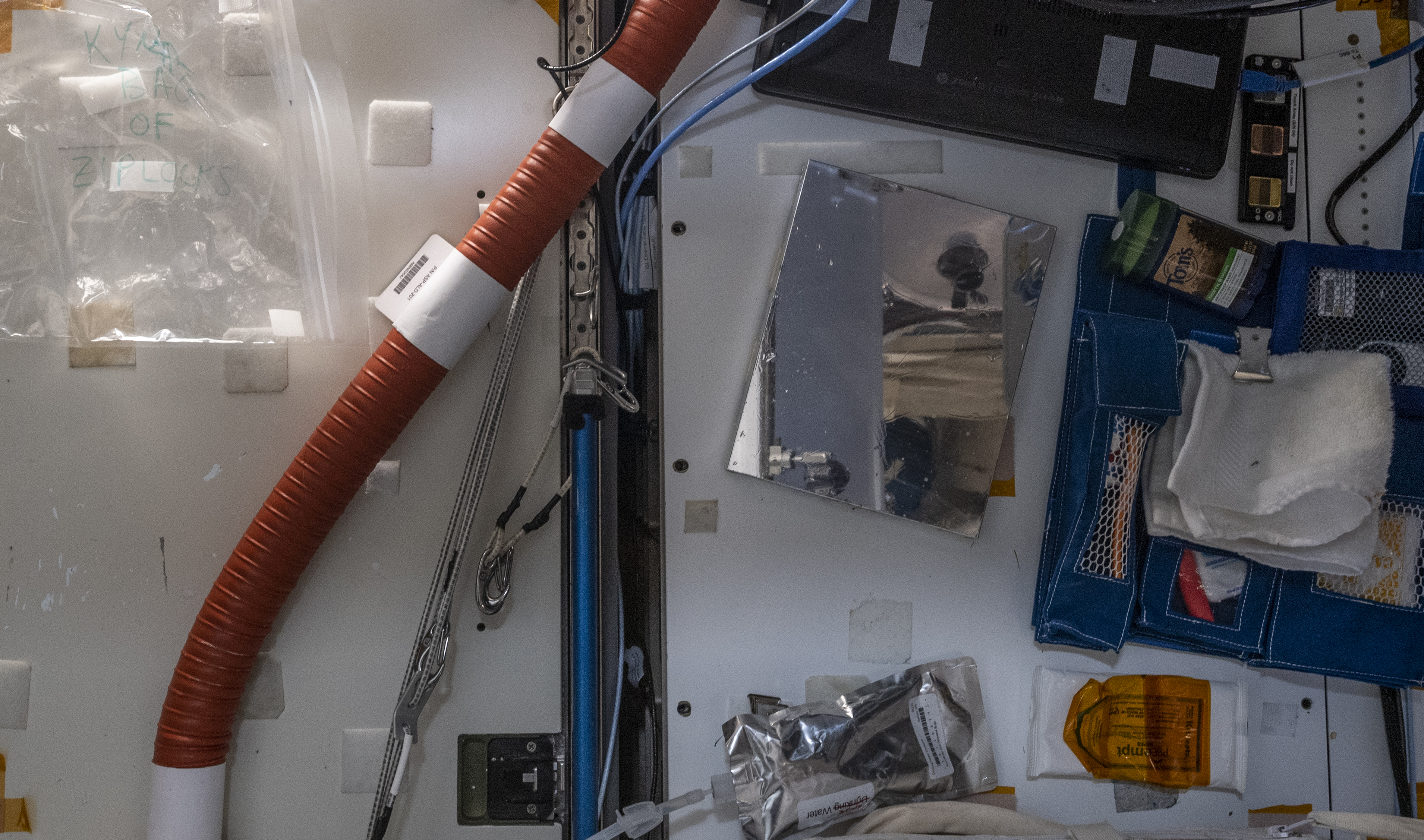

Supplement: S2 Dataset — (ZIP) [file pone.0304229.s003.zip › S05 - 28 - iss066e145991.jpg]

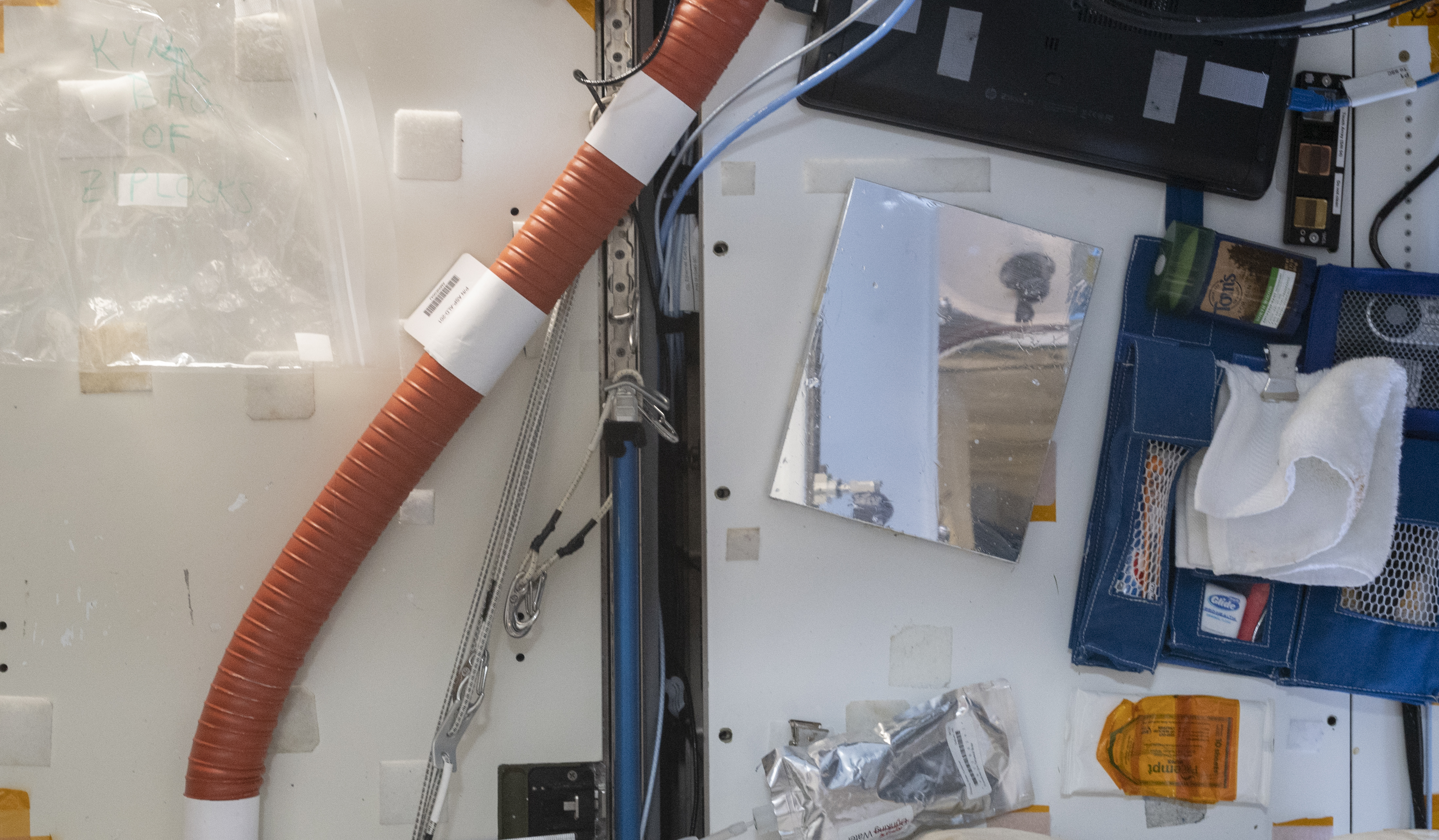

Supplement: S2 Dataset — (ZIP) [file pone.0304229.s003.zip › S05 - 29 - iss066e146570-2.jpg]

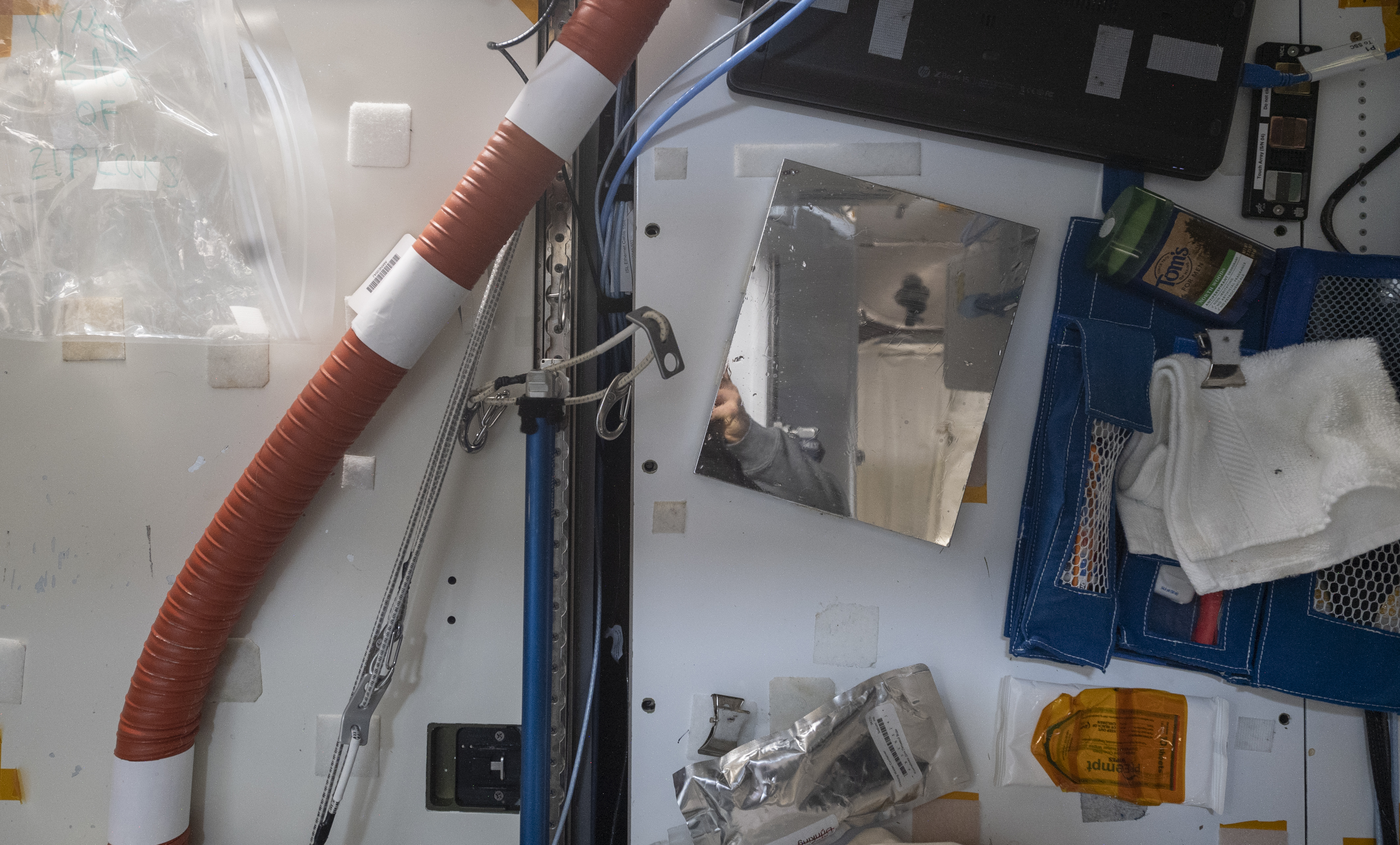

Supplement: S2 Dataset — (ZIP) [file pone.0304229.s003.zip › S05 - 30 - iss066e146496.jpg]

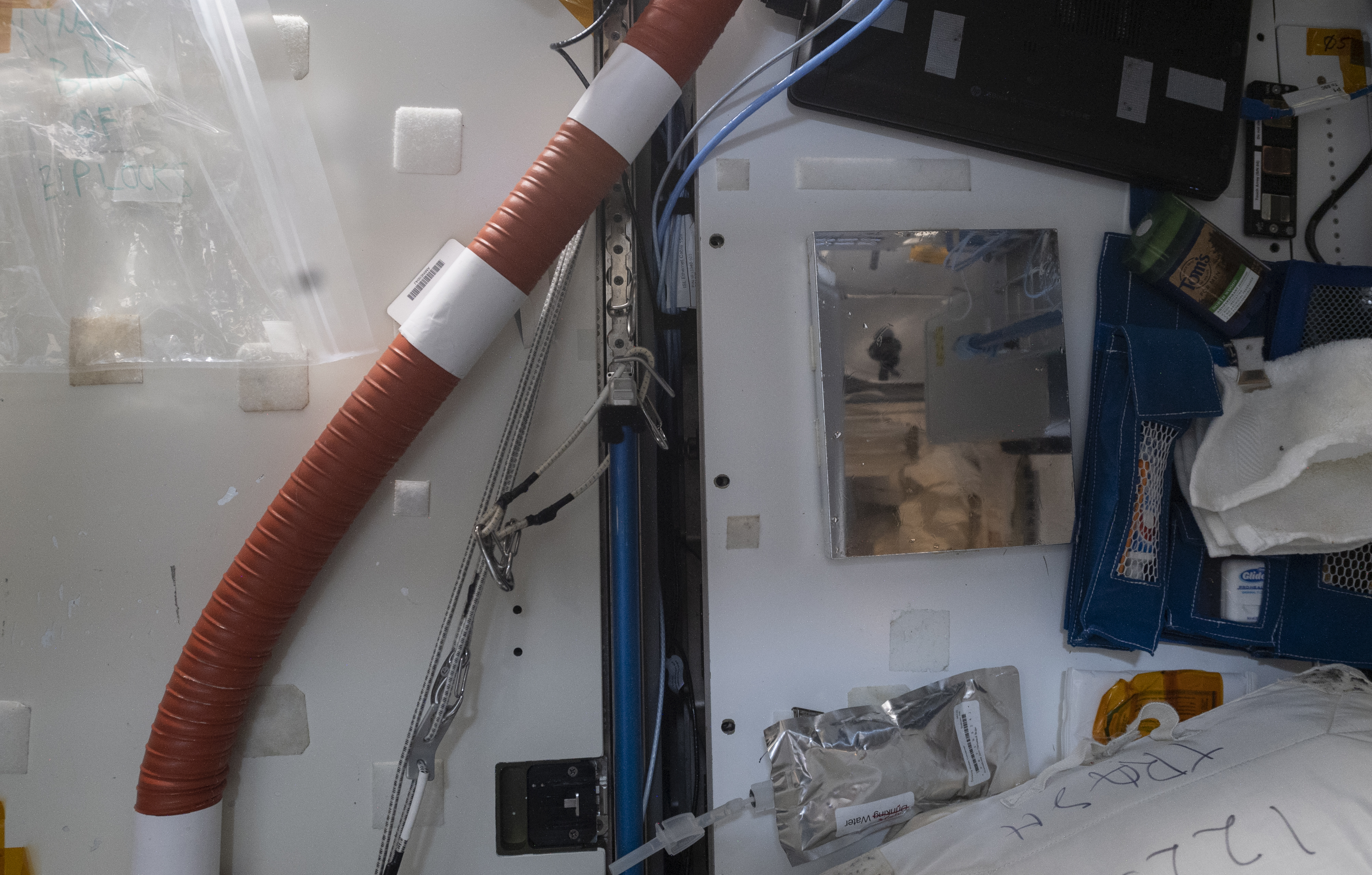

Supplement: S2 Dataset — (ZIP) [file pone.0304229.s003.zip › S05 - 31 - iss066e146509.jpg]

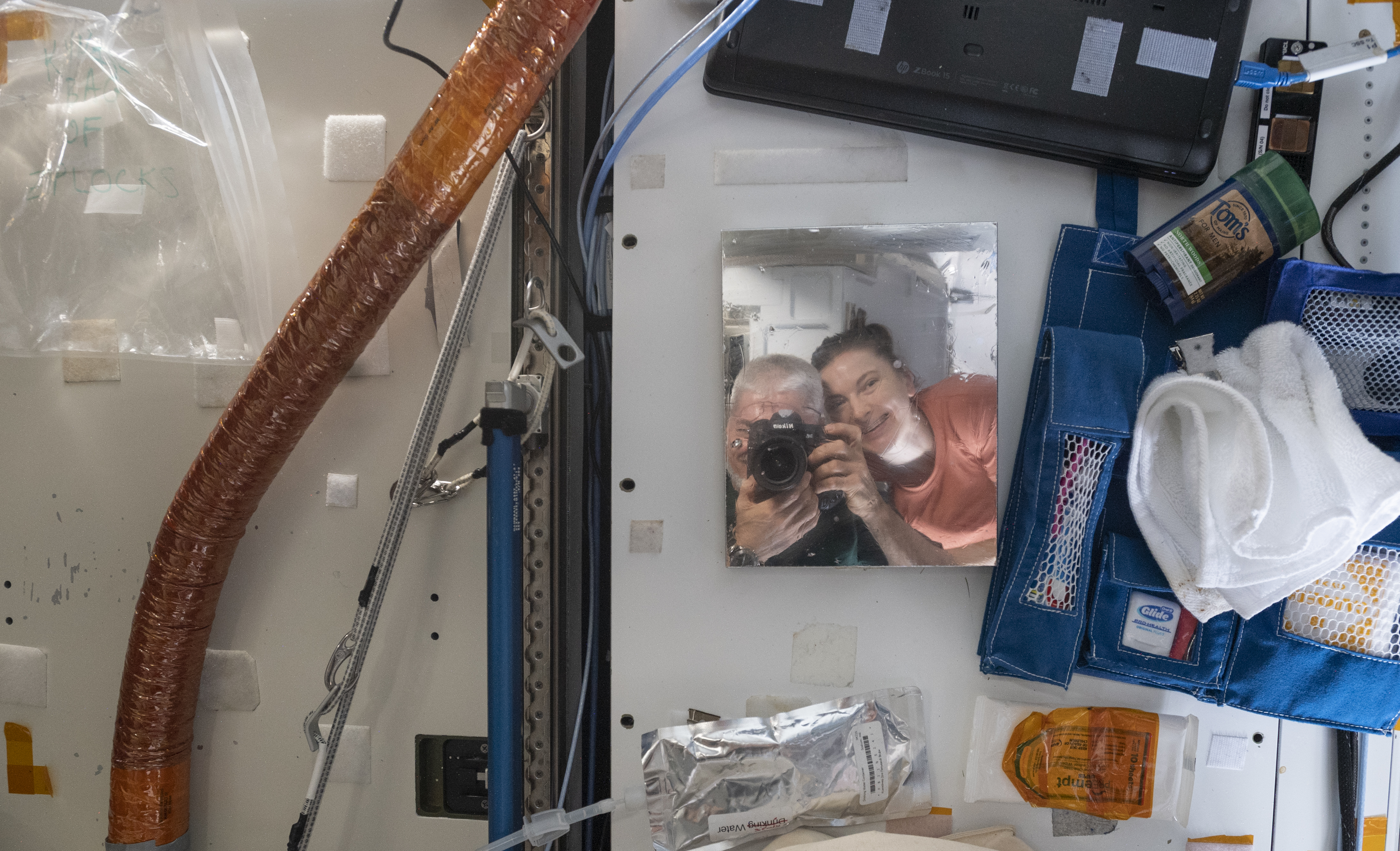

Supplement: S2 Dataset — (ZIP) [file pone.0304229.s003.zip › S05 - 32 - iss066e146542.jpg]

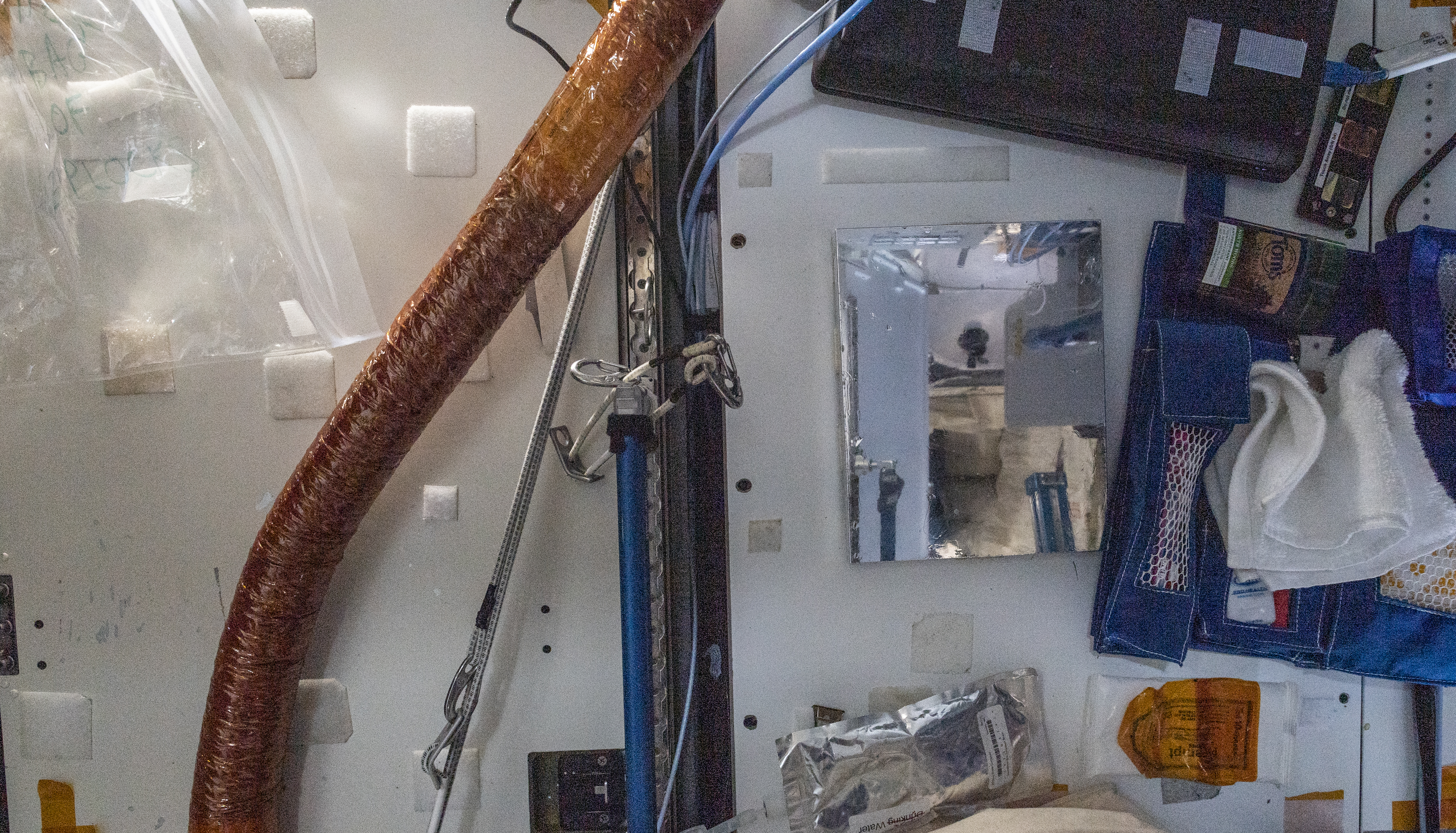

Supplement: S2 Dataset — (ZIP) [file pone.0304229.s003.zip › S05 - 33 - iss066e151985.jpg]

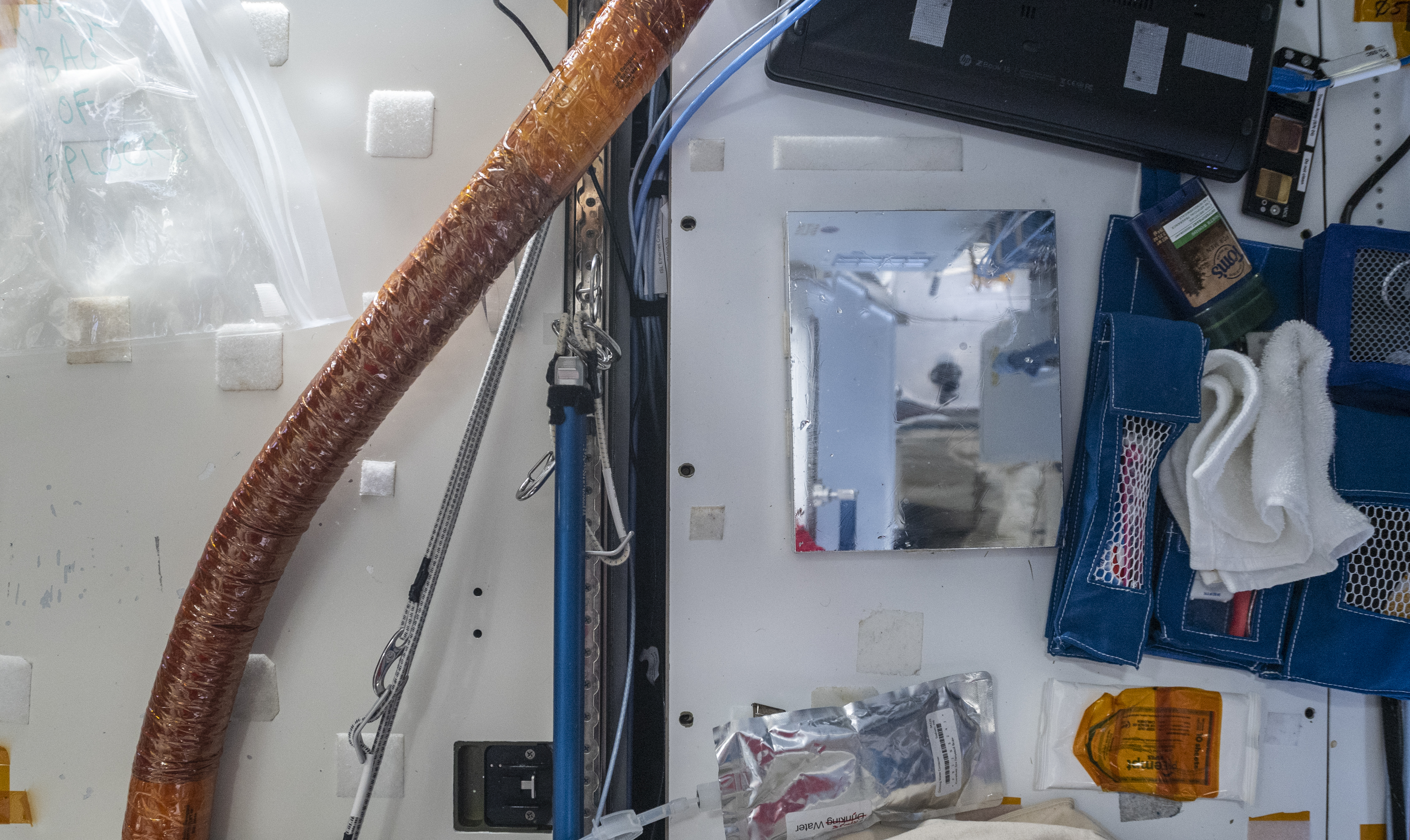

Supplement: S2 Dataset — (ZIP) [file pone.0304229.s003.zip › S05 - 34 - iss066e152121.jpg]

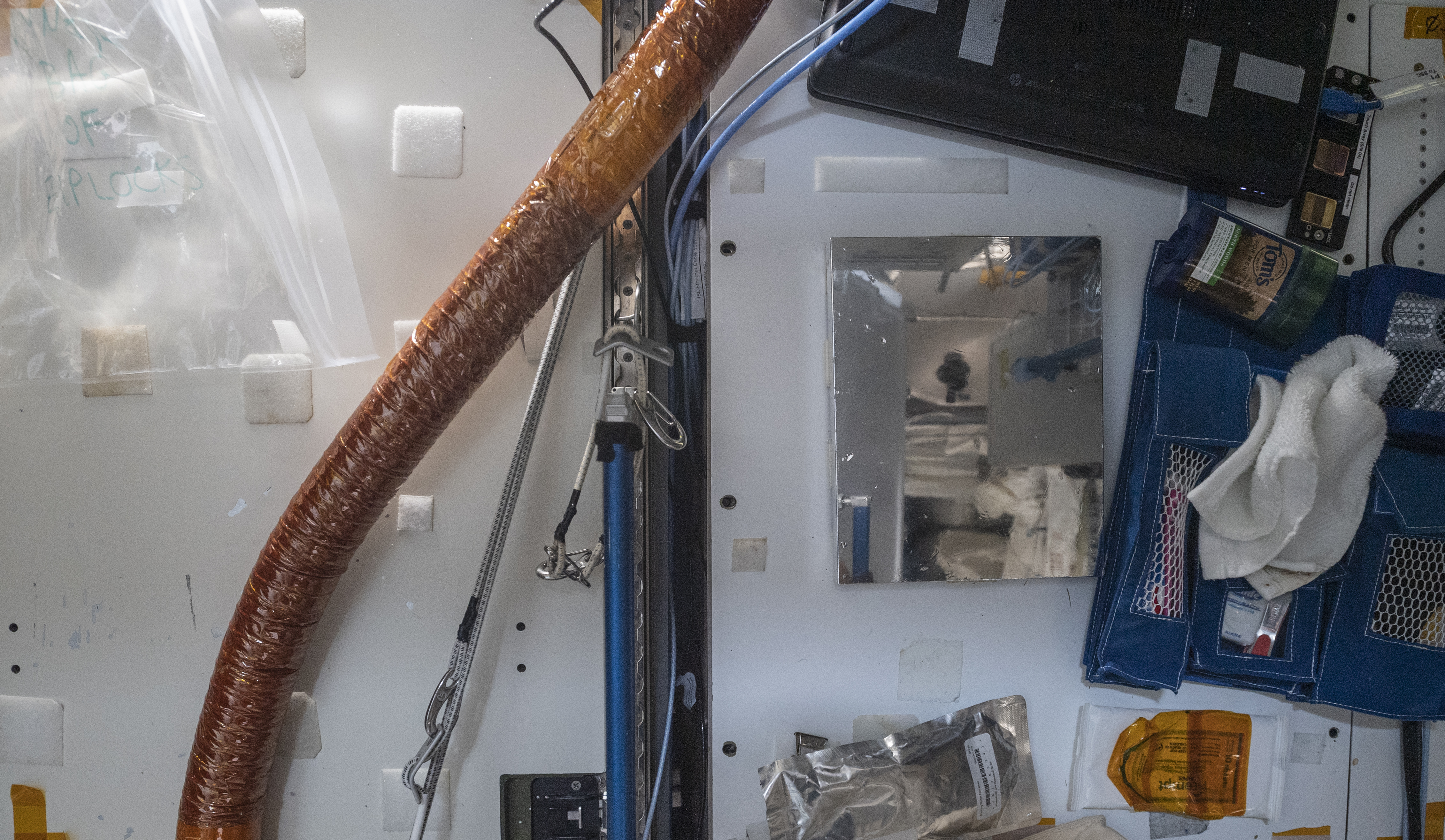

Supplement: S2 Dataset — (ZIP) [file pone.0304229.s003.zip › S05 - 35 - iss066e152780.jpg]

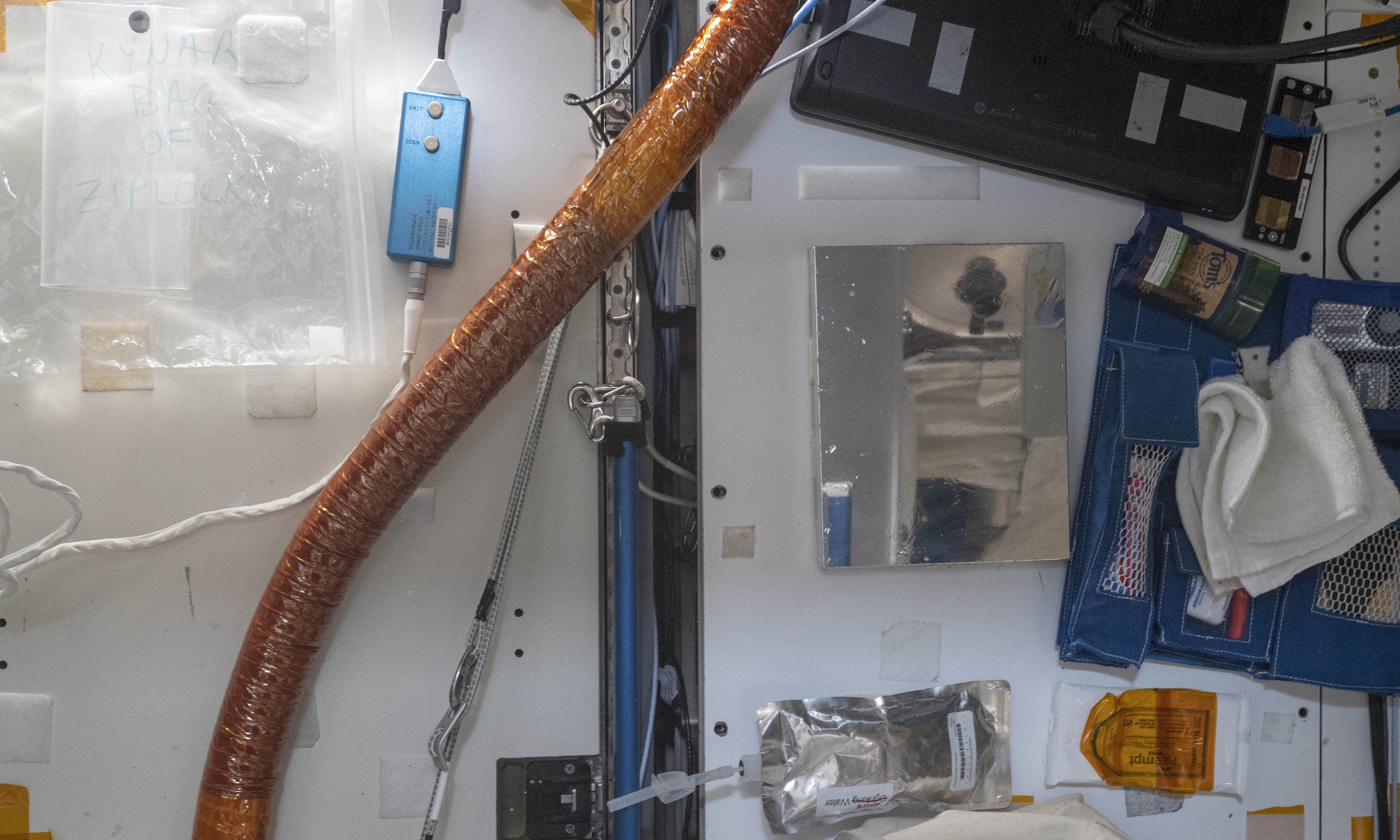

Supplement: S2 Dataset — (ZIP) [file pone.0304229.s003.zip › S05 - 36 - iss066e153152.jpg]

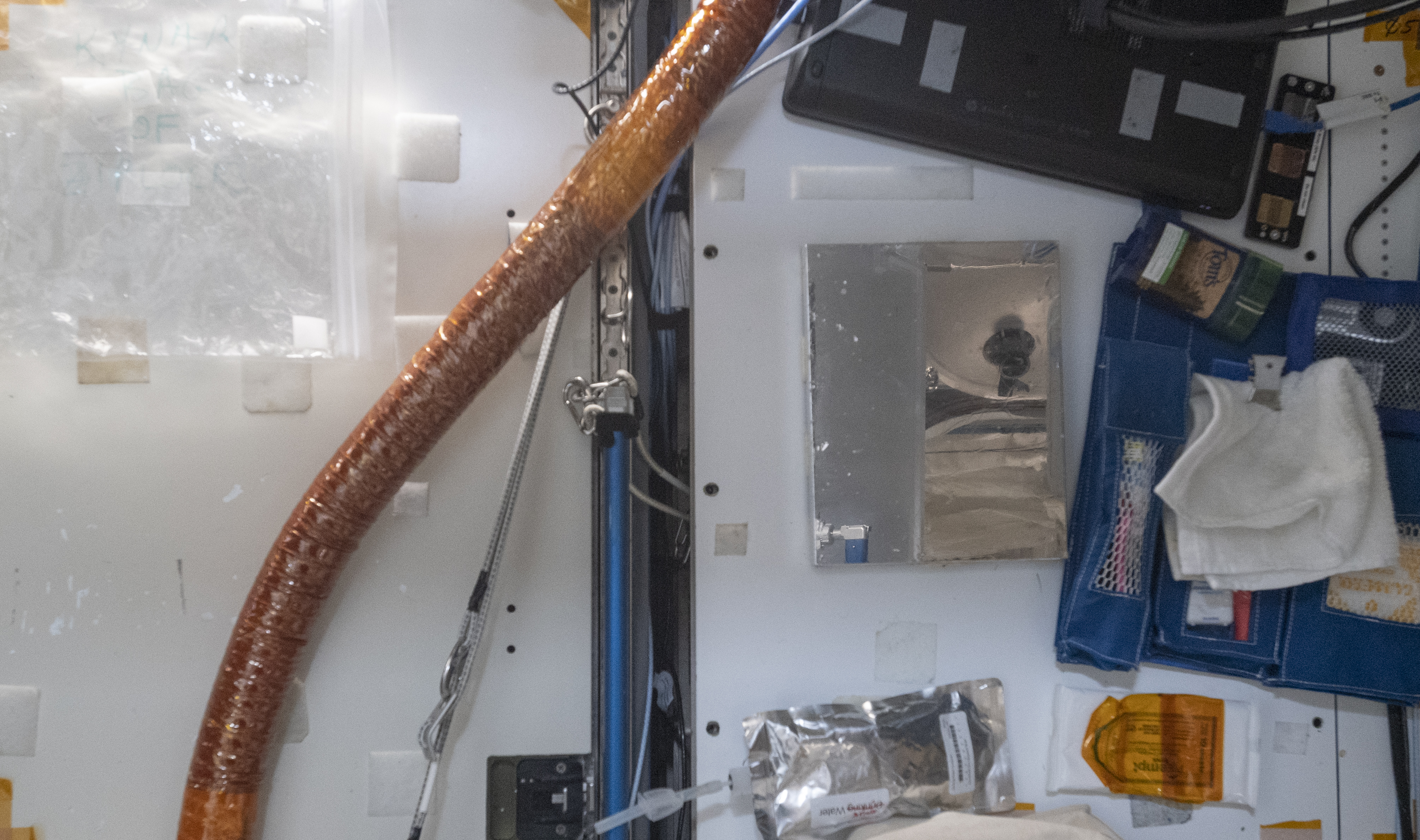

Supplement: S2 Dataset — (ZIP) [file pone.0304229.s003.zip › S05 - 37 - iss066e153176.jpg]

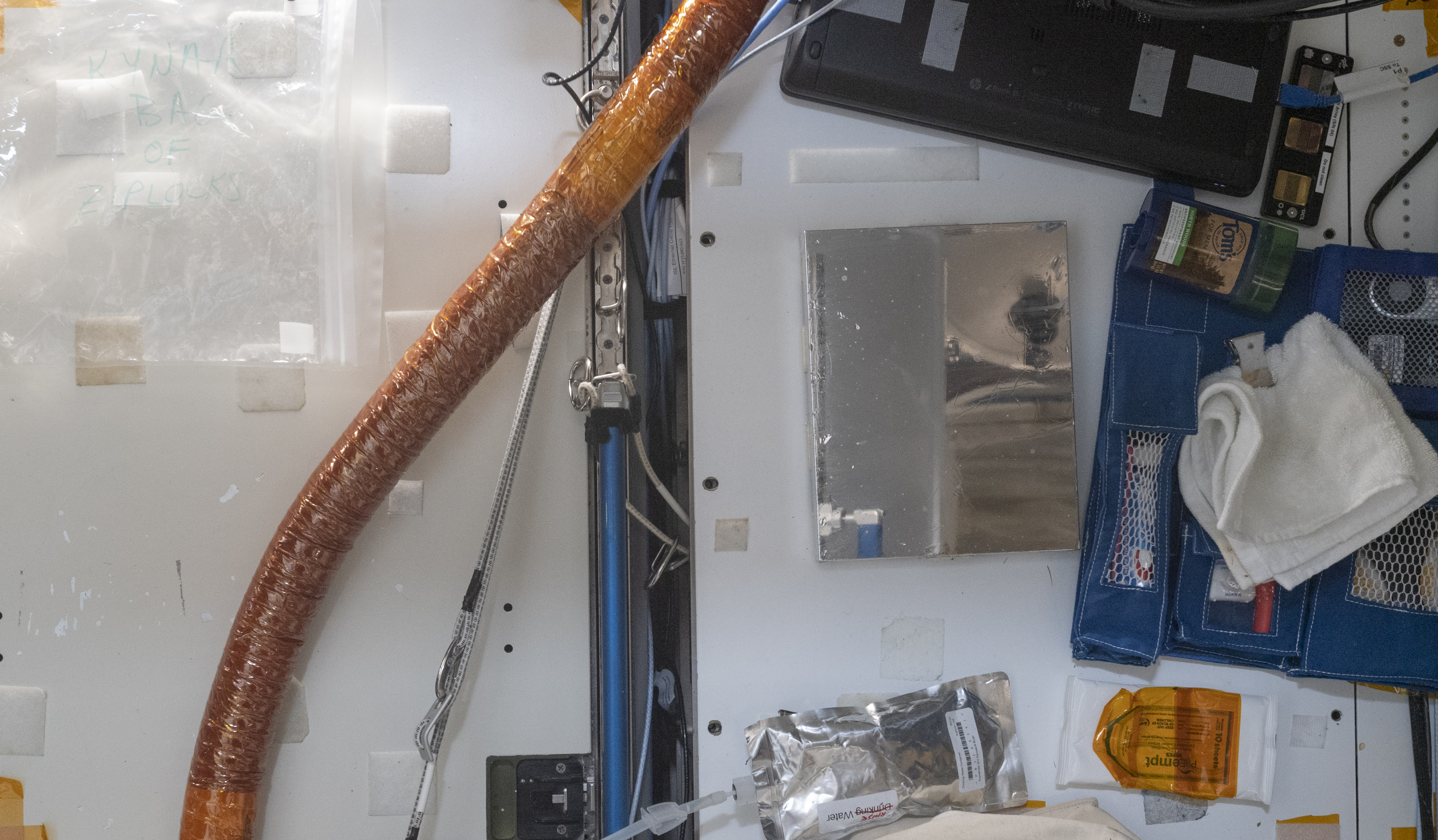

Supplement: S2 Dataset — (ZIP) [file pone.0304229.s003.zip › S05 - 38 - iss066e153268.jpg]

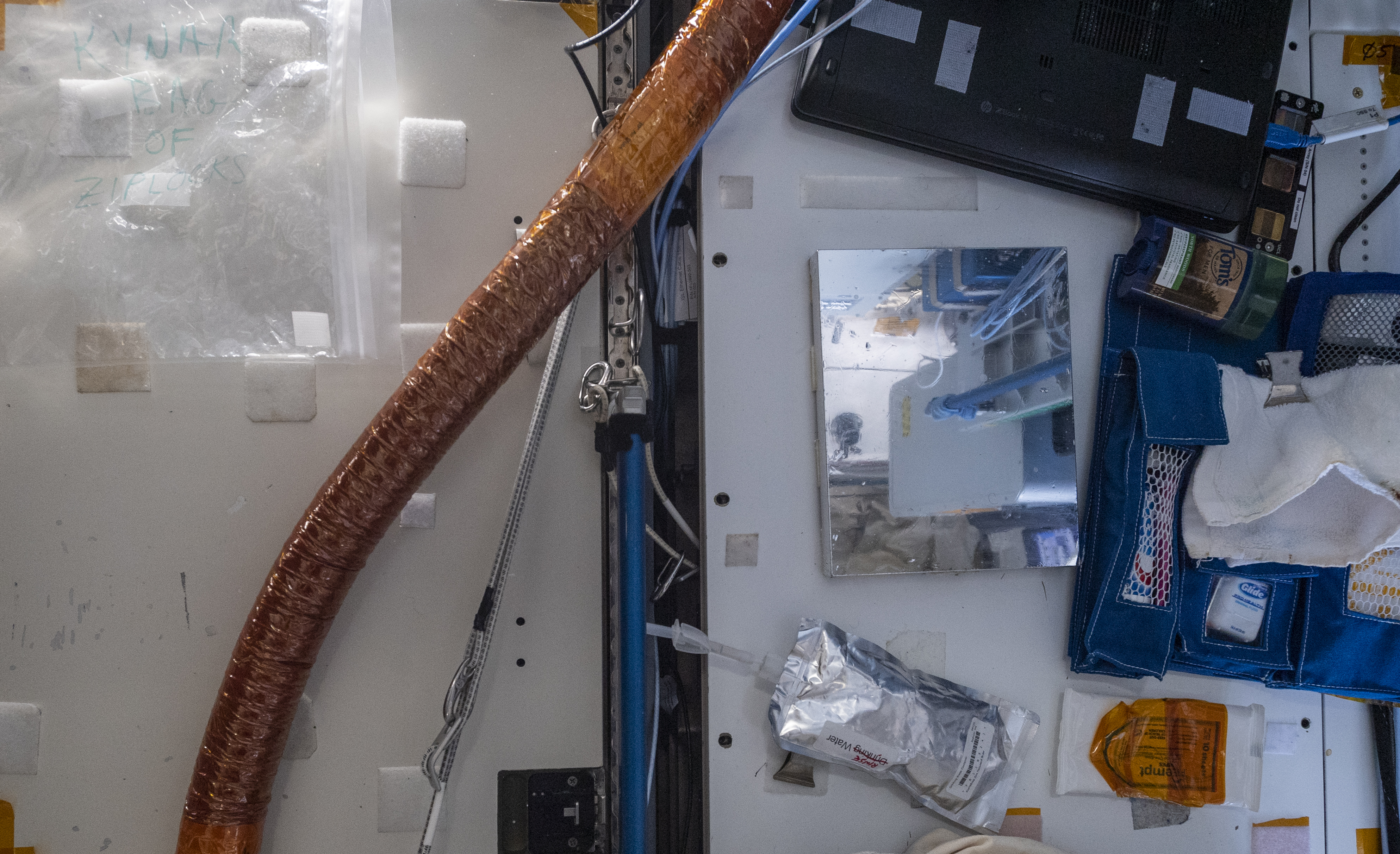

Supplement: S2 Dataset — (ZIP) [file pone.0304229.s003.zip › S05 - 39 - iss066e155018.jpg]
