# Supplementary figures and images for: Archaeology in space: The Sampling Quadrangle Assemblages Research Experiment (SQuARE) on the International Space Station. Report 1: Squares 03 and 05 (part 2 of 2)
Source: PLoS One. 2024 Aug 7;19(8):e0304229. doi: 10.1371/journal.pone.0304229 (PMC11305871; doi:10.1371/journal.pone.0304229)

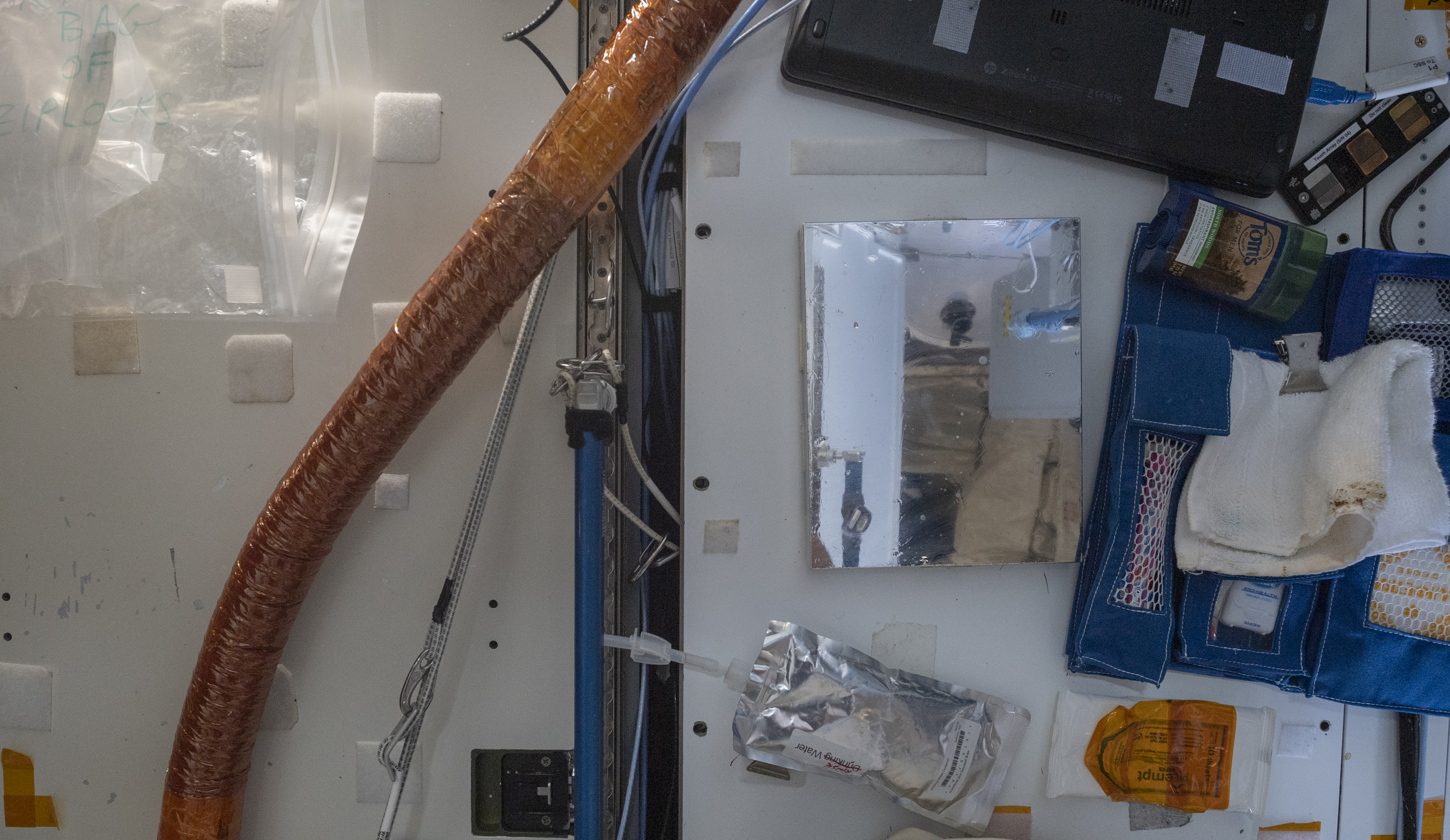

Supplement: S2 Dataset — (ZIP) [file pone.0304229.s003.zip › S05 - 40 - iss066e156058.jpg]

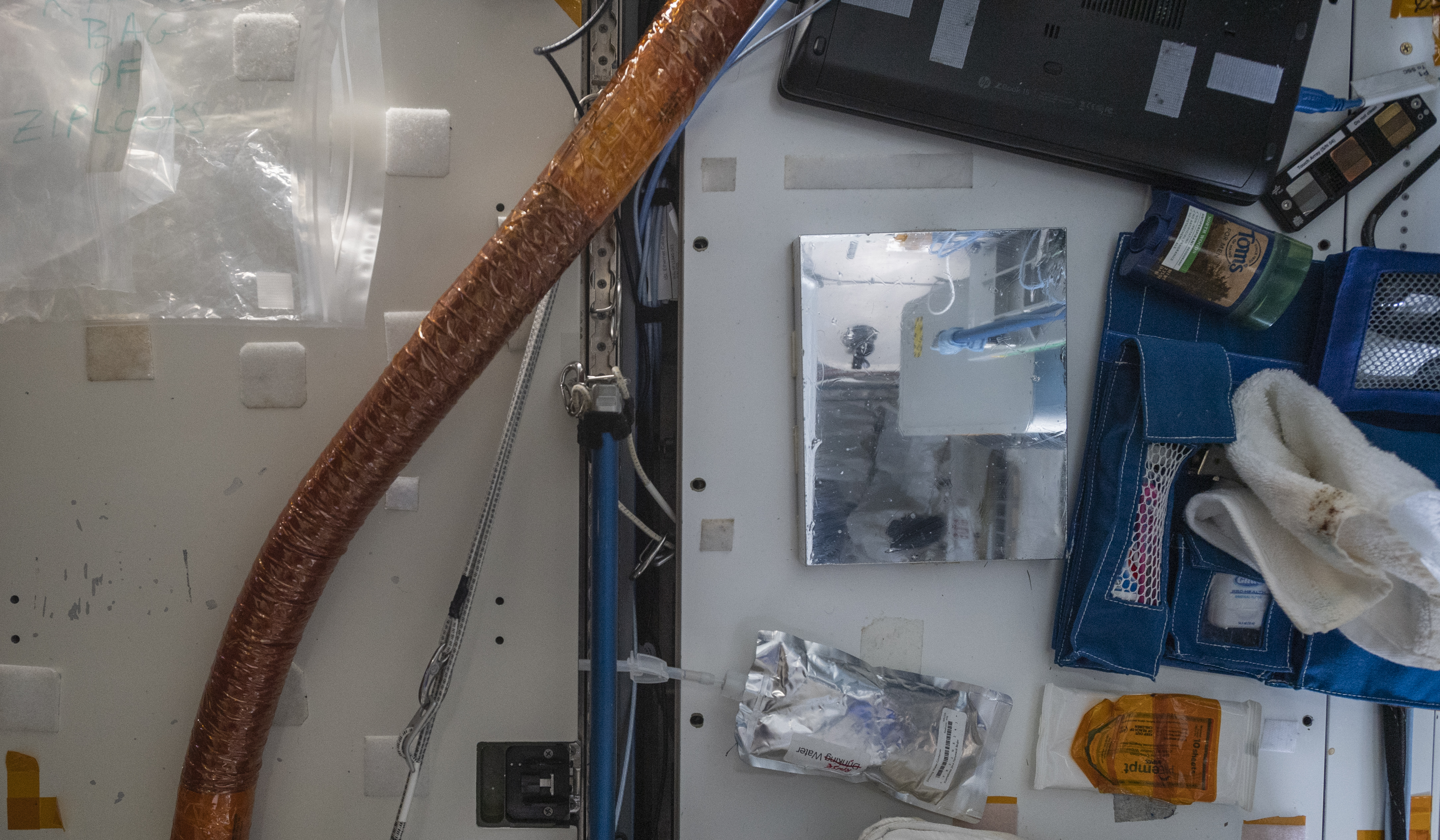

Supplement: S2 Dataset — (ZIP) [file pone.0304229.s003.zip › S05 - 41 - iss066e156464.jpg]

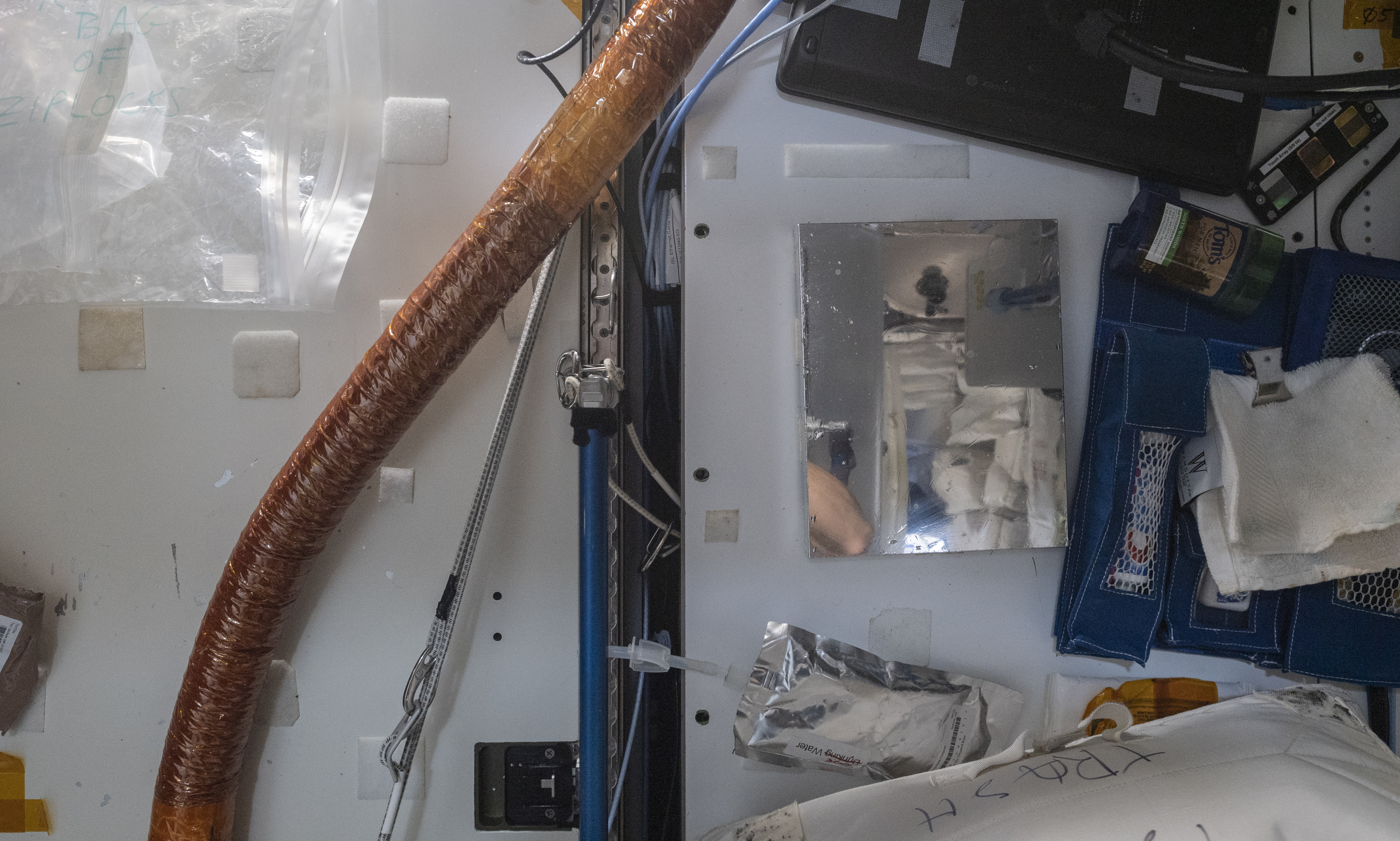

Supplement: S2 Dataset — (ZIP) [file pone.0304229.s003.zip › S05 - 42 - iss066e157068.jpg]

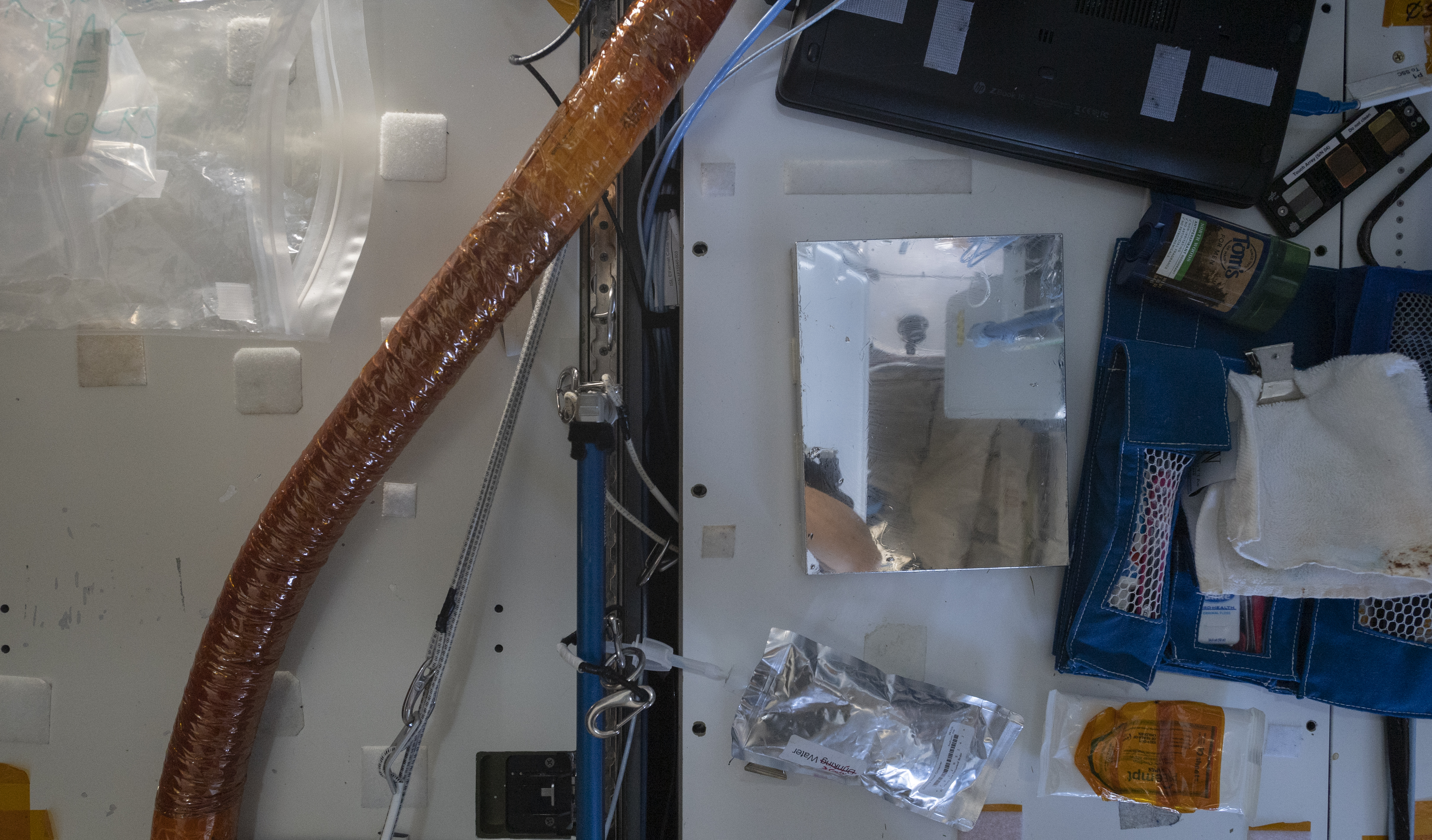

Supplement: S2 Dataset — (ZIP) [file pone.0304229.s003.zip › S05 - 43 - iss066e157149.jpg]

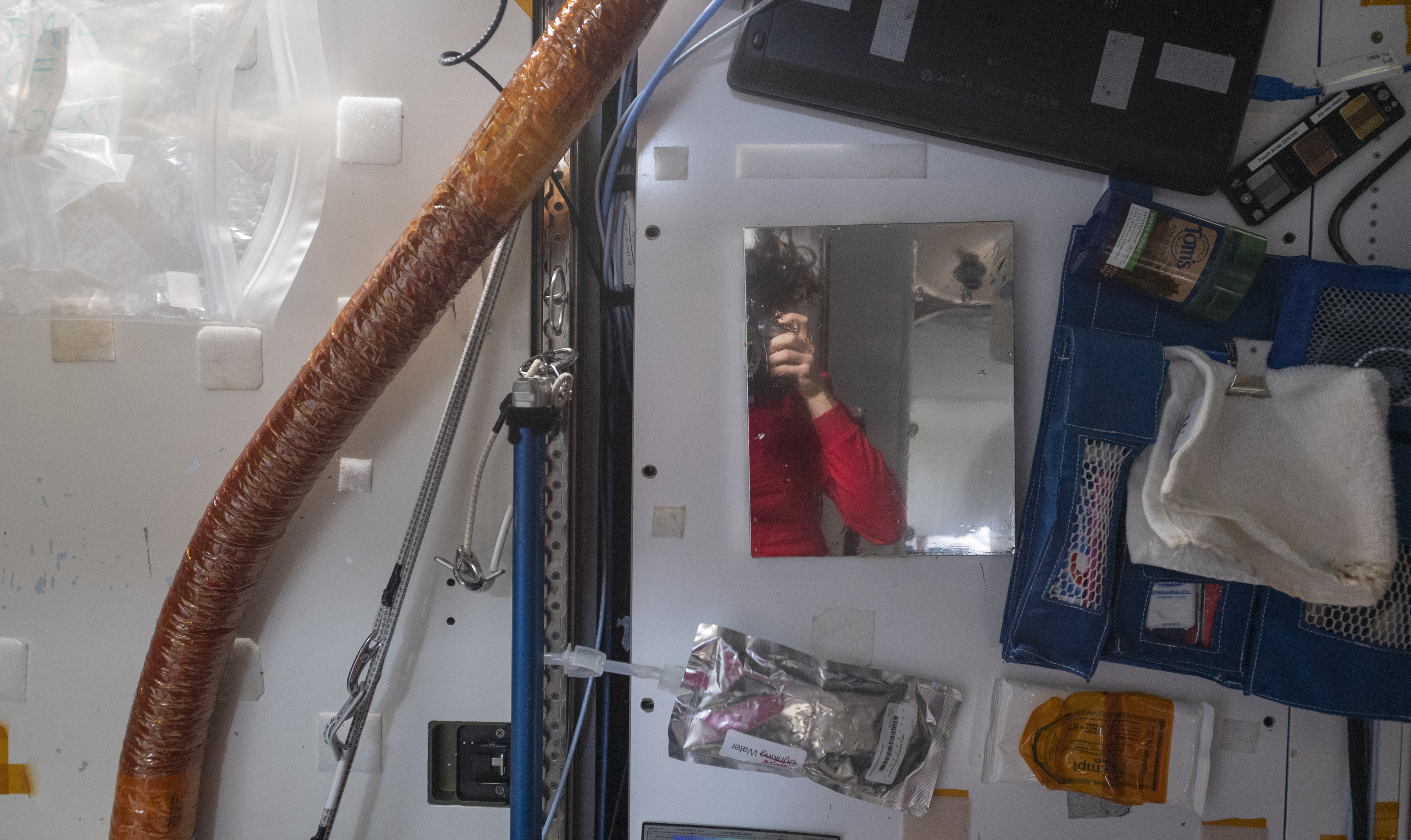

Supplement: S2 Dataset — (ZIP) [file pone.0304229.s003.zip › S05 - 44 - iss066e157139.jpg]

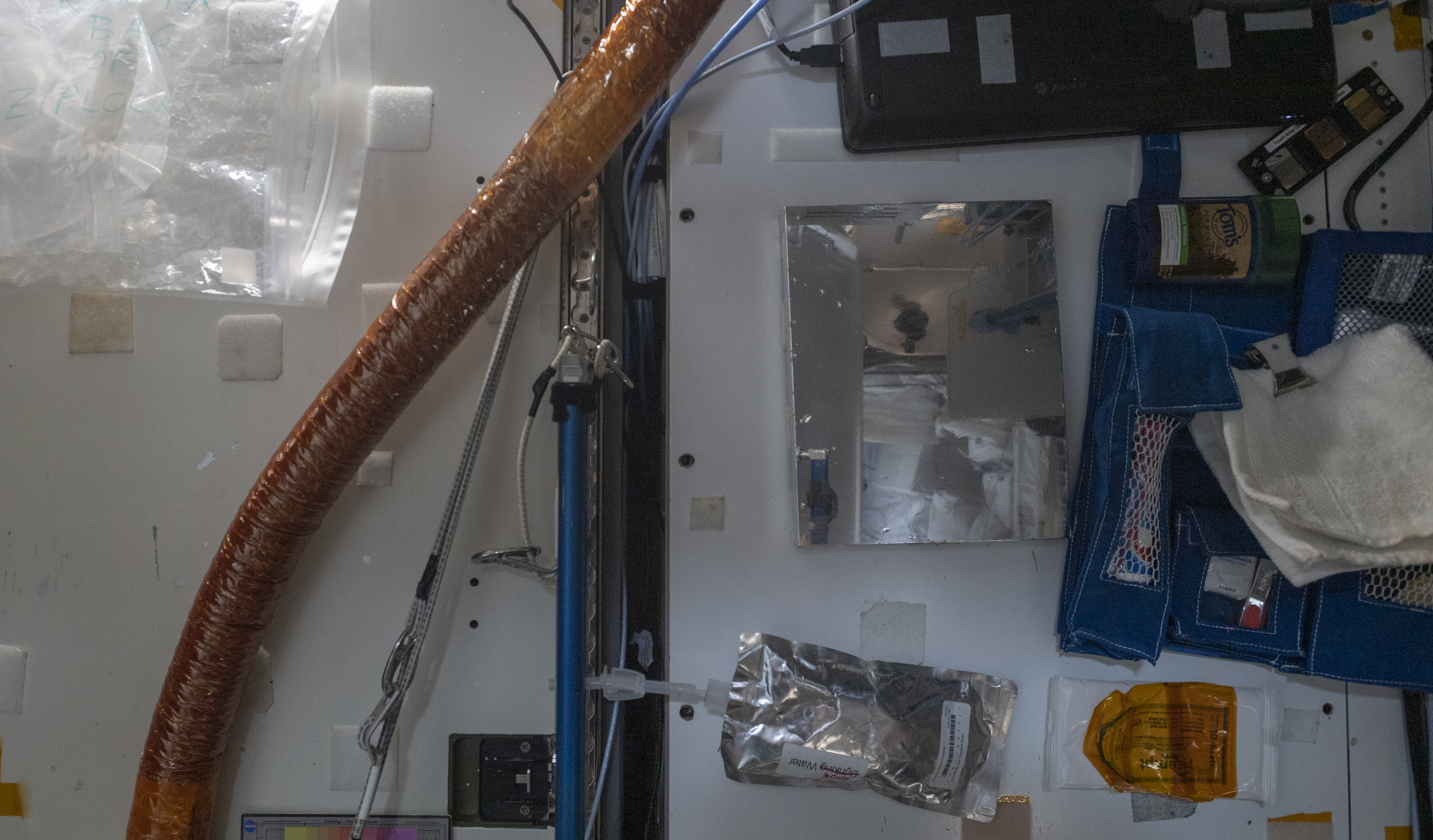

Supplement: S2 Dataset — (ZIP) [file pone.0304229.s003.zip › S05 - 45 - iss066e157613.jpg]

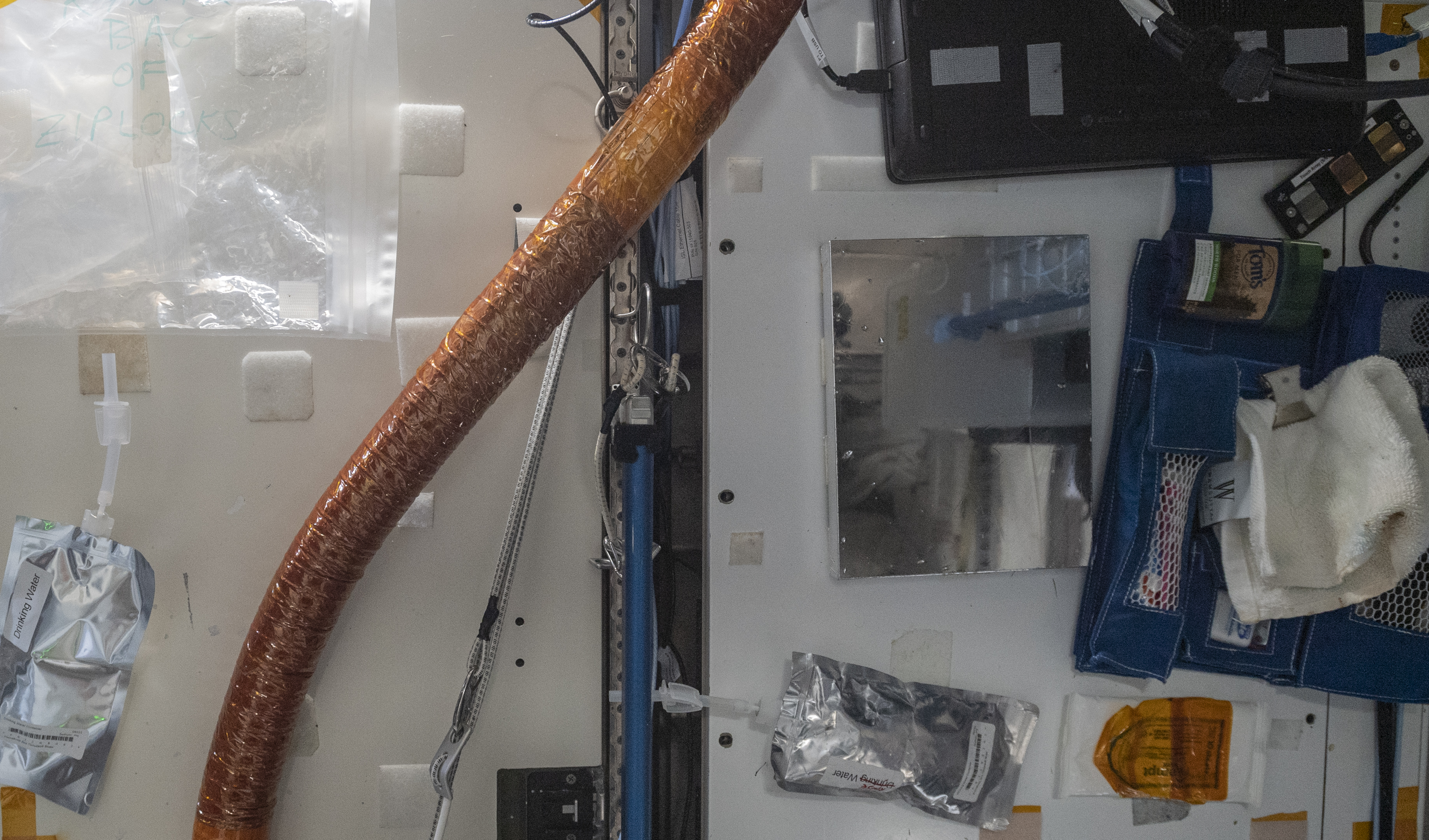

Supplement: S2 Dataset — (ZIP) [file pone.0304229.s003.zip › S05 - 46 - iss066e157924.jpg]

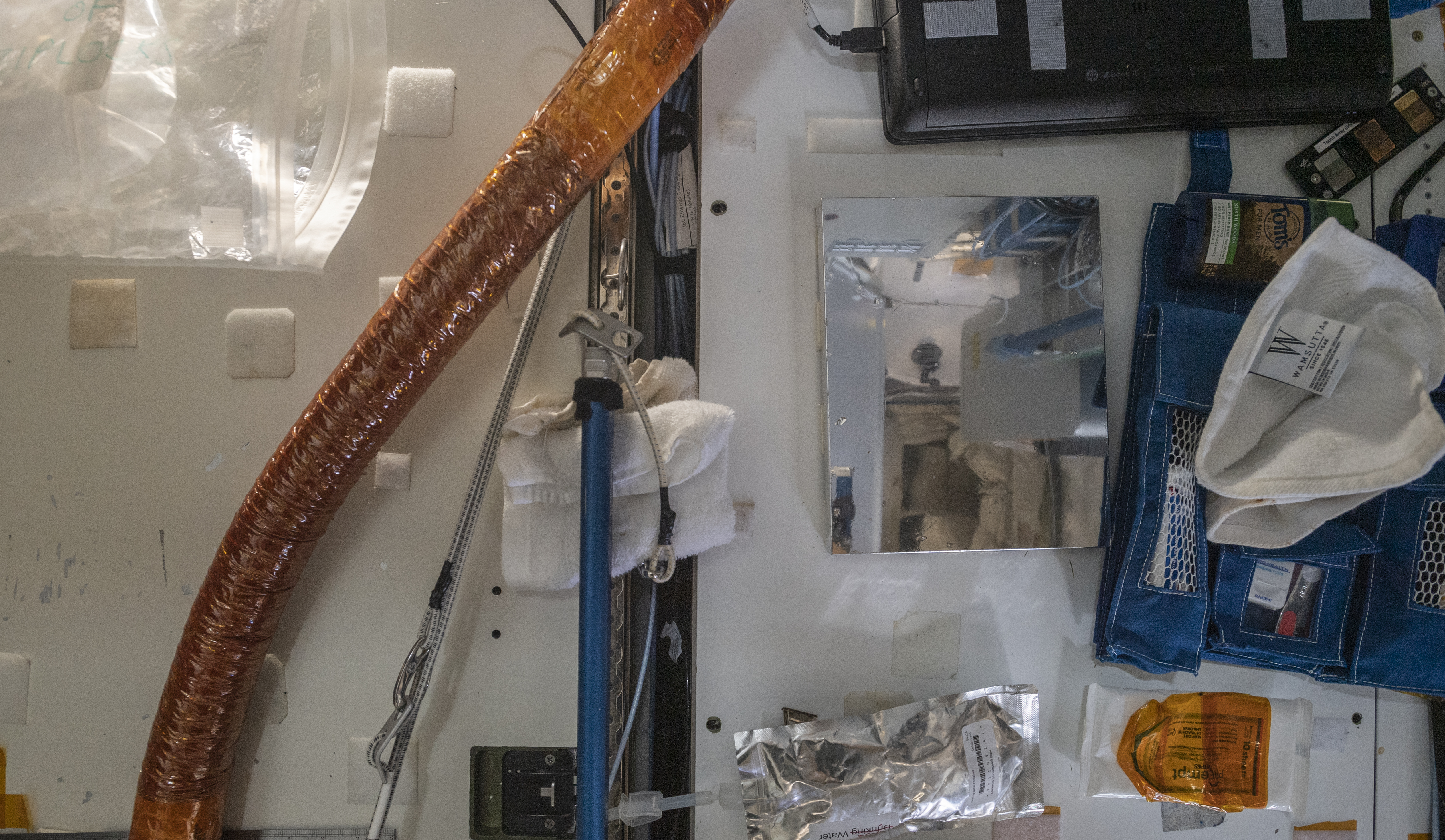

Supplement: S2 Dataset — (ZIP) [file pone.0304229.s003.zip › S05 - 47 - iss066e160726.jpg]

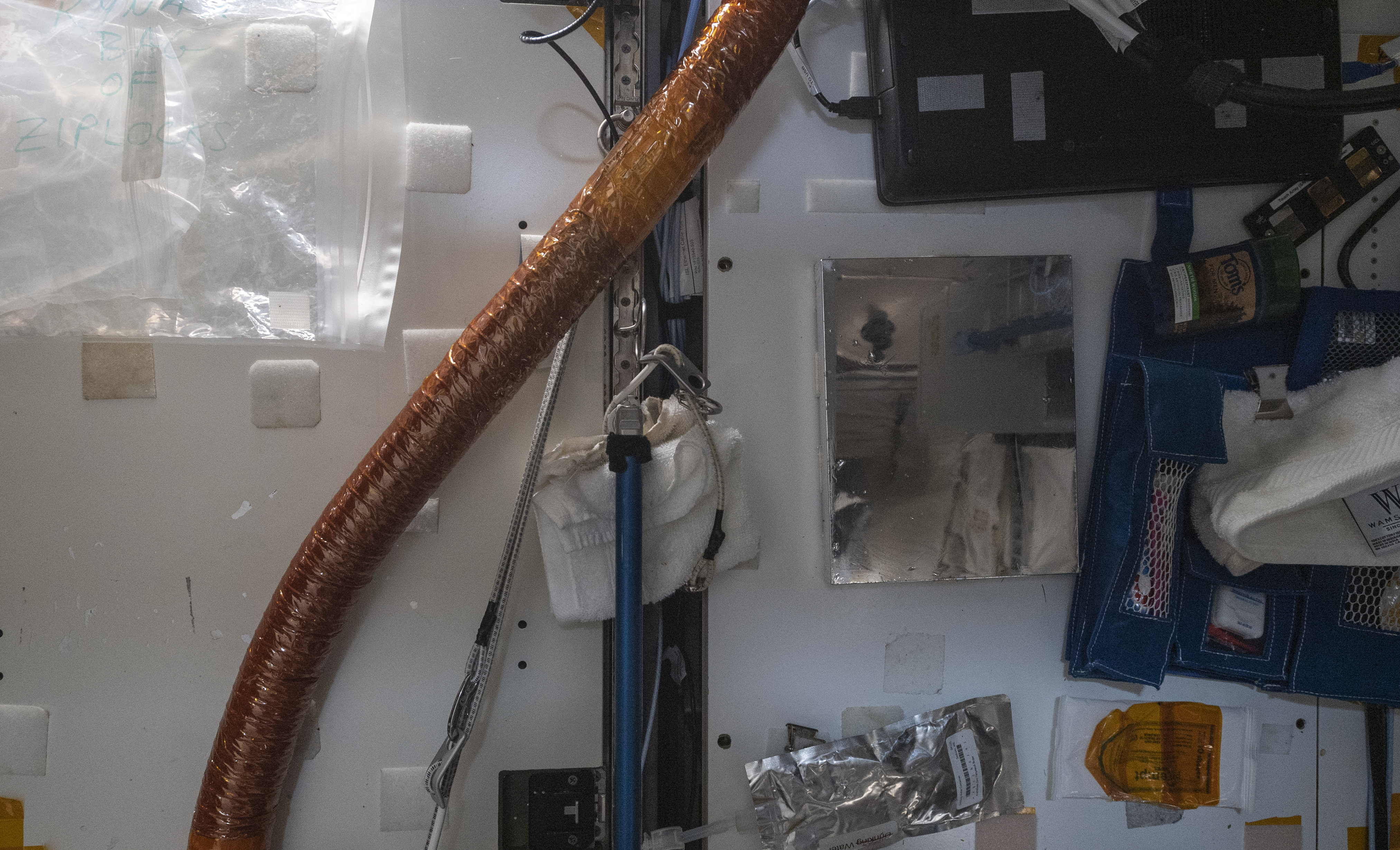

Supplement: S2 Dataset — (ZIP) [file pone.0304229.s003.zip › S05 - 48 - iss066e160941.jpg]

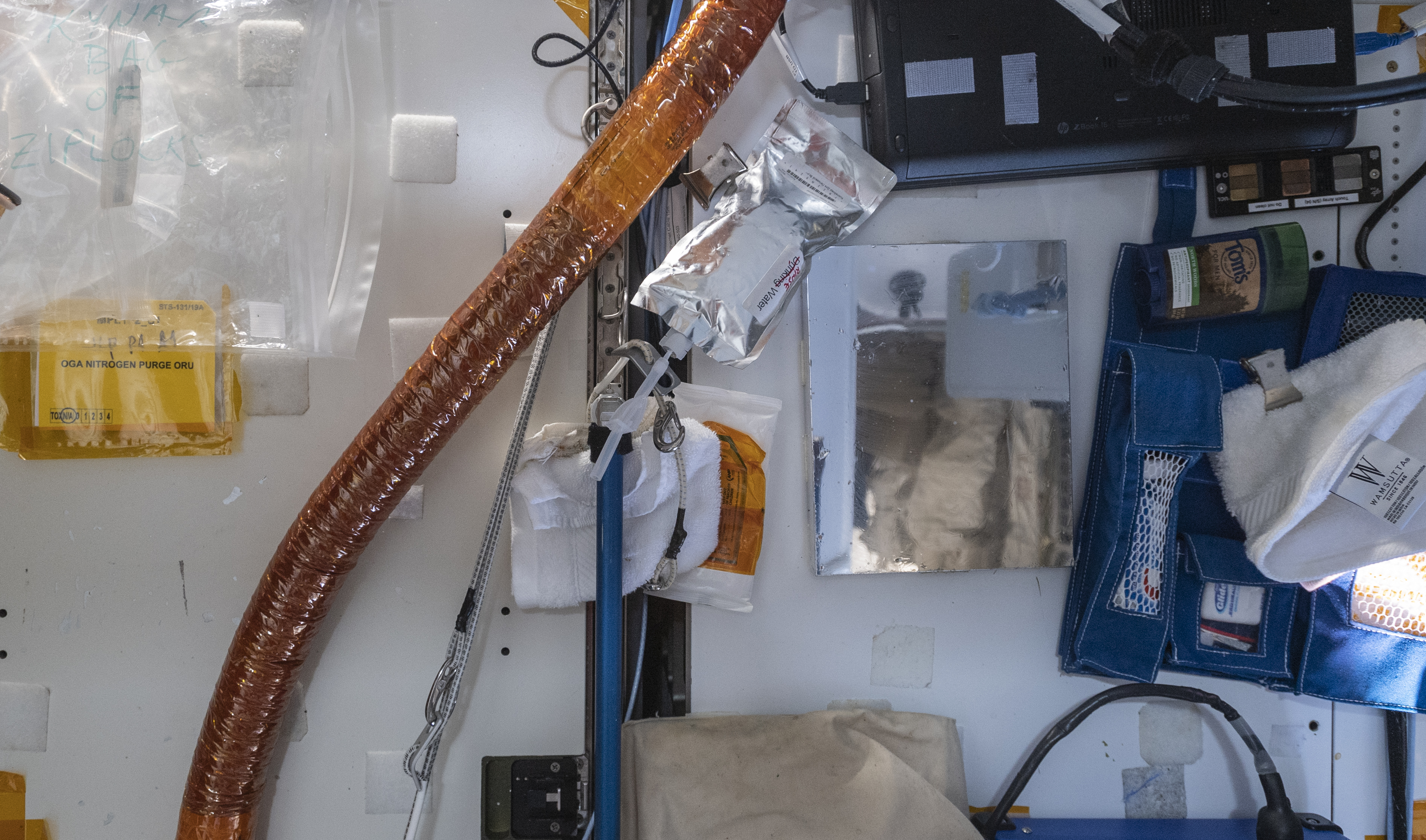

Supplement: S2 Dataset — (ZIP) [file pone.0304229.s003.zip › S05 - 49 - iss066e161300.jpg]

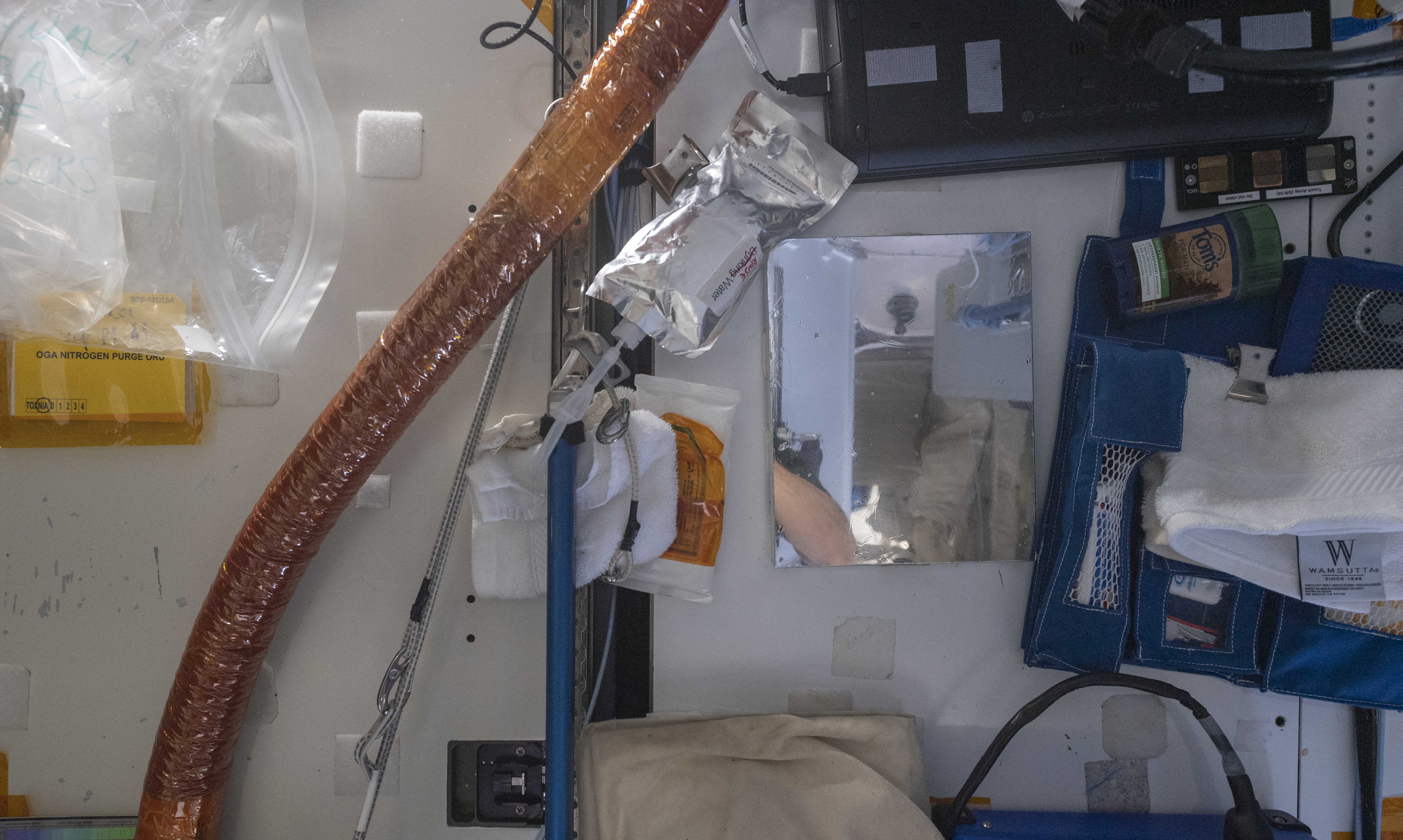

Supplement: S2 Dataset — (ZIP) [file pone.0304229.s003.zip › S05 - 50 - iss066e161510.jpg]

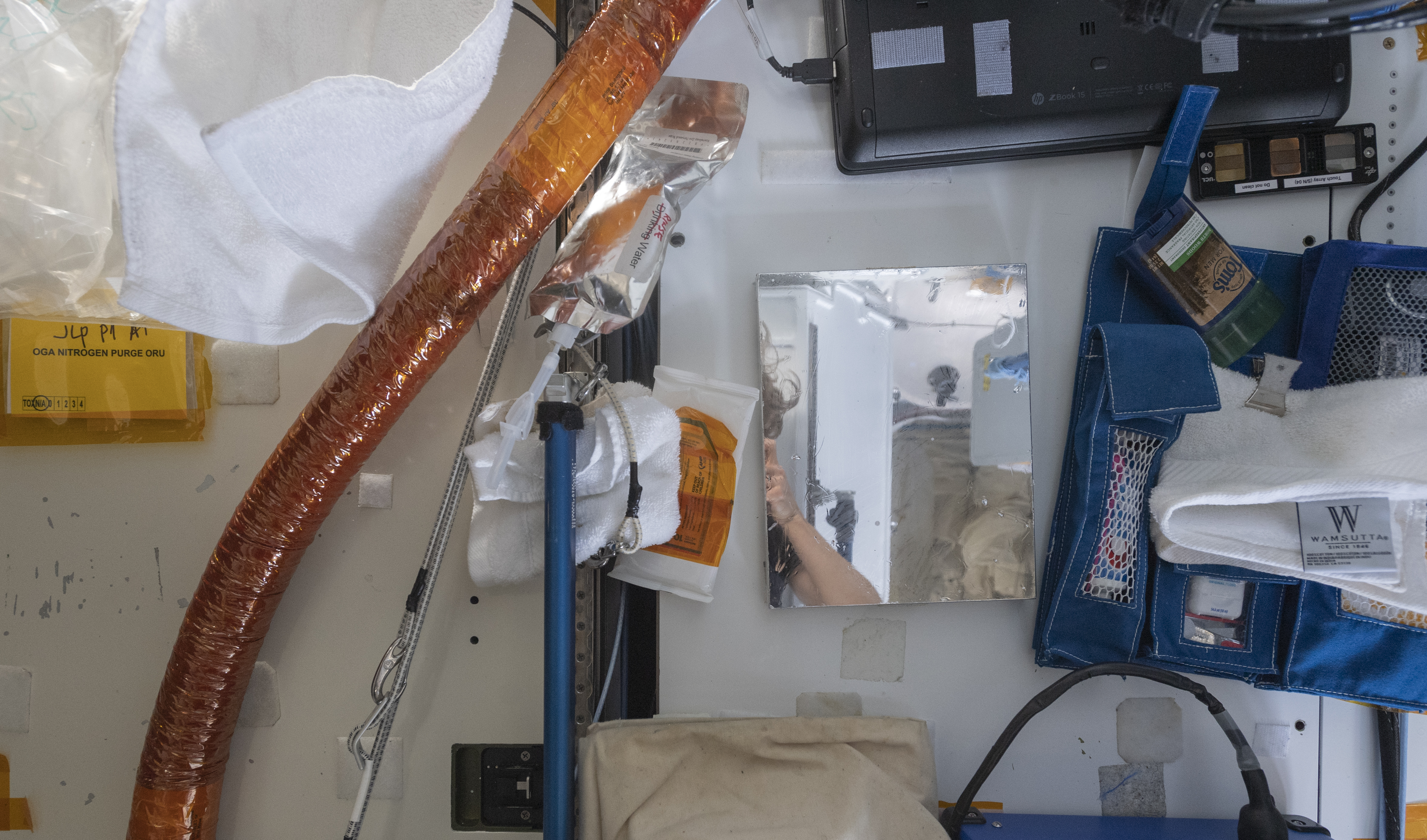

Supplement: S2 Dataset — (ZIP) [file pone.0304229.s003.zip › S05 - 51 - iss066e161494.jpg]

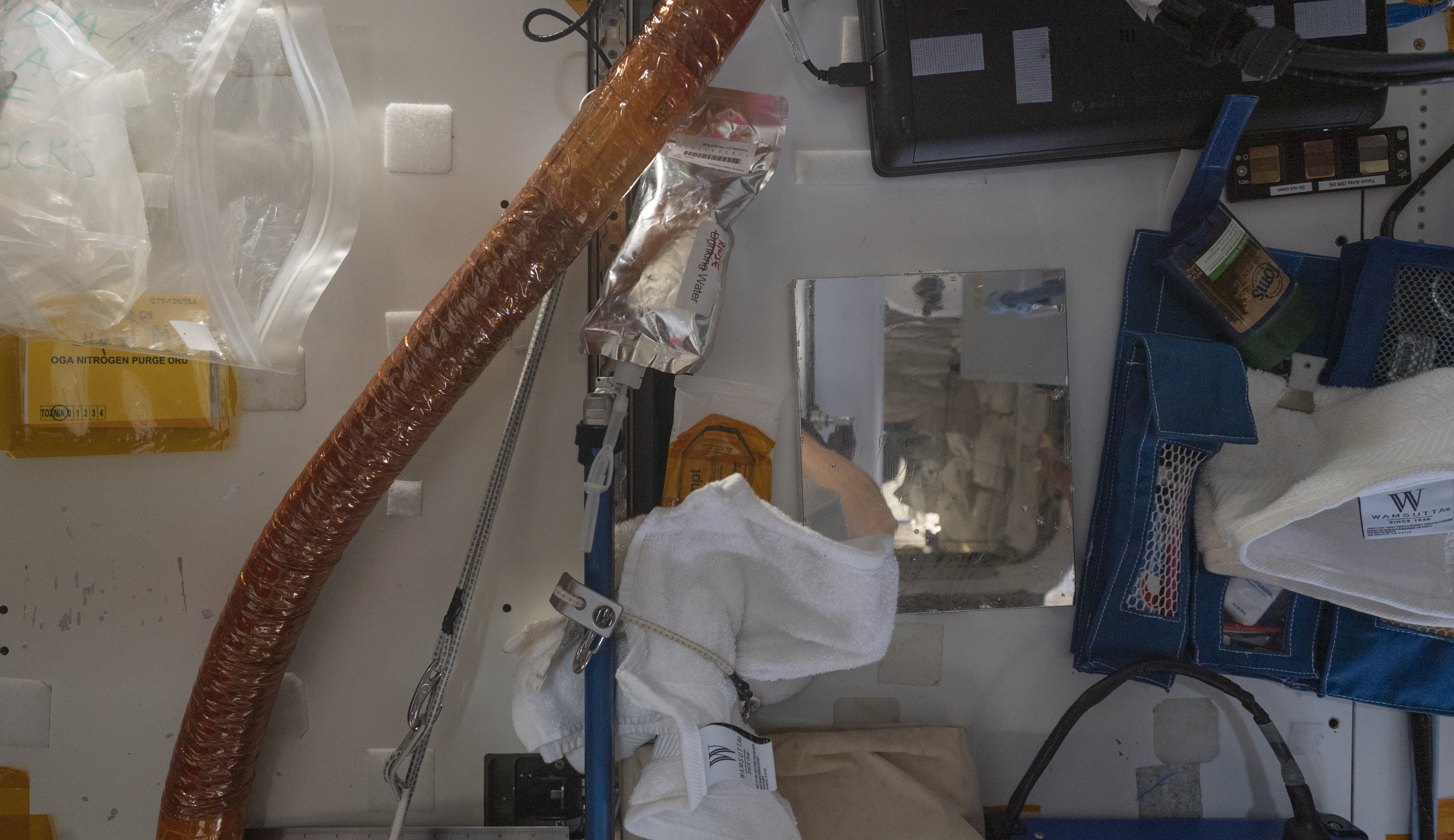

Supplement: S2 Dataset — (ZIP) [file pone.0304229.s003.zip › S05 - 52 - iss066e162139.jpg]

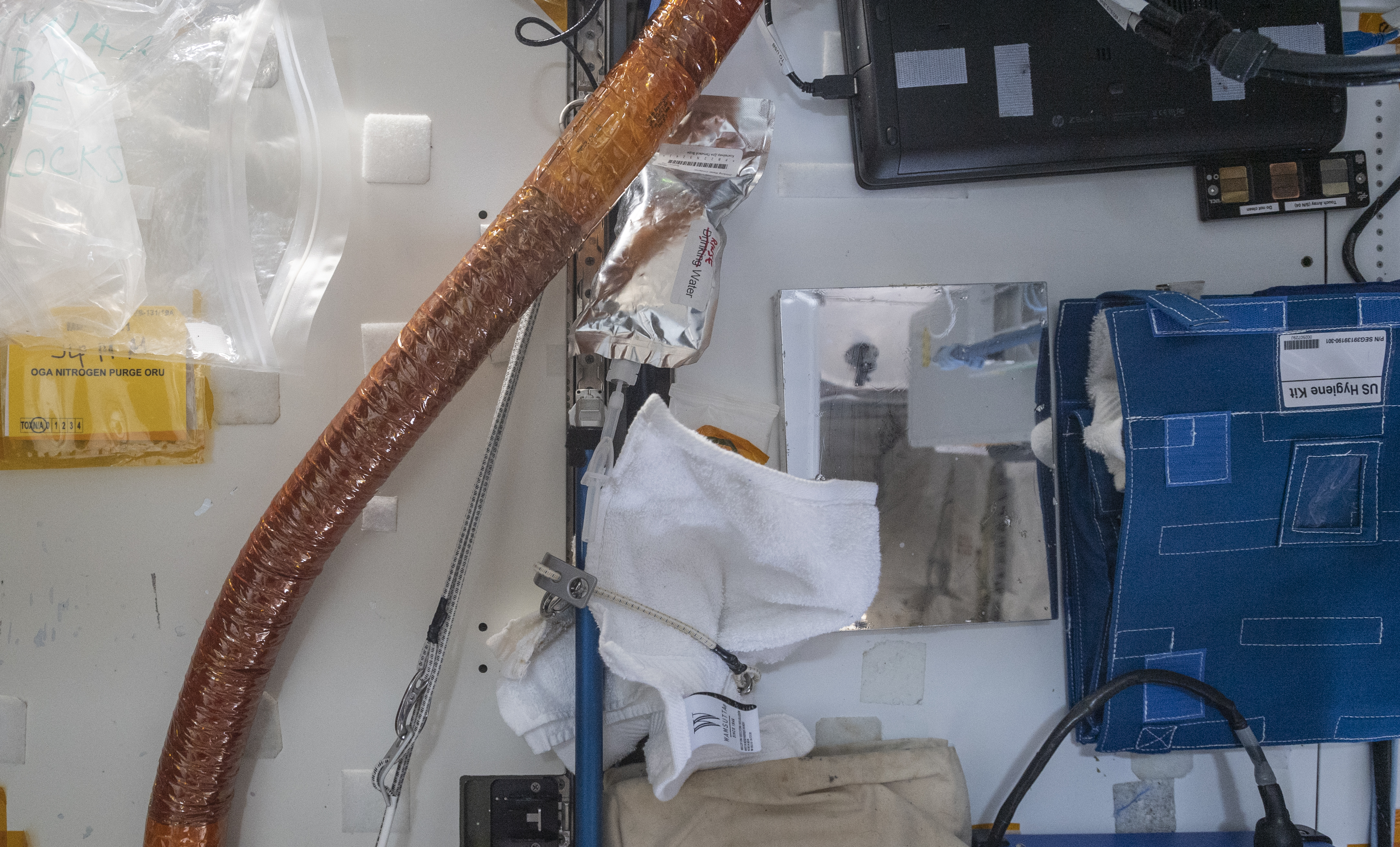

Supplement: S2 Dataset — (ZIP) [file pone.0304229.s003.zip › S05 - 53 - iss066e165123.jpg]

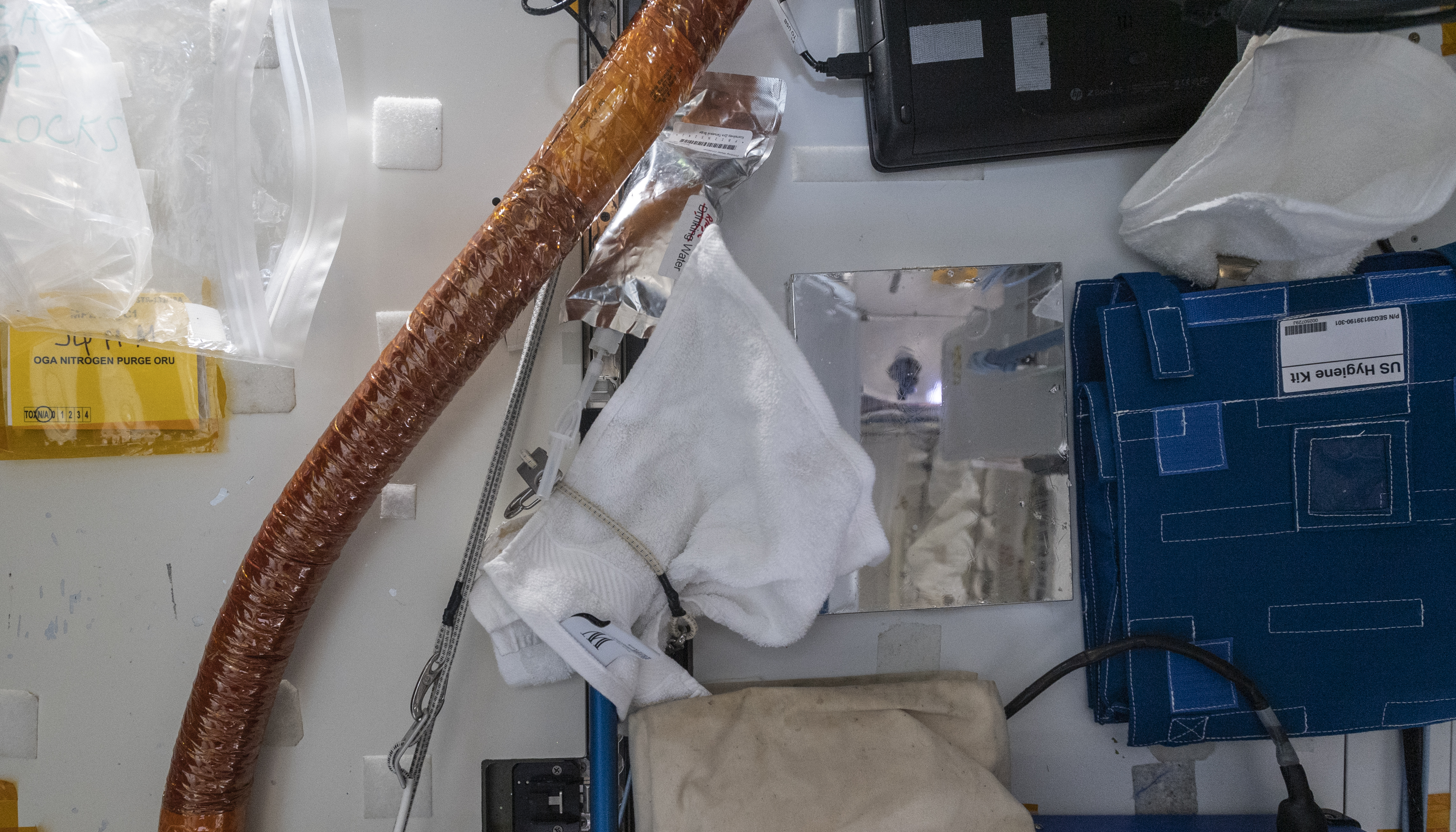

Supplement: S2 Dataset — (ZIP) [file pone.0304229.s003.zip › S05 - 54 - iss066e167413.jpg]

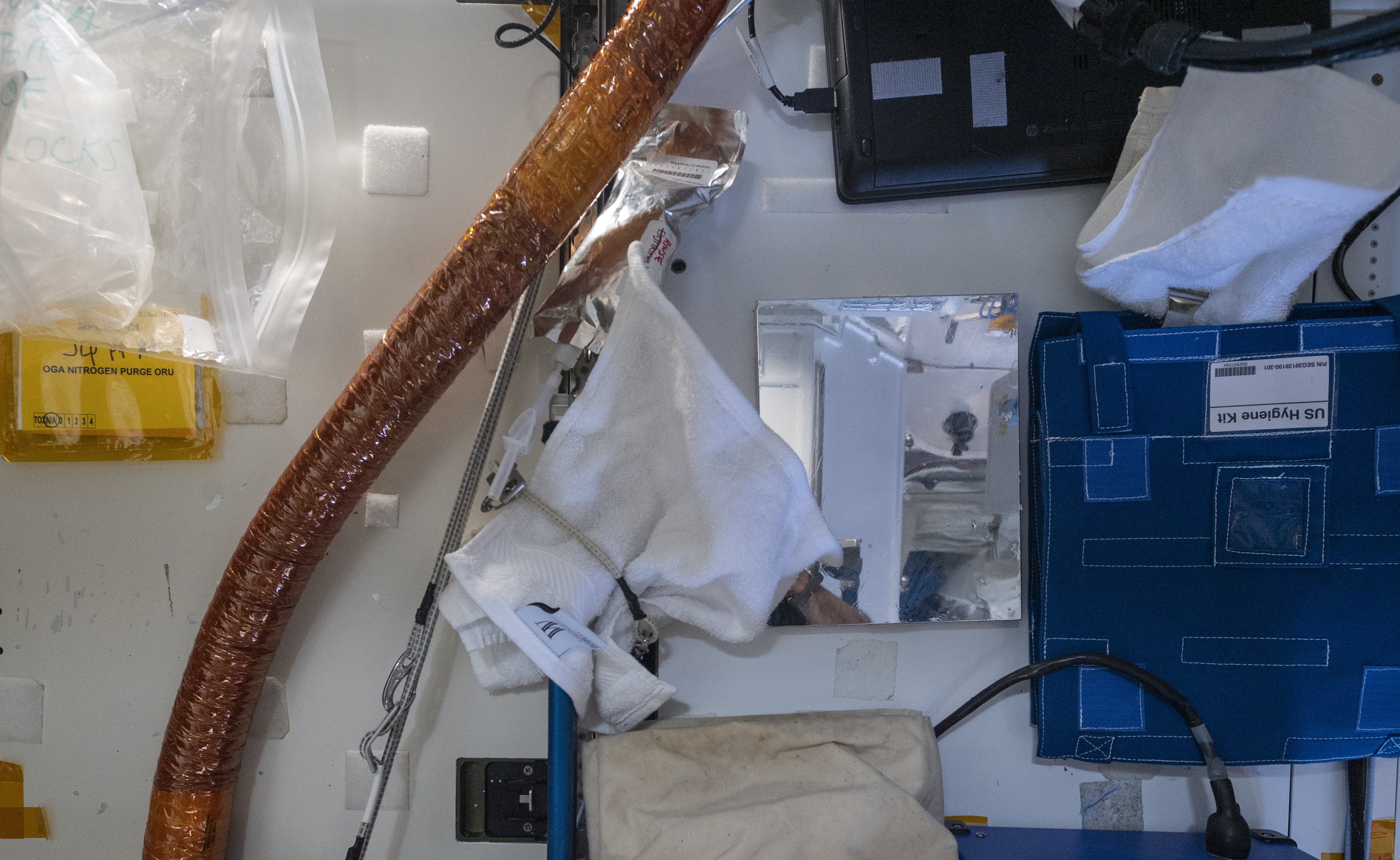

Supplement: S2 Dataset — (ZIP) [file pone.0304229.s003.zip › S05 - 55 - iss066e172267.jpg]

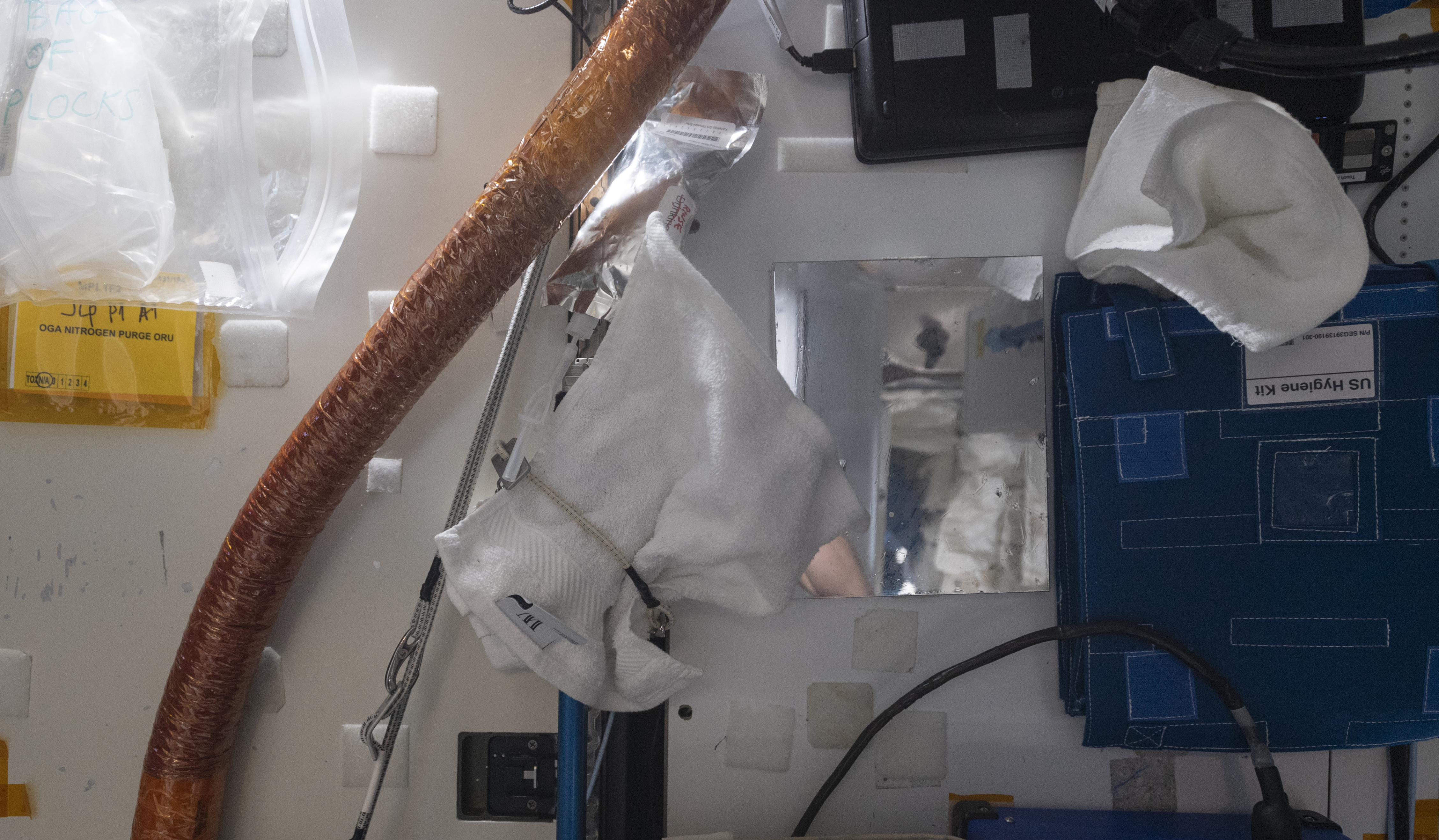

Supplement: S2 Dataset — (ZIP) [file pone.0304229.s003.zip › S05 - 56 - iss066e173225.jpg]

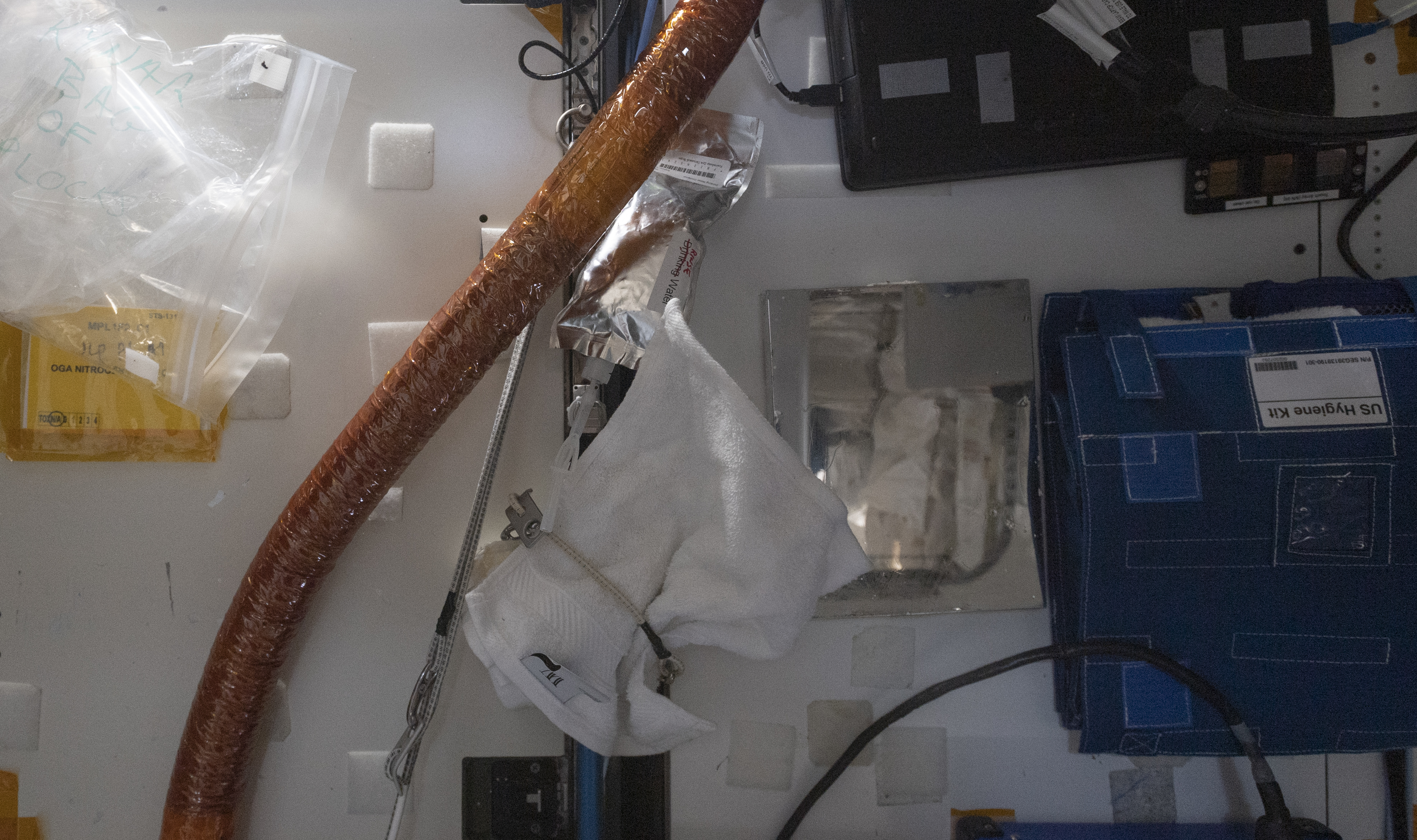

Supplement: S2 Dataset — (ZIP) [file pone.0304229.s003.zip › S05 - 57 - iss066e173218.jpg]

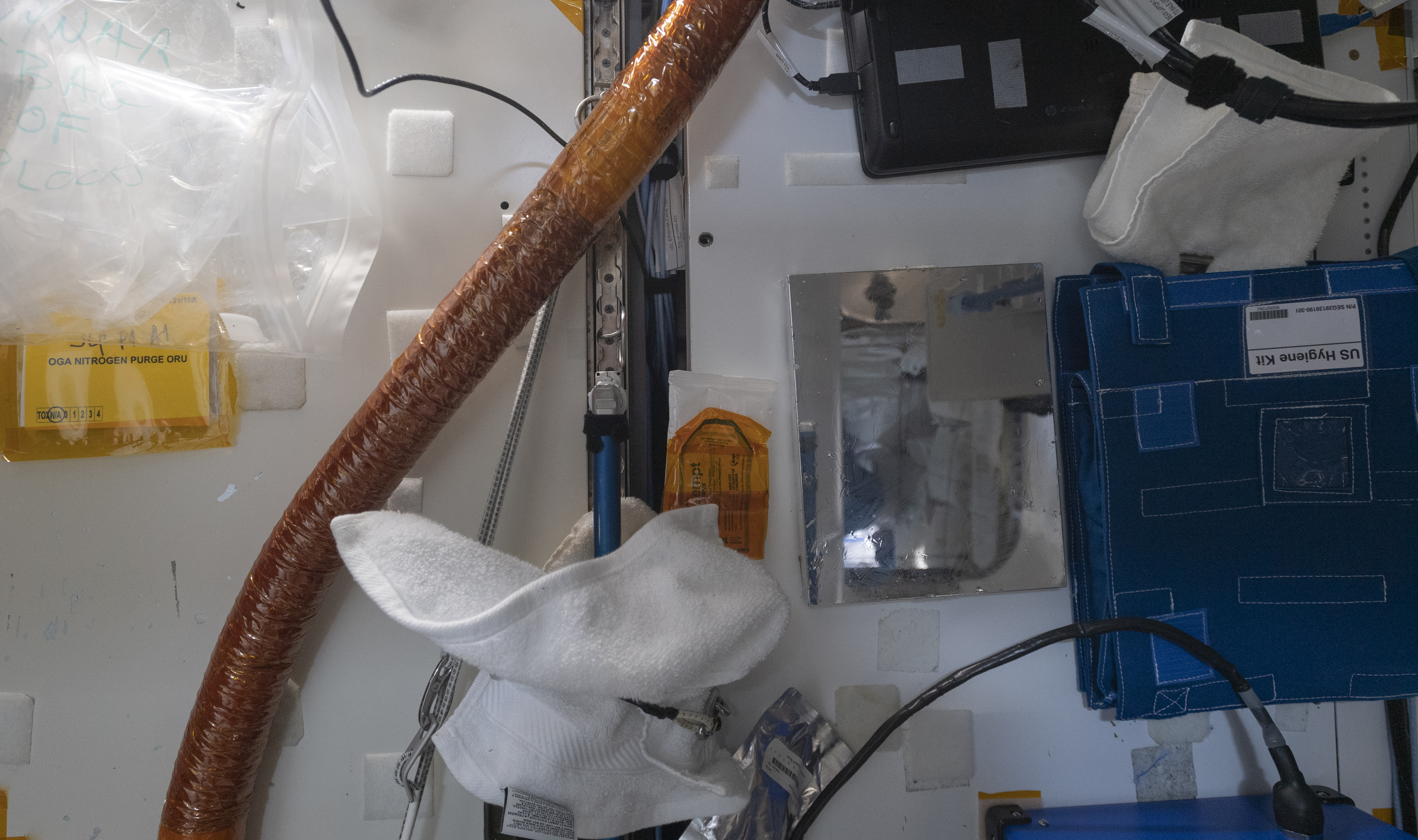

Supplement: S2 Dataset — (ZIP) [file pone.0304229.s003.zip › S05 - 58 - iss066e173135.jpg]

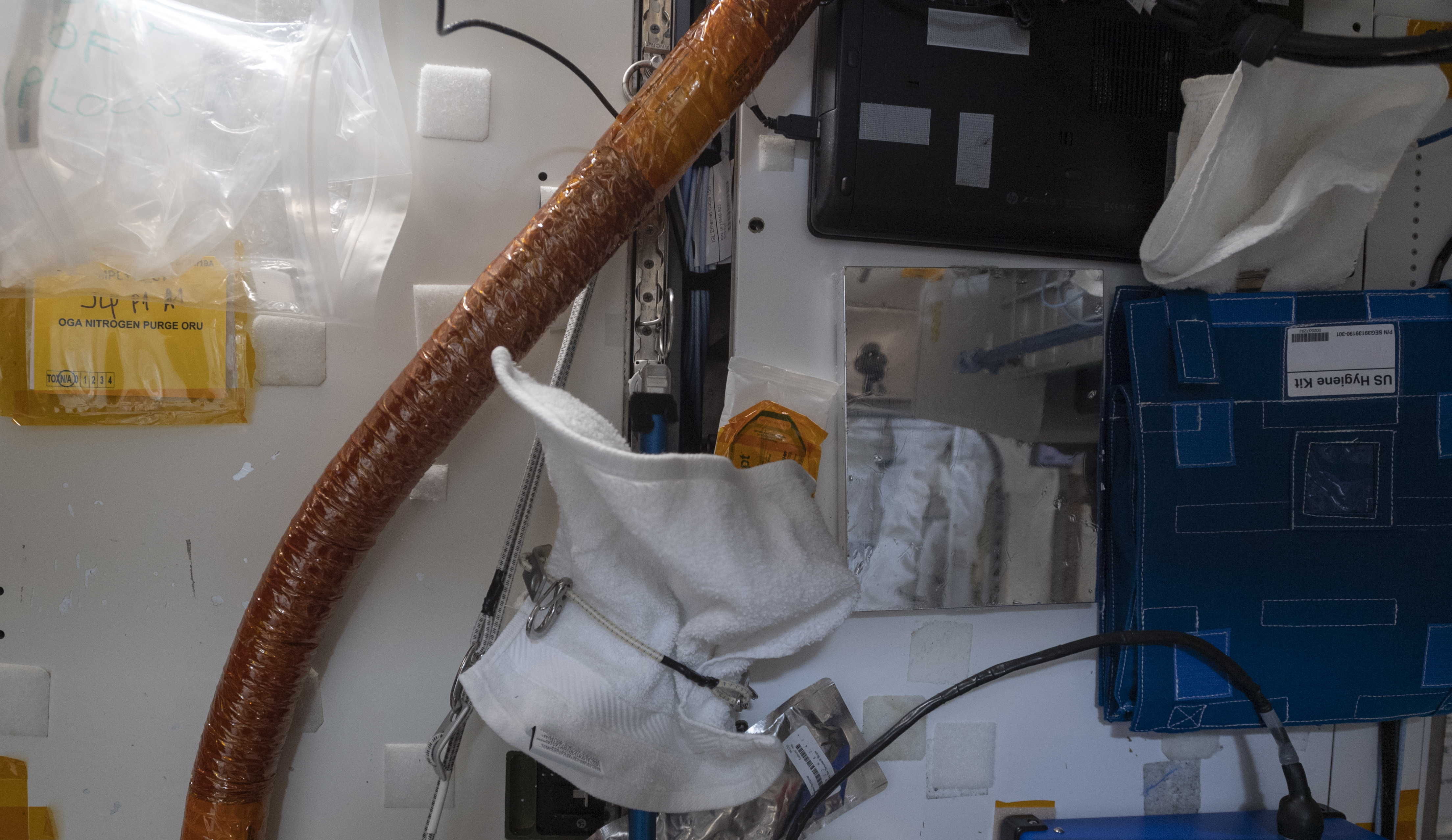

Supplement: S2 Dataset — (ZIP) [file pone.0304229.s003.zip › S05 - 59 - iss066e173148.jpg]

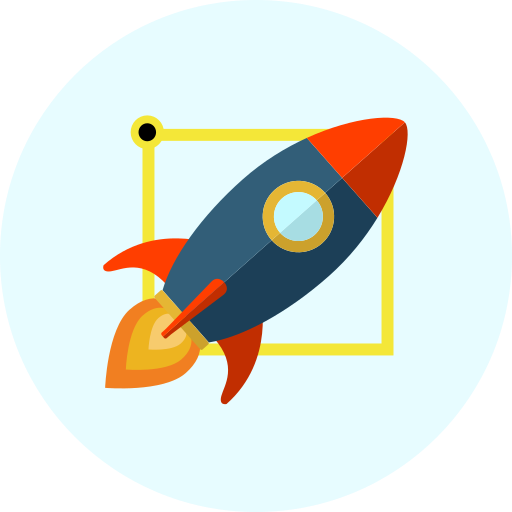

Supplement: S1 File — The archived version of the repository is at Zenodo, DOI: 10.5281/zenodo.10648399. (ZIP) [file pone.0304229.s005.zip › MRE-RocketAnno-master/icon.png]

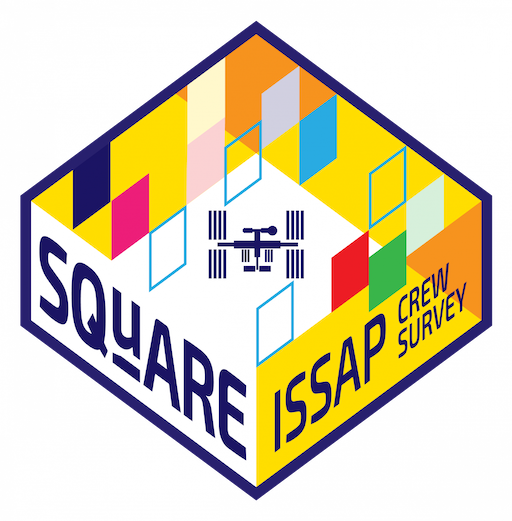

Supplement: S2 File — The code is available in the ‘SQuARE-notebooks’ repository on Github.com in the ‘notebooks’ subfolder at https://github.com/issarchaeologicalproject/SQuARE-notebooks/tree/main; archived version of the repository is at Zenodo, DOI: 10.5281/zenodo.10654812. The software can be run online in the Google Colab environment (https://colab.research.google.com) or any system running Jupyter Notebooks (https://jupyter.org/). (ZIP) [file pone.0304229.s006.zip › SQuARE-notebooks-main/square.png]
