# Supplementary material for: Archaeology in space: The Sampling Quadrangle Assemblages Research Experiment (SQuARE) on the International Space Station. Report 1: Squares 03 and 05
Source: PLoS One. 2024 Aug 7;19(8):e0304229. doi: 10.1371/journal.pone.0304229 (PMC11305871; doi:10.1371/journal.pone.0304229)
Supplement: S1 File — The archived version of the repository is at Zenodo, DOI: 10.5281/zenodo.10648399. (ZIP) [file pone.0304229.s005.zip › MRE-RocketAnno-master/src/app.html]

%sveltekit.head%


%sveltekit.body%
